# Nf2/FGFR1/AKT axis directs cranial neural crest–derived skull morphogenesis via collagen synthesis and trafficking

**DOI:** 10.1172/jci.insight.191112

**Published:** 2025-09-23

**Authors:** Yuping Huang, Junguang Liao, Panpan Shen, Yiliang He, Fuju Sun, Qi Zhang, Changlin Zheng, Xingen Zhang, Haibo Li, Guiqian Chen

**Affiliations:** 1 Department of Biopharmaceutics, Zhejiang Provincial Engineering Research Center of New Technologies and Applications for Targeted Therapy of Major Diseases, College of Life Science and Medicine, Zhejiang Sci-Tech University, Hangzhou, China.; 2 Department of Orthopedics, Jiaxing Key Laboratory for Minimally Invasive Surgery in Orthopedics & Skeletal Regenerative Medicine, Zhejiang Rongjun Hospital, Jiaxin, China.; 3 The Central Laboratory of Birth Defects Prevention and Control, Ningbo Women and Children’s Hospital, Ningbo Key Laboratory for the Prevention and Treatment of Embryogenic Diseases, The Affiliated Women and Children’s Hospital of Ningbo University, Ningbo, China.

**Keywords:** Bone biology, Cell biology, Bone development

## Abstract

Cranial neural crest cells (CNCs) play a critical role in craniofacial bone morphogenesis, engaging in intricate interactions with various molecular signals to ensure proper development, yet the molecular scaffolds coordinating these processes remain incompletely defined. Here, we identify neurofibromin 2 (*Nf2*) as a critical regulator to direct CNC-derived skull morphogenesis. Genetic ablation of *Nf2* in murine CNCs causes severe craniofacial anomalies, featuring declined proliferation and increased apoptosis in osteoprogenitors, impaired type I collagen biosynthesis and trafficking, and aberrant osteogenic mineralization. Mechanistically, we uncover that Nf2 serves as a molecular linker that individually interacts with FGF receptor 1 (FGFR1) and Akt through spatially segregated phosphor-sites, and structural modeling and mutagenesis identified Ser10 and Thr230 as essential residues, with Thr230 mutation selectively ablating Akt binding while preserving FGFR1 association. Strikingly, Akt inhibition phenocopied *Nf2* deficiency, reducing collagen production and Nf2 phosphorylation, whereas phospho-mimetic *Nf2* (T230D) rescued CNC-derived osteogenic defects in *Nf2*-mutant animals. Our findings underscore the physiological significance of Nf2 as a phosphorylation-operated scaffold licensing the FGFR1/AKT axis to regulate collagen type I biogenesis and trafficking, ensuring normal CNC-derived osteogenesis and craniofacial bone development, thus exposing the Nf2/FGFR1/AKT signaling axis as a therapeutic target and promising advancements in treatment of craniofacial anomalies.

## Abstract 2

Our study identified the Nf2-FGFR1-Akt axis as a critical regulator of collagen type I synthesis and trafficking in CNC-derived osteoblasts, ensuring proper osteogenesis and skull morphogenesis.

## Graphical abstract

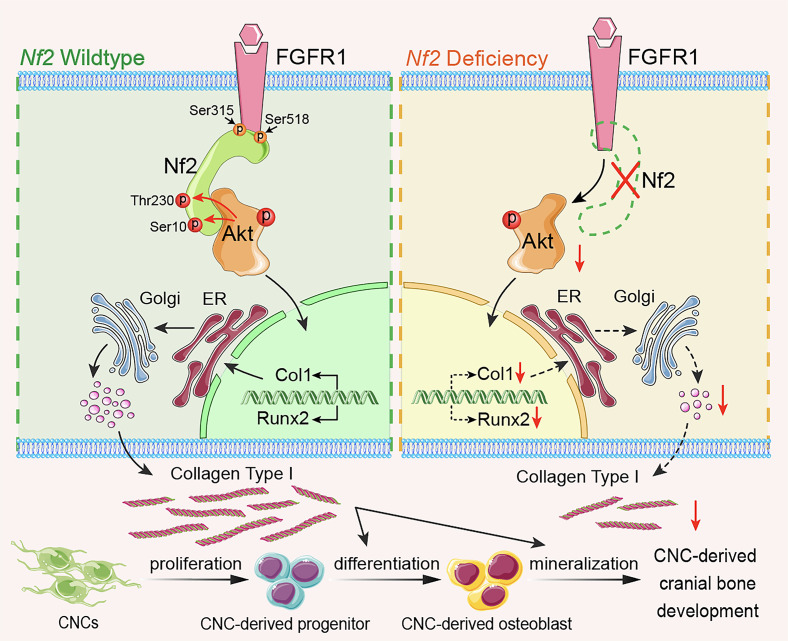

## Introduction

Cranial neural crest cells (CNCs) are a unique population of stem cells that delaminate from the dorsal neural tube and migrate to diverse locations, driving cell differentiation and orchestrating the formation of multiple organs, including the craniofacial skeleton (1–4). The pivotal role of CNCs in craniofacial bone development has been well documented across species, with most craniofacial bones, such as the frontal bone (5–7), originating from CNCs, and others, like the parietal bones, deriving from paraxial mesoderm (8–11). Transgenic mouse models, including *Wnt1-Cre* and *P0-Cre*, have been instrumental in tracing and characterizing CNC lineages (6). The precise regulation of CNC lineage commitment, differentiation, and patterning during embryogenesis is critical, as disruptions in these processes lead to congenital craniofacial anomalies (12–15), underscoring the importance of CNCs in both development and therapeutic interventions for craniofacial defects.

The fibroblast growth factor (FGF) signaling pathway, comprising 22 ligands and 4 receptors (FGFR1-4), along with downstream effectors such as PI3K-AKT, PLCγ, STAT, and MAPK (16–18), plays a central role in CNC-derived osteogenic differentiation (19), cranial bone growth (19, 20), and skull defect repair (21). *FGFR1-3* mutations are implicated in craniofacial dysostosis syndromes (22–24), including craniosynostosis and cleft syndromes (25, 26), highlighting the pathway’s importance in craniofacial morphogenesis. *FGFR1* deficiency in CNCs leads to ectopic osteogenesis (27) and severe craniofacial malformations (28), while *FGFR2-S252W* mutation induces premature cranial suture closure (29) and craniosynostosis through Runx2-dependent osteogenic programs (30). These findings emphasize precise spatiotemporal regulation of FGF signaling is indispensable for proper cranial neural crest–mediated osteogenesis and skull morphogenesis, with even minor perturbations leading to profound craniofacial malformations.

Neurofibromin 2 (*Nf2*), encoding a protein called Merlin, a well-characterized tumor suppressor (31–33), undergoes dynamic phosphorylation-dependent regulation (e.g., Ser10, Thr230, Ser315, Ser518) by kinases such as PKA (34–36), PI3K/AKT (37), and p21-activated kinase (PAK) (38) to control cell proliferation and morphogenesis. *Nf2* is highly expressed in developing brains and migrating CNCs (39, 40), which is a vital cell population for skull morphogenesis. For instance, *Nf2* deficiency using *Wnt1-Cre* in CNCs disrupts dorsal root ganglion expansion (41) and tongue formation (42), and its loss using *Nestin-Cre* caused defects in tissue fusion (43). Notably, *Nf2* loss using *Mx1-Cre* in hematopoietic stem cells increases trabecular bone mass and marrow vascularity and VEGF production (44, 45). Additionally, our study showed that *Nf2* ablation in skeletal mesenchymal cells using *Prx1-Cre* results in defective cranial bone ossification (46) and long bone development (47). How Nf2 specifically coordinates CNC-derived skull development through a molecular mechanism remains poorly understood.

In this study, through neural crest–specific *Nf2* ablation (*Wnt1-Cre*), we uncover Nf2’s essential role as a phosphorylation-dependent scaffold that spatiotemporally couples FGFR1/AKT signaling to orchestrate type I collagen biogenesis and vesicular trafficking during craniofacial osteogenesis. Our findings not only establish Nf2 as a critical molecular nexus in CNC-derived skeletal development but also pinpoint its therapeutic potential for treatment of craniofacial deformities.

## Results

### Nf2 ablation in CNCs results in severe abnormalities of craniofacial bones.

**Figure 1:**
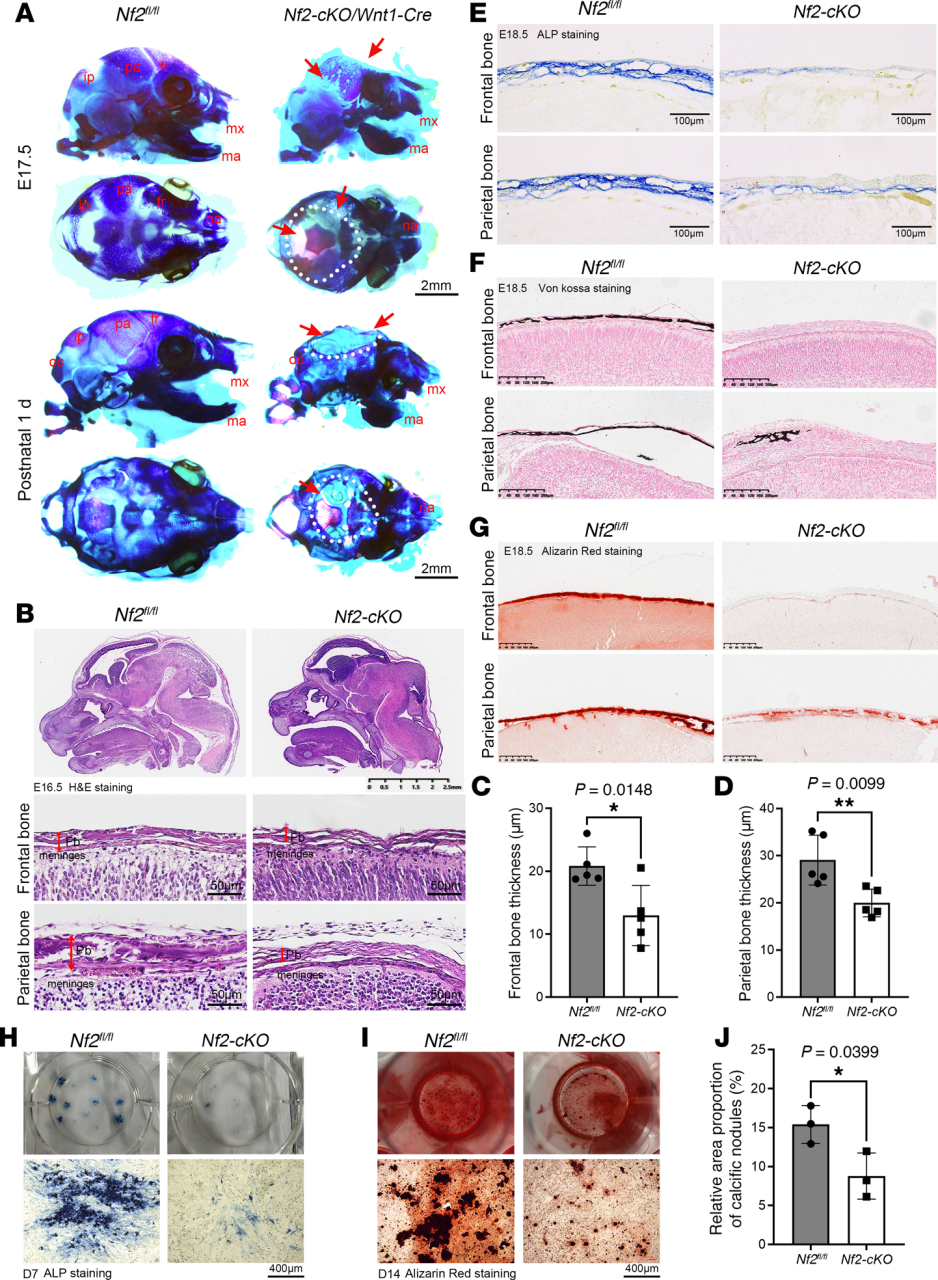
Neural crest–specific *Nf2* deletion impairs craniofacial bone development. (**A**) Whole-mount skeletal staining (Alcian blue and Alizarin red) reveals severe craniofacial defects in *Nf2*-mutant mice at E17.5 and newborn stages, with hypoplastic frontal (fb), parietal (pb), interparietal (ib), and occipital (oc) bones. Nasal bones (na), mandible (ma), and maxilla (mx) are indicated. Scale bars: 2 mm. (**B**–**D**) H&E staining shows reduced frontal and parietal bone thickness in *Nf2* mutants at E16.5, which is quantified in **C** and **D**. (**E**–**G**) Histochemical analysis demonstrated impaired CNC-derived osteogenesis in *Nf2*-mutant mice. (**E**) Diminished ALP activity at E18.5. Scale bars: 100 μm. (**F**) Declined mineralization (von Kossa) at E18.5. (**G**) Decreased calcified matrix (alizarin red S staining) at E18.5. (**H**–**J**) Primary CNC cultures recapitulate in vivo defects. (**H**) ALP staining (D7) shows impaired differentiation. Scale bars: 400 μm. (**I**) Alizarin red S staining (D14) confirmed deficient mineralization. Scale bars: 400 μm. (**J**) Quantification of calcified nodules. Data were expressed as means ± SD, and each dot represents an individual biological replicate. *P* values were calculated by unpaired Student’s *t* test with 2-tailed analysis without adjustments. *P < 0.05, **P < 0.01.

To explore whether *Nf2* plays a role in CNC-derived craniofacial bone development, we specifically knocked out *Nf2* (48) in CNCs using *Wnt1-Cre* (5, 49). *Nf2-cKO Wnt-Cre* mice (*Nf2-cKO*) died after birth. *Nf2*-*cKO* mice displayed abnormalities in the developing forebrain and midbrain regions at E8.5 (Supplemental Figure 1A, arrow; supplemental material available online with this article; https://doi.org/10.1172/jci.insight.191112DS1). At E13.5 and E15.5, *Nf2*-mutant mice exhibited severe skull defects (Supplemental Figure 1A, arrow). Skeleton staining revealed that *Nf2*-deficient mice had smaller skull size (E17.5–P1) (Figure 1A), and the frontal and parietal bones were severely impaired, with massive arrest of skull vault mineralization compared with the controls (Figure 1A, arrow, dotted line). *Wnt1-Cre* is specially labeling neural crest–derived cells, such as neural crest–derived frontal bone and meninges, but not mesoderm-derived parietal bones (5, 6). The interaction of the meninges with the parietal bone has been reported to be critical for normal skull development, such as Tgfbr2 as a potential paracrine factor between parietal and frontal bones (20, 50). Here, in our study, we also found that mesoderm-derived parietal bones were also severely flawed in mice, suggesting a paracrine effect of Nf2 signals in CNC-derived meninges at work in developing mesoderm-derived parietal bone. Our data demonstrated that Nf2 signals are critically required for organizing the development of neural crest–derived frontal bones and mesoderm-derived parietal bones.

Epithelial-mesenchymal interactions are also critical for craniofacial development (51, 52). To explore the potential function of *Nf2* in this process, we deleted *Nf2* in an epithelium-specific model using *K14-Cre* (53). Alizarin red S staining showed that epithelium-specific knockout of *Nf2* did not cause abnormalities in the craniofacial bones, such as frontal bone and parietal bones (Supplemental Figure 1B), indicating that *Nf2* plays an essential role in ecto-mesenchymal cells but not in epithelial cells to direct skull bone development.

At the histological level, H&E staining presented defective skull vaults at E16.5 in *Nf2* mutants (Figure 1B, arrow), including anomalous frontal and parietal bones. The frontal bone and parietal bone (Figure 1, B–D) exhibited remarkably reduced thickness. Alkaline phosphatase (ALP) staining was utilized to evaluate the activities in CNC-derived osteoprogenitors at E12.5. We discovered that *Nf2* mutants displayed greatly declined ALP activities in CNC-derived osteoblasts at E18.5 (Figure 1E). von Kossa staining and alizarin red S staining was used to visualize the mineralization, and we found that *Nf2*-mutant mice displayed severely flawed ossification at frontal bones (Figure 1, F and G) and parietal bones (Figure 1, F and G).

To further verify the osteogenic activity in vitro, we collected the first branchial arch at E10.5, constructed a primary neural crest cell extraction and culture system (54), and then inoculated them to induce their differentiation into osteoblasts. At the day 7 (D7) and D14 differentiation stages, ALP staining (Figure 1H) and alizarin red S staining (Figure 1I) were used, and we found that *Nf2* deletion resulted in diminished ALP activities and mineralization in CNC-derived osteoblasts. The relative area of calcified nodules was significantly declined in *Nf2*-mutant CNC osteoblasts (Figure 1J), thus illustrating a disrupted osteogenesis in *Nf2*-deficient CNCs. Collectively, we substantiated *Nf2*’s essential, cell-intrinsic requirement for normal CNC-derived osteogenesis and skull morphogenesis.

### Nf2 governs CNC-derived osteoprogenitor proliferation, survival, and differentiation.

**Figure 2:**
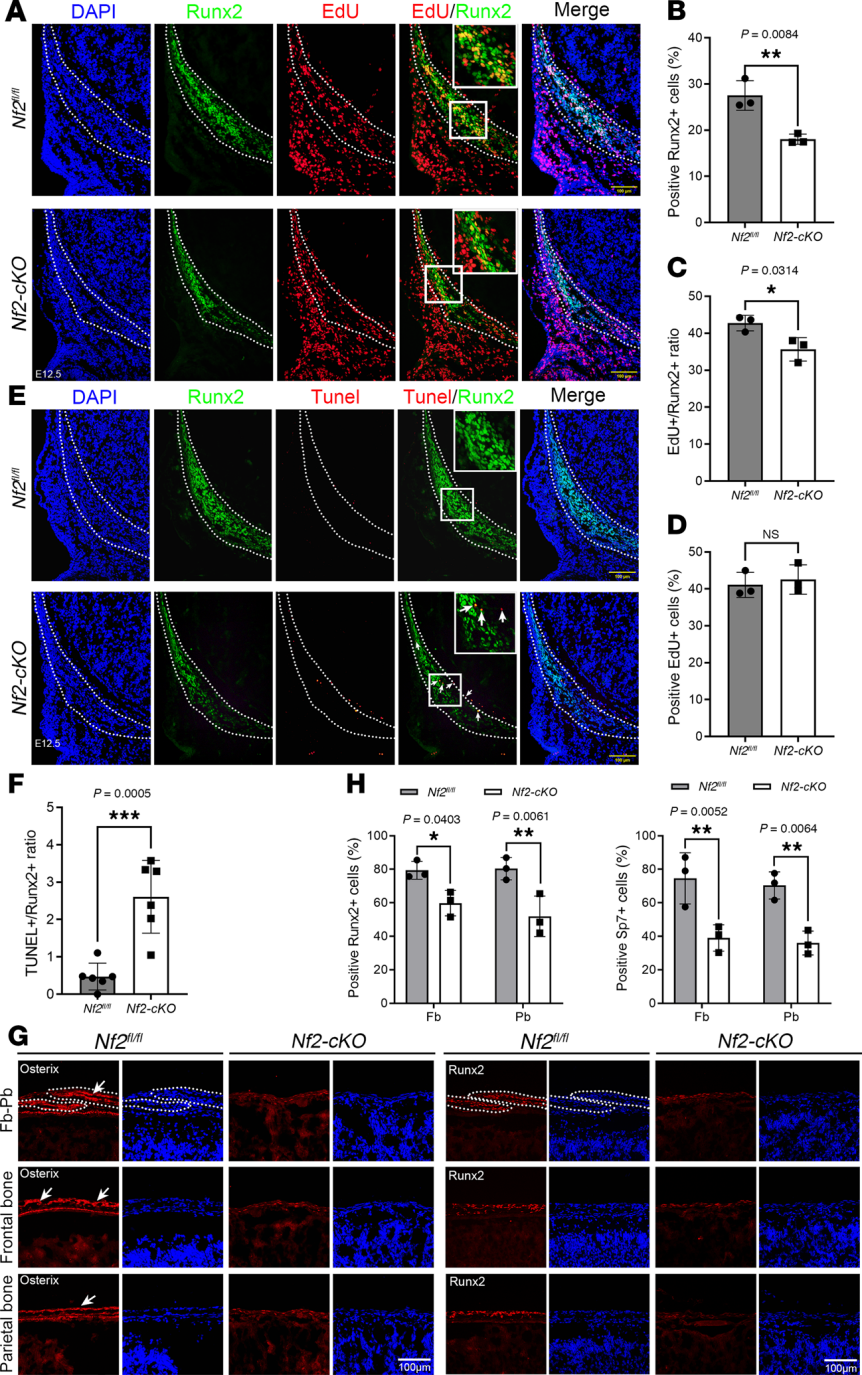
*Nf2* deletion disrupts CNC-derived osteoprogenitor proliferation and differentiation. (**A**) Costaining of Runx2 (osteoprogenitor marker) and EdU (proliferation) in E12.5 coronal sections. Dotted lines demarcate osteogenic zones. *Nf2* mutants show reduced Runx2^+^EdU^+^ cells. Scale bars: 100 μm. (**B**–**D**) Quantification reveals fewer Runx2^+^ cells (**B**), lower Runx2^+^EdU^+^ proliferation (**C**), and unchanged EdU^+^ cells in nonosteogenic regions (**D**) in *Nf2* mutants. (**E** and **F**) Apoptosis analysis shows TUNEL^+^Runx2^+^ cells (arrow) increase in *Nf2* mutants at E12.5, and more apoptotic osteoprogenitors (**F**) were quantified in *Nf2* mutants. Scale bars: 100 μm. (**G**) Immunofluorescence staining of Runx2 and Osterix at E18.5 shows impaired terminal differentiation. Reduced immunosignals of Runx2 and Osterix were found in frontal bone (fb) and parietal bone (pb), and we found near-absent markers at the frontal-parietal junction. Scale bars: 100 μm. (**H**) Quantification of Runx2 and Sp7 expression level in *Nf2* mutants. Data were expressed as means ± SD, and each dot represents an individual biological replicate. *P* values were calculated by unpaired Student’s *t* test with 2-tailed analysis without adjustments (**B**–**D** and **F**) or 2-way ANOVA multiple-comparison test (**H**). *P < 0.05, **P < 0.01, ***P < 0.001.

During cranial development, CNCs migrate before the neural tube is closed, where these migrated cells begin to differentiate into mesenchymal cells and then produce craniofacial structures. Embryonic stage E12.5 is critical for establishing the condensation of CNC-derived mesenchyme cells to form frontal primordia (55). Runx2 is an early marker to distinguish osteogenic progenitor cells and required for CNC-derived osteogenic lineage development (56). The efficacy of *Nf2* knockout was verified in *Nf2*-mutant mice at E12.5 (Supplemental Figure 2B). Then, we discovered that the number of Runx2^+^ osteoprogenitors was significantly diminished in *Nf2*-mutant mice at E12.5 (Figure 2, A and B). EdU incorporation assay was performed to evaluate the cell proliferation. We found that the ratio of EdU^+^/Runx2^+^ was significantly decreased in *Nf2*-deficient mice compared with the control at E12.5 (Figure 2, A and C). No difference in cell proliferation was found in the nonosteoblast area in *Nf2* mutants compared to the control (Figure 2, A and D), indicating that *Nf2* deficiency causes defective CNC-derived osteoprogenitor proliferation. In addition, TUNEL staining was used to evaluate cell apoptosis, and we observed increased apoptotic cells in Runx2^+^ osteoprogenitors in *Nf2*-knockout mice at E12.5 (Figure 2E, arrow), and the ratio of TUNEL^+^/Runx2^+^ was significantly greater in *Nf2* mutants than the control (Figure 2F), implying that unbalanced cell proliferation and apoptosis cause the defective skull development in *Nf2* mutants.

To initiate a normal osteogenesis, Runx2 and Osterix/Sp7, 2 fundamental transcriptional factors, are definitely required for bone ossification (57–59). We performed immunofluorescence staining on E18.5 sagittal skull tissues for Runx2 and Osterix. The results showed that the immunosignals of Runx2^+^ and Osterix^+^ were significantly diminished in frontal bones in *Nf2*-mutant mice compared with the controls (Figure 2, G, arrow, and H), while the immunosignals of Runx2^+^ and Osterix^+^ were hardly detectable in parietal bones of *Nf2-cKO* mice (Figure 2, G, arrow, and H), indicating that *Nf2*’s dual regulatory roles as a cell-autonomous requirement for CNC-derived osteogenesis and a paracrine modulator of mesodermal bone formation. Collectively, our findings demonstrated *Nf2* as a nodal point in a transcriptional network governing craniofacial morphogenesis, with particular dominance in the neural crest lineage.

### Nf2 mediates the synthesis and trafficking of type I collagen in CNC-derived osteoblasts.

**Figure 3:**
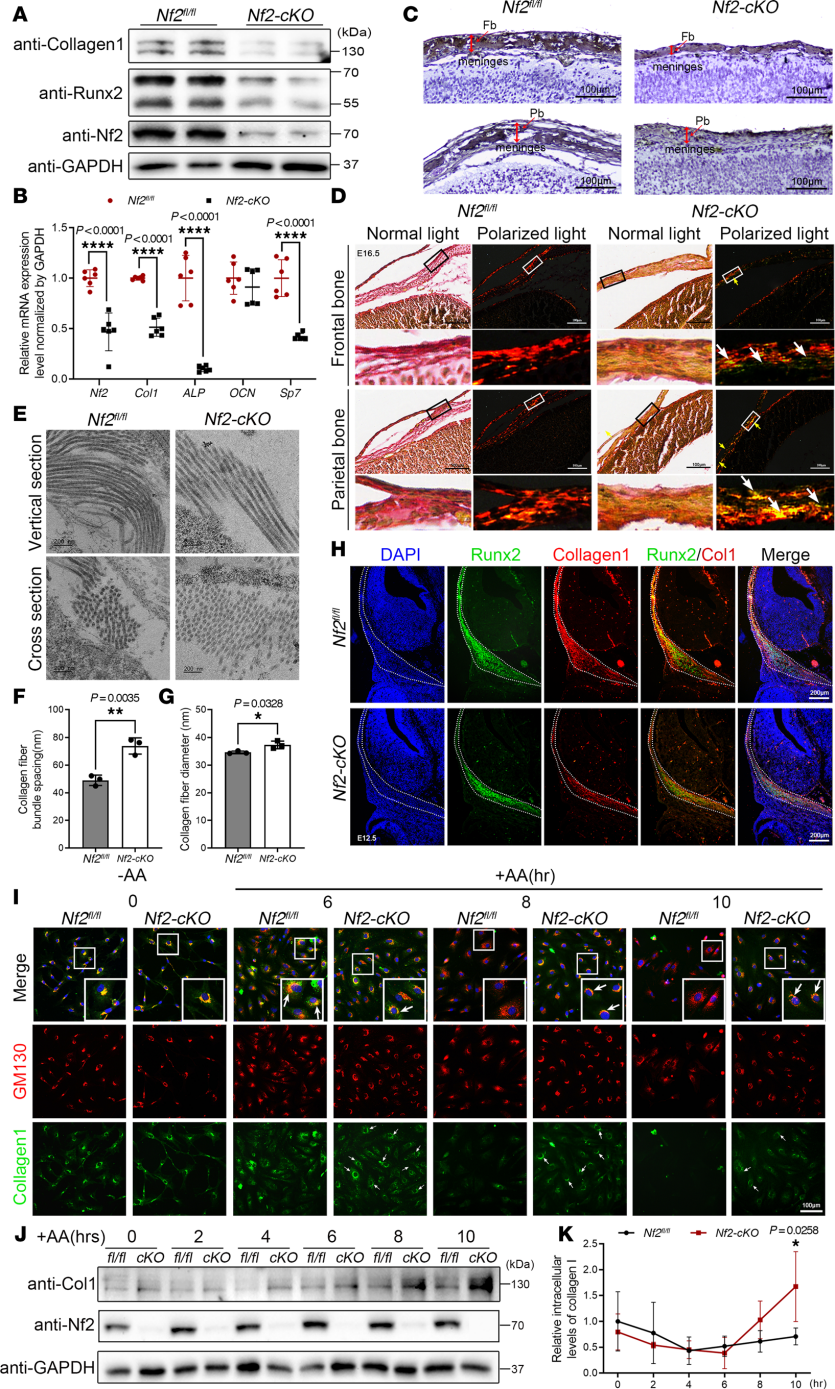
Nf2 regulates type I collagen biosynthesis and secretion in CNC-derived osteoblasts. (**A** and **B**) Molecular profiling reveals osteogenic defects: Western blot analysis (**A**) shows reduced osteogenic markers in *Nf2*-mutant mice at D7, and quantitative reverse transcription PCR (qRT-PCR) (**B**) confirms severe downregulation of *ALP*, *RUNX2*, *Sp7*, and *Col1*) in *Nf2* deficient CNC-derived osteoblasts. (**C**–**H**) Collagen matrix abnormalities. (**C**) IHC shows less type I collagen in E16.5 *Nf2*-mutant coronal sections. Scale bars: 100 μm. (**D**) Picrosirius red staining reveals disorganized collagen fibers (arrow) in *Nf2*-mutant mice at E16.5. Scale bars: 100 μm. (**E**) TEM demonstrates defective fibril ultrastructure at E18.5, and quantification shows wider collagen fiber diameter (**G**). Scale bars: 200 nm. (**F**) Increased interfibrillar spacing in *Nf2* mutants. (**H**) Costaining confirms reduced collagen in Runx2^+^ osteoprogenitors at E12.5. Scale bars: 200 μm. (**I** and **J**) Secretory pathway impairment. (**I**) AA-stimulated (50 μg/mL) *Nf2*-mutant CNC-derived osteoblasts retain pro–collagen I in Golgi (GM130^+^, arrow). Scale bars: 100 μm. (**J** and **K**) Western blot quantified more intracellular collagen retention in *Nf2* mutants after AA stimulation for the indicated time course. Data were expressed as means ± SD, and each dot represents an individual biological replicate. *P* values were calculated by unpaired Student’s *t* test with 2-tailed analysis without adjustments (**F** and **G**) and 2-way ANOVA multiple-comparison test (**B** and **K**). *P < 0.05, **P < 0.01, ****P < 0.0001.

In CNC-derived osteoblasts, the activities of Runx2 and type I collagen (*Col1*) were substantially attenuated in *Nf2* deficient mice (Figure 3A). The relative gene expressions of osteoblast-specific marker genes (*ALP*, *RUNX2*, *Sp7*, *Col1*) were significantly declined on D7 (Figure 3B). The immunohistochemical staining showed that the synthesis of type I collagen was significantly reduced in *Nf2*-mutant skull tissues (Figure 3C and Supplemental Figure 2E). Additionally, the immunosignals of type I collagen were noticeably diminished in Runx2^+^ osteoprogenitors in *Nf2* mutants at E12.5 (Figure 3H). Type I collagen provides mechanical support and participates in bone mineralization (60, 61). Impaired synthesis of type I collagen causes various bone diseases, including osteogenesis imperfecta (62). To further visualize the integrity of collagen fibers, Picrosirius red staining and transmission electron microscopy (TEM) were used, and we discovered that *Nf2-cKO* mice had discontinuous type I collagen fibrils (Figure 3D, arrow, and Supplemental Figure 2F); the collagen fibers in the *Nf2* mutants were looser than in the control (Figure 3E); and the interfibrillar spacing was increased (Figure 3F) and the fibril diameter was wider in *Nf2* mutants (Figure 3G), revealing Nf2’s unprecedented role in coordinating both collagen production and extracellular matrix maturation, whose failure phenocopies osteogenesis imperfecta–like pathogenesis through disrupted fibrillogenesis and mineralization competence.

In rough endoplasmic reticulum (rER), the pro-collagen molecules undergo multiple posttranslational modifications; e.g., the hydroxylation of amino acid and lysine residues is catalyzed by prolyl 3-hydroxylase (P3H), prolyl 4-hydroxylase (P4H), and lysyl hydroxylase (LH). All 3 enzymes require ascorbic acid (AA) as a cofactor (63, 64). Western blot analysis revealed no significant differences in ER stress markers (GRP78/BiP and ATF4) (65) between *Nf2*-mutant and control osteoblasts (Supplemental Figure 2C), indicating that ER stress is not notably activated under these conditions. In control CNC-derived osteoblasts, Nf2 partially colocalized with the Golgi marker Golgi matrix protein 130 (GM130) by immunofluorescence (Supplemental Figure 2D), suggesting its potential involvement in secretory protein trafficking. To study the secretion and trafficking of collagen in CNC-derived osteoblasts, we stimulated primary CNC-derived osteoblasts with AA and detected intracellular type I pro-collagen levels (Figure 3I). Following the AA stimulation for 6 hours, most CNC-derived osteoblasts showed successful transport of type I pro-collagen to the Golgi apparatus in controls, which was colabeled using GM130 antibody (Figure 3I, arrow, and Supplemental Figure 2G). After the AA stimulation for 8 hours, many CNC-derived osteoblasts actively secreted collagen from the Golgi apparatus externally in controls, as less GM130 and pro-collagen colabeling was found, indicating a dynamic trafficking for synthesized type I collagen. On the contrary, most *Nf2*-mutant CNC-derived osteoblasts showed colabeling of GM130 and pro-collagen after AA stimulation, suggesting that *Nf2*-mutant osteoblasts showed insufficient collagen transport from the Golgi apparatus (Figure 3I, arrow, and Supplemental Figure 2G).

To validate the intracellular type I collagen synthesis, we analyzed intracellular collagen protein and found that the intracellular type I collagen in CNC-derived osteoblasts was higher in the cytoplasm than that in controls after AA treatment for 6 hours, and this trend was significantly higher after AA treatment for 10 hours in *Nf2*-mutant CNC-derived osteoblasts (Figure 3, J and K), suggesting that *Nf2*-mutant osteoblasts showed longer retained synthesized type I collagen. Intracellular collagen of control CNC-derived osteoblasts was extracellularly secreted at regular frequency (Figure 3, J and K), which was momentous to maintain a usual bone matrix activity for osteogenesis. Our data establish Nf2 as a regulator of collagen trafficking fidelity, whose dysfunction leads to pathological ECM accumulation mimicking osteogenesis imperfecta–type secretory defects.

### Nf2 plays a crucial role in modulating FGFR1/Akt signaling in CNC-derived osteoblasts.

**Figure 4:**
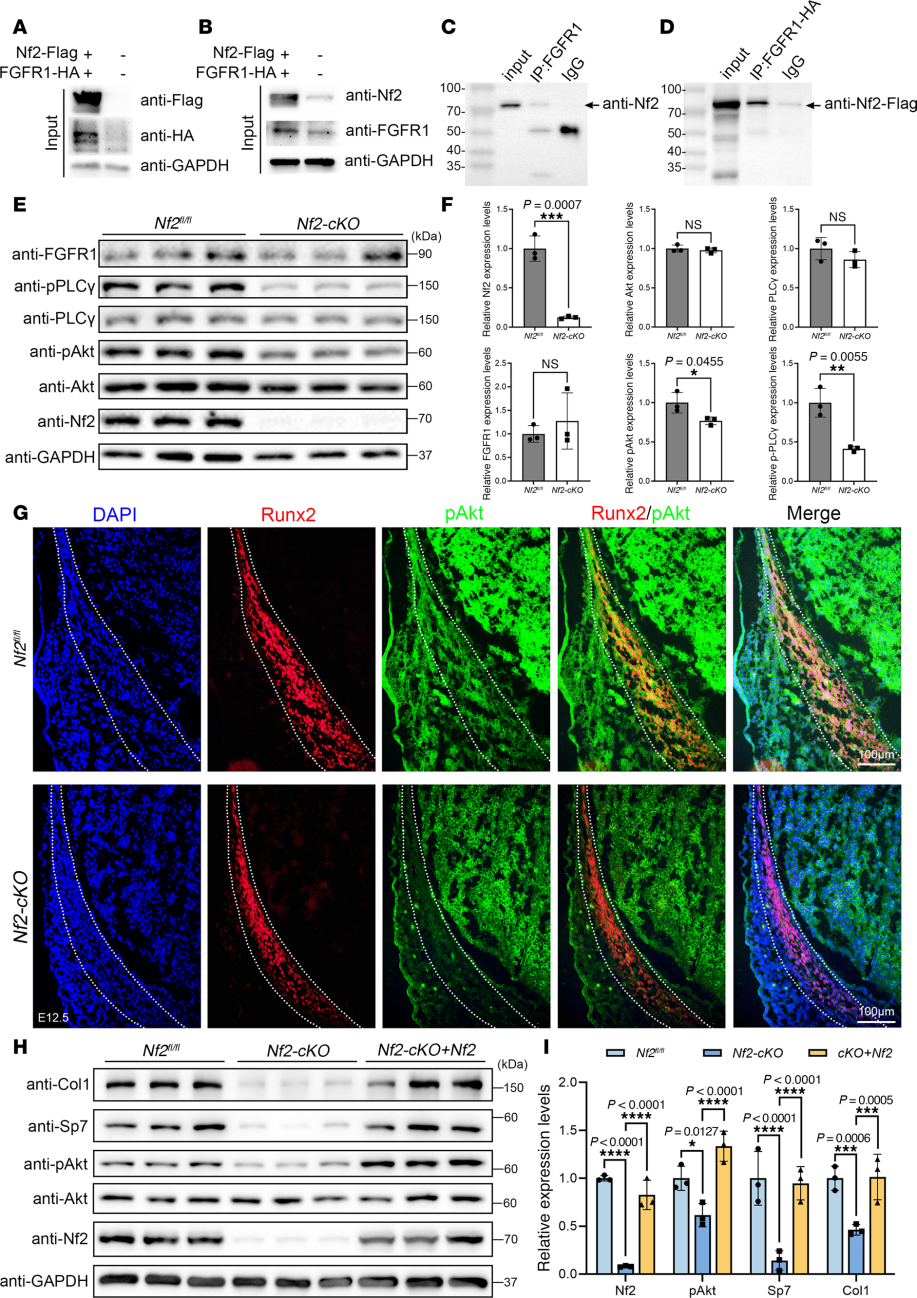
Nf2 scaffolds FGFR1/Akt signaling to regulate CNC-derived osteoblast function. (**A**–**D**) Nf2-FGFR1 interaction mapping. (**A** and **B**) Co-immunoprecipitation (co-IP) in HEK293T cells confirms specific Nf2-FGFR1 binding (HA/FLAG tags) (**D**). (**C**) Endogenous co-IP validates Nf2-FGFR1 complex in primary CNC-derived osteoblasts. (**E** and **F**) Downstream signaling defects in *Nf2*-mutant CNC-derived osteoblasts. Western blot shows unchanged FGFR1 but reduced p-Akt and p-PLCγ in *Nf2*-mutant primary CNC-derived osteoblasts (**E**). Quantification confirms selective pathway impairment (normalized to GAPDH) (**F**). (**G**) Costaining of Runx2 and p-Akt in E12.5 coronal sections shows reduced p-Akt^+^/Runx2^+^ osteoprogenitors in *Nf2* mutants (dotted lines demarcate osteogenic zone). Scale bars: 100 μm. (**H** and **I**) Western blot shows the levels of p-Akt, Osterix/Sp7, and collagen type I were sufficiently rescued after the lentivirus-mediated Nf2 overexpression in *Nf2*-mutant osteoblasts (**H**). (**I**) Quantification of expression level of p-Akt, Sp7, and collagen type I (normalized to GAPDH). Data were expressed as means ± SD, and each dot represents an individual biological replicate. *P* values were calculated by unpaired Student’s *t* test with 2-tailed analysis without adjustments (**F**) and 2-way ANOVA multiple-comparison test (**I**). *P < 0.05, **P < 0.01, ***P < 0.001, ****P < 0.0001.

Nf2 has been shown to interact with diverse membrane receptors, such as VEGFR (66) and EGFR (67). PDGFRα/β (68, 69) and FGFR1-4 (70–72) have been documented to be indispensable for CNC development. To explore the potential mechanism of Nf2 in CNC-derived osteoblasts, we conducted co-immunoprecipitation (co-IP) experiments using numerous transient expression plasmids for PDGFRα/β and FGFR1-4 to evaluate the potential interaction with Nf2. We discovered that Nf2 exhibited no interaction with PDGFRα/β and FGFR2-4 (Supplemental Figure 3D). Interestingly, upon the successful transfection of Nf2-Flag and FGFR1-HA plasmids into HEK293T cells (Figure 4, A and B), we uncovered a specific interaction between FGFR1-HA and Nf2-Flag (Figure 4D). Subsequently, we collected primary CNC-derived osteoblasts, performed endogenous co-IP, and validated that FGFR1 and Nf2 form a functional complex (Figure 4C).

FGFR signaling is remarkably influential for CNC development, and FGFR binds to ligands and undergoes receptor dimerization, leading to the activation of central downstream pathways, for instance PI3K/AKT and PLCγ (73). We found that the activity of FGFR1 remained unchanged in *Nf2*-deficient CNC-derived osteoblasts (Figure 4, E and F). This was further supported by immunofluorescence staining (Supplemental Figure 2A). Its downstream signaling was selectively impaired, as the phosphorylated PLCγ (p-PLCγ) was significantly diminished in *Nf2*-mutant CNC-derived osteoblasts (Figure 4, E and F). Akt acts as the main output of PI3K signaling (74), and the activity of p-Akt was significantly decreased in *Nf2*-mutant CNC-derived osteoblasts (Figure 4, E and F), suggesting Nf2 specifically regulates signal transduction rather than receptor activation. Spatial analysis revealed that this defect originated in CNC-derived osteoprogenitors, and the immunosignals of p-Akt were decreased in CNC-derived Runx2^+^ osteoprogenitors in *Nf2*-mutant mice at E12.5 (Figure 4G). Additionally, *Nf2* overexpression mediated by lentivirus in *Nf2*-mutant osteoblasts rescued p-Akt, collagen type I, and Osterix/Sp7 levels (Figure 4, H and I), suggesting Nf2 works as an upstream modulator of Akt activity during CNC-derived osteogenesis. These results establish Nf2 as a molecular switch that licenses FGFR1 signaling specifically through the PI3K/Akt axis during CNC osteogenesis.

### Nf2 couples with Akt to regulate osteogenic differentiation and collagen biosynthesis in CNC-derived osteoblasts.

**Figure 5:**
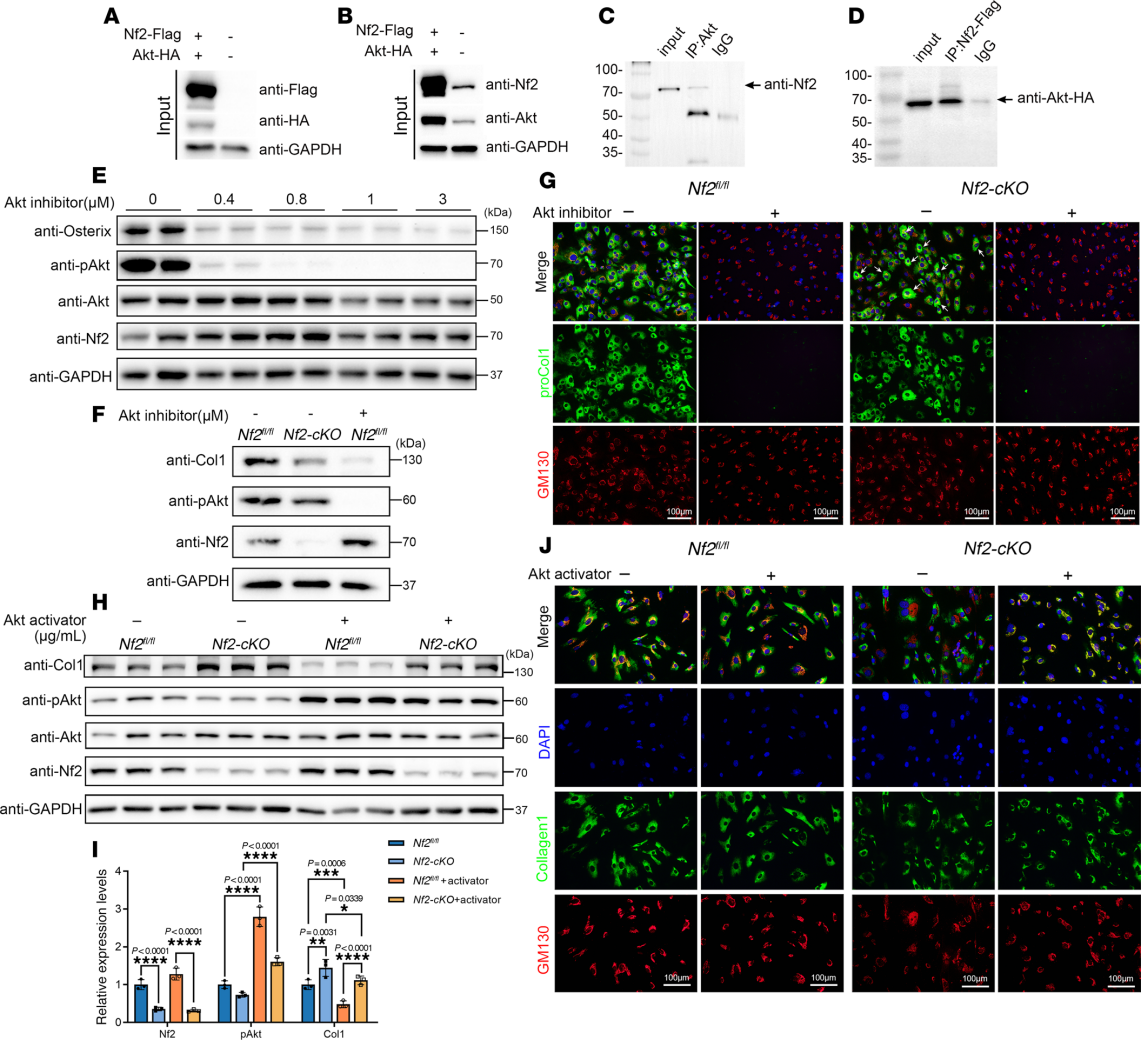
Nf2-Akt interaction governs osteogenic function and collagen production. (**A**–**D**) Nf2-Akt complex formation. (**A** and **B**) Reciprocal co-IP in HEK293T cells demonstrates direct Nf2-Flag/Akt-HA binding (anti-FLAG/HA) (**D**). (**C**) Endogenous Nf2-Akt interaction is confirmed in primary CNC-derived osteoblasts. (**E**–**G**) Akt dependence of osteogenic function. (**E**) Dose-dependent Akt inhibition (MK2206, 24 h) reduces Osterix activity in *Nf2*-mutant primary CNC-derived osteoblasts. (**F**) Western blot shows less collagen I after Akt inhibition. (**G**) Immunofluorescence reveals near-complete loss of pro–collagen I in Golgi (GM130^+^) upon Akt inhibition treatment, as emphasized by arrows. Scale bars: 100 μm. (**H** and **I**) Western blot analysis for measuring the amount of intracellular type I collagen after Akt activator stimulation for the indicated time course (SC79, 12 hours) (**H**). Quantification of intracellular type I collagen and p-Akt (normalized to GAPDH) (**I**). (**J**) Immunofluorescence staining reveals the cellular localization of pro–collagen I in Golgi (GM130^+^) treated with dose-dependent Akt activator for the indicated time. Scale bars: 100 μm. Data were expressed as means ± SD, and each dot represents an individual biological replicate. *P* values were calculated by 2-way ANOVA multiple-comparison test (**I**). *P < 0.05, **P < 0.01, ***P < 0.001, ****P < 0.0001.

Nf2 has been reported to interact with Akt in cancers (75). To further explore the relationship between Akt and Nf2 in osteoblasts, we generated exogenous plasmids Nf2-Flag and Akt-HA (Figure 5, A and B). A co-IP experiment was performed and found that Nf2 and Akt displayed exogenous interactions (Figure 5D). Furthermore, in primary CNC-derived osteoblasts, we substantiated the endogenous interaction between Nf2 and Akt (Figure 5C), while revealing no direct FGFR1-Akt binding (Supplemental Figure 3, A and B). co-IP assays showed that Nf2 interacts with both FGFR1 and Akt individually, but no interaction was detected between FGFR1 and Akt in the presence of Nf2, excluding the formation of a stable ternary complex (Supplemental Figure 3C). Subsequently, we used Akt inhibitors (MK2206) (76, 77) to treat the primary CNC-derived osteoblasts for 24 hours. We found that pharmacological inhibition of Akt markedly reduced p-Akt level (Figure 5E and Supplemental Figure 2H) and Osterix expression in CNC-derived osteoblasts (Figure 5E and Supplemental Figure 2H), exhibiting Akt’s critical role in maintaining the osteogenic program in *Nf2* mutants.

To further authenticate the effect of Akt activity on type I collagen synthesis, Akt inhibitor (MK2206) at the concentration of 0.5 μM was applied to treat primary CNC-derived osteoblasts for 24 hours. Our results showed that the activity of type I collagen was substantially declined when the activity of p-Akt was effectively inhibited using Akt inhibitor (Figure 5F). Furthermore, the immunofluorescence staining substantiated that when Akt inhibitor was applied, the synthesis of pro–collagen type I nearly disappeared in primary CNC-derived osteoblasts compared with controls not treated with inhibitor (Figure 5G). To clarify whether Akt activation can rescue collagen secretion defects in *Nf2*-deficient osteoblasts, we treated primary CNC-derived osteoblasts with Akt activator (SC79) at 8 μg/mL for 12 hours. Western blot analysis showed increased level of intracellular type I collagen (Figure 5, H and I), and immunofluorescence revealed enhanced type I pro-collagen localization to the Golgi compartments (Figure 5J), suggesting that Akt activation is sufficient to enhance collagen secretion. However, Akt antagonist or agonist administration in vivo failed to rescue the *Nf2*-mutant phenotype, probably due to the blood-brain barrier. Collectively, our data underscored that Nf2-mediated FGFR1/Akt signaling is indispensable for the biosynthesis of type I collagen in CNC-derived osteoblasts.

### Nf2 requires Akt-mediated phosphorylation for osteogenic function but not FGFR1 binding.

Nf2 protein adopts a tripartite architecture comprising an N-terminal (NTD) FERM (F for 4.1 protein, E for ezrin, R for radixin, and M for moesin) domain, a central helix (α-helical) domain, and a C-terminal domain (CTD), with intramolecular FERM-CTD associations dictating its closed/inactive versus open/active conformations (78). Phosphorylation at 4 conserved residues (Ser10, Thr230, Ser315, Ser518) serves as a molecular switch controlling this conformational equilibrium, with distinct kinases selectively modifying these sites, e.g., PKA (Ser10, Ser518) (34, 35), AKT (Thr230, Ser315) (79), and PAK (Ser518) (80, 81), revealing Nf2 as a phospho-integration hub that translates diverse extracellular cues into coordinated functional outputs.

**Figure 6:**
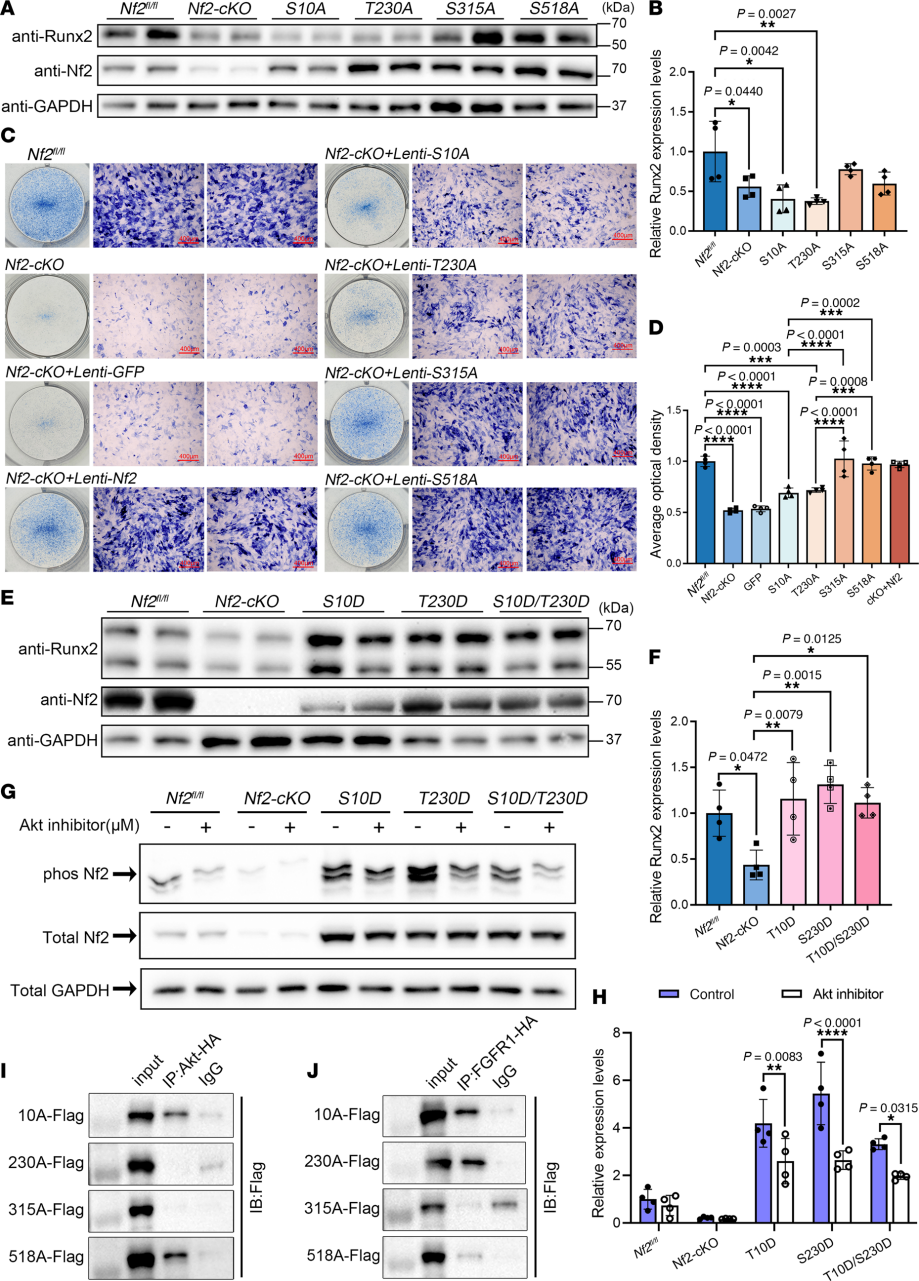
Phospho-regulation of Nf2 at Ser10/Thr230 controls osteogenic capacity through Akt coupling. (**A**–**D**) Phospho-ablative Nf2 mutants impair osteogenesis. (**A** and **B**) Constructing Nf2 single-site mutation plasmids (S10A, T230A, S315A, S518A). Nf2 (T230A) and Nf2(S10A) reduce Runx2 activity in *Nf2*-mutant CNC-derived osteoblasts compared with the *Nf2*-mutant controls, while S315A/S518A show minimal effects. (**C** and **D**) ALP activity is selectively diminished in Nf2-T230A and S10A mutants. Scale bars: 400 μm. (**E** and **F**) Phospho-mimetic rescue: Constructing Nf2 single-site continuous phosphorylation plasmids (S10D, T230D, S10D/T230D). Nf2 (T230D) and Nf2(S10D) sufficiently restore Runx2 in *Nf2*-mutant primary osteoblasts compared with the control (**F**). (**G** and **H**) Akt dependence assay. (**G**) Akt inhibitor–treated primary osteoblasts (24 hours) showed reduced Nf2 phosphorylation following lentivirus-mediated Nf2 (T230D) and Nf2(S10D) transfection, and untreated cells were used as controls, confirming Akt mediates Ser10/Thr230 modification. (**H**) Quantification of relative levels of Nf2 phosphorylation (normalized to GAPDH). (**I** and **J**) Site-specific binding assay. (**I**) Co-IP assays were performed in HEK293T cells transfected with site-mutant plasmids. T230A disrupts Nf2-Akt binding, while S10A maintains interaction. (**J**) Co-IP assays were performed in HEK293T cells transfected with *S10A-Flag*, *T230A-Flag*, *S315A-Flag*, *S518A-Flag*, and *FGFR1-HA* plasmids. S315A/S518A abolishes Nf2-FGFR1 coupling, with T230A/S10A unaffected. Data were expressed as means ± SD, and each dot represents an individual biological replicate. *P* values were calculated by 1-way ANOVA multiple-comparison test (**B**, **D**, and **F**) or grouped 2-way ANOVA multiple-comparison test (**H**). *P < 0.05, **P < 0.01, ***P < 0.001, ****P < 0.0001.

To advance the understanding on the physiological function between Akt and Nf2 in CNC-derived osteoblasts, single-site mutations on Nf2-Ser10, Nf2-Thr230, Nf2-Ser315, and Nf2-Ser518 were constructed, respectively, namely S10A, T230A, S315A, and S518A. Additionally, we constructed constitutive phosphorylation plasmids at Nf2-S10A and Nf2-T230A, namely S10D and T230D. In addition, GFP acts as a negative control, and lentivirus-mediated Nf2 site mutation–overexpressing plasmids were used. Functional dissection of Nf2 phosphorylation sites through lentivirus-mediated mutagenesis in *Nf2*-mutant primary CNC-derived osteoblasts revealed that Ser10 and Thr230 are critical for osteogenic activity: (a) Nonphosphorylatable mutants (S10A/T230A) failed to rescue Runx2 expressions (Figure 6, A and B) or ALP activity in *Nf2*-mutant CNC-derived osteoblasts compared with CNC-derived mutants and controls (Figure 6, C and D), while S315A/S518A showed near-normal function; (b) phospho-mimetic mutants (S10D/T230D) sufficiently restored Runx2 levels in *Nf2*-mutant osteoblasts (Figure 6, E and F), demonstrating that Ser10/Thr230 phosphorylations are essential for Nf2’s osteogenic function.

Akt has been reported to directly bind and phosphorylate Thr230 and Ser315 residues within Nf2, thus eliminating the NTD/CTD interaction of Nf2 (79). The phosphorylation of Akt can enhance the interaction between the “open” conformation of Nf2 and phosphatidylinositol (75). To delineate Akt’s role in Nf2 phosphorylation, we transfected plasmids of S10D, T230D, or double S10D/230D into *Nf2*-mutant primary CNC-derived osteoblasts and used Akt inhibitor to treat the transfected osteoblasts for 24 hours. The phospho-tag gel was used to evaluate the state of Nf2 phosphorylation, and we found that Akt inhibition noticeably reduced Nf2 phosphorylation under the conditions with constitutively expressed S10D and T230D sites in Nf2 (Figure 6, G and H), suggesting that Akt can exert sufficient kinase action to activate Nf2 phosphorylation at Ser10 and Thr230 sites in osteoblasts.

**Figure 7:**
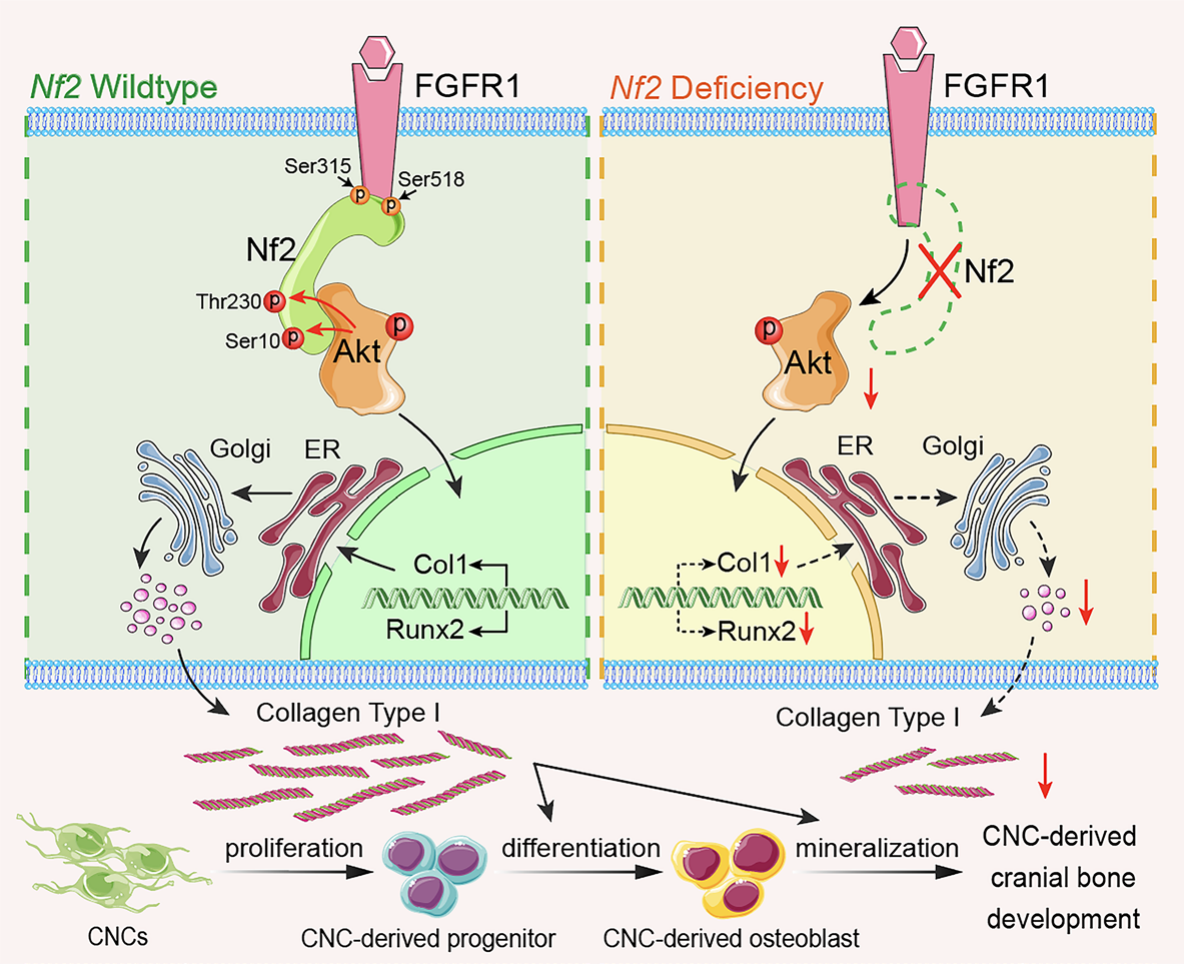
Molecular mechanism of Nf2-mediated FGFR1/AKT signaling in CNC-derived osteoblasts. Nf2’s domain-specific phosphorylation creates distinct functional modules: C-terminal S315/S518 mediate FGFR1 binding, while N-terminal T230 phosphorylation by AKT serves as the essential docking interface. This spatial segregation enables Nf2 to simultaneously coordinate FGFR1 signaling and Akt-dependent collagen regulation, with *Nf2* deficiency causing highly reduced AKT activation and impaired collagen trafficking, while phospho-mimetic T230D rescues mineralization defects, revealing a targetable phospho-switch for craniofacial development. In short, Nf2 plays a significant role in optimizing the coupling with FGFR1 and AKT to mediate the synthesis and trafficking of type I collagen for a normal CNC-derived osteogenesis and cranial bone development.

To explore the relevance among Nf2 phosphorylation, FGFR1, and Akt, we performed co-IP experiments and revealed that mutation on Ser10 of Nf2 does not cause the disassociation with Akt, but Thr230 does, suggesting that the conformation of Nf2 protein on phosphorylation sites may act differently at physiological state (Figure 6I). Site mutation on Ser315 in Nf2 also disconnected the interaction with Akt (Figure 6I), suggesting site Ser315 of Nf2 may function independently of or along with site Thr230 of Nf2 to interact with Akt. Additionally, mutations of Nf2-Ser315 or Nf2-Ser518 caused its uncoupling with FGFR1, but this was not true for mutations on Ser10 and Thr230 of Nf2 (Figure 6J), implying that sites of Ser315 and Ser518 in CTD of Nf2 are responsible for interacting with FGFR1 but not the sites of Ser10 and Thr230 in NTD of Nf2. Strikingly, phospho-mimetic mutants (S10D/T230D) maintained Akt (Supplemental Figure 3E) or FGFR1 (Supplemental Figure 3F) binding in the physiological state in CNC-derived osteoblasts. Our data underscore that the endogenous biological state of Nf2 phosphorylation is critically required to optimize its interactions with FGFR1 and Akt to facilitate intracellular modulation on collagen synthesis and trafficking (Figure 7) and thus to coordinate an established and standard CNC-derived osteogenic competence and cranial bone development.

## Discussion

### Nf2 plays a central role in orchestrating CNC-derived craniofacial bone development.

CNCs are pivotal for craniofacial development, with their dysregulation causing severe birth defects (82–84). While craniofacial bones arise from dual origins (CNC-derived frontal bones and mesoderm-derived parietal bones), the molecular regulation of CNC osteogenesis remains poorly understood. *Nf2* gene, expressed from E5.5 in mice (39, 40), is essential for embryogenesis (homozygous lethality) (85) and suppresses tumorigenesis in heterozygotes (86–95). Although known to regulate diverse tissues, such as in the skin (96), embryonic axis (97), neural tube (43, 98), tongue (42), endometrial gland (99), mammalian epidermis (96), eye (100), muscle (101), dorsal root ganglion (41), and cardiomyocytes (102), its hematopoietic lineage-specific role in promoting trabecular bone (44, 45) contrasts with our discovery of its critical requirement in CNC-derived osteogenesis. Neural crest–specific *Nf2* deletion causes severe skull vault defects, revealing its functions in CNC-derived osteoprogenitor maintenance, collagen matrix assembly, and FGFR1/Akt signaling, highlighting *Nf2* as a therapeutical target for CNC-related craniofacial anomalies.

### The Nf2/FGFR1/Akt axis is critical for CNC-derived osteogenesis via collagen synthesis and trafficking.

Collagen I biosynthesis and secretion constitute a fundamental determinant of bone matrix quality, providing both structural integrity and mineralization templates (60, 61). Despite their central role in skeletal development and diseases (62, 103), the regulatory mechanisms governing collagen dynamics in cranial osteogenesis remain undefined. We demonstrate that Nf2 controls the collagen secretory pathway through 2 coordinated mechanisms: (a) posttranslational modification via AA-dependent hydroxylation enzymes (P3H/P4H/LH) (63, 64) and (b) vesicular trafficking fidelity, as evidenced by profound collagen retention in *Nf2*-mutant osteoblasts following AA stimulation, suggesting an unrecognized role of Nf2 in regulating collagen biosynthesis.

Nf2 plays a significant role in connecting with assorted signaling molecules (33, 46). What is more, numerous signaling pathways were found to be substantially required during the contribution of CNCs to skull development, including bone morphogenetic proteins (104–106), PDGFs (107–109), WNT/β-catenin (11, 110, 111), Hedgehog signaling (112–115), and FGF signaling (70, 116, 117). In our study, we demonstrated that Nf2 exhibited no interaction with PDGFRα/β and FGFR2-4 (Supplemental Figure 3D) but showed a specific interaction with FGFR1 in CNC-derived osteoblasts. FGF signaling is critically required for established functions in CNC-derived osteogenic differentiation (19, 118), cranial bone growth (19, 20), and the repair of skull defects (21). *FGFR1-3* deficiency was frequently found as a cause in several craniofacial dysostosis syndromes (22–26). In particular, *FGFR1* loss in CNCs resulted in ectopic osteogenesis at the anterior frontal interface (27) and defective craniofacial morphology (28). Strikingly, while *Nf2* deletion preserves FGFR1 expression, it severely compromises downstream PI3K/AKT (116, 119, 120) and PLCγ (121, 122) signaling, revealing Nf2’s essential role as signal transducer rather than receptor stabilizer. Structural and functional analysis further demonstrates that Nf2 bridges FGFR1 and Akt, forming a tripartite complex that orchestrates collagen I biosynthesis and secretory trafficking. These findings establish the Nf2/FGFR1/Akt signaling axis as a lineage-specific regulator of CNC osteogenesis and a potential therapeutic target for collagen-related craniofacial disorders.

### Nf2 phosphorylation is required for optimized interaction with FGFR1 and Akt.

The Nf2/Merlin tripartite architecture (FERM–α-helical–CTD) undergoes phosphorylation-dependent conformational switching between closed (growth-suppressive) and open (growth-permissive) states (37, 38, 123). While 4 conserved phospho-sites integrate signals from diverse kinases — PKA (34–36) at Ser10 and Ser518, Akt (37) at Thr230 and Ser315, and PAK (38) at Ser518 — we identify Thr230 and Ser10 as critical regulatory nodes for CNC-derived osteogenesis. Functional studies demonstrate that phospho-ablative mutations (S10A/T230A) severely impair osteoblast differentiation, while phospho-mimetic variants (S10D/T230D) rescue mineralization defects, establishing a phosphorylation barcode that specifically licenses Nf2’s pro-osteogenic functions through Akt-dependent conformational activation in CNC-derived osteoblasts.

Akt has been described to directly bind and phosphorylate Thr230 and Ser315 residues of Nf2, thus eliminating the NTD/CTD interaction of Nf2 (79). The p-Akt enhanced the interaction between the open conformation of Nf2 and phosphatidylinositol (75). In our study, we revealed that Akt phosphorylates Ser10 and Thr230 in Nf2 protein in osteoblasts. However, mutation on Ser10 of Nf2 does not cause the disassociation with Akt, but Thr230 does, suggesting that phosphorylation on Ser10 and Thr230 may perform divergent physiological functions. Additionally, mutations of Ser315 and Ser518 of Nf2 caused its uncoupling with FGFR1 but not that on Ser10 and Thr230 of Nf2, implying that Ser315 and Ser518 of Nf2 plays an important role in interacting with FGFR1 but not Ser10 and Thr230 of Nf2. Meanwhile, phospho-mimetic Nf2 (S10D/T230D) stabilizes the tripartite complex, demonstrating that site-specific phosphorylation orchestrates signalosome assembly to regulate collagen trafficking, underscoring that the endogenous biological state of Nf2 is critical to optimize connecting with FGFR1 and Akt to facilitate intracellular modulation on collagen synthesis and trafficking.

Taken together, our findings unveil an Nf2/FGFR1/Akt signaling axis that orchestrates type I collagen synthesis and trafficking to ensure proper CNC-derived osteogenesis and cranial morphogenesis, thereby establishing this axis as a promising therapeutic target for innovative interventions in craniofacial anomalies.

## Methods

### Sex as a biological variable.

Our study examined male and female animals, and similar findings are reported for both sexes.

### Generation of Nf2-cKO mice.

The animals were maintained in the animal facilities in the Experimental Animal Center at the Hangzhou Normal University.

The *Nf2*-floxed allele (*fl/fl*) was provided by Inserm (48). *Wnt1-Cre* was provided by Zuoyun Wang (Fudan University, Shanghai, China). *Nf2^fl/fl^* mice and mice with tissue-specific promoter-driven *Cre* (*Wnt1-Cre*) were crossed to generate heterozygous mice, which were intercrossed with *Nf2^fl/fl^* to obtain homozygous *cKO* mice. *Cre*-negative littermates served as controls.

The following primers were used for genotyping (42, 46): 5′-CTTCCCAGACAAGCAGGGTTC-3′ and 5′-GAAGGCAGCTTCCTTAAGTC-3′ for *Nf2^fl/fl^* (~442 bp) and WT (~305 bp) fragments; 5′-TCCAATTTACTGACCGTACACC-3′ and 5′-CGTTTTCTTTTCGGATCC-3′ for the general Cre gene product (~372 bp). *Wnt1-Cre* was determined using *Wnt1-wt-F*: 5′-AGGCATGTGTTTCCCTTCTG-3′; *Wnt1-co-R*: 5′-GAGTCCAGGTCCTCTGGTTG-3′ and *Wnt1-ko-F*: 5′-CTCTTCCGGAGGAAAATGTC-3′, and a fragment of ~198 bp for WT and ~255 bp for homozygote can be visualized.

### Skeletal staining.

Alizarin red–Alcian blue staining was used to determine the skeletal structure in embryos. Skeletons in gestation at different developmental stages were double-stained for cartilage and bone using Alcian blue and alizarin red solution (46). All carcasses were skinned and fixed in 95% ethanol for 24 hours. Samples were then placed in 95% ethanol–Alcian blue (A5268, Sigma) and alizarin red solution (A5533, Sigma) (24, 48 hours respectively) for cartilage and bone staining, followed by a 95% ethanol wash (8 hours) and maceration in 1% KOH overnight at 4°C. Samples were cleared in 20%, 50%, 80% glycerol in 1% KOH for 12 hours for each step. Samples were stored in 100% glycerol for subsequent imaging.

### Histological tissue preparation and staining.

Histology and tissue preparation were performed as described previously. Briefly, pregnant mice were euthanized with CO_2_ followed by cervical dislocation. Embryos (E12.5–E18.5) were dissected from the uterus under a microscope. E0.5 was designated as noon of the day on which the dam was positive for the vaginal plug. Murine femurs and tibiae were harvested, skinned, and eviscerated before fixing in 4% paraformaldehyde (PFA) in 1× PBS overnight. For paraffin sections, specimen was twice dehydrated in 50% and then 70% ethanol solution and embedded in paraffin. Sections were cut at a thickness of 6 μm and mounted on Superfrost Plus slides (Thermo Fisher Scientific). For frozen sections, samples were infiltrated in 30% sucrose, embedded in optimum cutting temperature compound (23730571; Thermo Fisher Scientific), sectioned at 10 μm using a microtome (Leica LM 3095), and then mounted on Superfrost Plus slides.

### H&E staining.

Murine cranial bone paraffin sections were stained with H&E according to protocol (46, 124), then rehydrated. We stained the slides in hematoxylin. We rinsed using acid alcohol (1% HCl in 50% ethanol, EtOH) and counterstained with eosin. Slides were dehydrated in 100% EtOH and xylene.

### von Kossa staining.

To examine mineralization, sections were incubated in 1% silver nitrate for 30 minutes while under sunlight. Unreacted silver was washed with 5% sodium thiosulfate and distilled water for 5 minutes each, followed by counterstaining with nuclear fast red solution. Sections were dehydrated and coverslipped using permanent mounting medium.

### Alizarin red staining.

To examine mineralization, sections were stained with 2% alizarin red working solution for 20 minutes and washed with PBS for 5 minutes each. Sections were dehydrated and coverslipped using permanent mounting medium. For cell staining, we first washed the cells with PBS and then fixed the slides in 4% PFA. We added 2% alizarin red working solution and washed the cells.

### Immunostaining.

For immunohistochemistry staining (125, 126), the paraffin sections were used for the experiments. Sections were treated with 6% H_2_O_2_ in 100% methanol at room temperature (RT) for 5 hours to block endogenous peroxidase activity followed by rehydration through a descending methanol series (50%, 25%, and 0% in 0.1 M PBS) at RT for 30 minutes each. Antigen retrieval was performed by heating the tissues at 92°C–95°C for 5 minutes in Universal Antigen Retrieval Agent (CTS015; R&D Systems). We used 0.3% Triton X-100 in PBS (PBSX) to block nonspecific staining. Sections were then incubated with primary antibody in 10% normal donkey serum (NDS; D9663; Sigma-Aldrich) in PBSX at 4°C overnight (Supplemental Table 1). After rinses with PBS for 3 times, sections were incubated at 4°C for 24 hours with biotin-conjugated secondary antibody (BA-5000; Vector Laboratories) in 1% NDS in PBSX and then incubated at RT for 30 minutes with peroxidase-conjugated streptavidin in blocking solution (PK6200; Vector Laboratories). After 3 rinses in PBS, sections were preincubated in nickel-intensified DAB solution (SK4100; Vector Laboratories) without H_2_O_2_ at RT for 30 minutes followed by incubation with DAB solution containing 0.0003% H_2_O_2_. The reaction was stopped by PBS rinses twice and photographed in PBS. Counterstaining by hematoxylin was dependent on sections.

For immunofluorescence staining, frozen sections were air-dried at RT for 1 hour and rehydrated in 0.1 M PBS. Blocking of nonspecific staining was carried out by incubation with 10% NDS in 0.3% PBSX at RT for 30 minutes. Then, the sections were incubated with primary antibodies (Supplemental Table 1) in the carrier solution (5% normal donkey serum, 0.3% PBSX) at 4°C for overnight. Sections without a primary antibody treatment were used as negative control. Following 3 rinses in 0.1 M PBS, sections were incubated with Alexa Fluor 488– or 594–conjugated secondary antibody (1:500, Jackson ImmunoResearch) in carrier solution at RT for 1 hour. Following rinses with 0.1 M PBS, sections were counterstained with DAPI (200 ng/mL in PBS, D1306; Life Technologies) at RT for 10 minutes. After thorough rinsing in 0.1 M PBS, sections were air-dried and coverslipped with ProLong Diamond antifade mounting medium (P36970, Thermo Fisher Scientific). The sections were photographed using a laser scanning confocal microscope (Zeiss LSM 710).

### EdU analysis.

To collect tissues for EdU immunoreactions, EdU (B5002, Beyotime) was dissolved in Dulbecco’s phosphate-buffered saline (Cytiva) at 10 mg/mL and injected intraperitoneally at a single dose of 100 mg/kg 2 hours before embryo collection. EdU staining was performed based on the BeyoClick EdU-594 (C0078S, Beyotime). The Edu^+^ osteoprogenitor cells were counted using ImageJ (NIH) for data analysis. EdU^+^ cells were calculated from 3 samples using ImageJ.

### Apoptosis assay.

The apoptosis was determined by TUNEL labeling as described (11). The frozen sections were fixed and permeabilized, followed by TUNEL labeling using a One Step TUNEL Apoptosis Assay Kit (C1089, Beyotime) based on the protocol. The TUNEL^+^ cells were imaging using a confocal microscope (Zeiss LSM 710) and counted from 3 samples using ImageJ.

### Picrosirius red dye.

Sections first stained nuclei with hematoxylin for 5–10 minutes. We washed the slides in running tap water for 2 minutes. We put the slides in Picrosirius red (G1472, Solarbio) for 1 hour. Slides were dehydrated in 100% EtOH and xylene and mounted in Dendropanoxide (Thermo Fisher Scientific) to let dry overnight. Observation was under a bright-field or polarized microscope (SOPTOP and CX40P, both Ningbo Sunny Instruments Co., Ltd.).

### Cell culture and differentiation.

Primary CNC and calvarial osteoblast cell culture and osteogenic induction were performed as described (54). For CNCs, we harvested embryos E10.5 from the uterus and isolated the first branchial arch (BA1), then digested with TrypLE at 37°C for 10 minutes and centrifuged at 1,000*g*, and then discarded supernatant. We resuspended the cells with 100 μg/mL fibronectin solution at 37°C for 1.5 hours. Then we added 1 mL of Advanced DMEM including 10 μg/mL fibronectin and incubated at 37°C for 24 hours. We removed media, then cultured with Advanced DMEM/F-12 for several days. For osteoblast culture, calvarial bones were isolated from newborn mice or embryonic stage E18.5 from *Wnt1-Cre/Nf2-cKO* mutants and *Cre^−^ Nf2^fl/fl^* littermates using a dissecting microscope. After the removal of the periosteum and dura mater from the skull, the bones were then minced and digested using 0.2% dispase II and 0.1% collagenase in serum-free medium at 37°C. Digestion was carried out 3 times, and the first 2 digestions for 30 minutes were discarded. The last digestion for 90 minutes was pooled, centrifuged at 1,000*g*, and resuspended in α-MEM supplemented with 10% FBS and 100 infection units (IU)/mL Penicillin/Streptomycin (Gibco, Thermo Fisher Scientific) to reach cell confluence. For the osteogenic differentiation assay. Subsequently, cells were induced using osteogenic differentiation medium and BGJb medium (12591, Gibco, Thermo Fisher Scientific) supplemented with 10% (vol/vol) FBS, 50 μg/mL AA (A4544; Sigma-Aldrich), 5 mM glycerophosphate (G9891; Sigma-Aldrich), and 100 IU/mL Penicillin/Streptomycin (Gibco, Thermo Fisher Scientific). Osteoblasts were collected at different differentiation stages (d0, d7, d14).

### Phospho-Nf2 mutant and lentivirus packaging.

Overlapping PCR and pCDH-CMV-MCS plasmid was used to construct *Nf2* site mutation product (Ser10A, Thr230A, Ser315A, Ser518A), and we infected primary osteoblasts with lentivirus. Primer sequences are presented in Supplemental Table 2. For lentivirus packaging, the second-generation packaging plasmids (psPAX2 and pMD2.G) and lentivirus vector were cotransfected into HEK293T cells (3101HUMGNHu17, BMCR) using the Lipofectamine 8000 transfection reagent (Beyotime, C0533) following manufacturer’s instructions as described (11, 46), and the virus was harvested 48 hours and 72 hours posttransfection. Viral titers were determined in HEK293T cells, and lentivirus infection was performed by incubating primary cells with 5 IU per cell (about 1 × 10^5^ IU per well in a 24-well plate) in the presence of 10 μg/mL polybrene overnight.

### Protein transport assays and immunocytochemistry.

To analyze intracellular Col1a1 (114), cells were cultured for 12 hours after seeding and then treated or not treated with 50 μg/mL AA. Cells were fixed with 4% PFA for 20 minutes at RT and permeabilized with 0.3% PBSX for 15 minutes at RT. They were then blocked in 10% donkey serum in PBSX for 60 minutes at RT before staining with primary antibodies. The primary antibodies used were as follows: collagen type I (1:500, Abcam; ab21286), GM130 (1:500, Proteintech; 11308-1-Ig). The sections were photographed using a laser scanning confocal microscope (Zeiss LSM 710).

### TEM analysis.

Skull tissues were fixed for at least 24 hours in Karnovsky’s fixative, decalcified in 10% EDTA for 1 week, and then refixed for at least 24 hours in Karnovsky’s fixative. Samples were then secondarily fixed in 1% osmium tetroxide for 1 hour, dehydrated in increasing concentrations of EtOH, embedded in epoxy resin, sectioned at 100 nm thickness using an ultramicrotome (Leica EM UC7), stained with uranyl acetate and lead citrate, and imaged at 80 kV using a JEM-1400 Flash TEM equipped with a Gatan digital camera.

### Western blot assay.

Proteins from cultured osteoblasts at different differentiated stages from *Wnt1-Cre Nf2-cKO* mutants and *Cre^−^ Nf2^fl/fl^* littermate controls were extracted using RIPA buffer (1% NP-40, 150 mmol/L NaCl, 50 mmol/L Tris-HCl, 0.5% sodium deoxycholate, 0.1% SDS, 1 mmol/L EDTA, pH 7.4). The concentration of extracted proteins was determined using BCA protein assay kit (Pierce, Thermo Fisher Scientific). An amount of total protein was loaded into each lane. SDS-PAGE was used to resolve the protein bands and transferred to the PVDF membrane. Nonspecific binding was blocked using blocking buffer containing 5% milk powder in Tris-buffered saline and Tween-20 buffer (TBST) buffer (20 mM Tris pH 7.5, 150 mM NaCl, 0.1% Tween 20) at RT for 2 hours. The membranes were incubated with primary antibodies (Supplemental Table 1) in blocking buffer at 4°C for 24 hours. Following 3 rinses in TBST (15 min/each), membranes were incubated with conjugated secondary antibodies (Supplemental Table 1) in blocking buffer at RT for 2 hours. ChemiDocMP Imaging System (Bio-Rad ChemiDoc) was used to image the Western blot bands as described (11, 127). For Akt kinase activity assay, osteoblasts were treated or not treated with Akt inhibitor (SF2712) and extracted by RIPA. Phos-tag gel electrophoresis (198-17981, FUJIFILM) was used to resolve the protein bands and transfer to the PVDF membrane.

### co-IP assay.

For co-IP, cell lysates were prepared from cells cultured on a 10 cm dish in 500 μL co-IP buffer (25 mM Tris, 150 mM NaCl, 1% NP-40, 1 mM EDTA, 5% glycerol, pH 7.4) supplemented with proteinase and phosphatase inhibitor cocktail (P1050, Beyotime) (46). Cell lysates were incubated with co-IP antibody or normal IgG with rotation overnight at 4°C. Then Dynabeads (10001D, Thermo Fisher Scientific) were incubated for 30 minutes and then washed using DynaMag-2 magnet (12321D, Thermo Fisher Scientific) and eluted in SDS elution buffer (50 mM Tris, 2% SDS, 10% glycerol) at 60°C for 10 minutes. The supernatant containing co-IP products was supplemented with 100 mM DTT and 0.1% bromophenol blue, denatured at 95°C for 10 minutes, and submitted for Western blot analysis.

### RNA isolation and qRT-PCR analysis.

Total RNA was extracted from cultured cells at day 7 and day 14 as indicated with TRIzol reagent (15596018, Life Technologies, Thermo Fisher Scientific). Mouse cDNA was reversed-transcribed from 0.5 g total RNA with Hifair III 1st Strand cDNA Synthesis SuperMix for qPCR (11141ES10, YEASNE). The qRT-PCR was performed using 1-step RT-PCR System with Hieff qPCR SYBR Green Master Mix (11202ES03, YEASNE). Expression levels of each gene were given relative to Gapdh. The results are presented as means ± SD of triplicate. Primer sequences are presented in Supplemental Table 3.

### Statistics.

Data are presented as mean ± SD from at least 3 biological replicates. For comparisons between 2 groups, unpaired 2-tailed Student’s *t* test was used. For experiments involving more than 2 groups or 2 independent variables, 1-way or 2-way ANOVA was performed. A *P* value less than 0.05 was considered statistically significant. Statistical analyses were performed using GraphPad Prism 9 (GraphPad Software).

### Study approval.

All the animal experiments were approved by the animal committee of Zhejiang Sci-Tech University (201911001).

### Data availability.

Values for all data points in graphs can be found in the manuscript and Supporting Data Values file.

## Author contributions

Y Huang and JL contributed to experiment performance, data analysis, literature review formation, and writing. PS, Y He, FS, QZ, and CZ contributed to data analysis on molecular subcloning. Y Huang, XZ, and HL contributed to revision and literature review formation. GC contributed to data analysis, revision, literature review formation, supervision, and funding grant acquisition.

## Supplementary Material

Supplemental data

Unedited blot and gel images

Supporting data values

## Version 1 09/23/2025

Electronic publication
