# Supplementary material for: Nf2/FGFR1/AKT axis directs cranial neural crest–derived skull morphogenesis via collagen synthesis and trafficking
Source: JCI Insight. 2025 Sep 23;10(18):e191112. doi: 10.1172/jci.insight.191112 (PMC12487865; doi:10.1172/jci.insight.191112)
Supplement: Supplemental data [file jciinsight-10-191112-s074.pdf]

## Supplemental Tables and Figures

**Table S1** Primary antibodies used for immunohistochemistry or Western Blotting

| Primary antibody | Cat. number | Source | Dilution |
|---|---|---|---|
| Nf2/merlin | 12888S | Abcam, Cambridge, United Kingdom | 1:1000(WB) |
| Nf2/merlin | AF7563 | Beyotime Biotechnology, Shanghai, China | 1:100(IF) |
| GAPDH | 60004-1-Ig | Proteintech, Wuhan, China | 1:5000(WB) |
| Runx2 | AF2593 | Beyotime Biotechnology, Shanghai, China | 1:1000(WB) 1:500(IF) |
| Sp7 | ab227820 | Abcam, Cambridge, United Kingdom | 1:1000(WB) 1:500(IF) |
| GM130 | 11308-1-Ig | Proteintech, Wuhan, China | 1:500(IF) |
| COL1 | ab21286 | Abcam, Cambridge, United Kingdom | 1:500(IF) |
| COL1 | AF1840 | Beyotime Biotechnology, Shanghai, China | 1:1000(WB, IHC) |
| COL1 | SP1.D8 | Developmental Studies Hybridoma Bank, DHSB | 1:100(IF) |
| AKT | 9272S | Cell Signaling Technology, Danvers, MA | 1:1000(WB) 1:500(IF) |
| P-AKT(S473) | 4060S | Cell Signaling Technology, Danvers, MA | 1:1000(WB) 1:100(IF) |
| FGFR1 | 60325-1-Ig | Proteintech, Wuhan, China | 1:1000(WB) 1:100(IF) |
| PLCγ | 2822S | Cell Signaling Technology, Danvers, MA | 1:1000(WB) |
| P-PLCγ | 3285S | Cell Signaling Technology, Danvers, MA | 1:1000(WB) |
| GRP78 BiP | 200310-4F11 | Zen-bio, Chengdu, China | 1:1000(WB) |
| ATF4 | R381426 | Zen-bio, Chengdu, China | 1:1000(WB) |
| FLAG | 1479S | Cell Signaling Technology, Danvers, MA | 1:1000(WB) |
| HA | 3724S | Cell Signaling Technology, Danvers, MA | 1:1000(WB) |
| FLAG | 66008-4-Ig | Proteintech, Wuhan, China | 1:1000(WB) |
| HA | 66006-2-Ig | Proteintech, Wuhan, China | 1:1000(WB) |
| Normal Rabbit IgG | 2729 | Cell Signaling Technology, Danvers, MA | 1:1000(WB) |
| Alexa Fluor 488-labeled Goat Anti-Rabbit IgG(H+L) | 111-545-144 | Jackson ImmunoResearch, West Grove, PA | 1:500 |
| Alexa Fluor 594-labeled Goat Anti-Rabbit IgG(H+L) | 705-585-003 | Jackson ImmunoResearch, West Grove, PA | 1:500 |
| Alexa Fluor 488-labeled Goat Anti-Mouse IgG(H+L) | 715-545-150 | Jackson ImmunoResearch, West Grove, PA | 1:500 |
| Alexa Fluor 594-labeled Goat Anti-Mouse IgG(H+L) | 715-585-150 | Jackson ImmunoResearch, West Grove, PA | 1:500 |
| Peroxidase AffiniPure Donkey Anti-Rabbit IgG (H+L) | 711-035-152 | Jackson ImmunoResearch, West Grove, PA | 1:500 |
| Peroxidase AffiniPure | 715-035-150 | Jackson ImmunoResearch, West | 1:500 |

| | | | |
|---|---|---|---|
| Donkey Anti-Mouse IgG (H+L) | | Grove, PA | |
| Goat Anti-Rabbit IgG (H+L)-Biotinylated | BA-1000-1.5 | Vector Laboratories, Newark, CA | 1:500 |

**Table S2** Primers used in Nf2 site mutation construction.

| Gene | Forward (5'-3') | Reverse (5'-3') |
|---|---|---|
| *overlap* | *tagagctagcgaaTTATGGCCGGAGCCATCG* | *tcgcggccgcggatcTCAAATGCAGATAGGTCTTCTGCCTTG* |
| *Ser10A* | *TCTCGCATGAGATTCAGCTCA* | *TGAGCTGAATCTCATGCGAGA* |
| *Thr230A* | *TAAAAAGGGCGCGGAGTTGCTGC* | *GCAGCAACTCCGCGCCCTTTTTA* |
| *Ser315A* | *AAAGCTGACGCTTTAGAAGTT* | *AACTTCTAAAGCGTCAGCTTT* |
| *Ser518A* | *AGCGACTTTCTATGGAGAT* | *TATCTCCATAGAAAGTCGCT* |
| *Ser10D* | *TTCTCGCATGGACTTCAGCTCA* | *TGAGCTGAAGTCCATGCGAGAA* |
| *Thr230D* | *TAAAAAGGGCGACGAGTTGCTGC* | *GCAGCAACTCGTCGCCCTTTTTA* |

**Table S3** Primers used in qRT-PCR analysis.

| Gene | Forward (5'-3') | Reverse (5'-3') |
|---|---|---|
| *Nf2* | *CATGAGCTTCAGCTCACTCAAGAGGAAG* | *ATCCCCGCTTGTGCACAGAGGGGTCATAG* |
| *Alp* | *GGACAGGACACACACACACA* | *CAAACAGGAGAGCCACTTCA* |
| *OCN* | *TGAGCTTAACCCTGCTTGTG* | *TAGGGCAGCACAGGTCCTA* |
| *Col1* | *ATAGCTCGTCACAAGCAGGG* | *TGACAAAGCCTTCATGTCCA* |
| *Sp7* | *CCTACTTACCCATCTGACTTTGCT* | *CTTATAGACATCTTGGGGTAGGACA* |

## Supplemental Figures

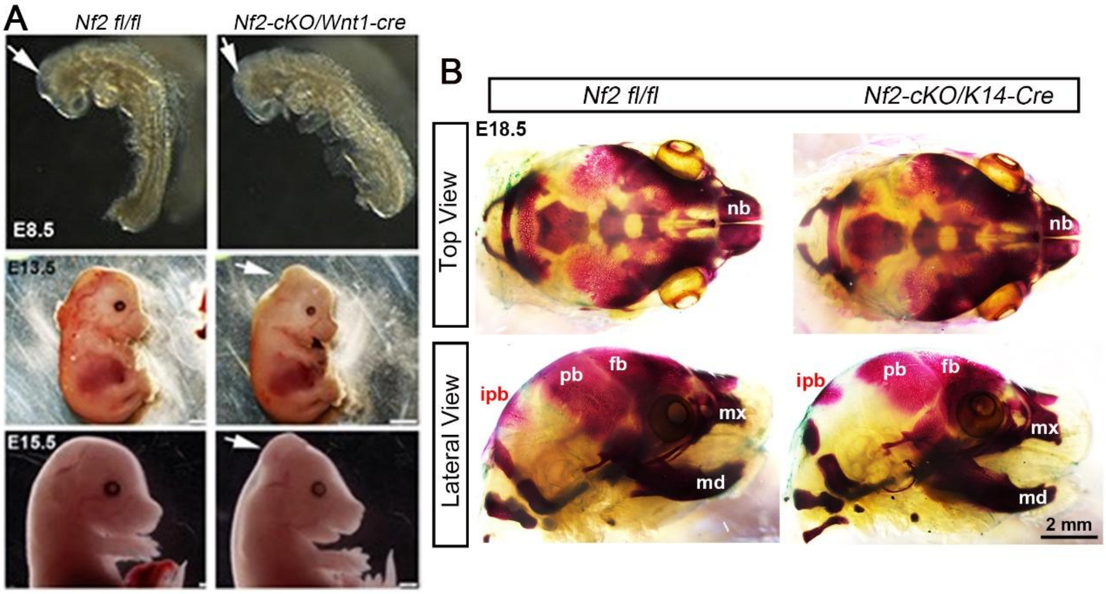


**Fig 1S** ***Epithelial-specific Nf2 deletion reveals normal craniofacial bone development.***

**A:** The appearance of the phenotype of the cranial neural crest-specific *Nf2* deletion at E8.5, E13.5 and E15.5, with visible midfacial hypoplasia. Scale bars: 2 mm.

**B:** Alizarin red-stained skulls of the *Nf2 fl/fl* and *Nf2-cKO/K14-Cre* mice at E18.5 show normal craniofacial bone development. fb, frontal bone; pb, parietal bone; ib, interparietal bone; oc, occipital bone; na, nasal bone; ma, mandible; mx, maxilla.
Scale bars: 2 mm.

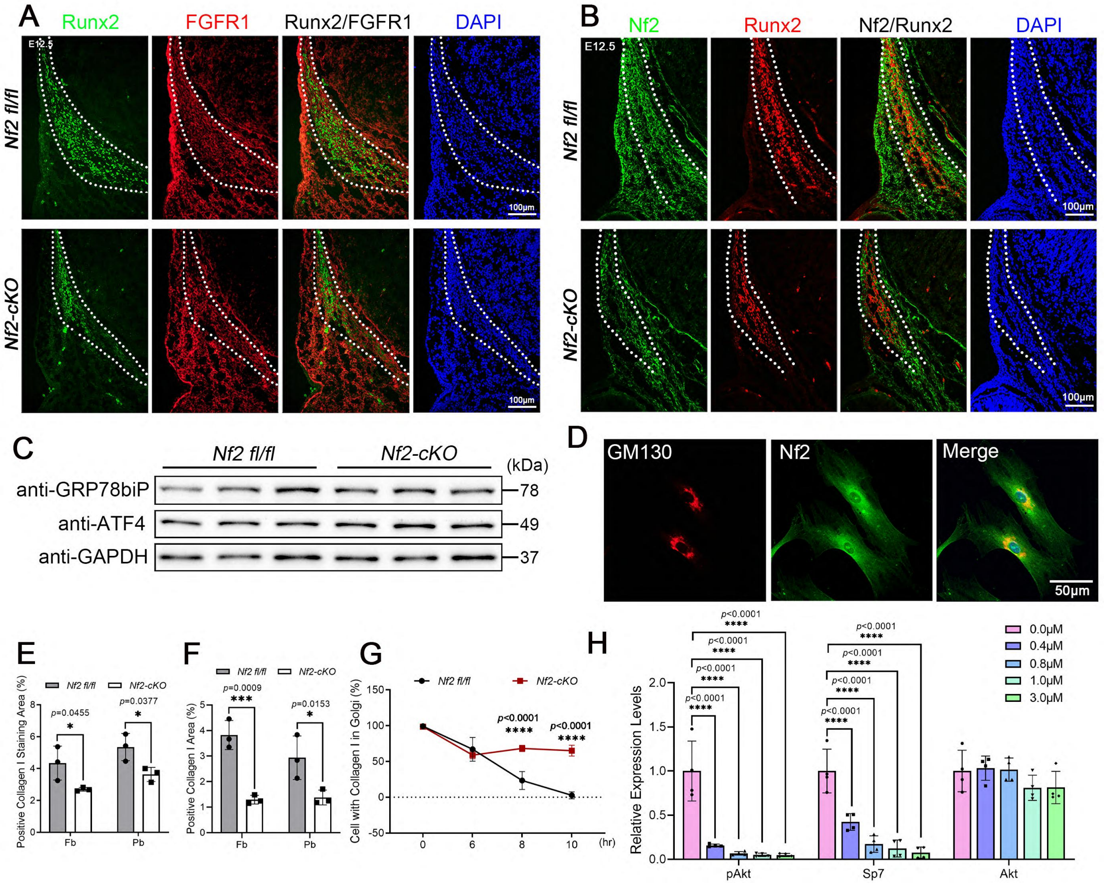


**Fig 2S Localization of FGFR1 and Nf2 in early osteoprogenitors and analysis of ER stress in *Nf2* mutants.**

<u>A:</u> Co-localization of Runx2$^{+}$ osteoprogenitors and FGFR1 in E12.5 coronal sections (dotted lines demarcate osteogenic zone). *Nf2* mutant mice maintains normal FGFR1 expression in Runx2$^{+}$ osteoprogenitors. Scale bars: 100 µm.

<u>B:</u> Co-localization of Runx2$^{+}$ osteoprogenitors and Nf2 in E12.5 coronal sections (dotted lines demarcate osteogenic zone). *Nf2* mutant mice maintains normal FGFR1 expression in Runx2$^{+}$ osteoprogenitors. Scale bars: 100 µm.

<u>C:</u> Western blot analysis of ER stress sensor molecules in *Nf2* mutant CNC-derived osteoblasts.

<u>D:</u> Colocalization of Nf2 and GM130 was analyzed by immunofluorescence staining in *Nf2* mutant CNC-derived osteoblasts. Scale bars: 50 µm.

<u>E:</u> Quantification of positive Collagen I staining area in *Nf2* mutant.

<u>F:</u> Quantification of positive Collagen I area by polarized Sirius Red staining in polarized light.

<u>G:</u> Quantification of the percentage of the cells with collagen I in the Golgi.

<u>H:</u> Quantification of expression level of pAkt, Sp7 and Akt (normalized to GAPDH).

Data were expressed as means ± SD and each dot represents an individual biological replicate. *P* values were calculated by unpaired Student's *t*-test with two-tailed

analysis without adjustments (E, F and G) and 2-way ANOVA with Tukey's multiple-comparison test (H). *$p < 0.05$, **$p < 0.01$, ***$p < 0.001$, ****$p < 0.0001$.

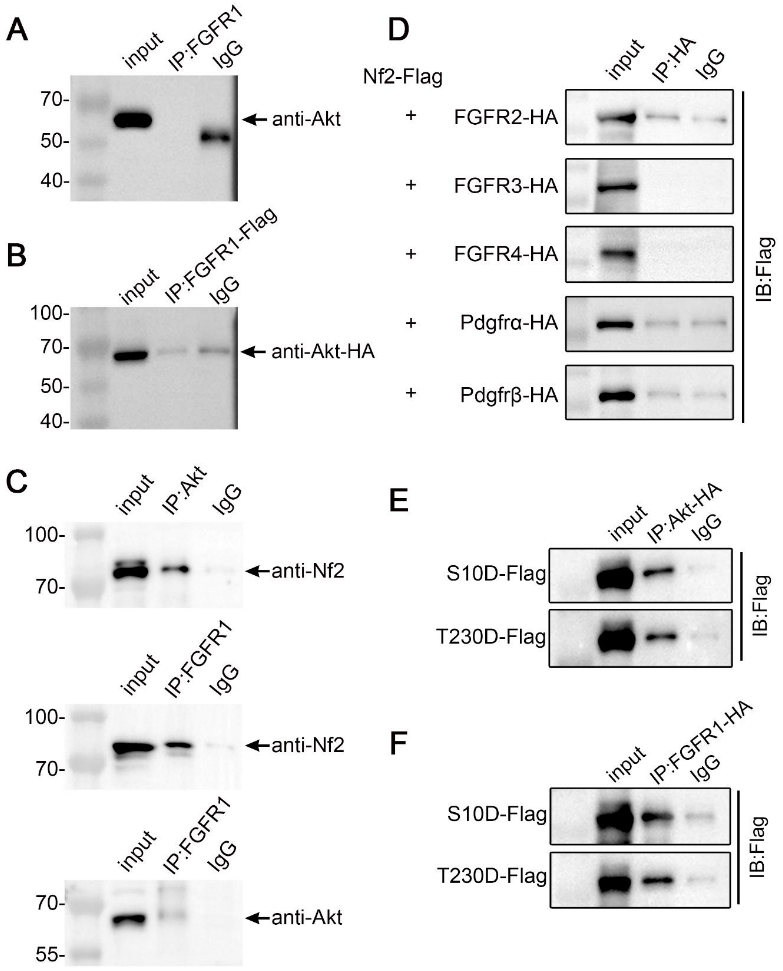


**Fig 3S Different receptors were evaluated the interactions with Nf2.**

**A-B:** No direct FGFR1-Akt complex in primary osteoblasts (**B**) and HEK293T cells using co-IP assay.

**C:** co-IP assay was performed with HEK293T cells transfected with the indicated plasmids, *FGFR1-Flag*, *Akt-HA*, and co-expression of Nf2. co-IP was performed using FGFR1, Akt and Nf2 antibody. Western blot analysis showed that Nf2 interacts with both FGFR1 and Akt individually. However, no interaction was detected between *FGFR1-Flag* and *Akt-HA*, suggesting that Nf2 does not bridge FGFR1 and Akt into a stable ternary complex under the tested conditions.

**D:** co-IP assay showed that *Nf2-Flag* and *FGFR2-HA*, *FGFR3-HA*, *FGFR4-HA*, *Pdgfrα-Flag*, *Pdgfrβ-Flag* could not form an interaction in HEK293T cells.

**E:** co-IP assay was performed with HEK293T cells transfected with the indicated plasmids. The same amount of tagged Nf2 single site continuous phosphorylation plasmids (*S10D-Flag, T230D-Flag*) and *Akt-HA* plasmids were co-transfected. Immunoprecipitation was performed using a FLAG, HA antibody.

**F:** co-IP assay was performed with HEK293T cells transfected with the indicated

plasmids. The same amount of tagged Nf2 single site continuous phosphorylation plasmids (*S10D-Flag, T230D-Flag* and *FGFR1-HA* plasmids were co-transfected. Immunoprecipitation was performed using a FLAG, HA antibody.
