# Supplementary material for: Nf2/FGFR1/AKT axis directs cranial neural crest–derived skull morphogenesis via collagen synthesis and trafficking
Source: JCI Insight. 2025 Sep 23;10(18):e191112. doi: 10.1172/jci.insight.191112 (PMC12487865; doi:10.1172/jci.insight.191112)
Supplement: Unedited blot and gel images [file jciinsight-10-191112-s075.pdf]

Uncropped gels for Western Blots

Figure 3A

Figure 3A

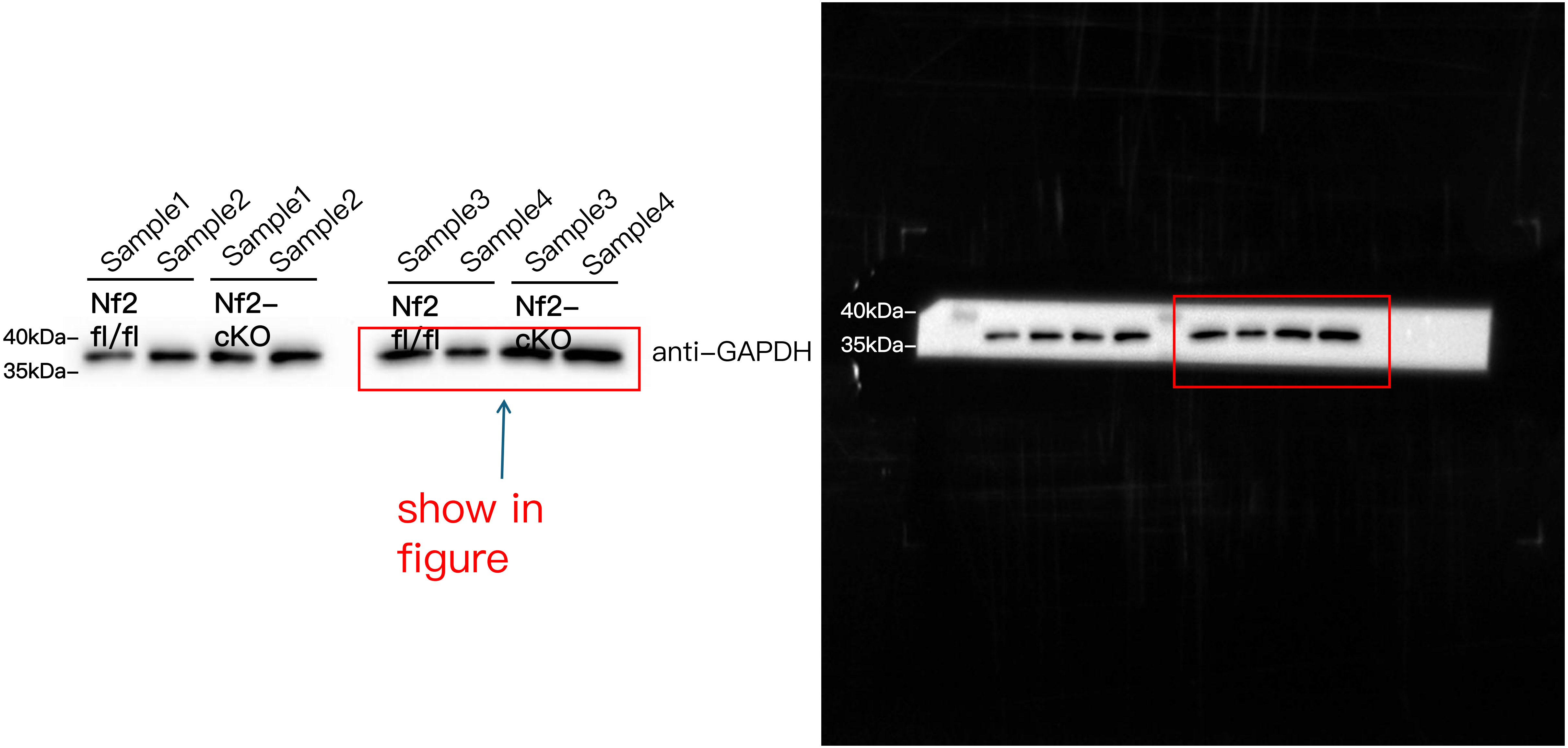

Sample1
Sample2
Sample1
Sample2
Sample3
Sample4
Sample3
Sample4
Nf2 fl/fl
Nf2–cKO
Nf2 fl/fl
Nf2–cKO
40kDa–
35kDa–
anti–GAPDH
show in figure
40kDa–
35kDa–

Figure 3A

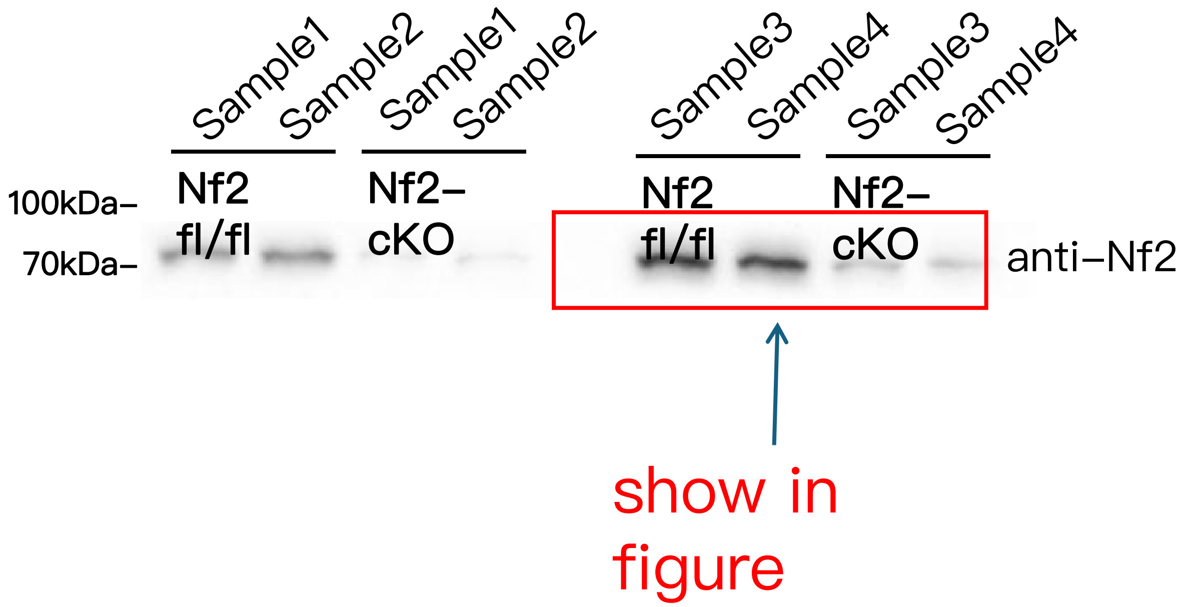

Sample1
Sample2
Sample1
Sample2
Sample3
Sample4
Sample3
Sample4
Nf2 fl/fl
Nf2– cKO
Nf2 fl/fl
Nf2– cKO
100kDa–
70kDa–
anti–Nf2
show in figure

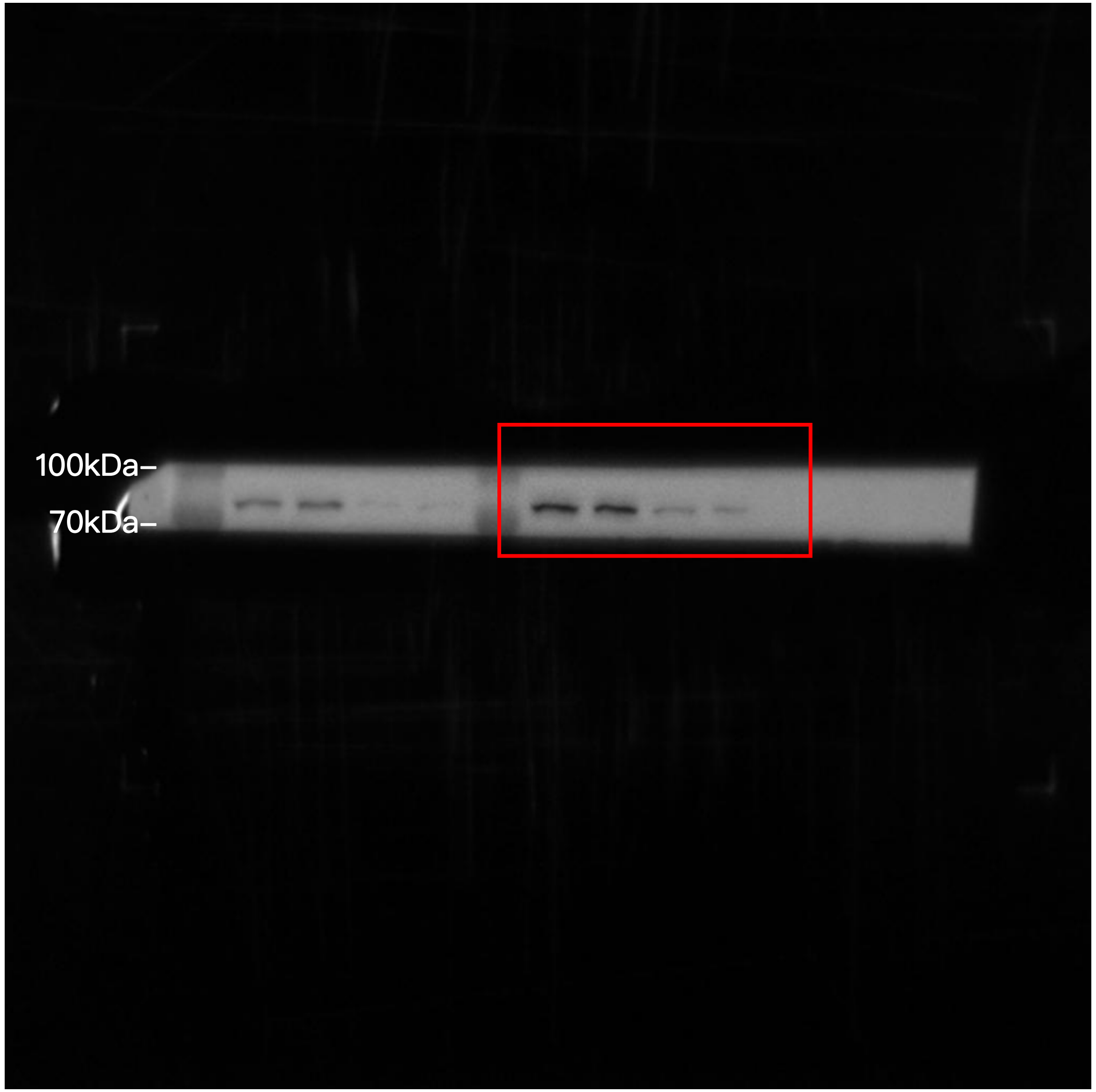

100kDa–
70kDa–

Figure 3A

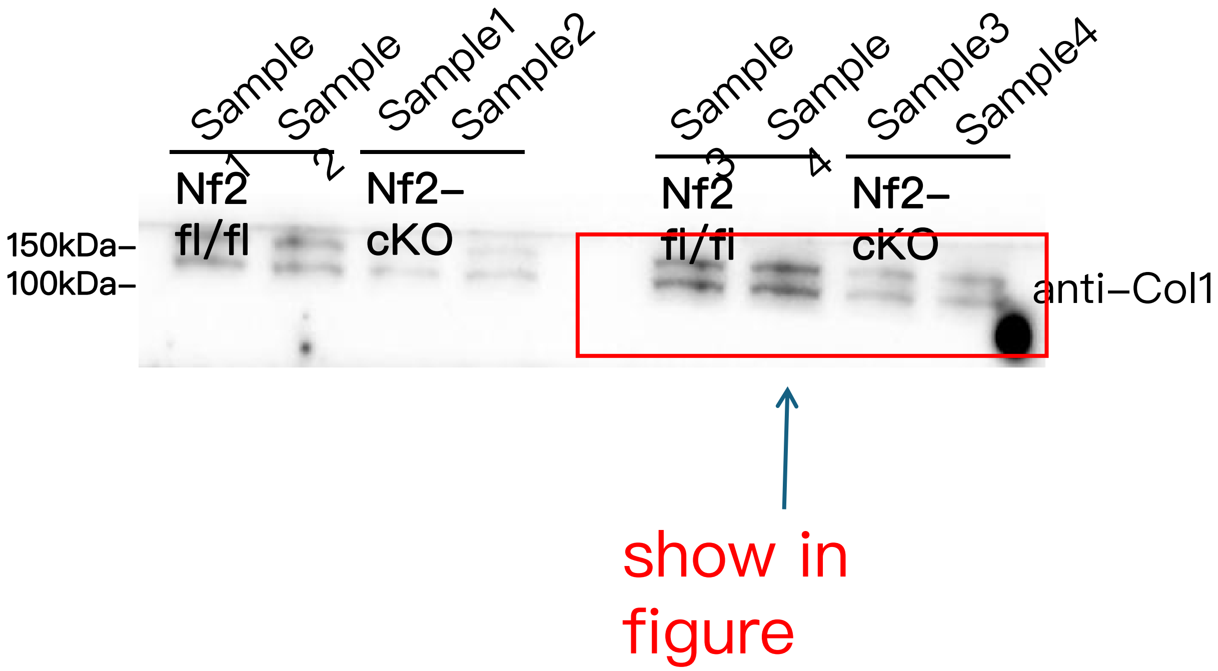
Sample 1
Sample 2
Sample1
Sample2
Sample 3
Sample 4
Sample3
Sample4
Nf2 fl/fl
Nf2–cKO
Nf2 fl/fl
Nf2–cKO
150kDa–
100kDa–
anti–Col1
show in figure

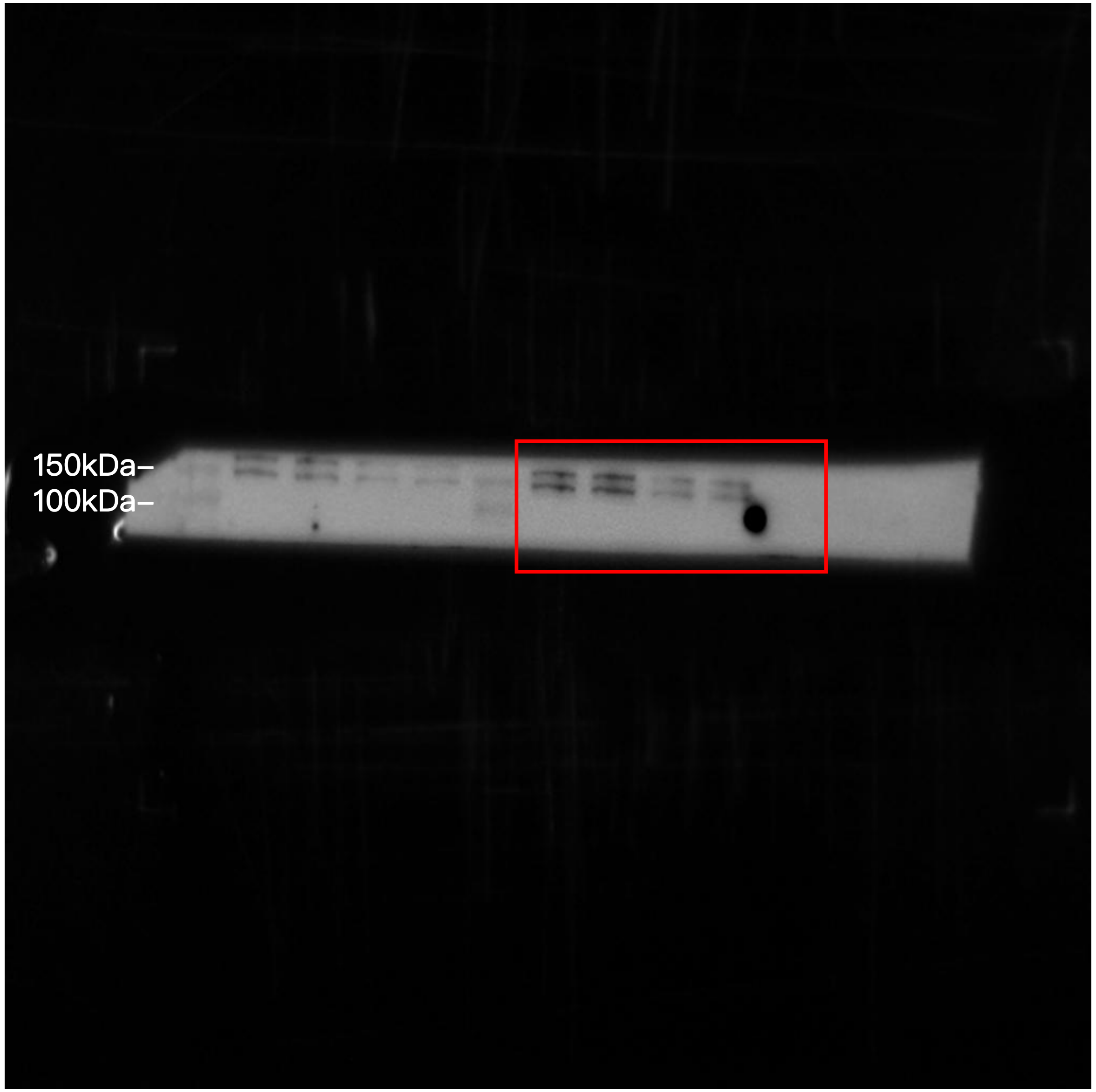
150kDa–
100kDa–

Figure 3A

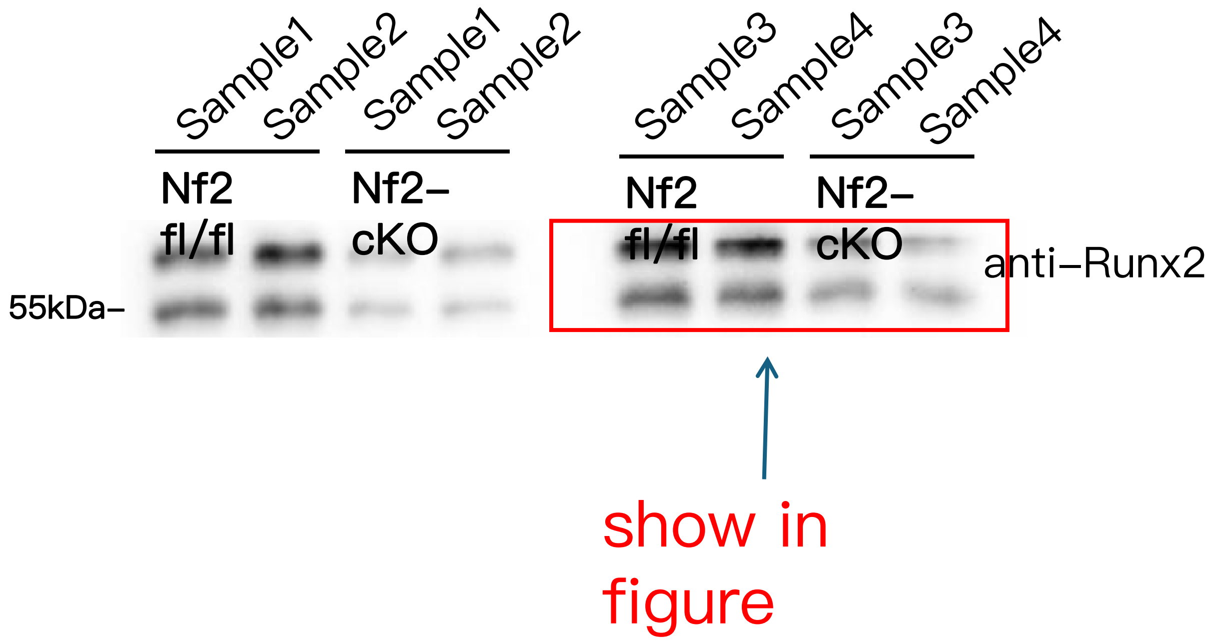
Sample1
Sample2
Sample1
Sample2
Sample3
Sample4
Sample3
Sample4
Nf2 fl/fl
Nf2-cKO
Nf2 fl/fl
Nf2-cKO
55kDa-
anti-Runx2
show in figure

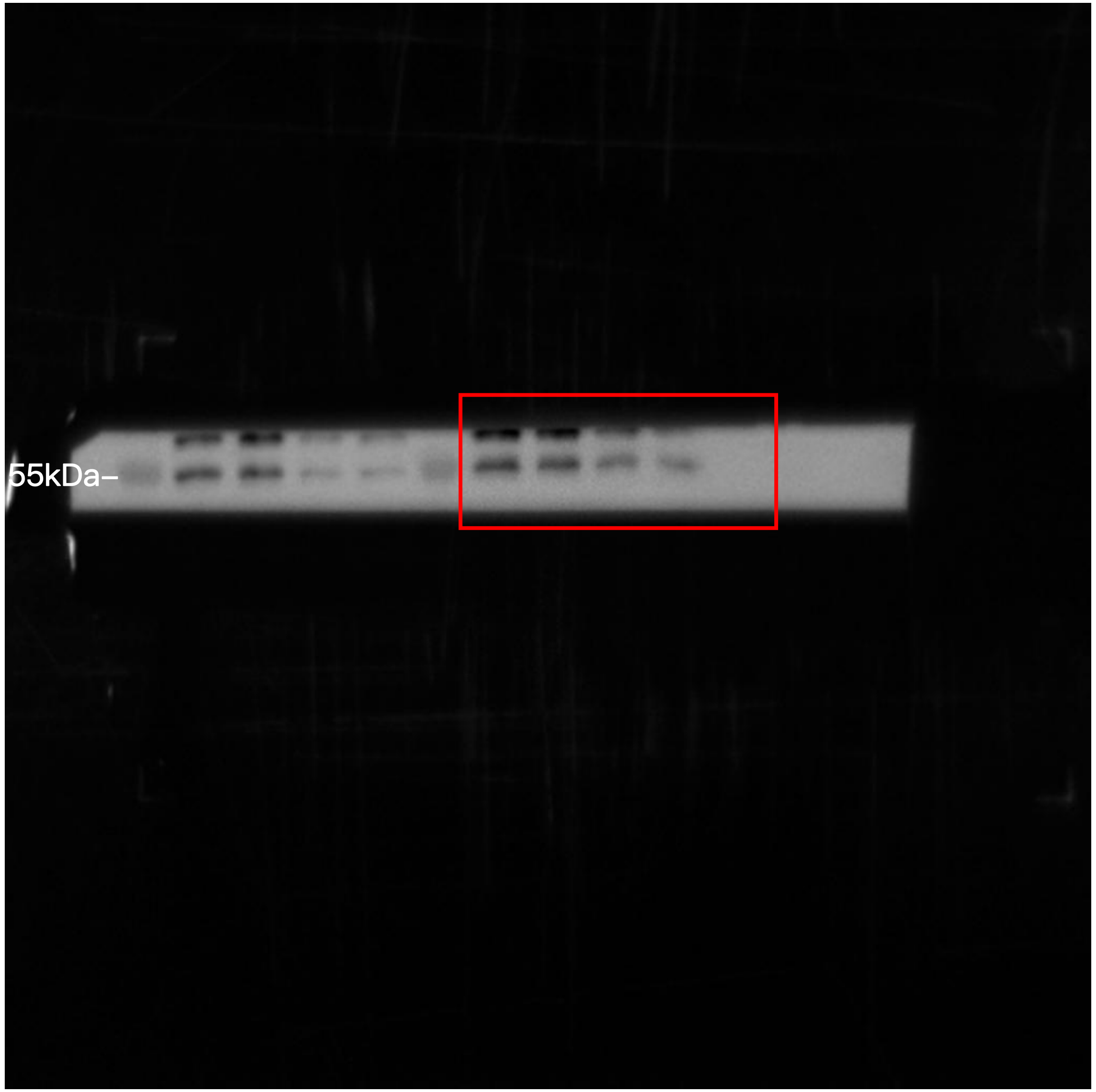
55kDa-

Figure 3J

Figure 3J Repeat1

show in figure

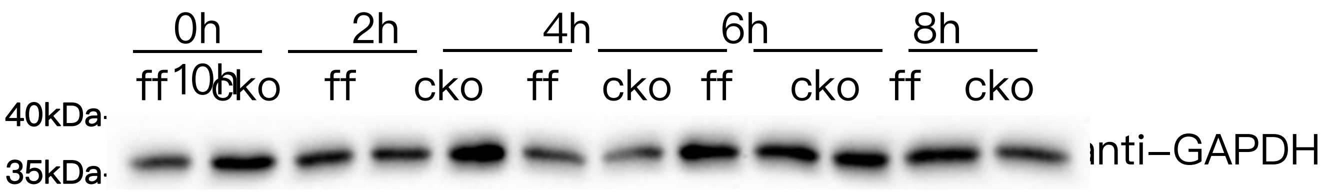

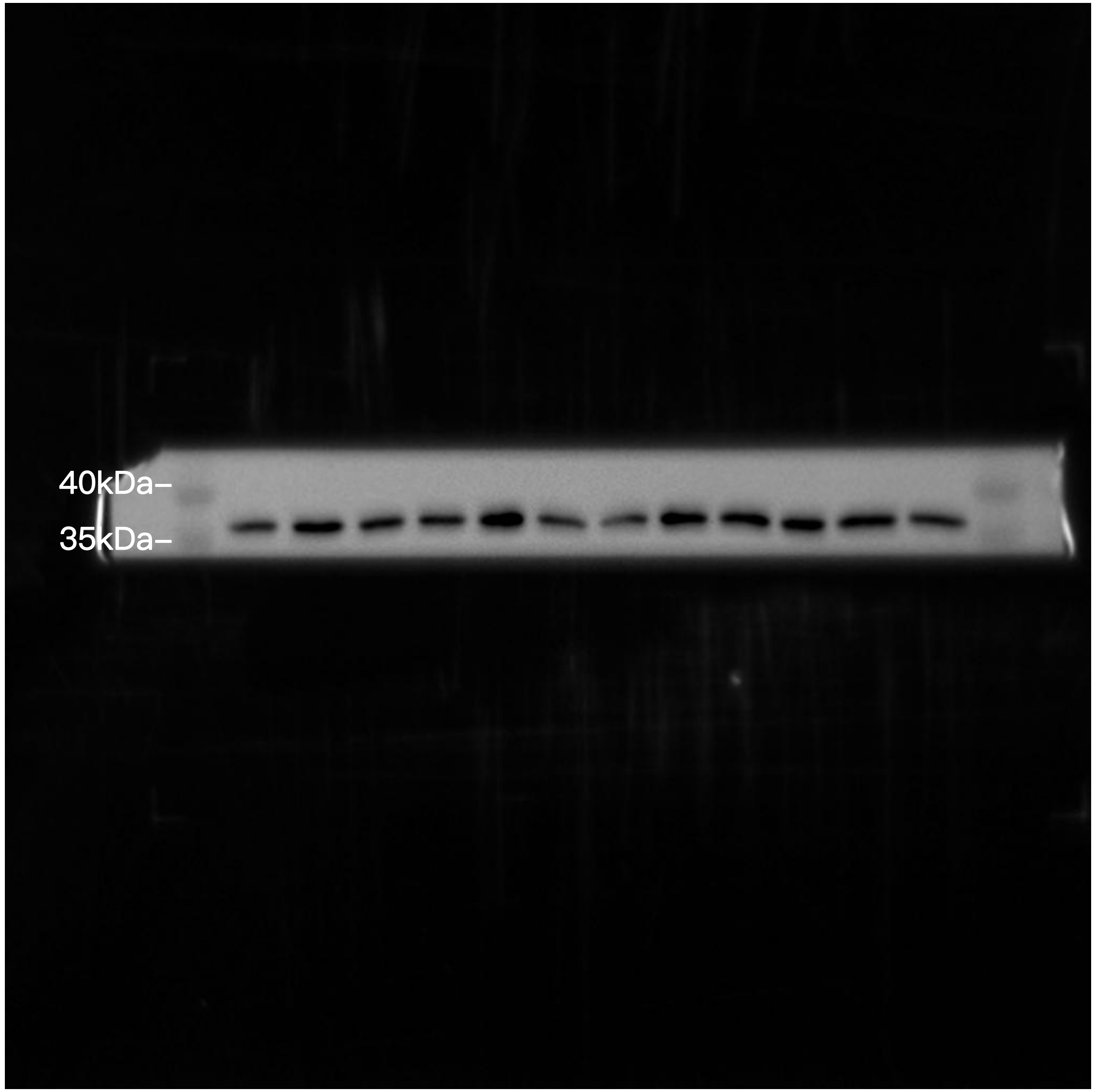

Figure 3J Repeat1

show in figure

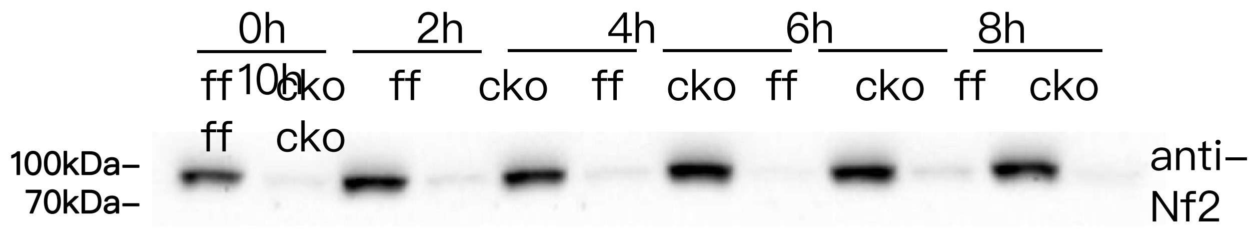

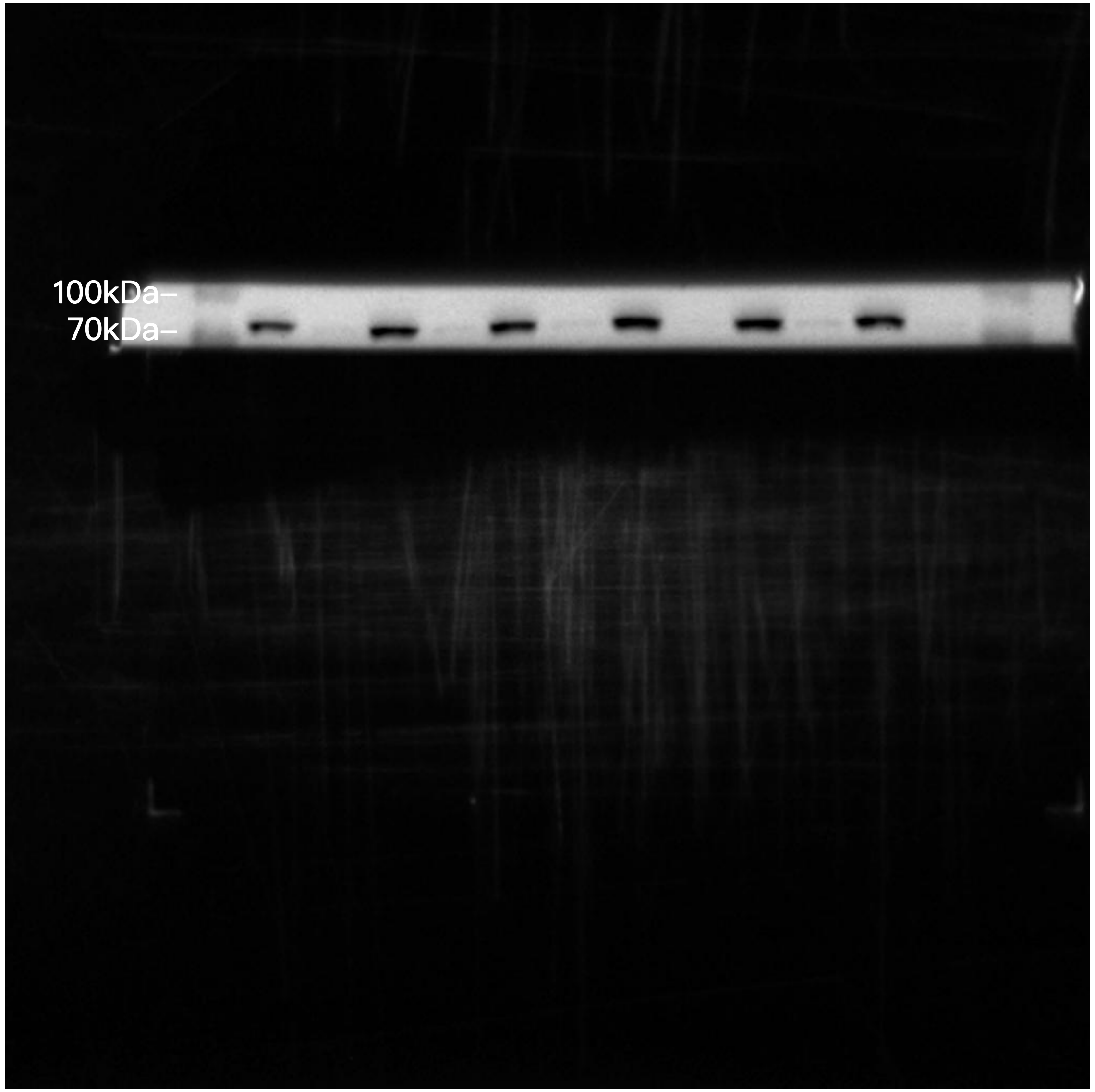

Figure 3J Repeat1

show in figure

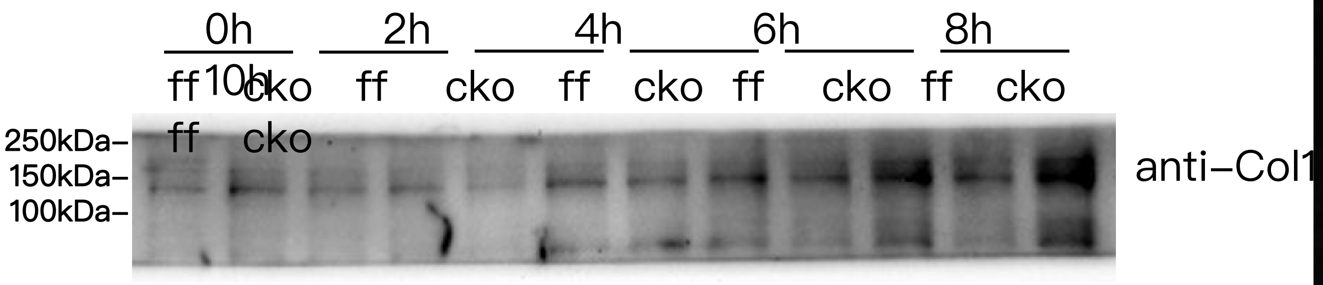

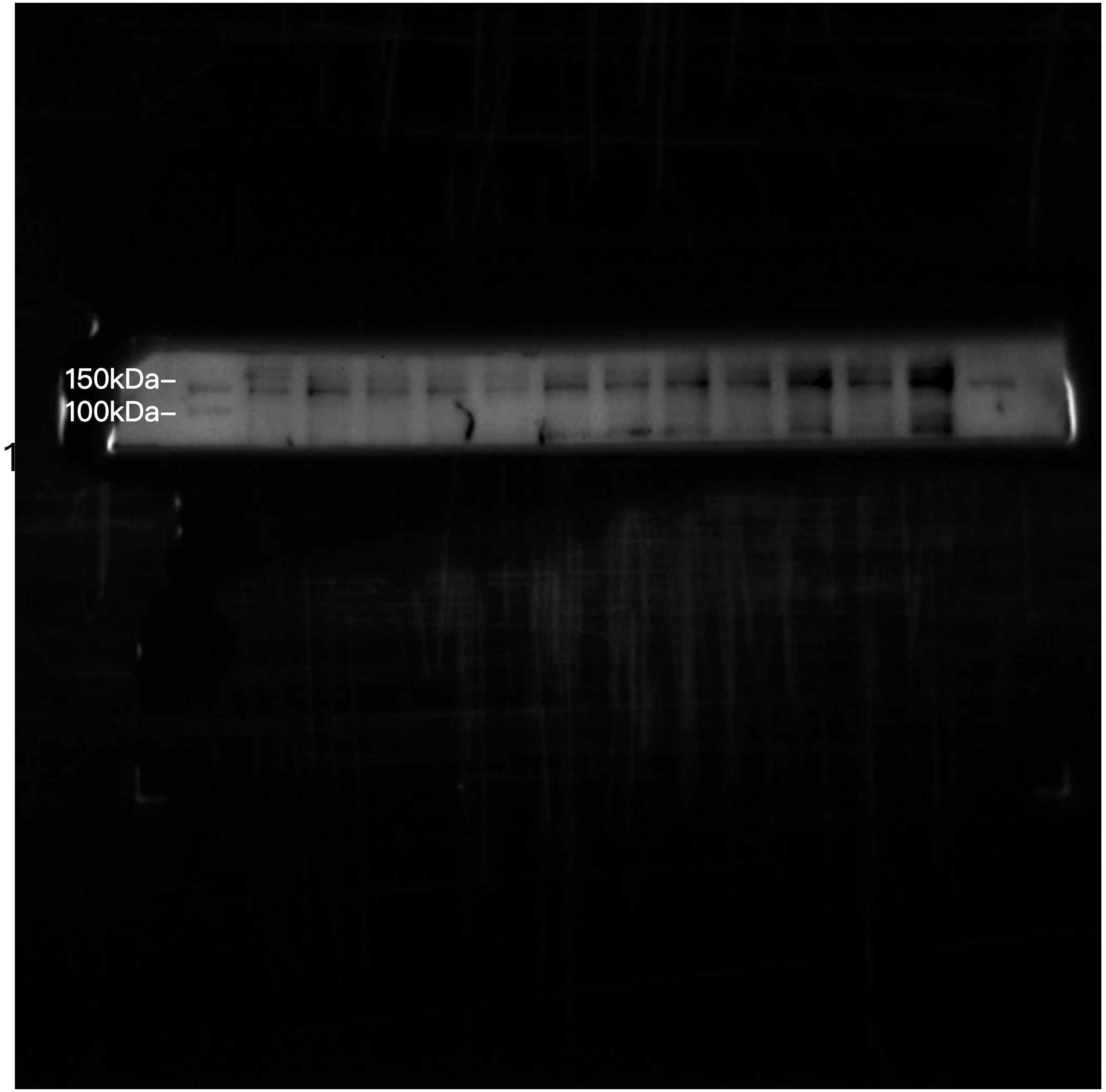

Figure 3J Repeat2

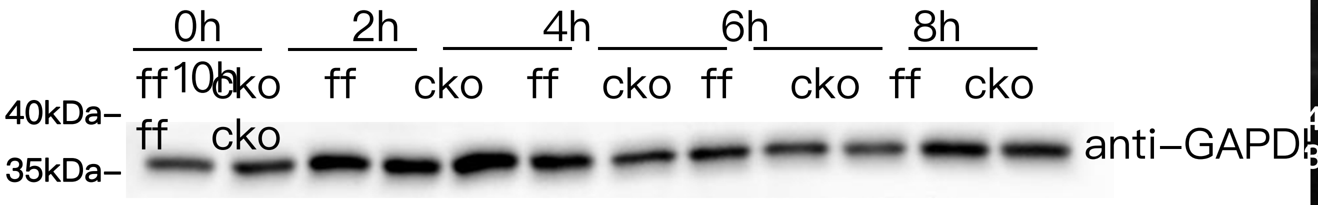

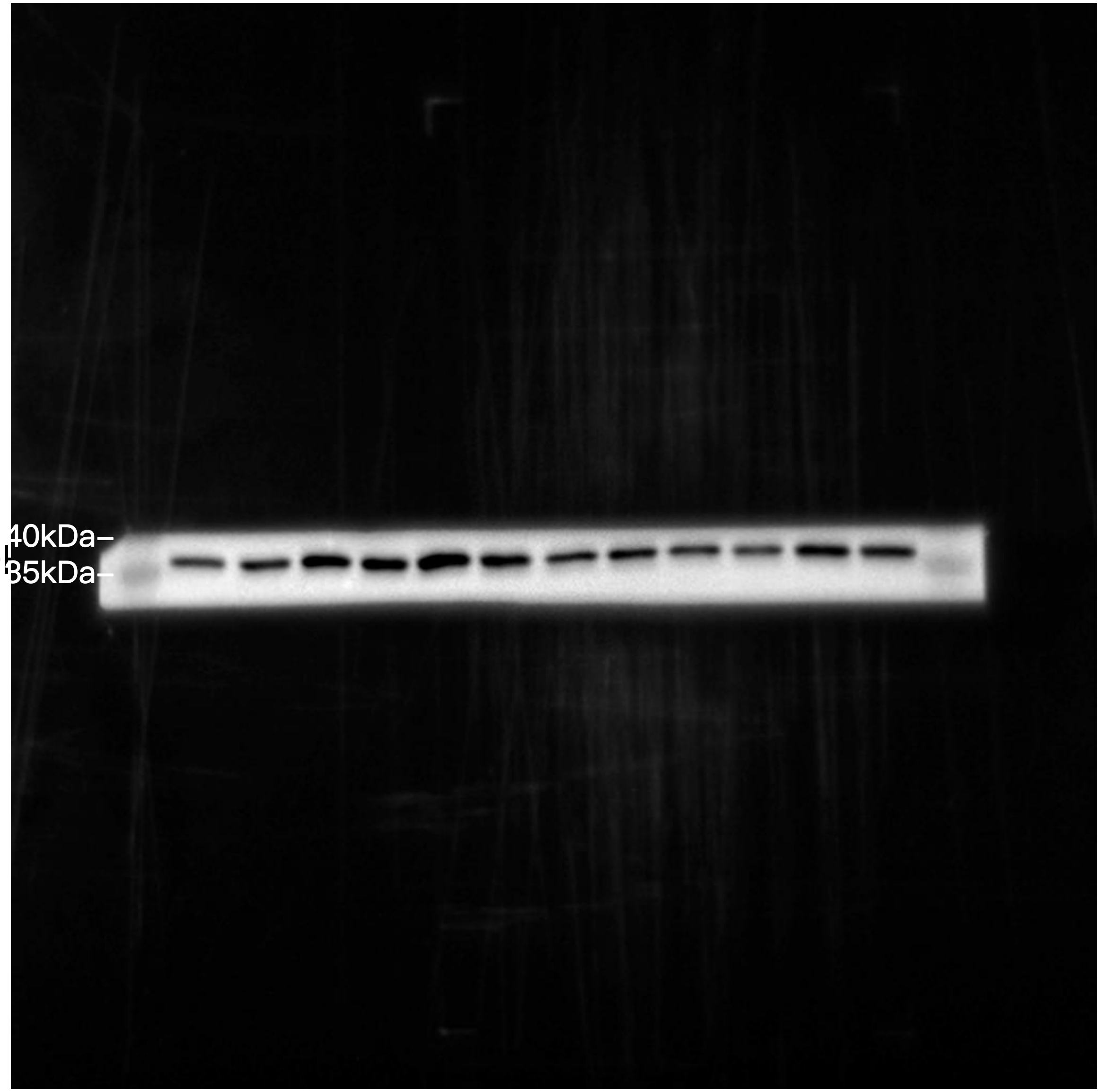

Figure 3J Repeat2

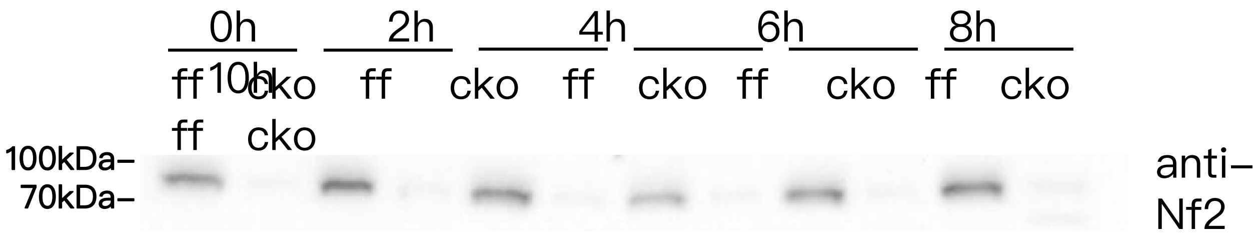

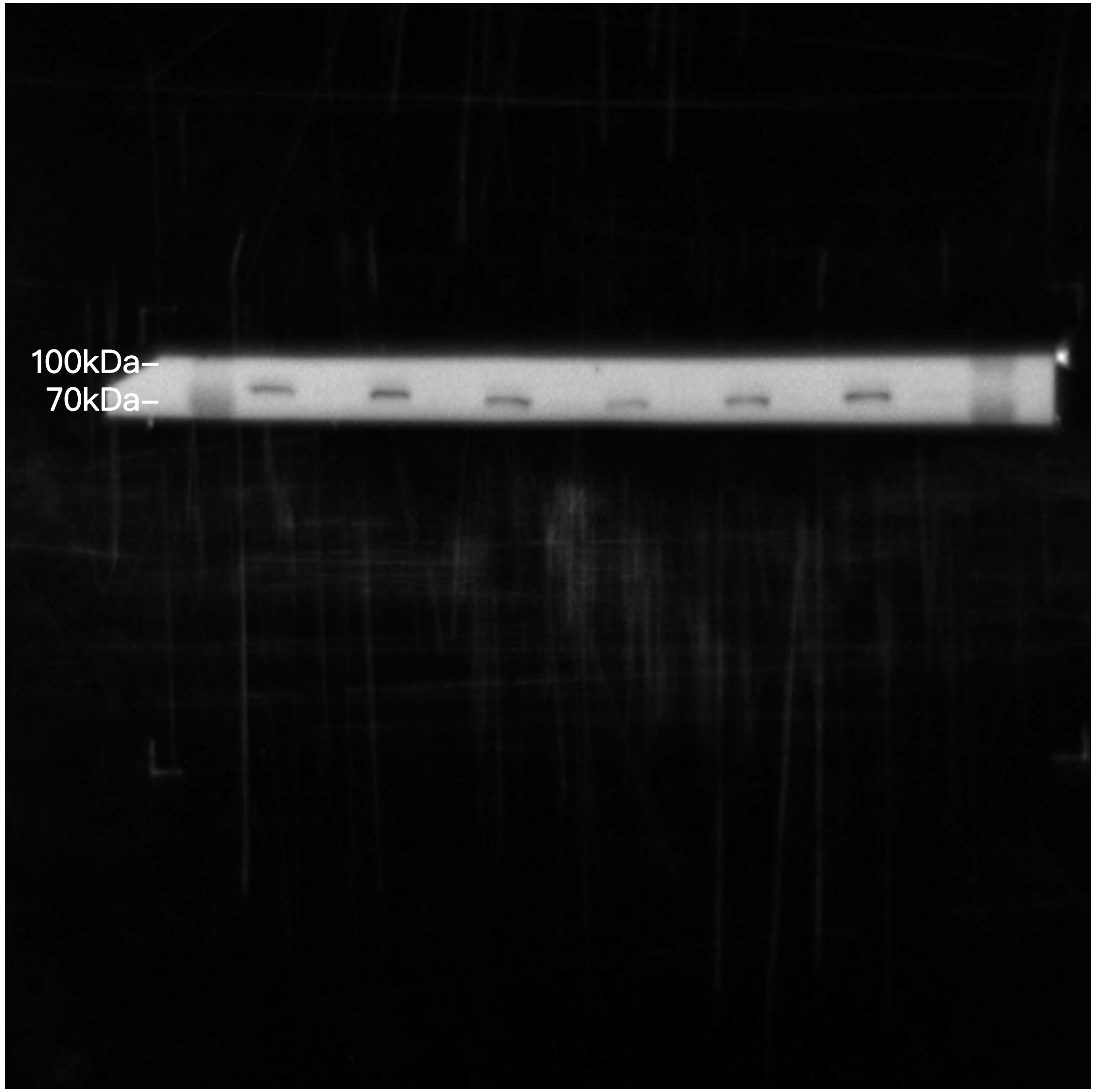

Figure 3J Repeat2

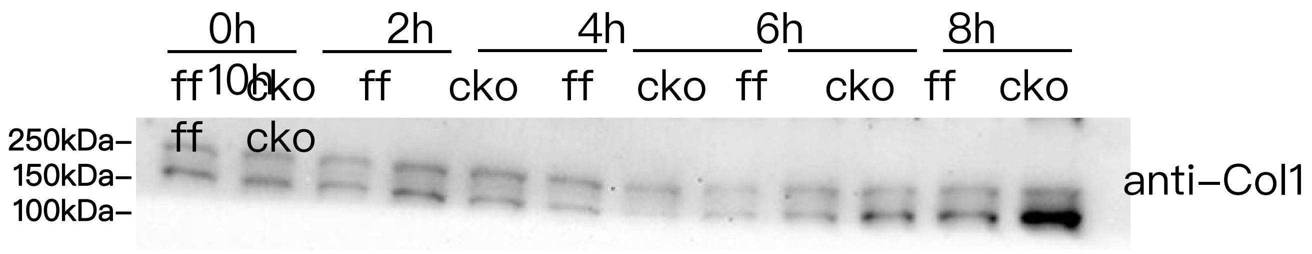

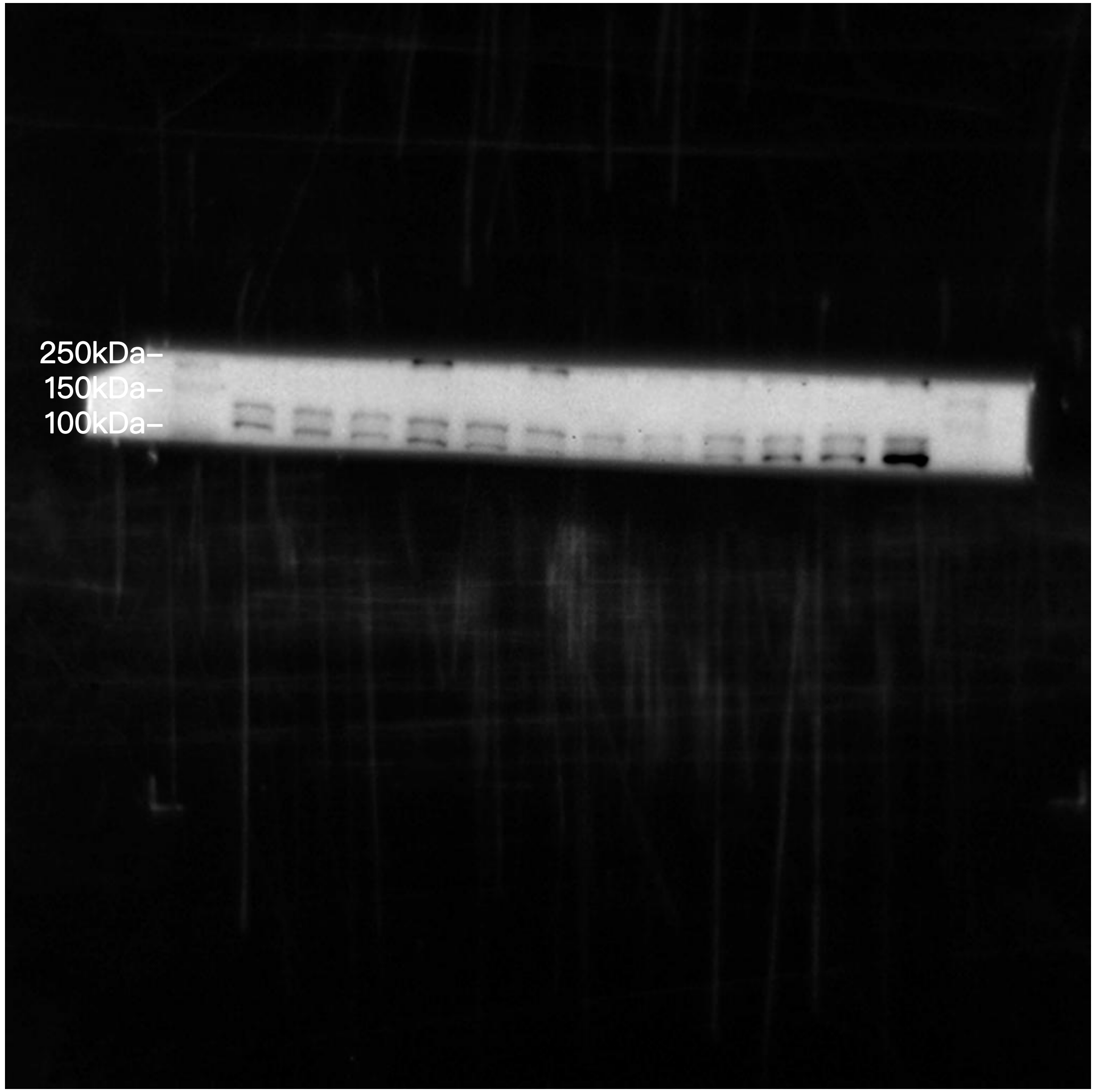

Figure 3J Repeat3

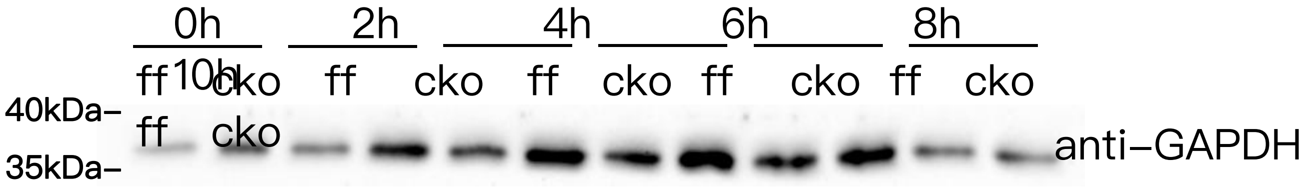

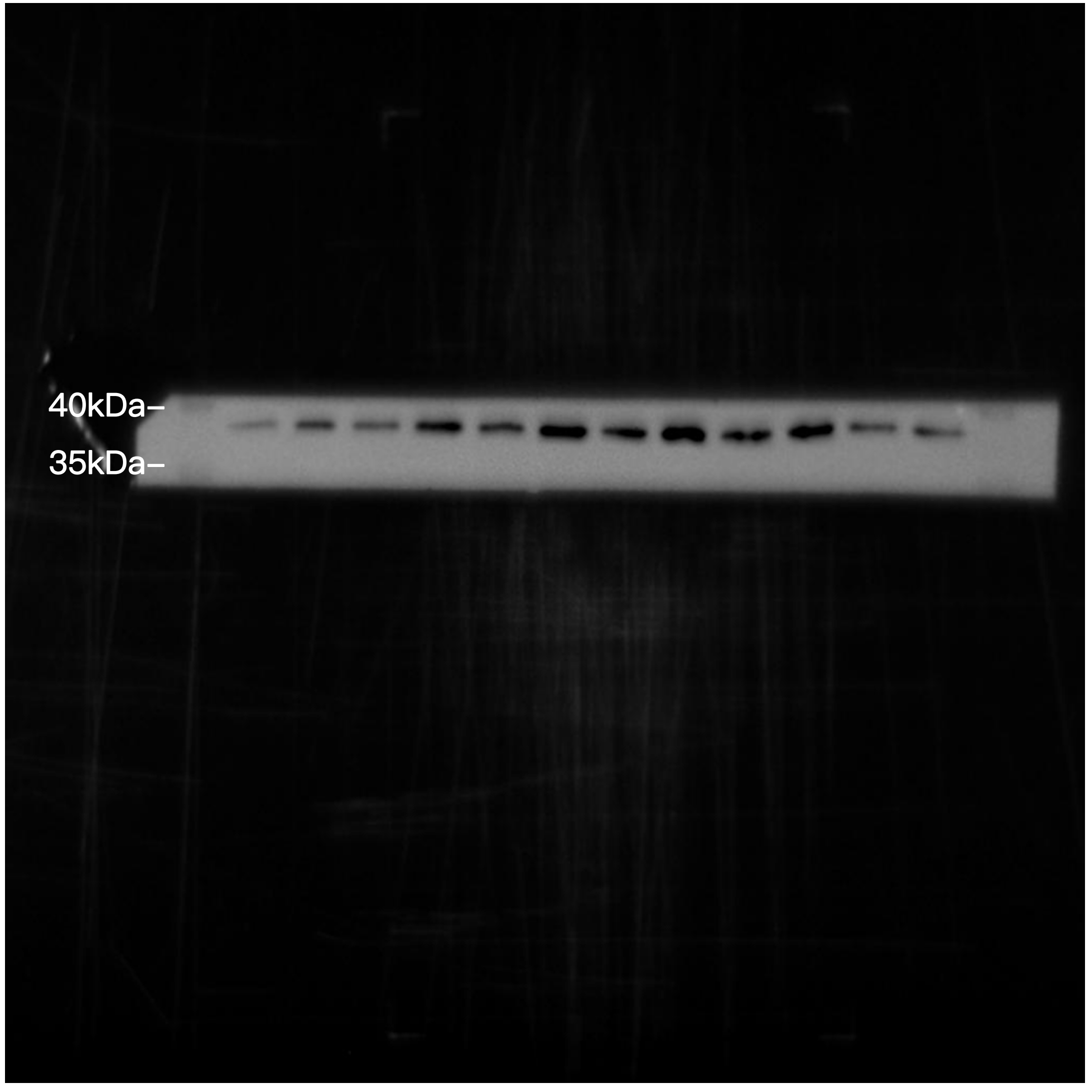

Figure 3J Repeat3

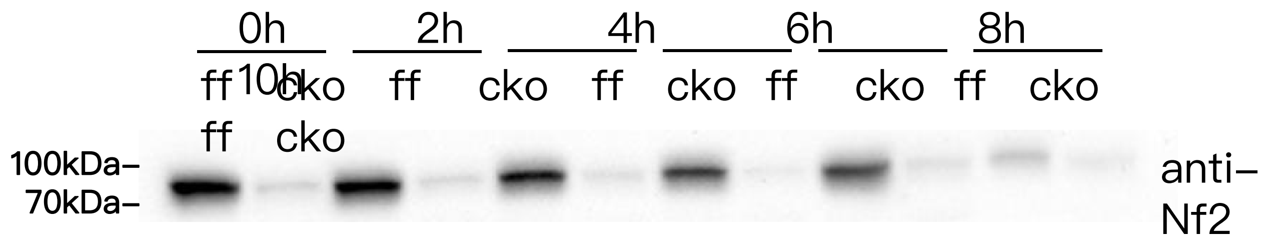

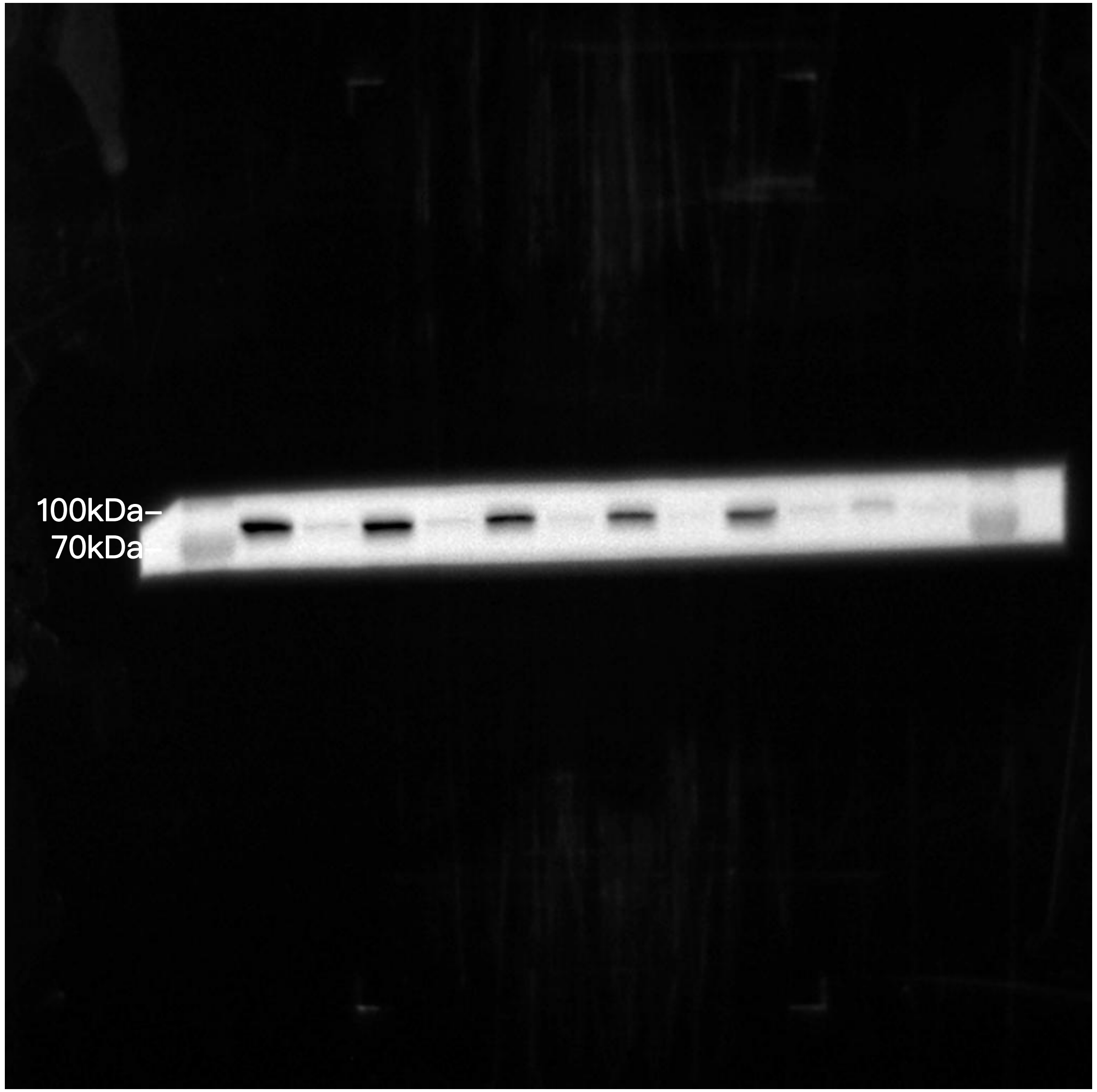

Figure 3J Repeat3

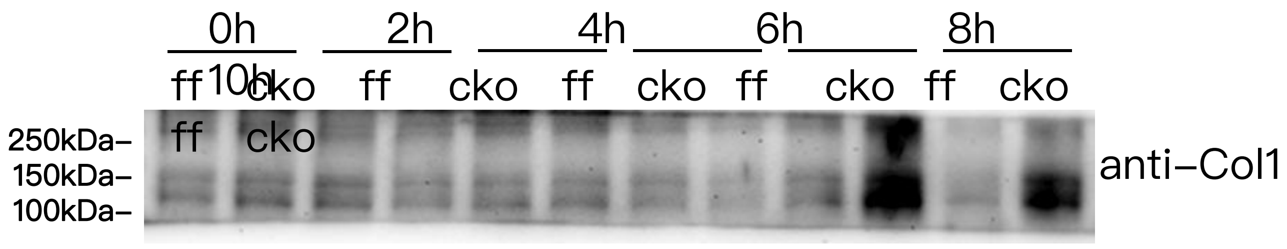

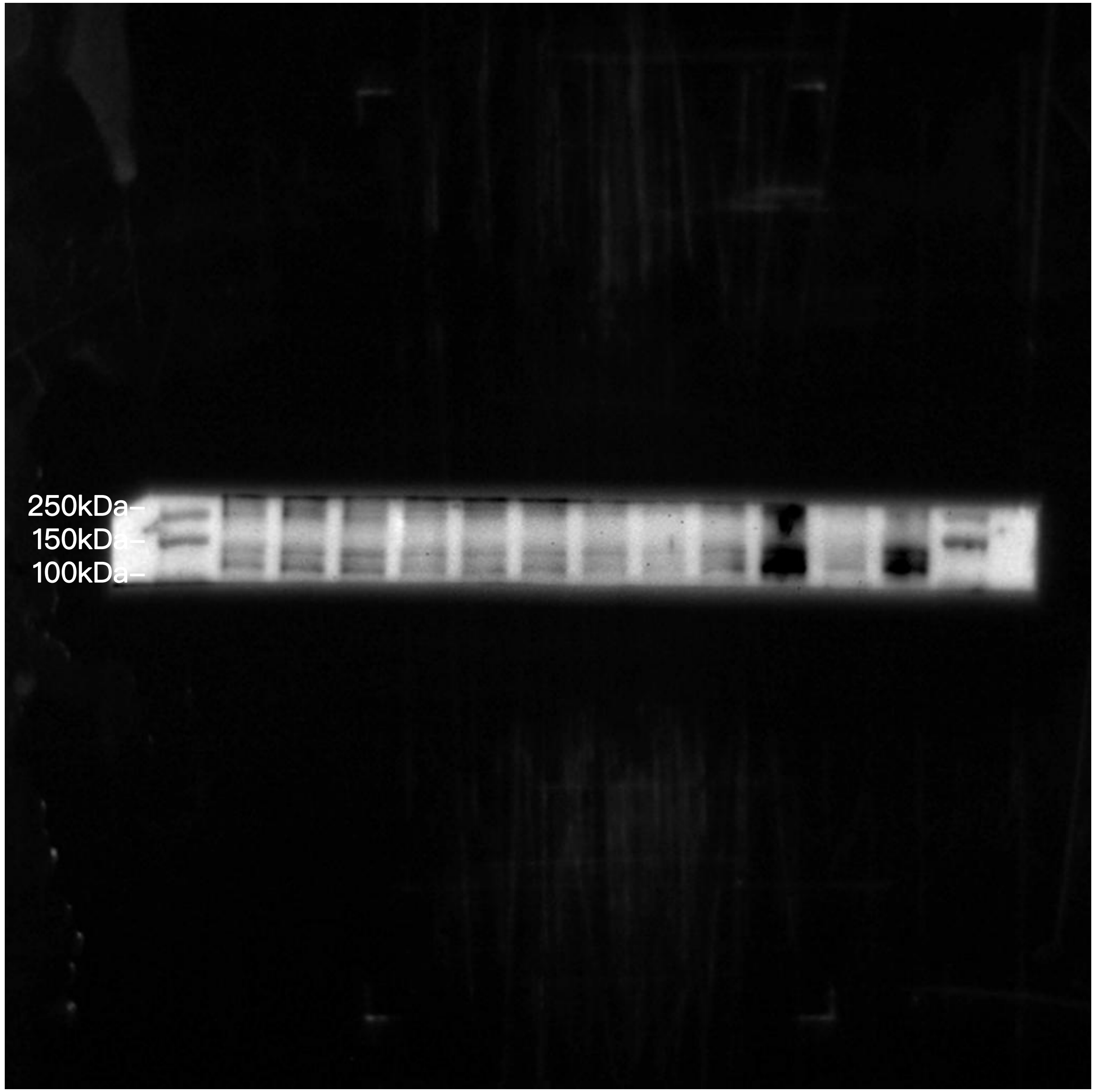

Figure 4A

Figure 4A

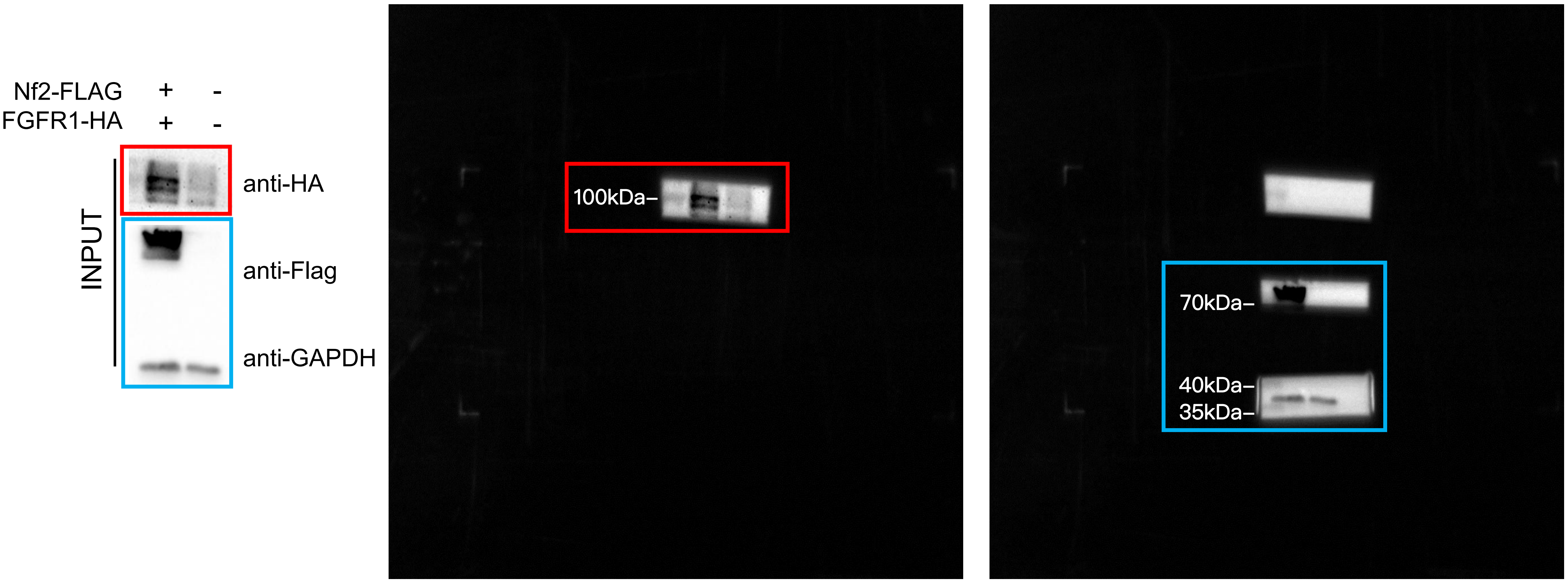
Nf2-FLAG + -
FGFR1-HA + -
INPUT
anti-HA
anti-Flag
anti-GAPDH
100kDa–
70kDa–
40kDa–
35kDa–

Figure 4B

Figure 4B

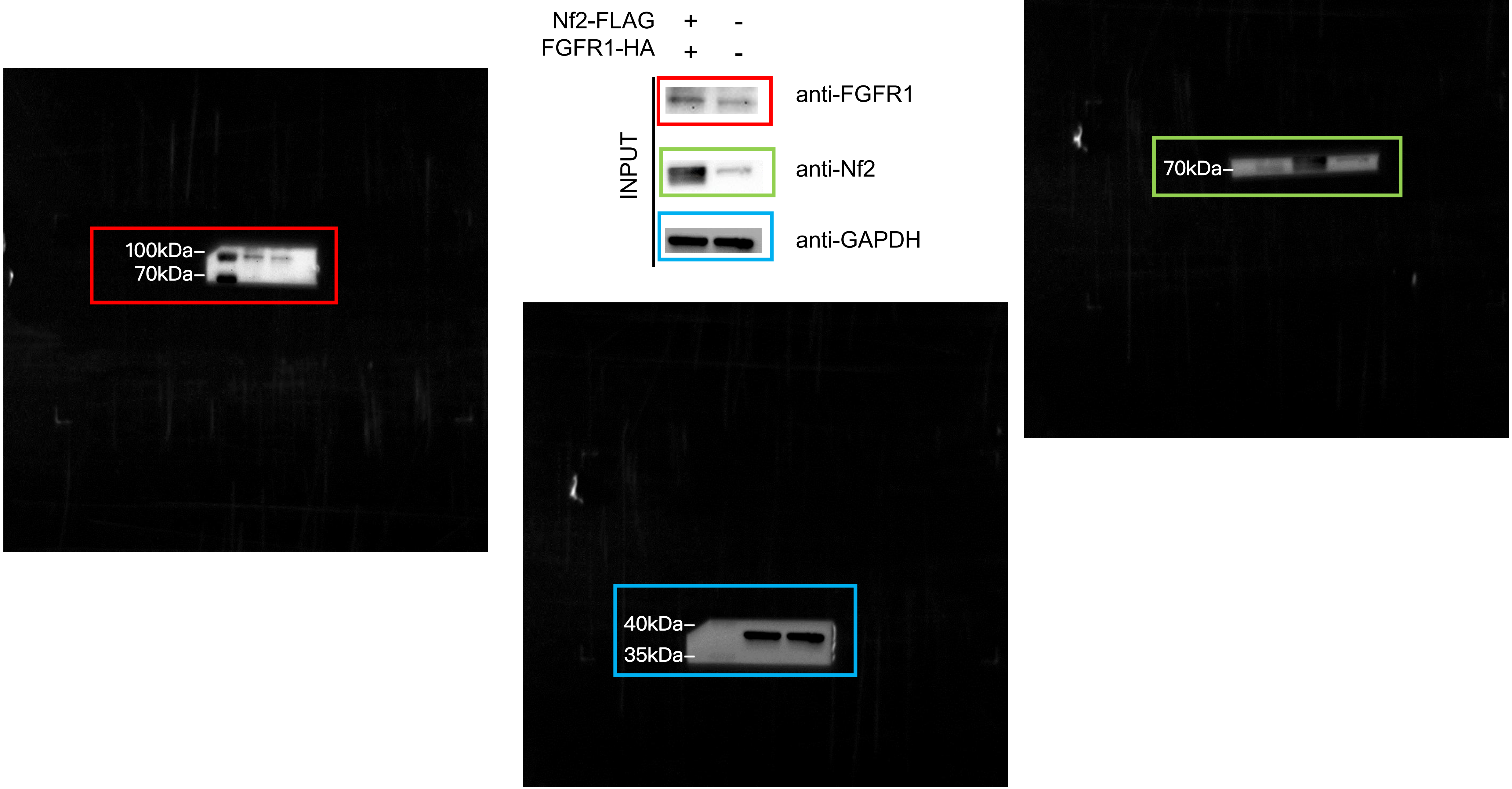

Nf2-FLAG + -
FGFR1-HA + -
INPUT
anti-FGFR1
anti-Nf2
anti-GAPDH
100kDa–
70kDa–
70kDa–
40kDa–
35kDa–

Figure 4C

Figure 4C

show in figure

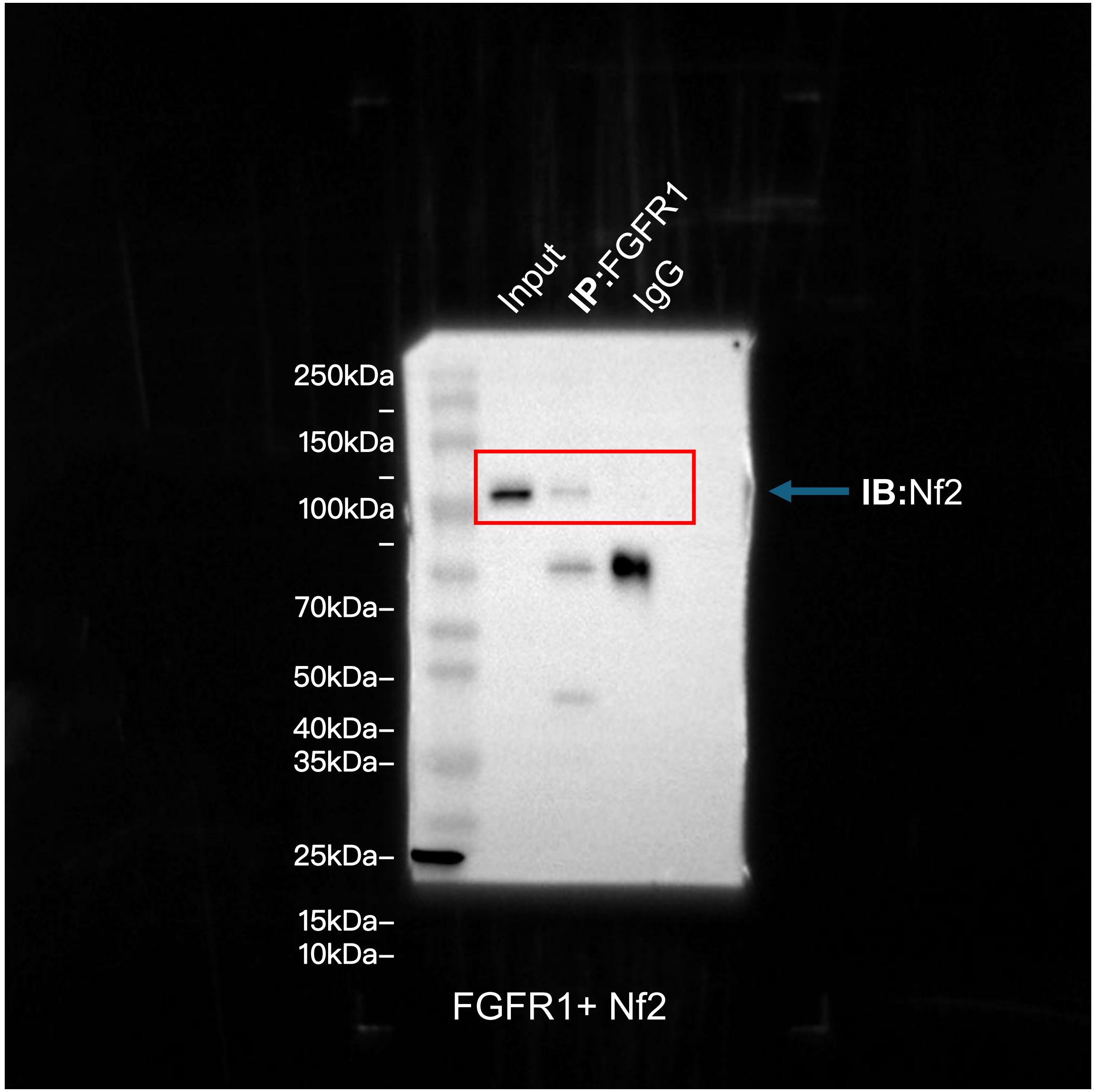

Figure 4C

重新做了coip

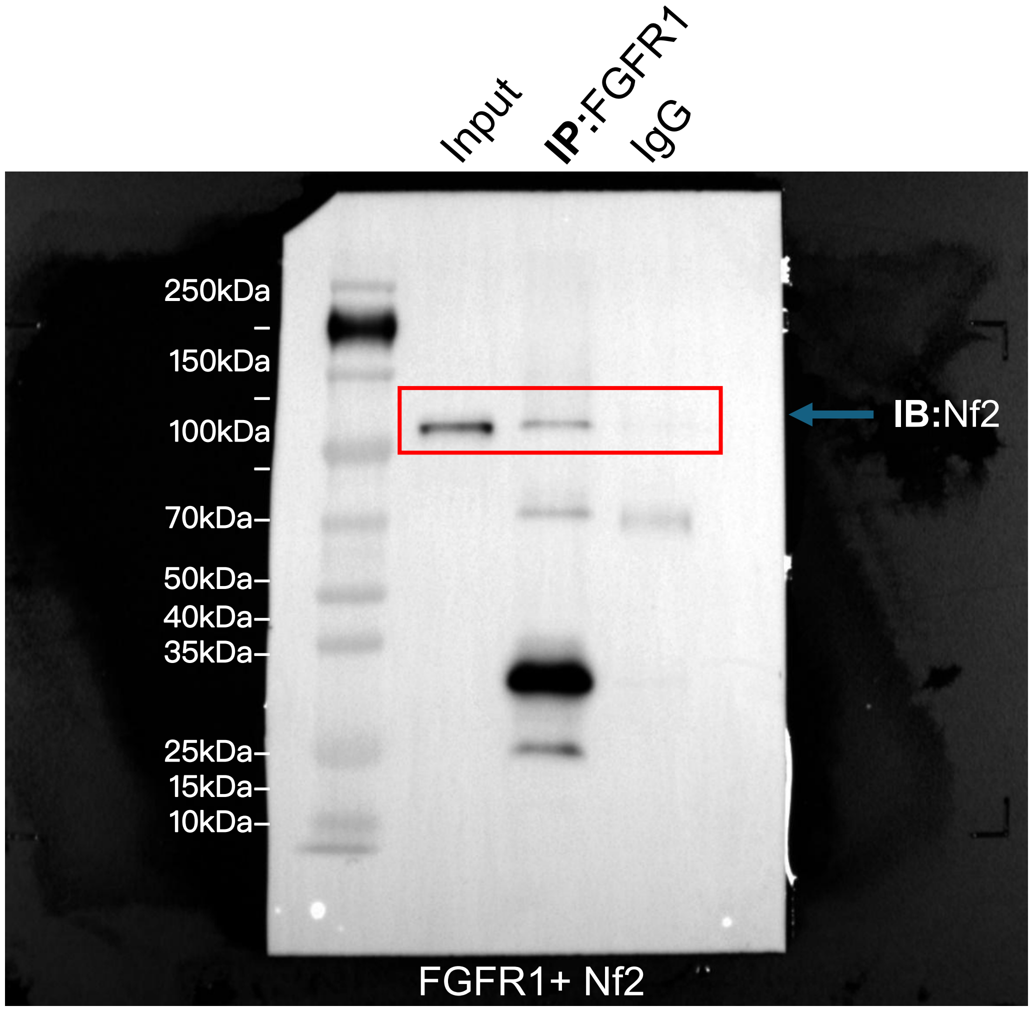

Figure 4D

Figure 4D

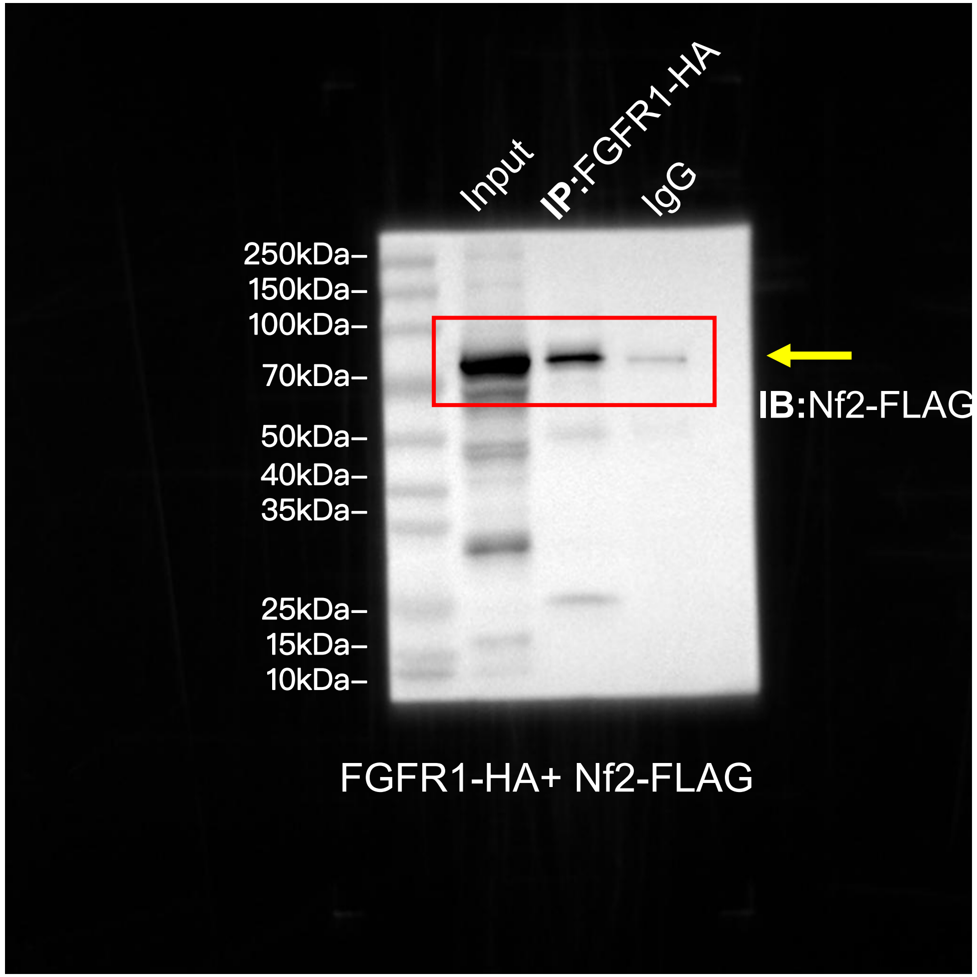
Input
IP:FGFR1-HA
IgG
250kDa–
150kDa–
100kDa–
70kDa–
50kDa–
40kDa–
35kDa–
25kDa–
15kDa–
10kDa–
IB:Nf2-FLAG
FGFR1-HA+ Nf2-FLAG

Figure 4E

Figure 4E

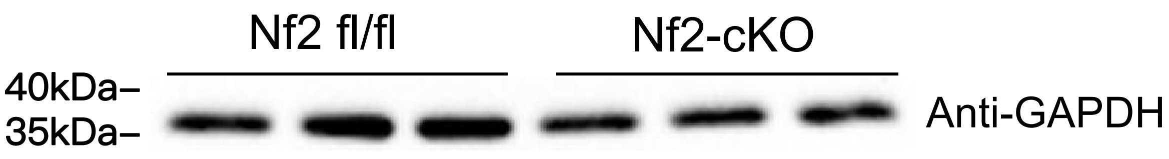

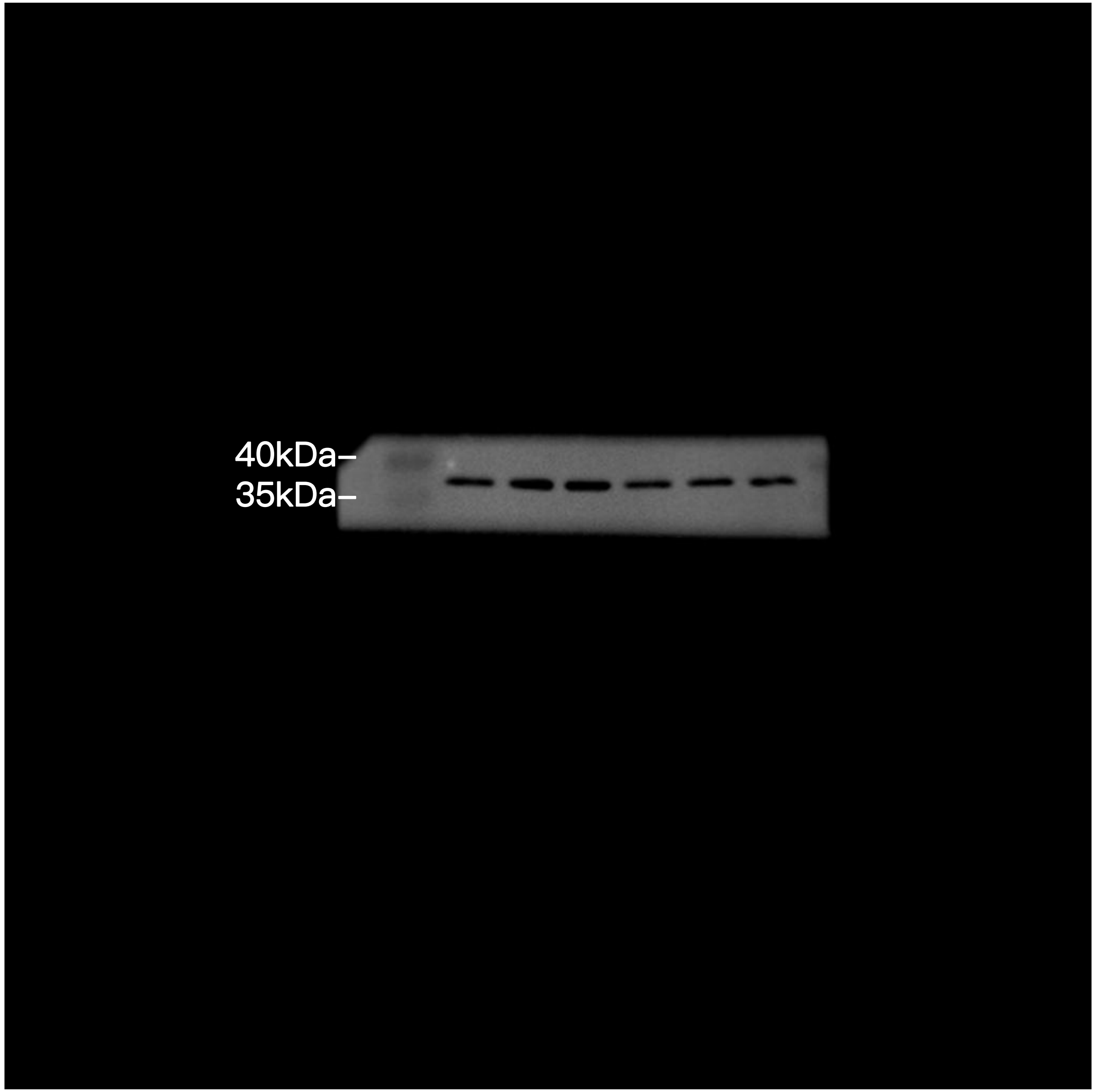

Figure 4E

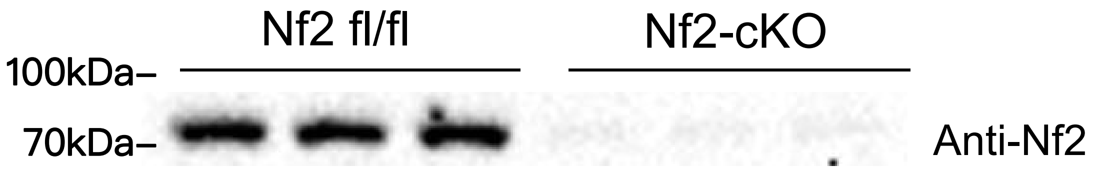

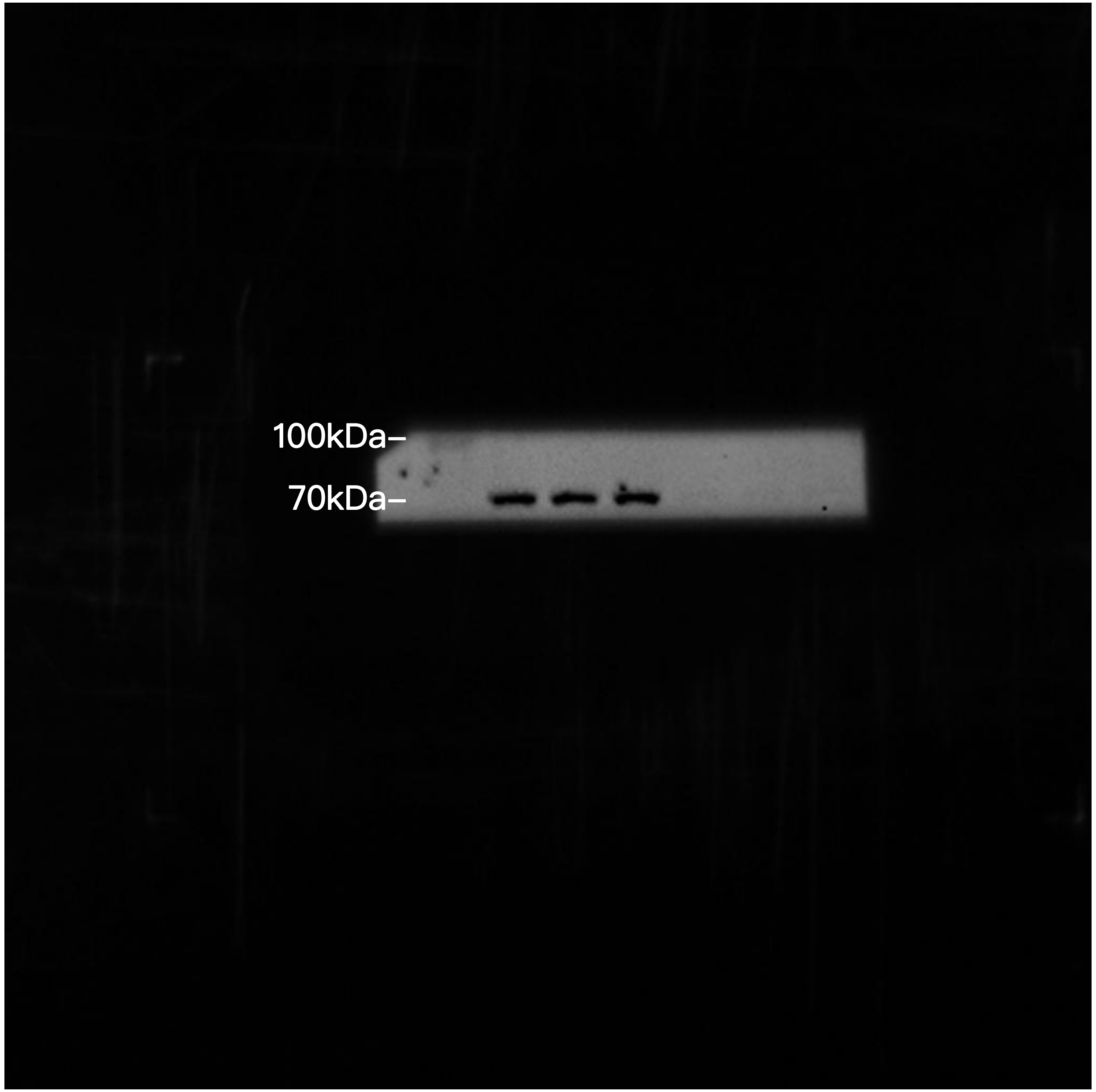

Figure 4E

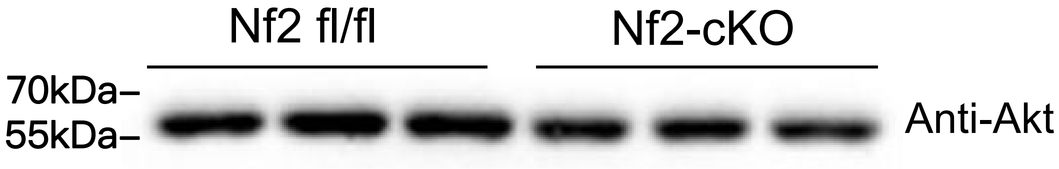

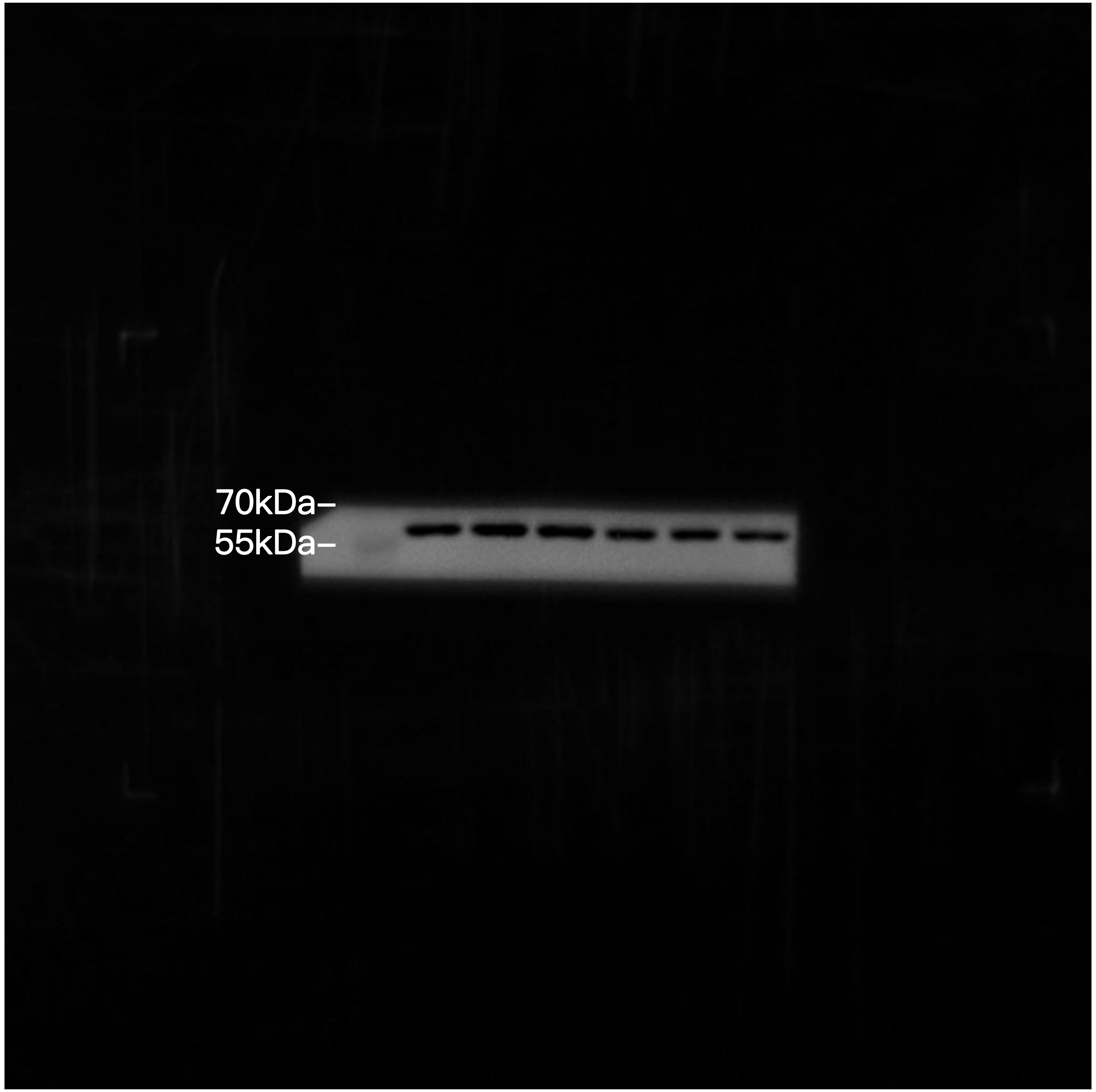

Figure 4E

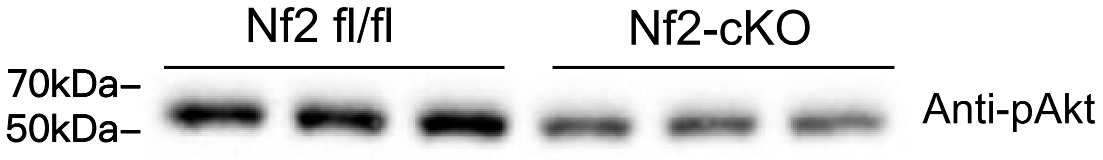

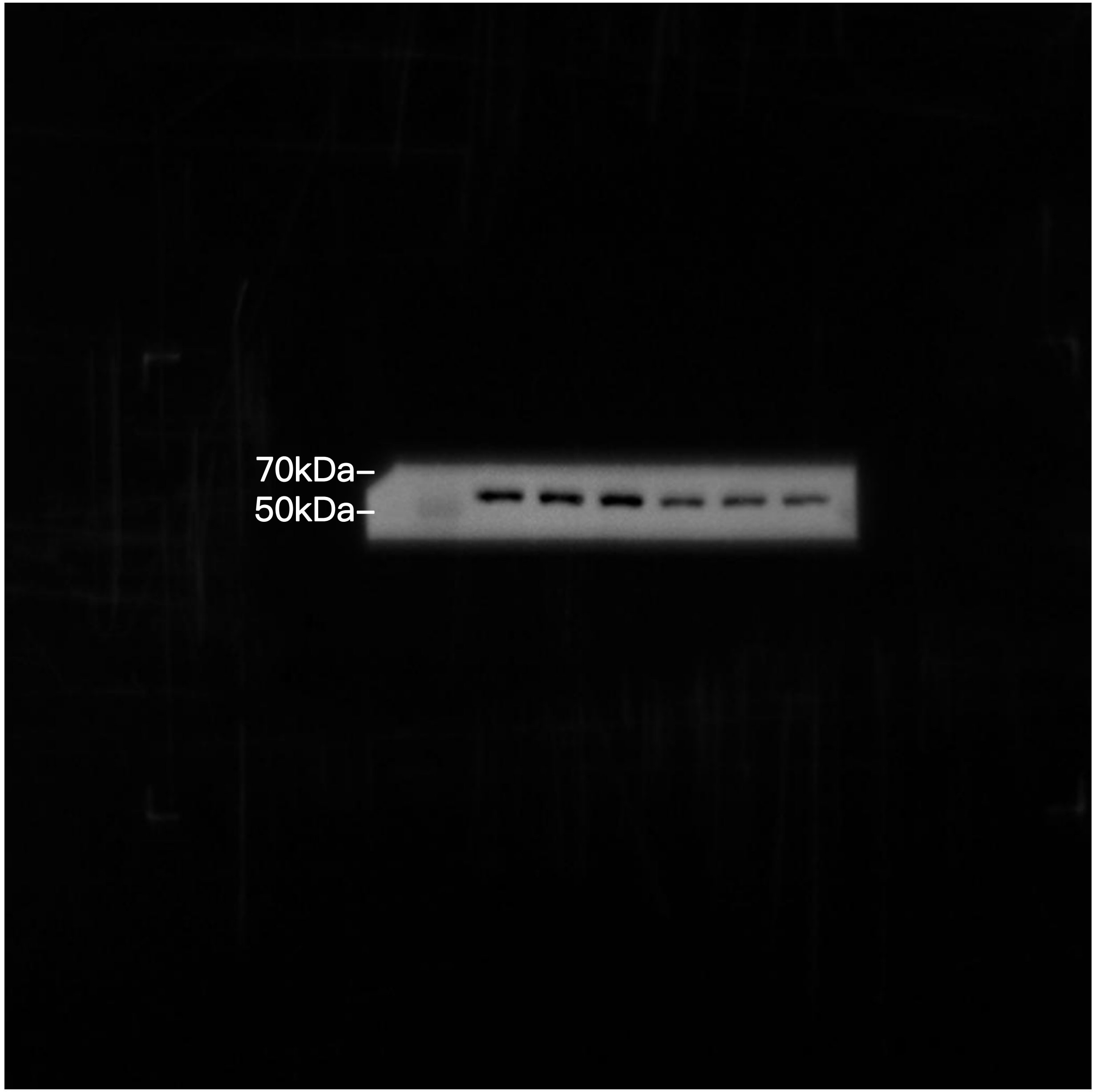

Figure 4E

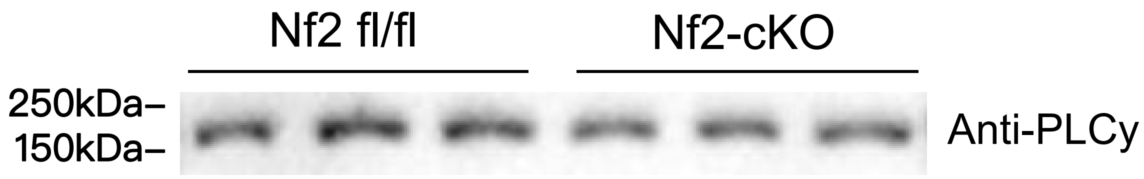

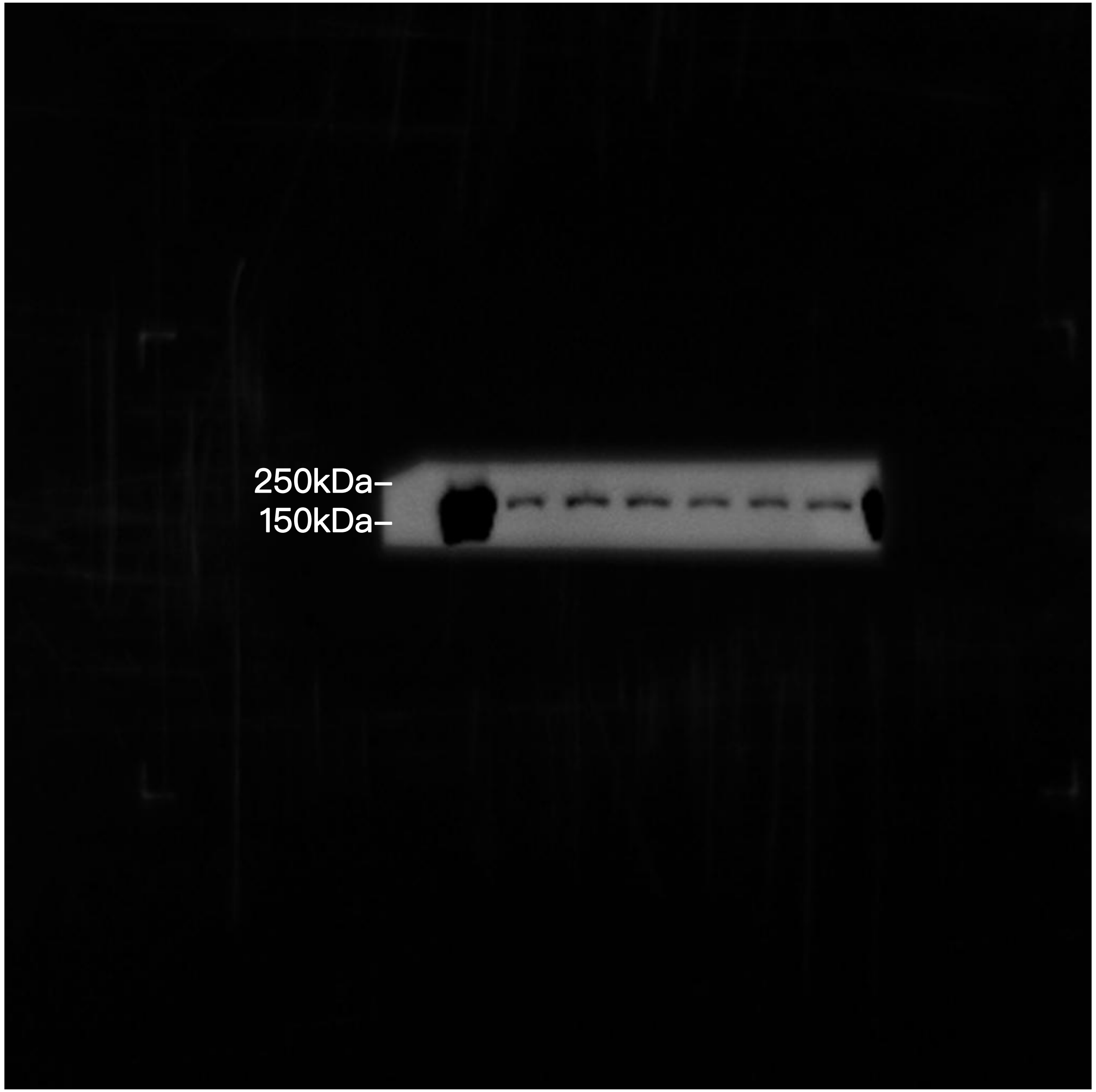

Figure 4E

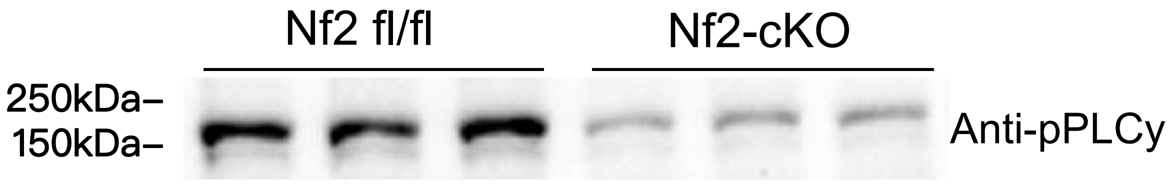

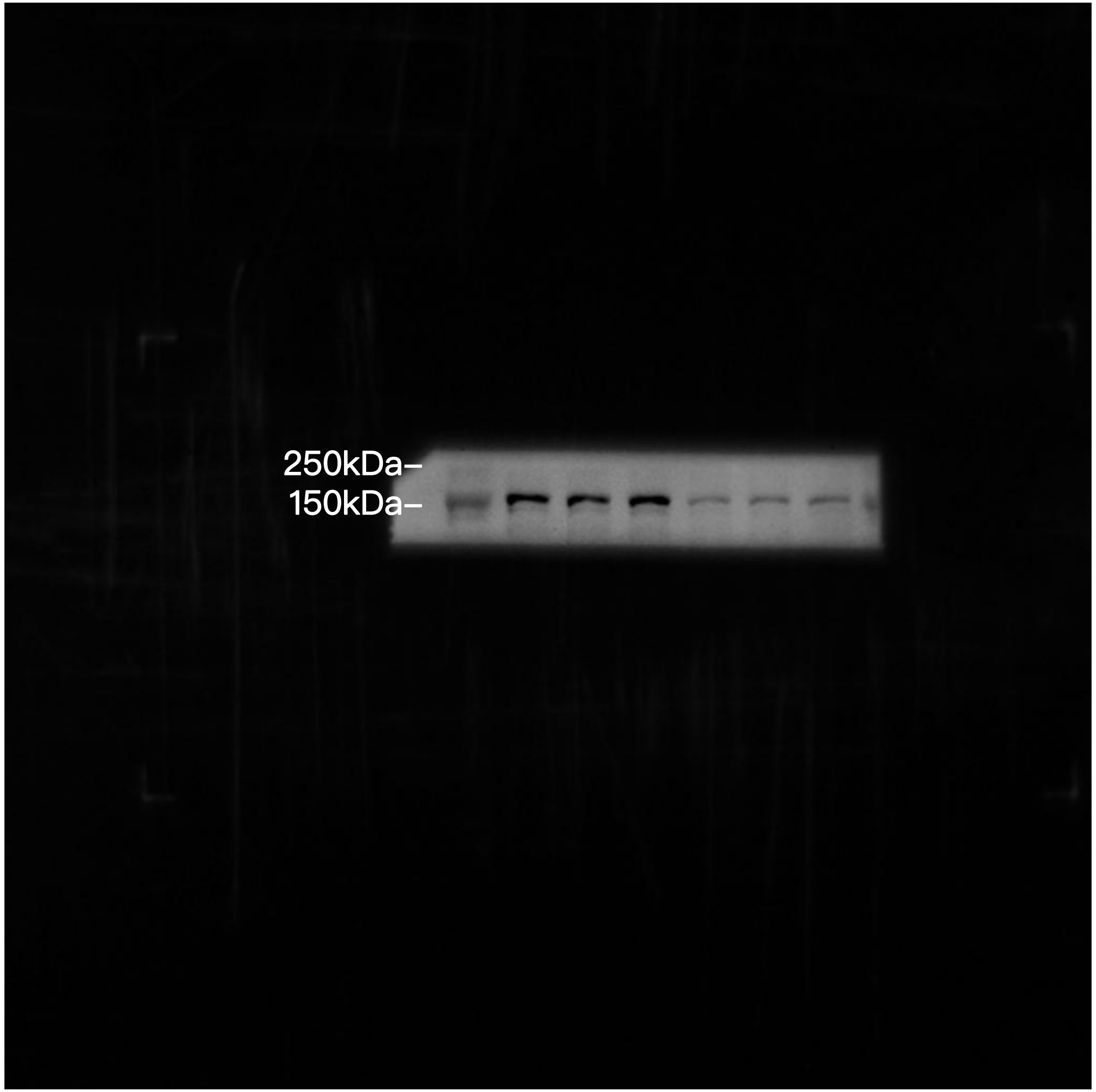

Figure 4E

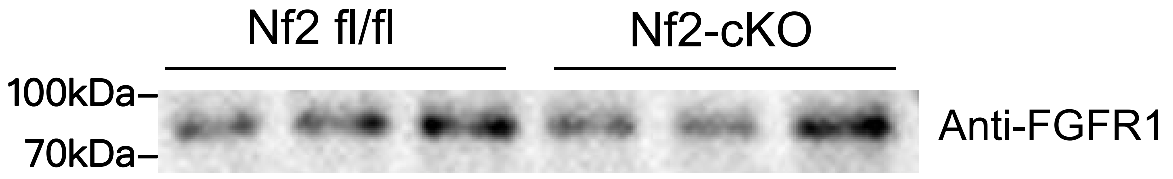

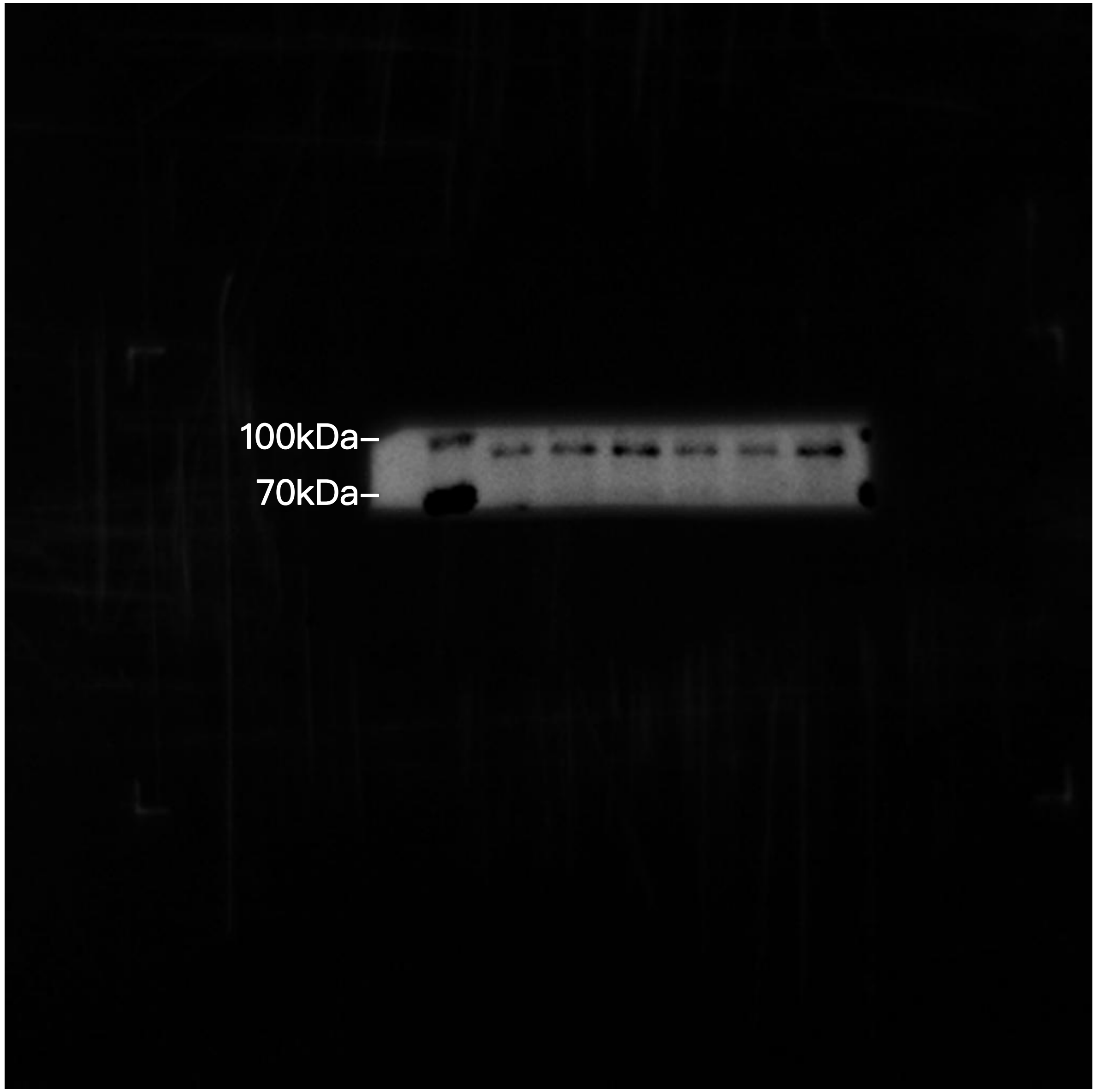

Figure 4H

Figure 4H

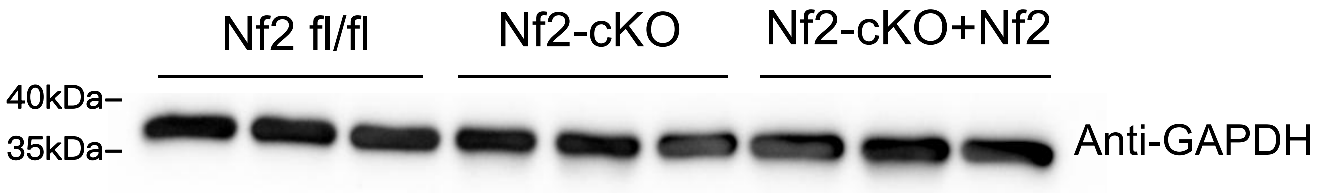
Nf2 fl/fl
Nf2-cKO
Nf2-cKO+Nf2
40kDa–
35kDa–
Anti-GAPDH

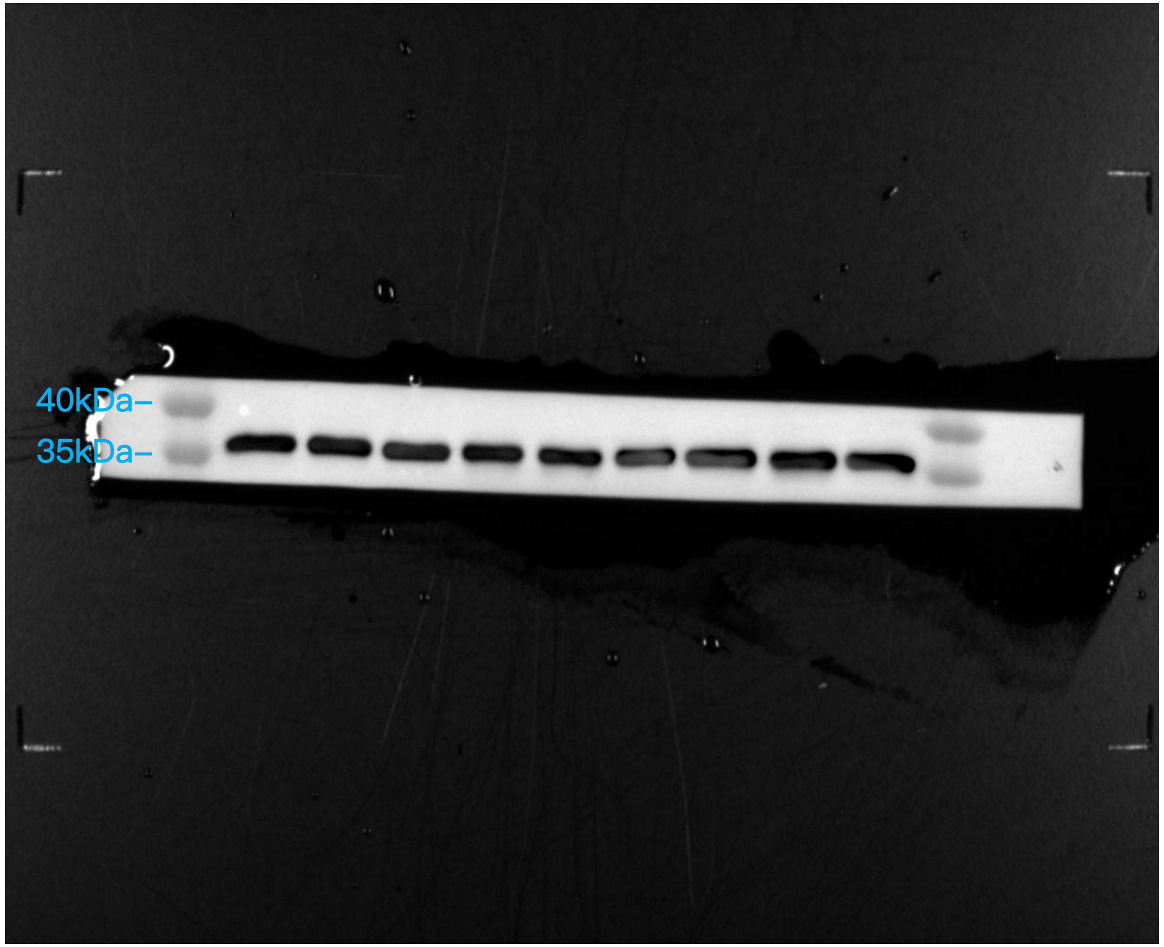
40kDa–
35kDa–

Figure 4H

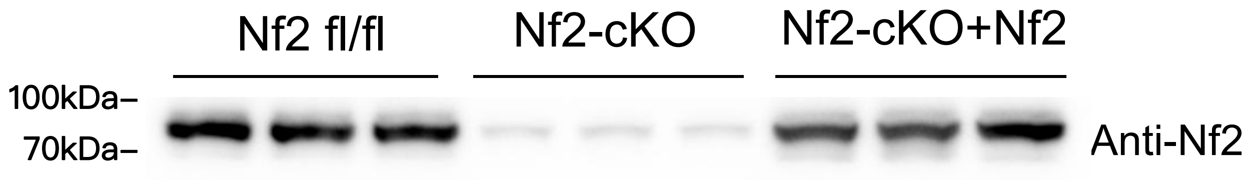
Nf2 fl/fl
Nf2-cKO
Nf2-cKO+Nf2
100kDa–
70kDa–
Anti-Nf2

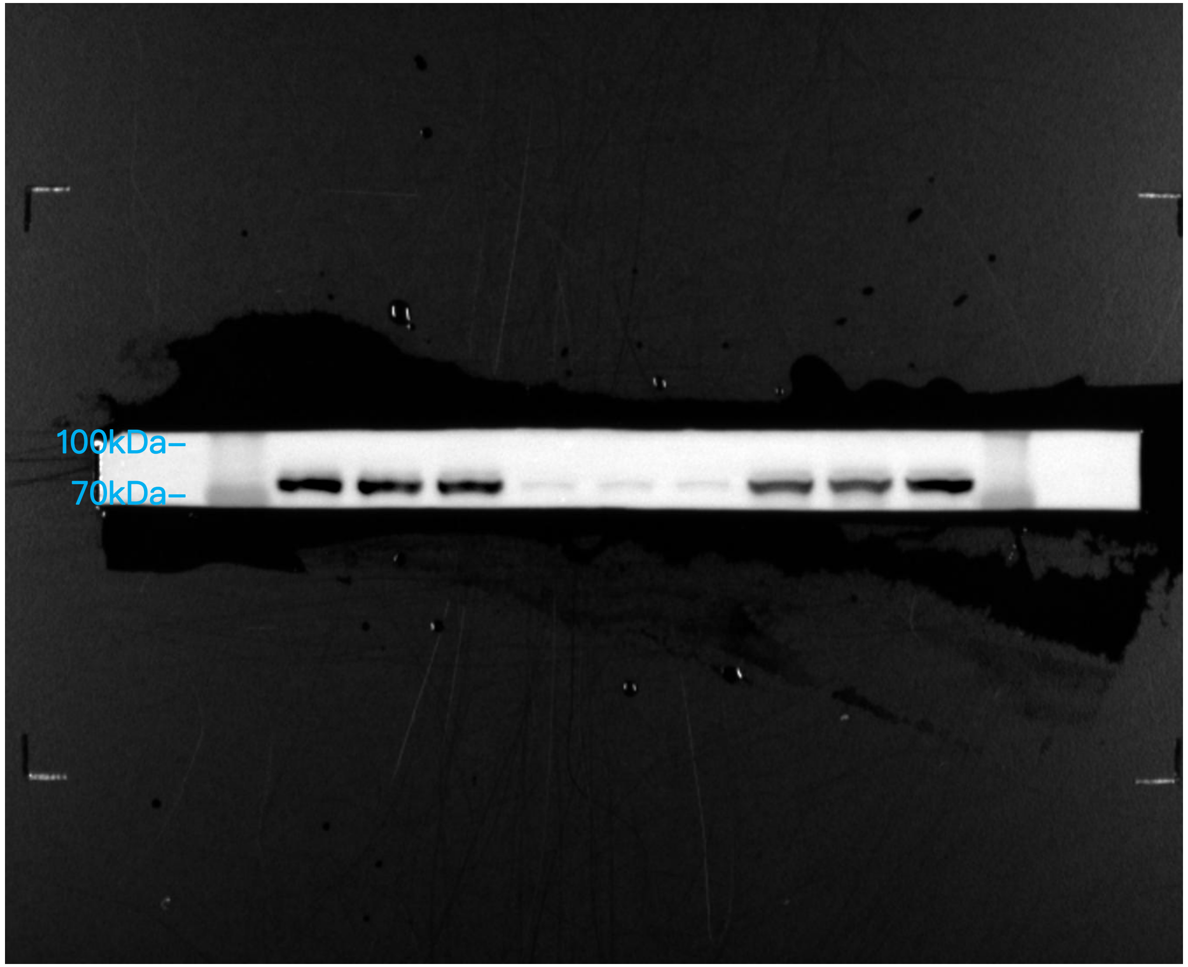
100kDa–
70kDa–

Figure 4H

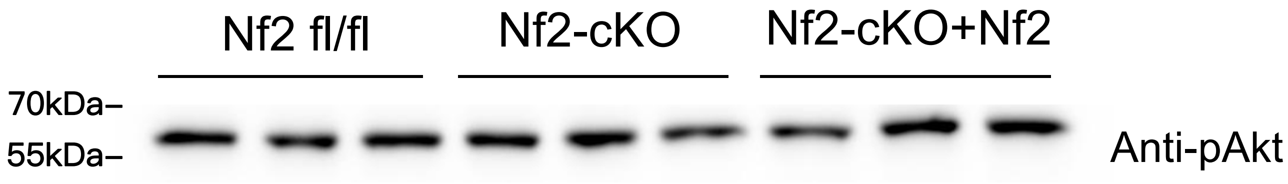
Nf2 fl/fl
Nf2-cKO
Nf2-cKO+Nf2
70kDa–
55kDa–
Anti-pAkt

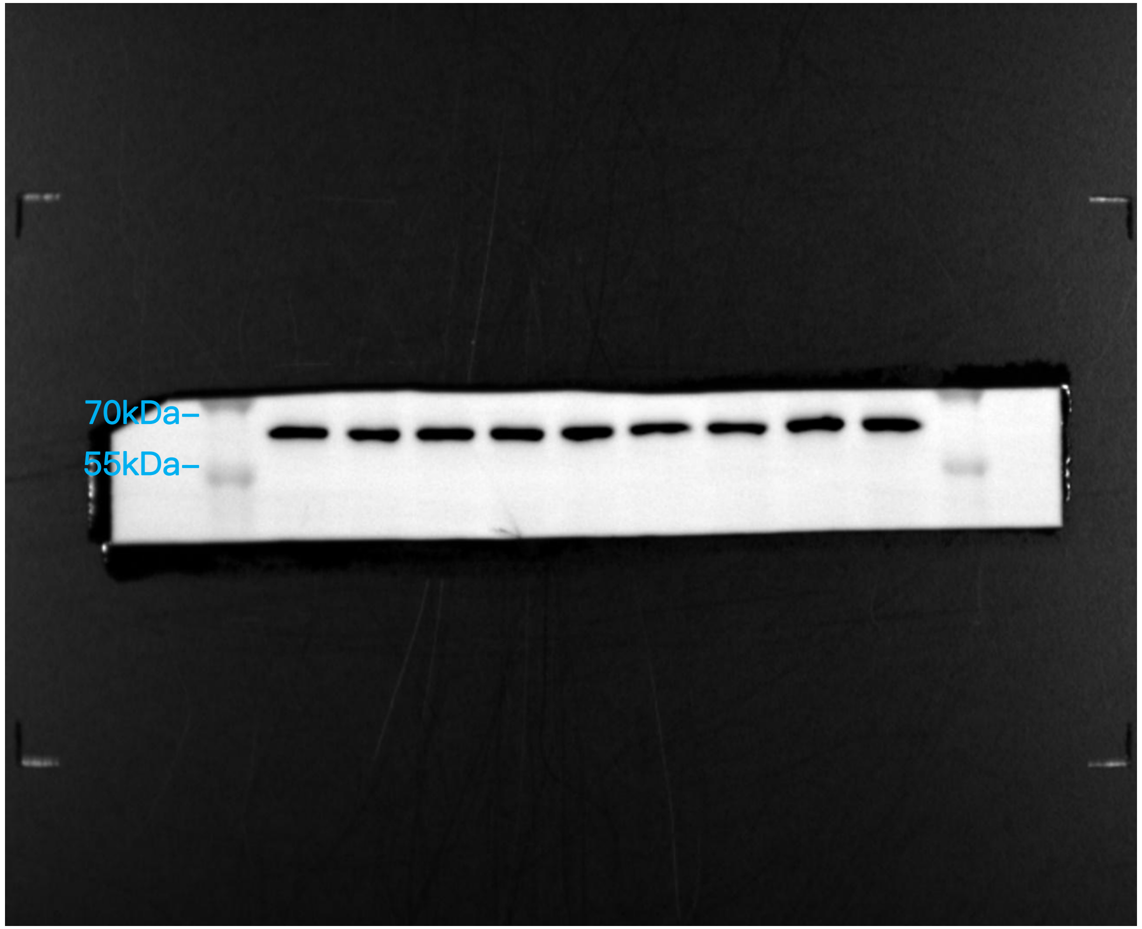
70kDa–
55kDa–

Figure 4H

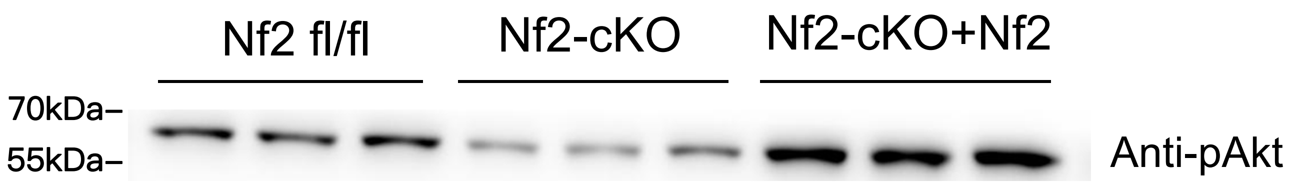
Nf2 fl/fl
Nf2-cKO
Nf2-cKO+Nf2
70kDa–
55kDa–
Anti-pAkt

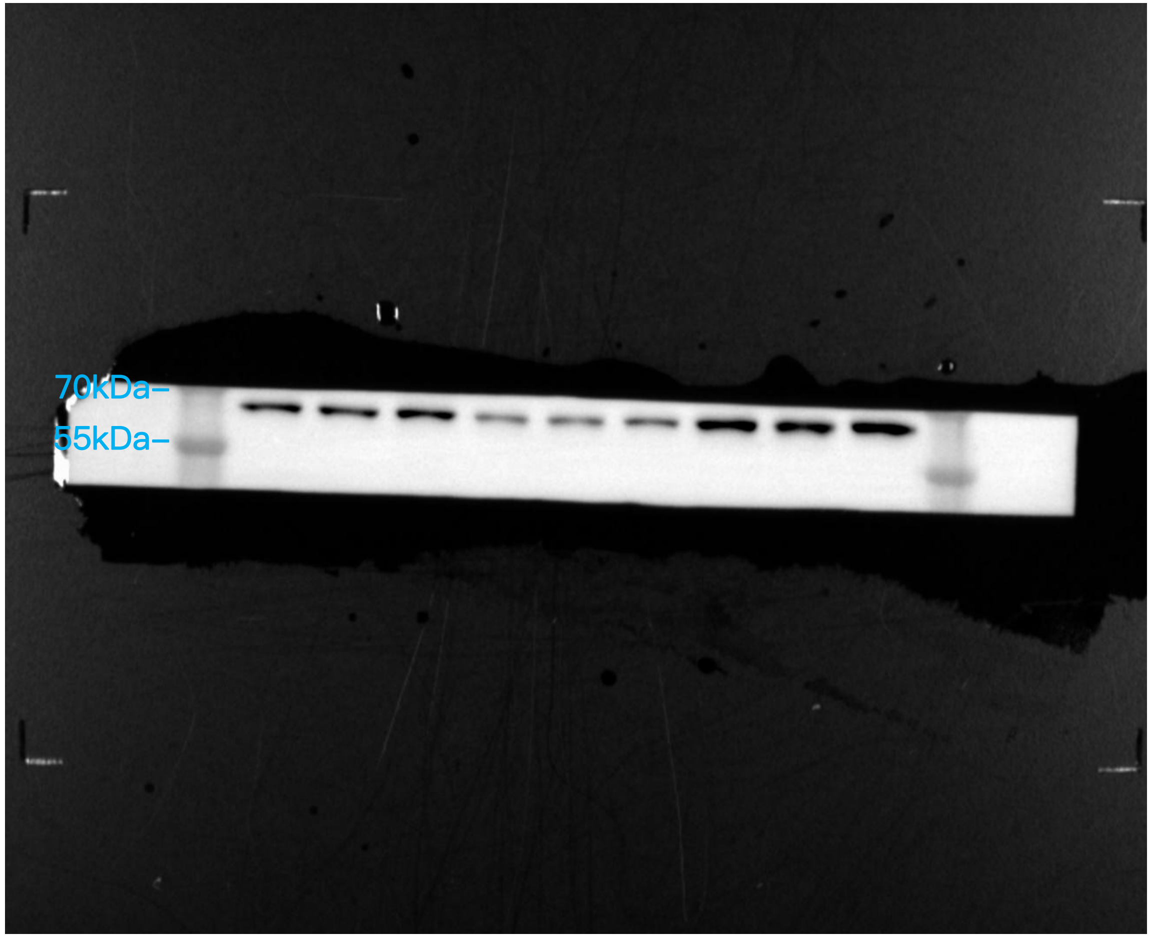
70kDa–
55kDa–

Figure 4H

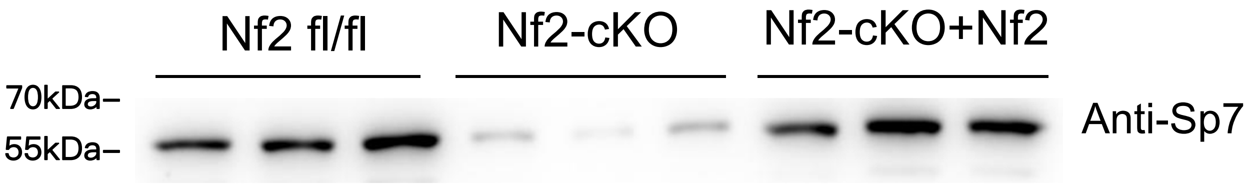

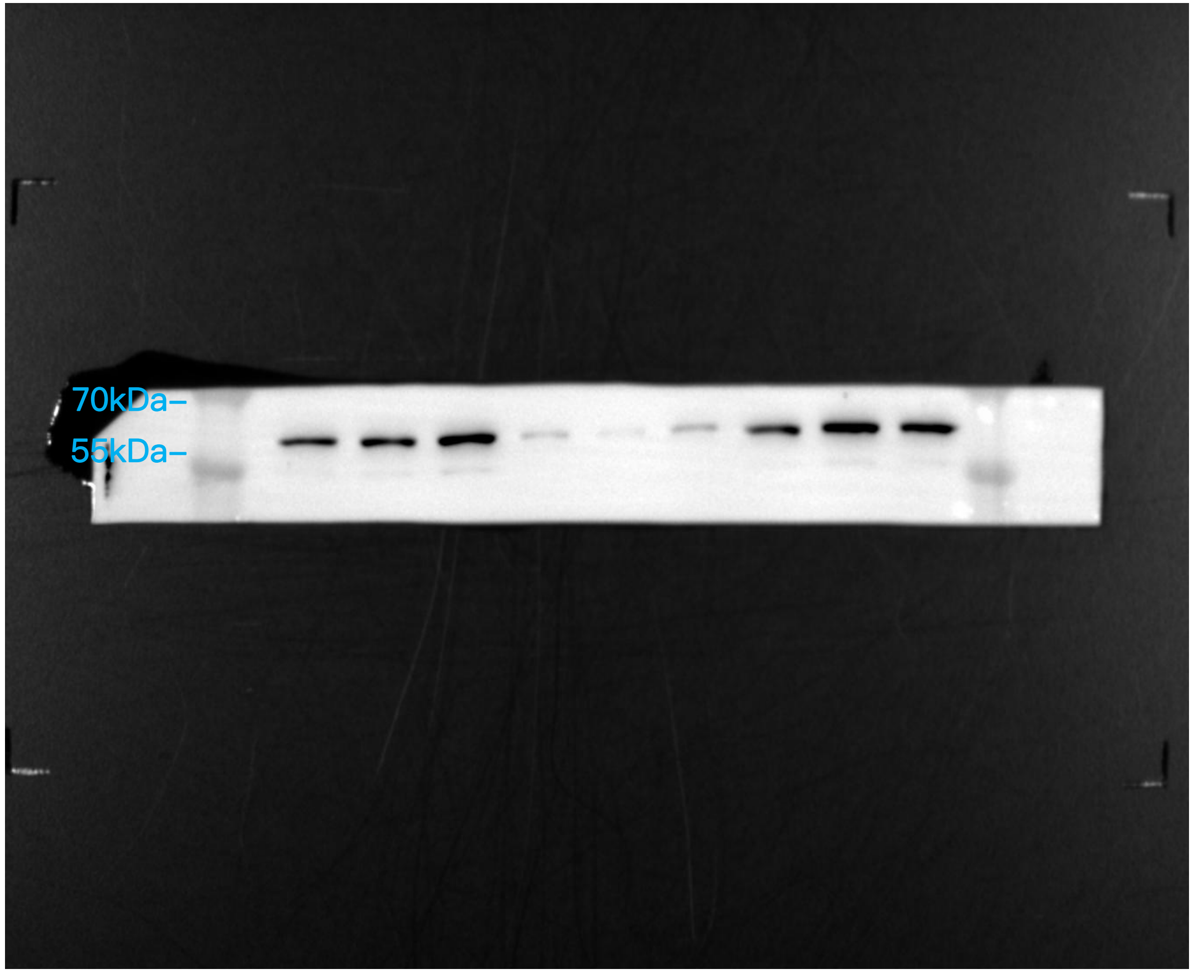

Figure 4H

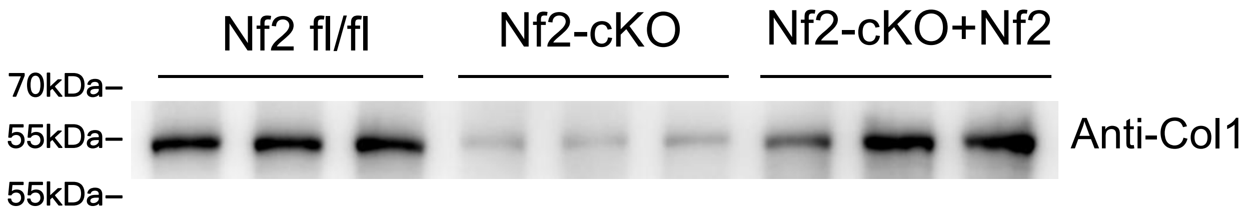
Nf2 fl/fl
Nf2-cKO
Nf2-cKO+Nf2
70kDa–
55kDa–
55kDa–
Anti-Col1

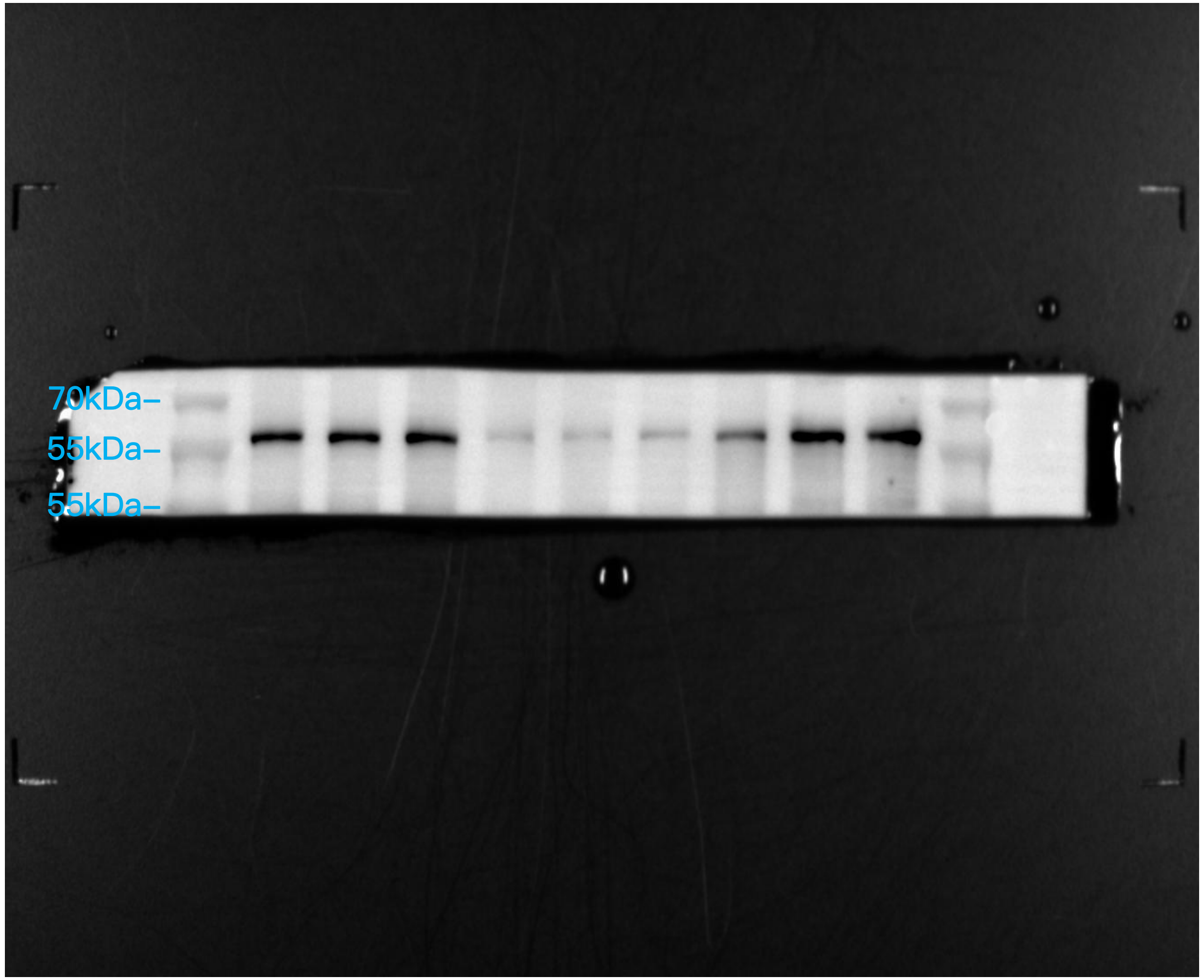
70kDa–
55kDa–
55kDa–

Figure 5A–B

Figure 5A

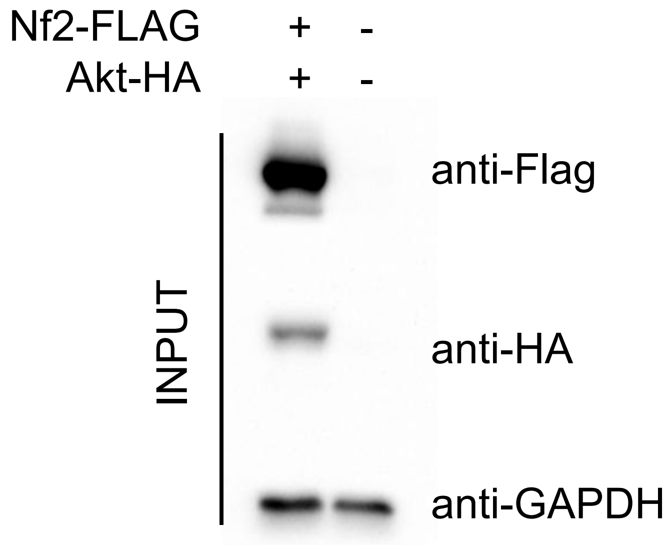

Nf2-FLAG + -
Akt-HA + -
INPUT
anti-Flag
anti-HA
anti-GAPDH

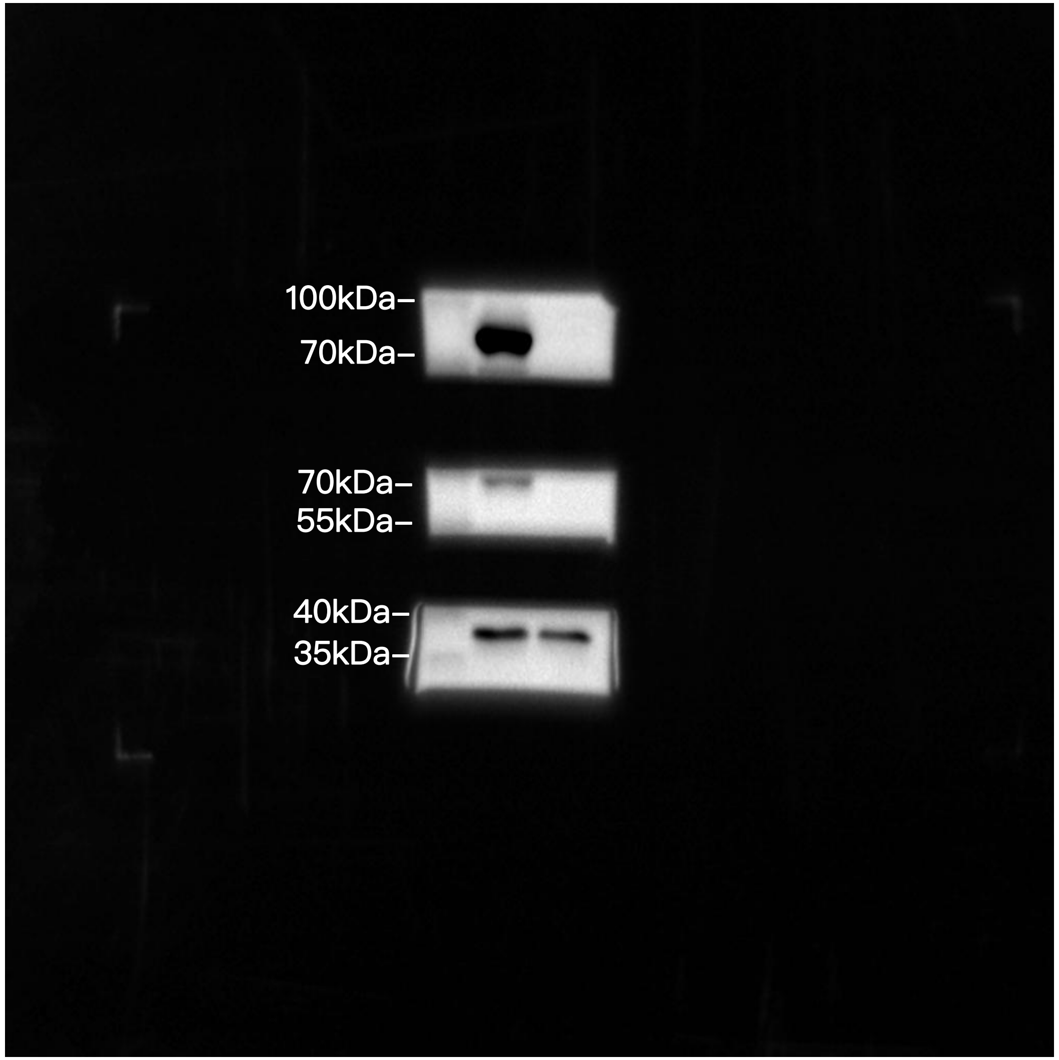

100kDa–
70kDa–
70kDa–
55kDa–
40kDa–
35kDa–

Figure 5B

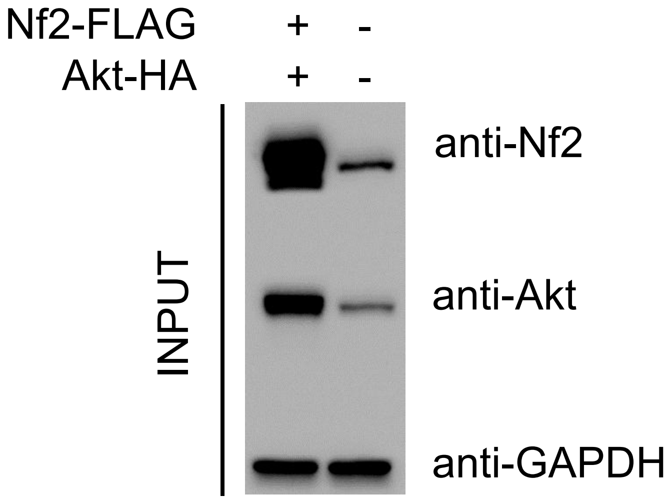
Nf2-FLAG + -
Akt-HA + -
INPUT
anti-Nf2
anti-Akt
anti-GAPDH

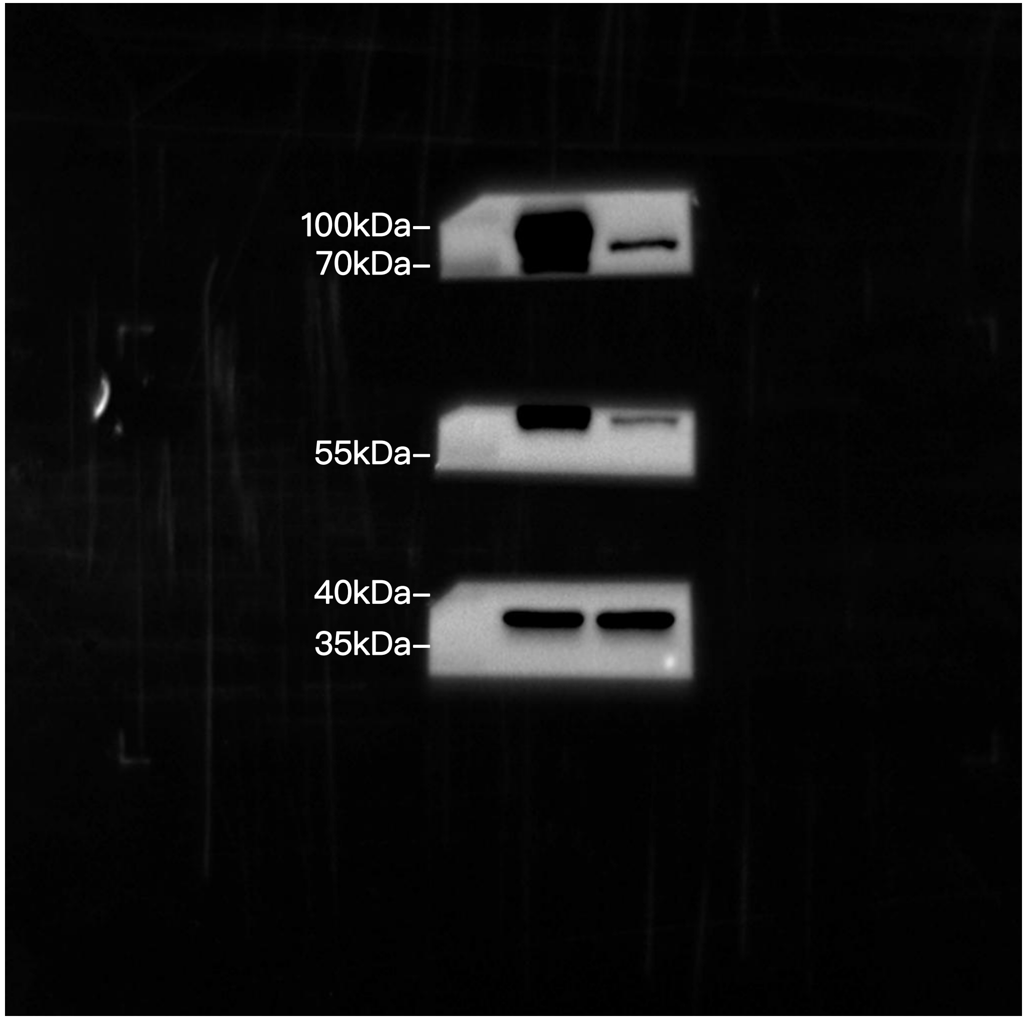
100kDa–
70kDa–
55kDa–
40kDa–
35kDa–

Figure 5C–D

Figure 5C

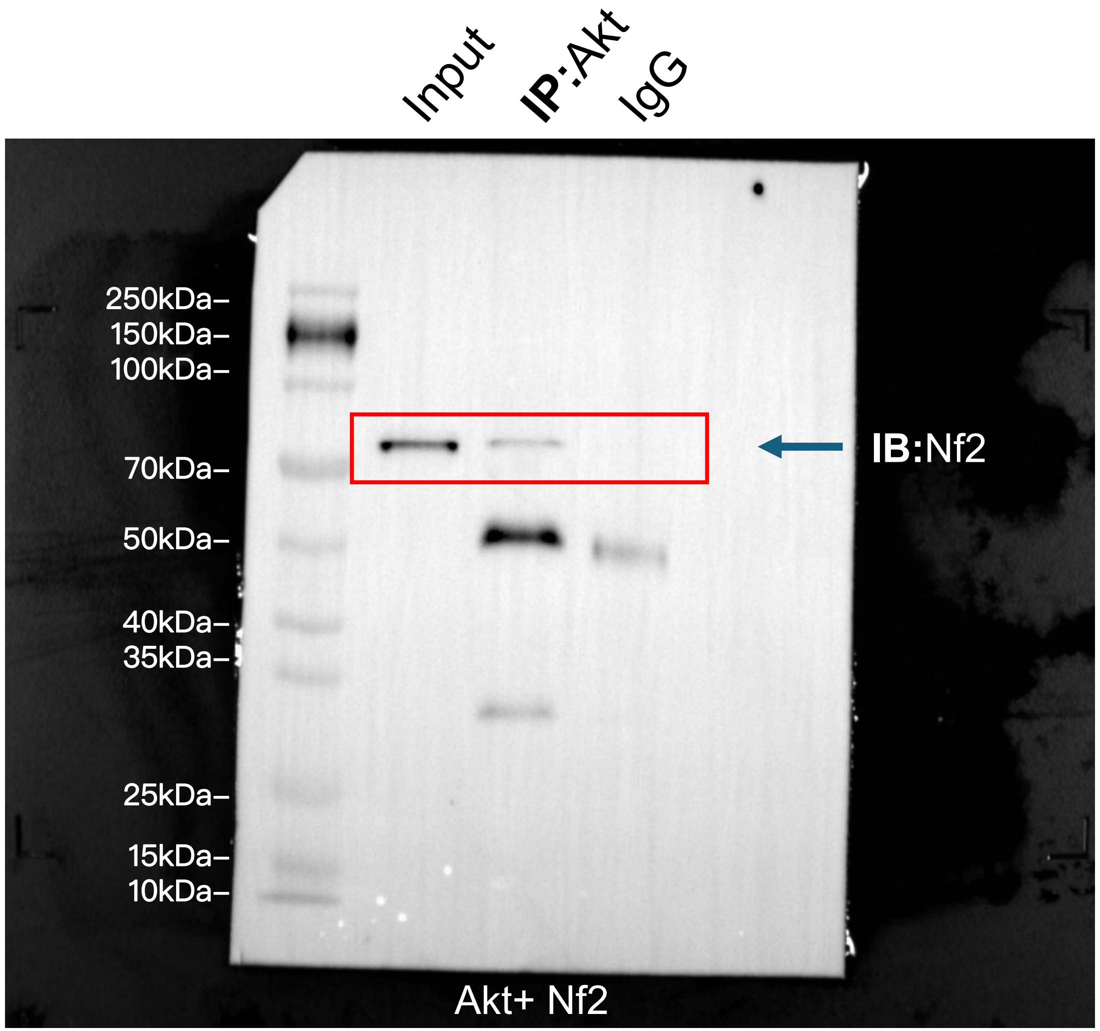
Input
IP:Akt
IgG
250kDa–
150kDa–
100kDa–
70kDa–
50kDa–
40kDa–
35kDa–
25kDa–
15kDa–
10kDa–
IB:Nf2
Akt+ Nf2

Figure 5D

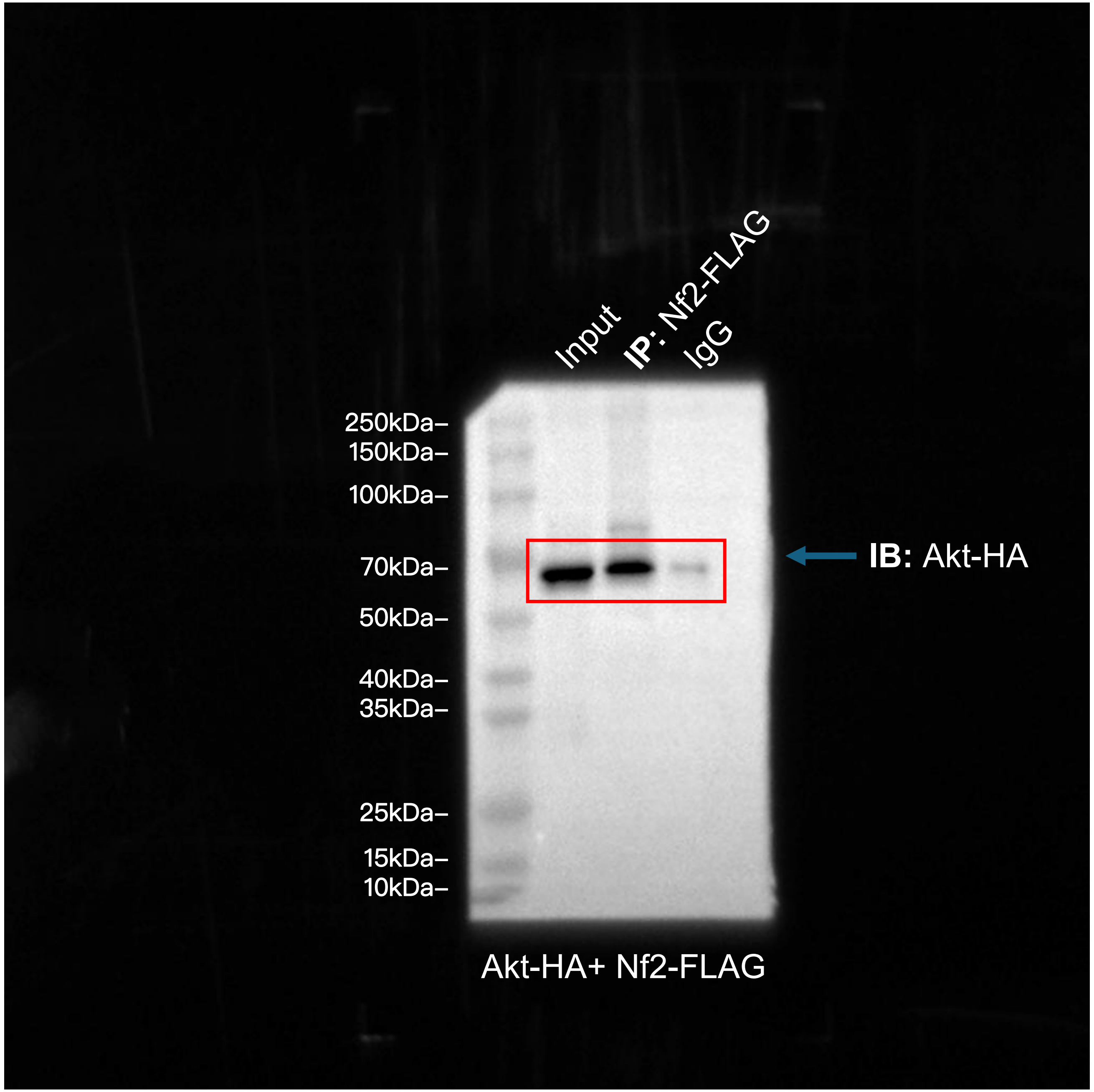

Input
IP: Nf2-FLAG
IgG
250kDa–
150kDa–
100kDa–
70kDa–
50kDa–
40kDa–
35kDa–
25kDa–
15kDa–
10kDa–
IB: Akt-HA
Akt-HA+ Nf2-FLAG

Figure 5E

Figure 5E

show in figure

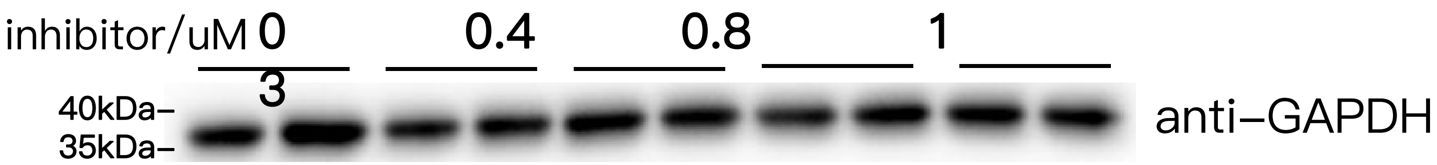

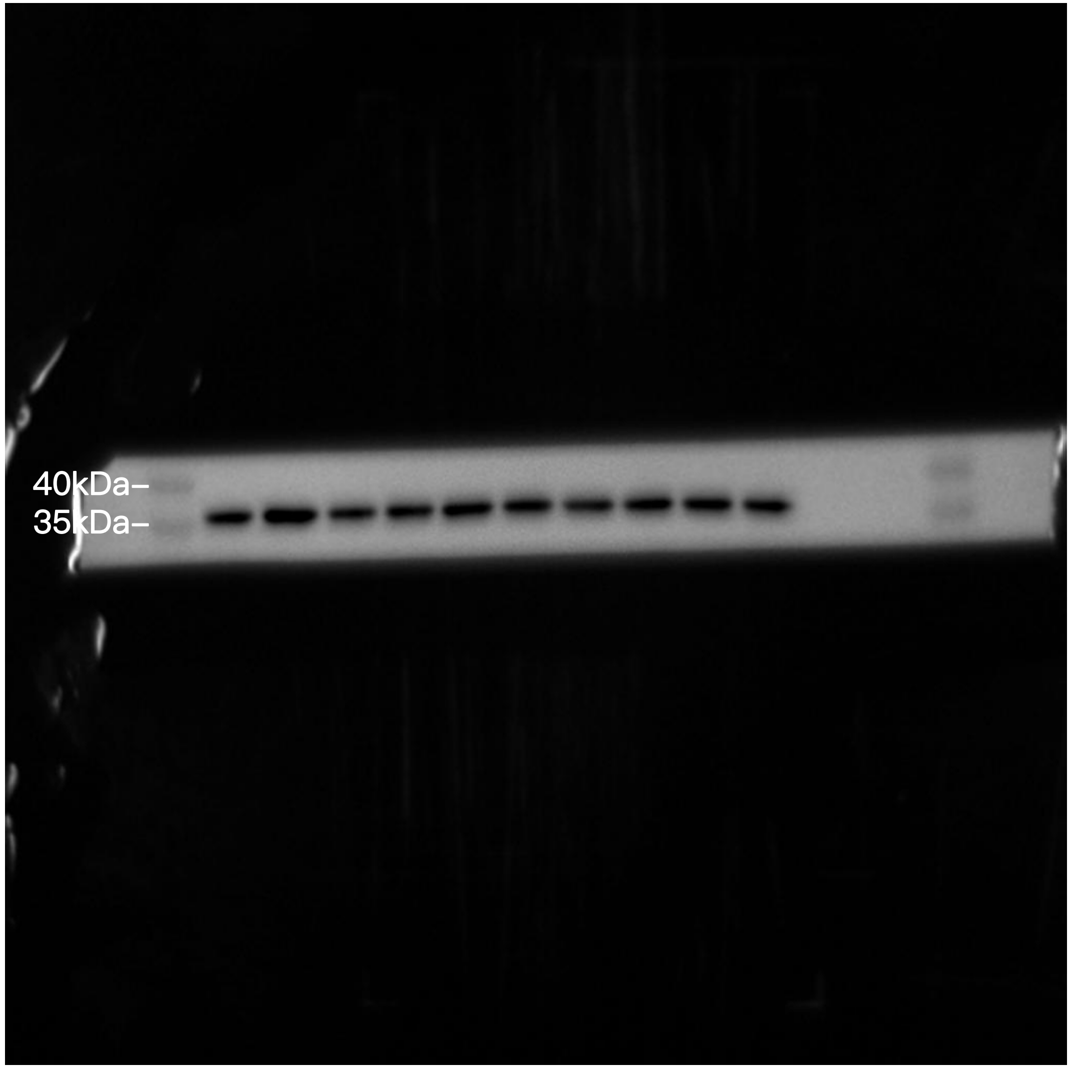

Figure 5E

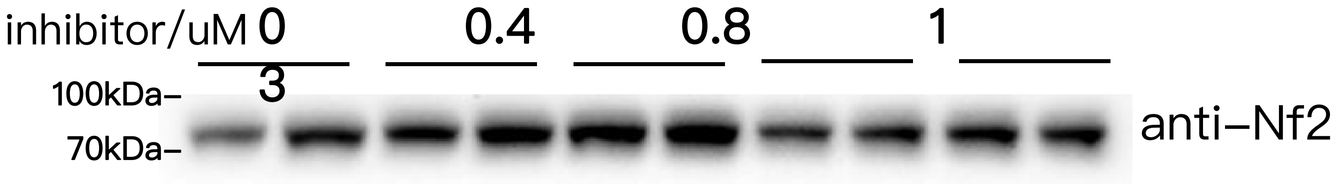

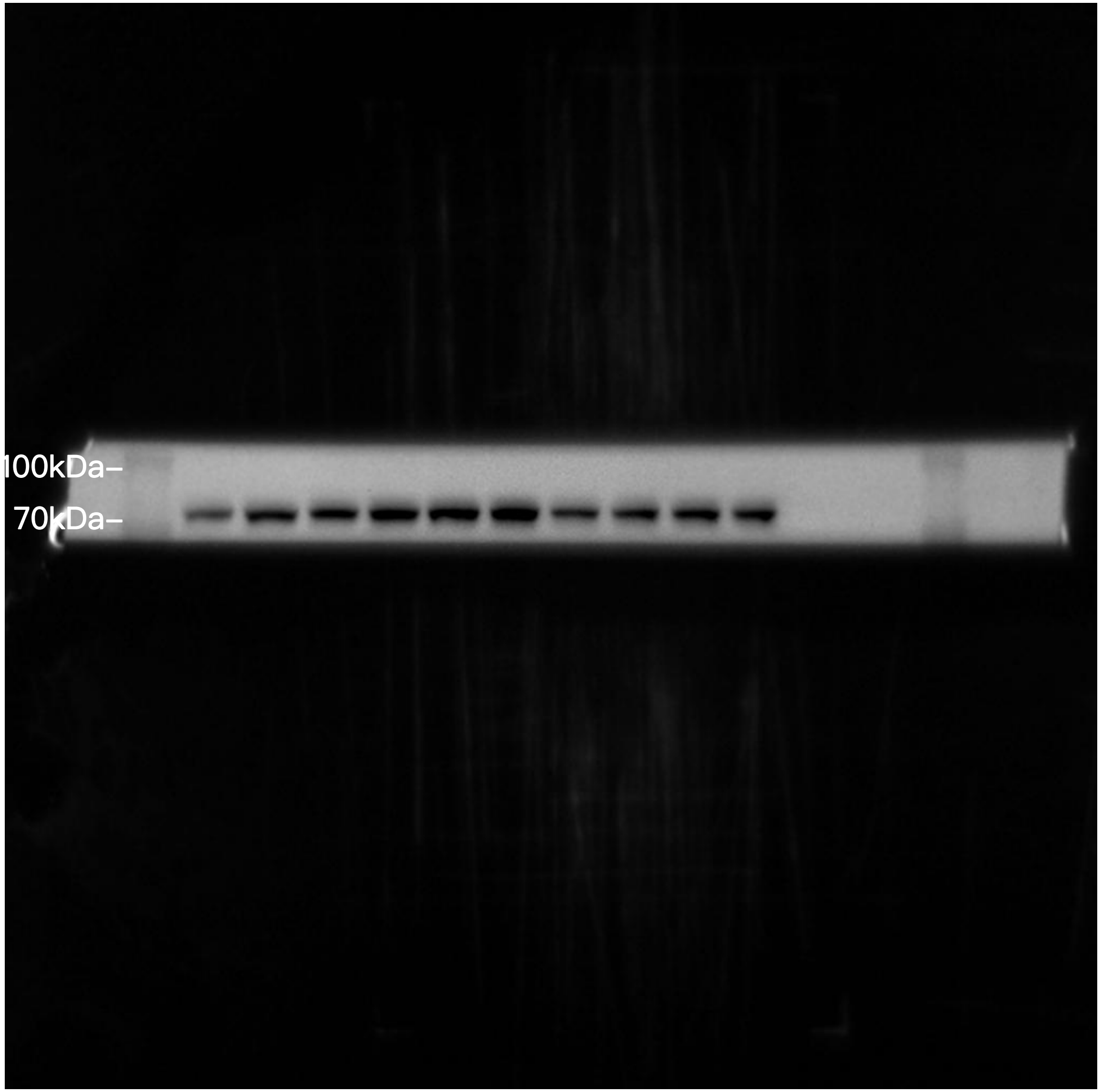

Figure 5E

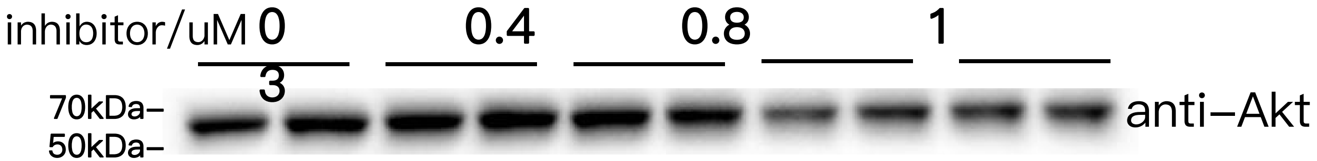

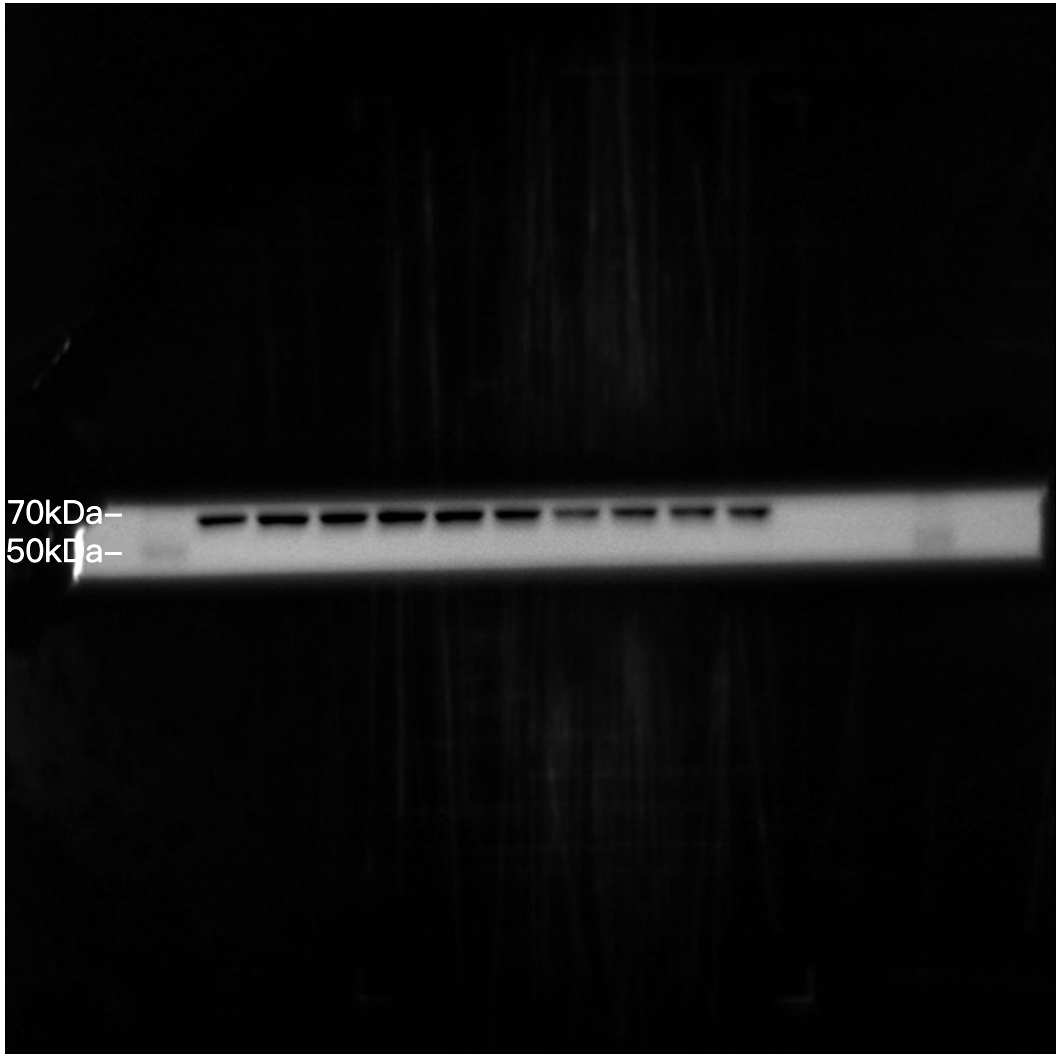

Figure 5E

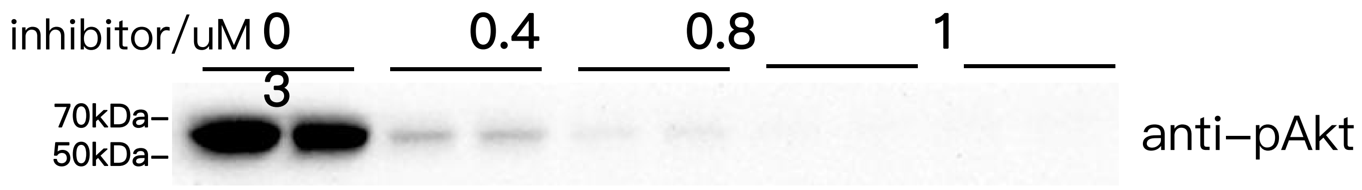

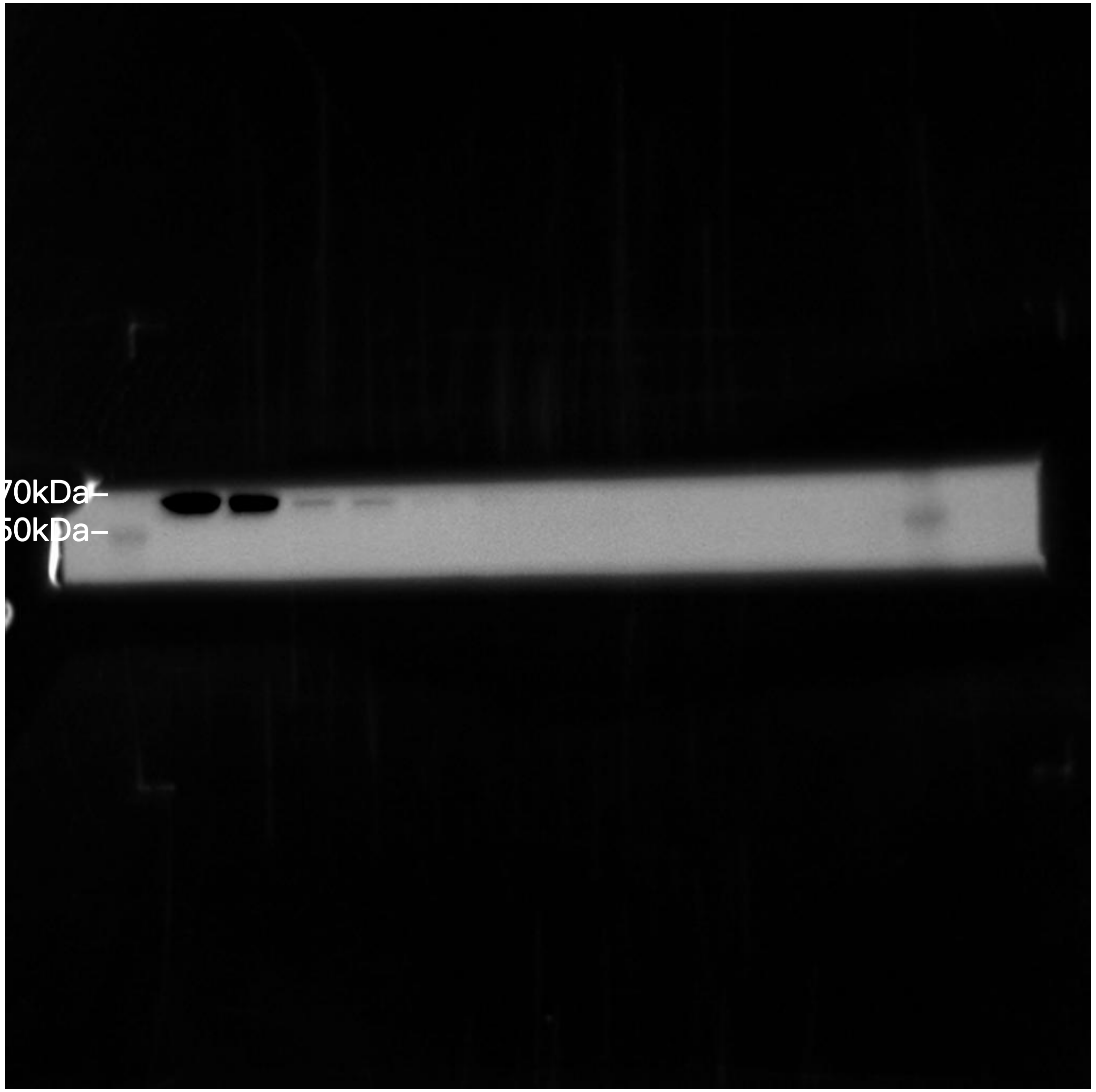

Figure 5E

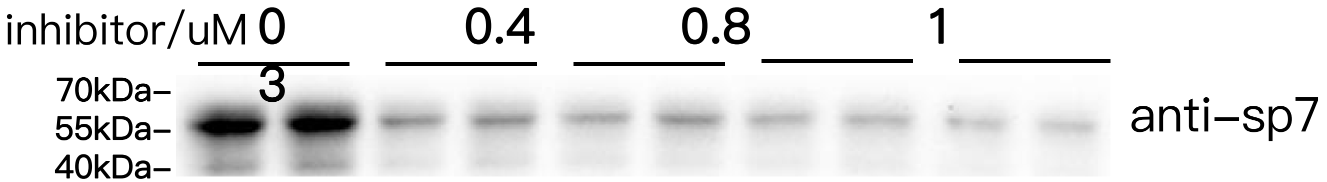
inhibitor/uM 0
0.4
0.8
1
3
70kDa–
55kDa–
40kDa–
anti–sp7

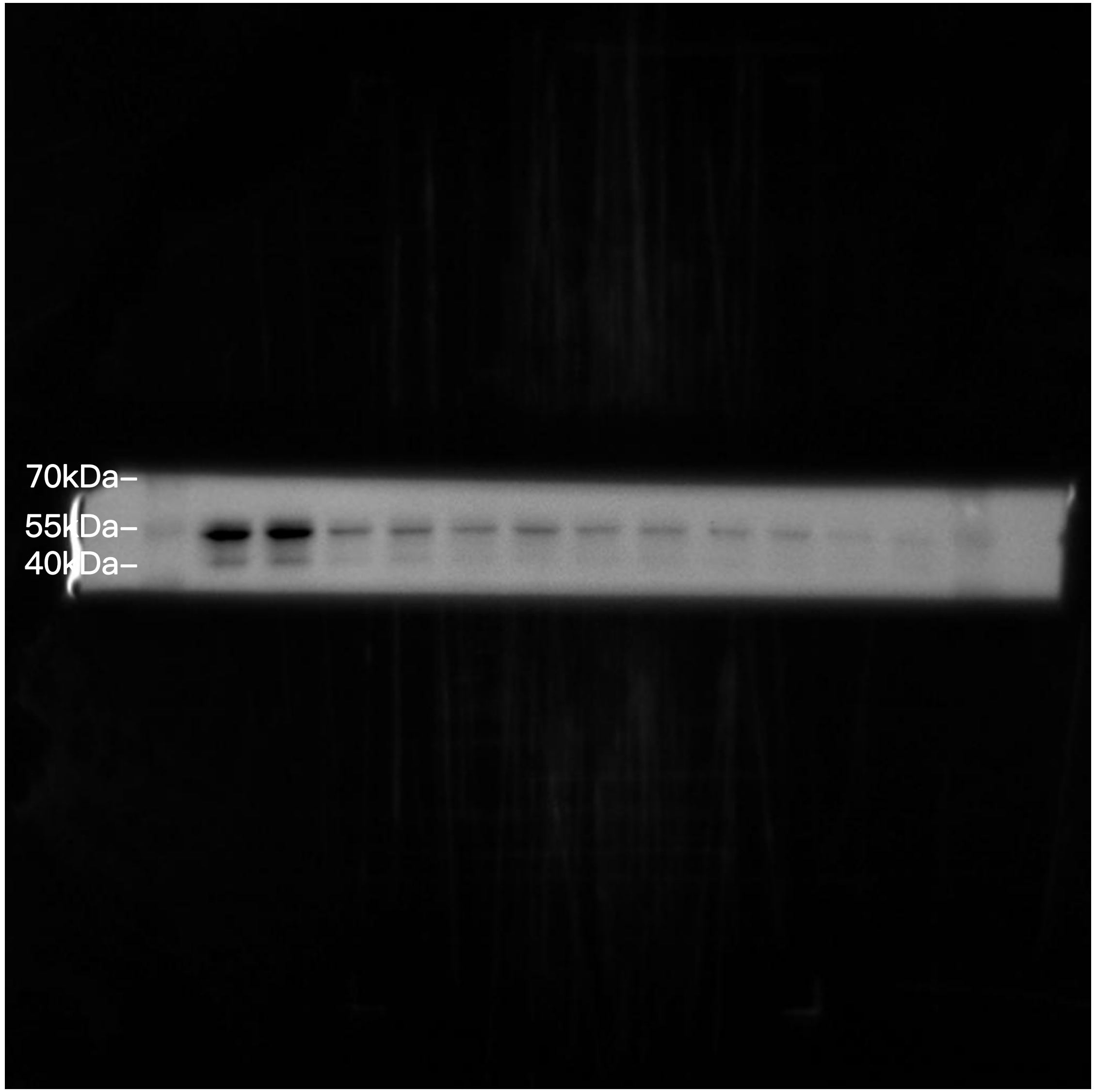
70kDa–
55kDa–
40kDa–

Figure 5F

Figure 5F

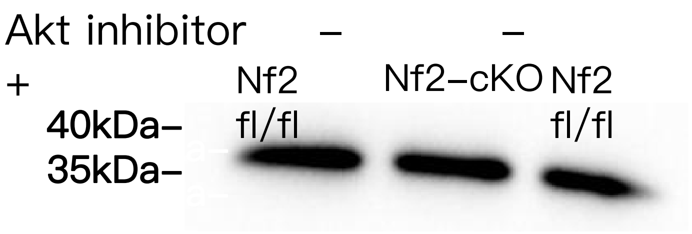

anti–GAPDH

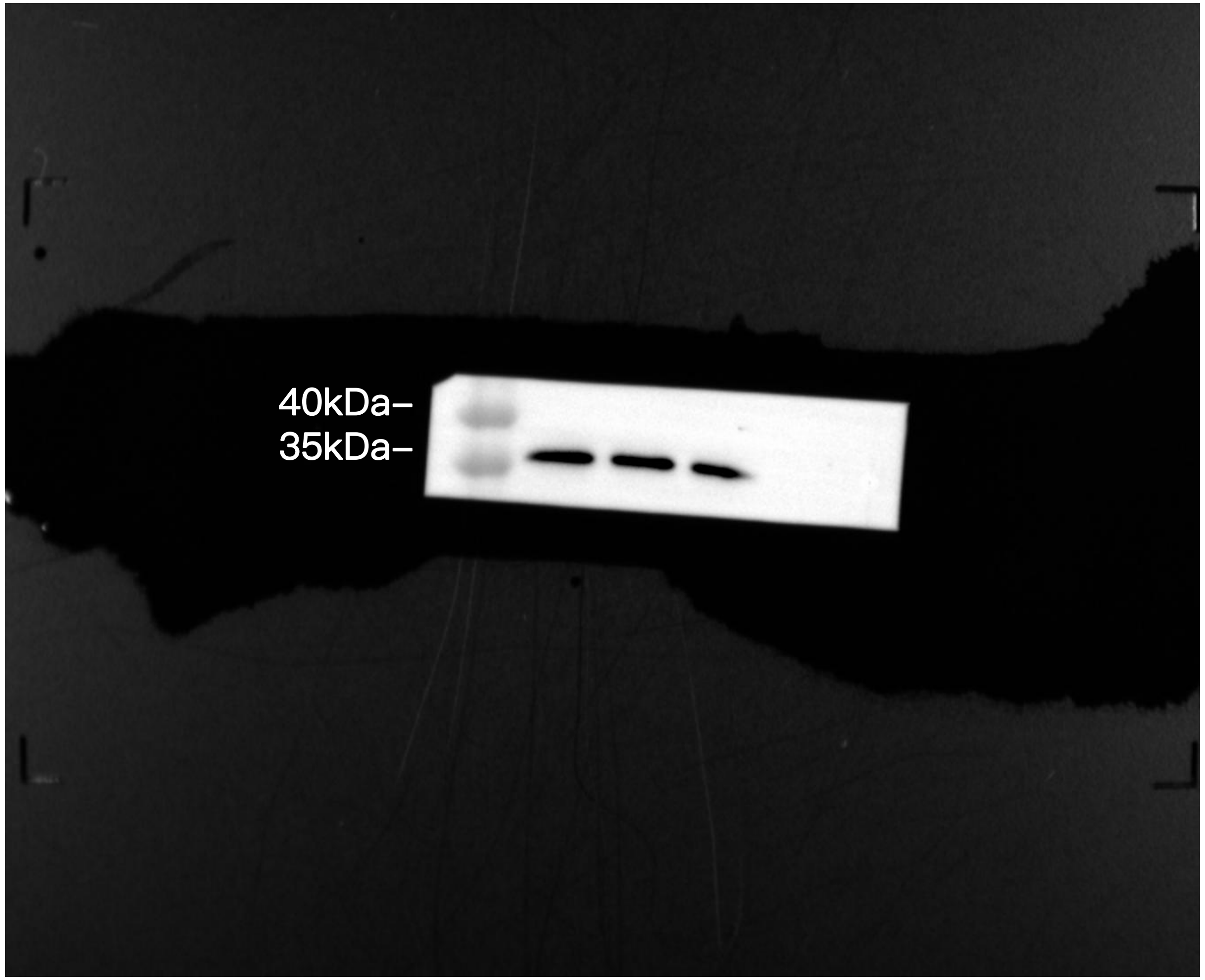

Figure 5F

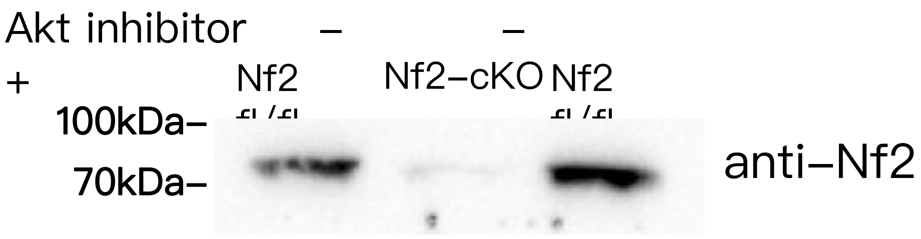

Akt inhibitor
–
–
+
Nf2 fl/fl
Nf2–cKO
Nf2 fl/fl
100kDa–
70kDa–
anti–Nf2

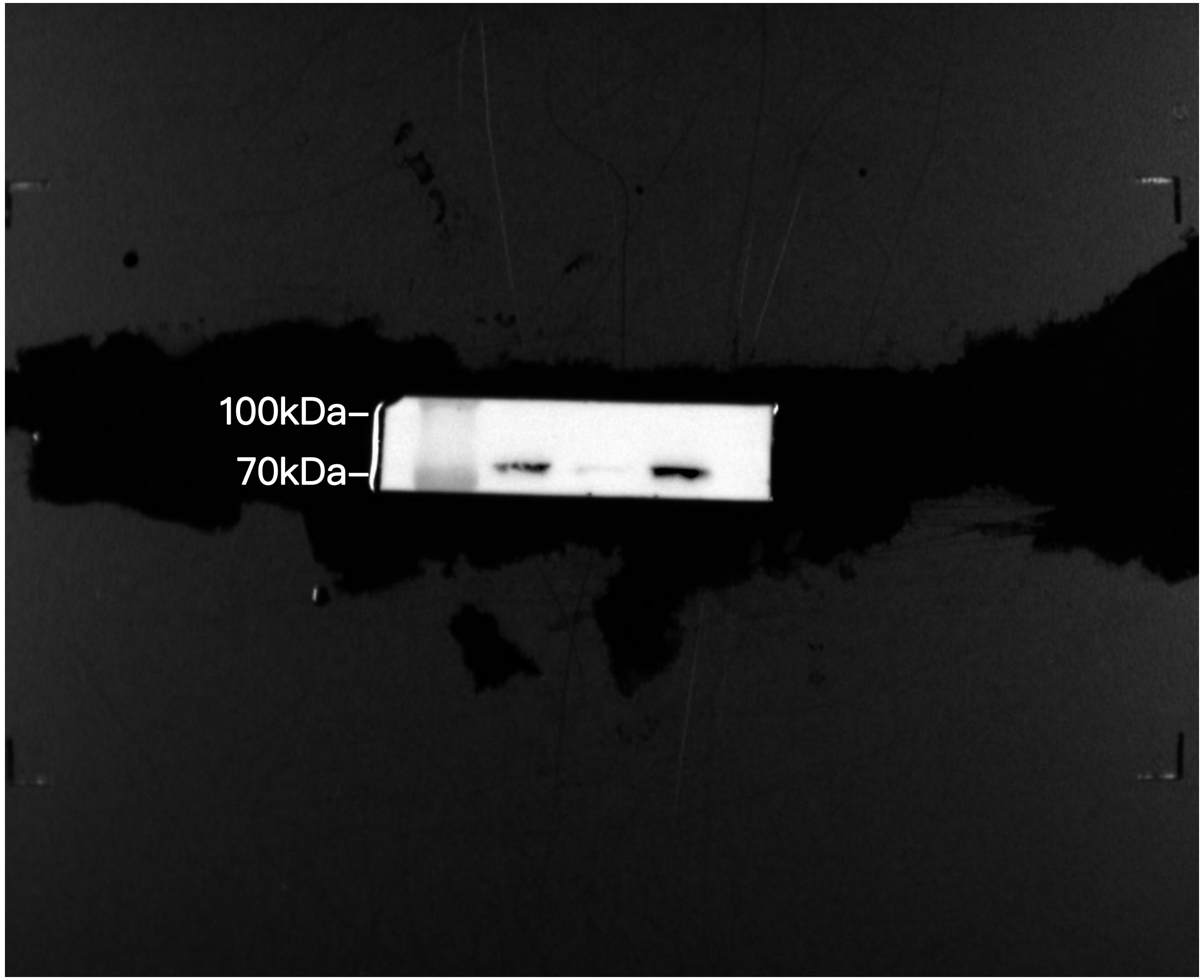

100kDa–
70kDa–

Figure 5F

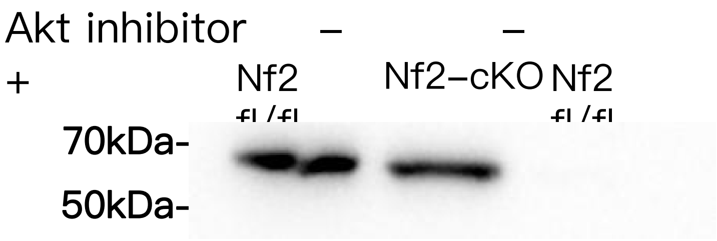

anti–
pAkt

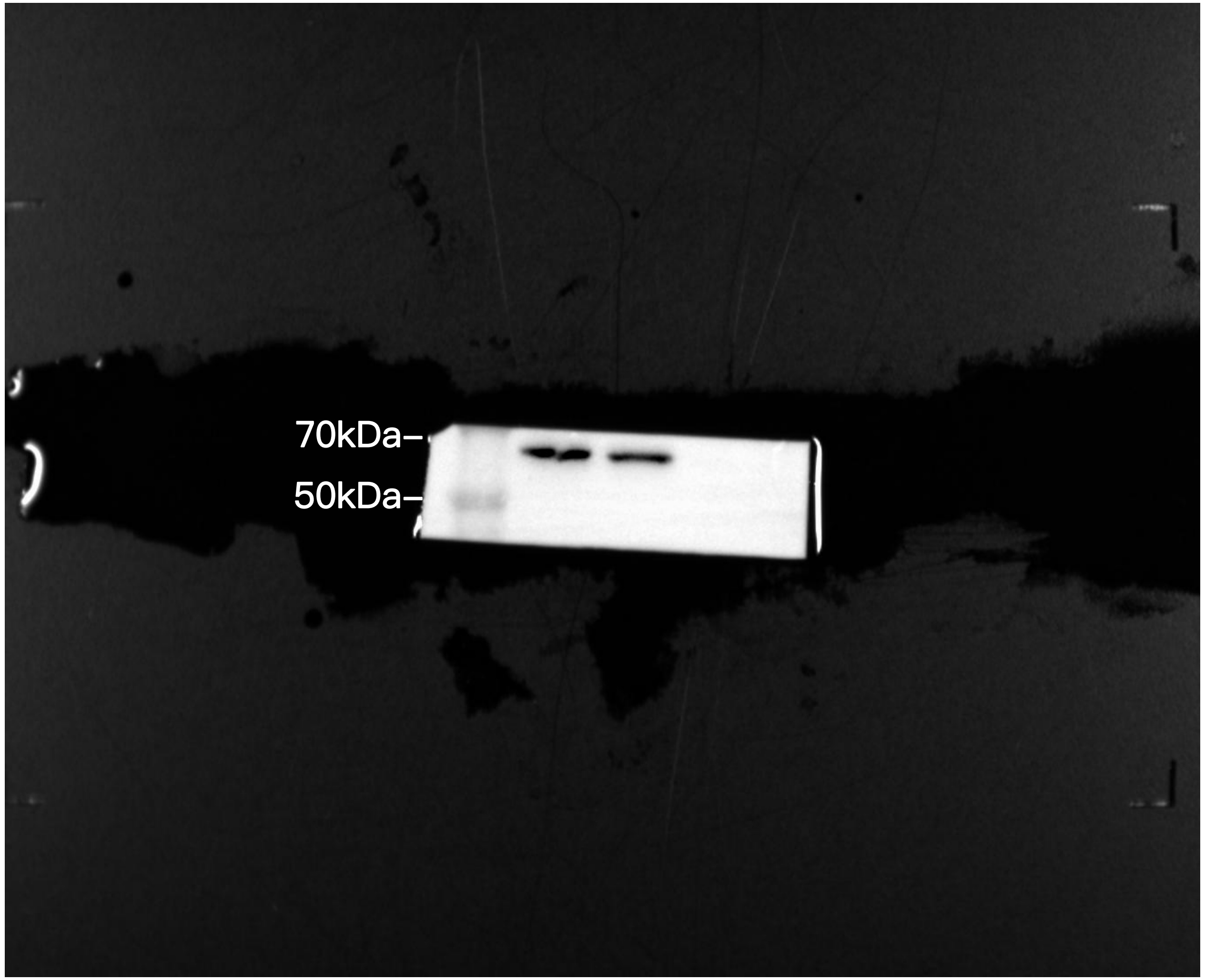

Figure 5F

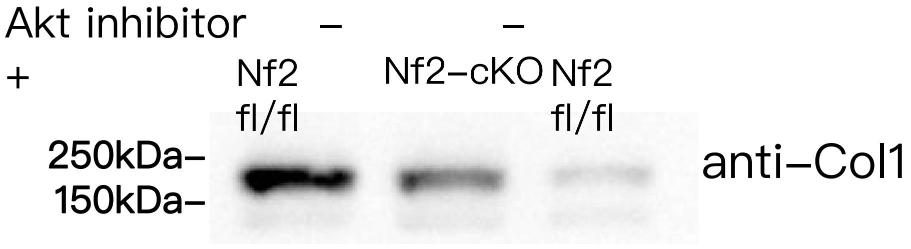

Akt inhibitor – – +
Nf2 fl/fl
Nf2–cKO
Nf2 fl/fl
250kDa–
150kDa–
anti–Col1

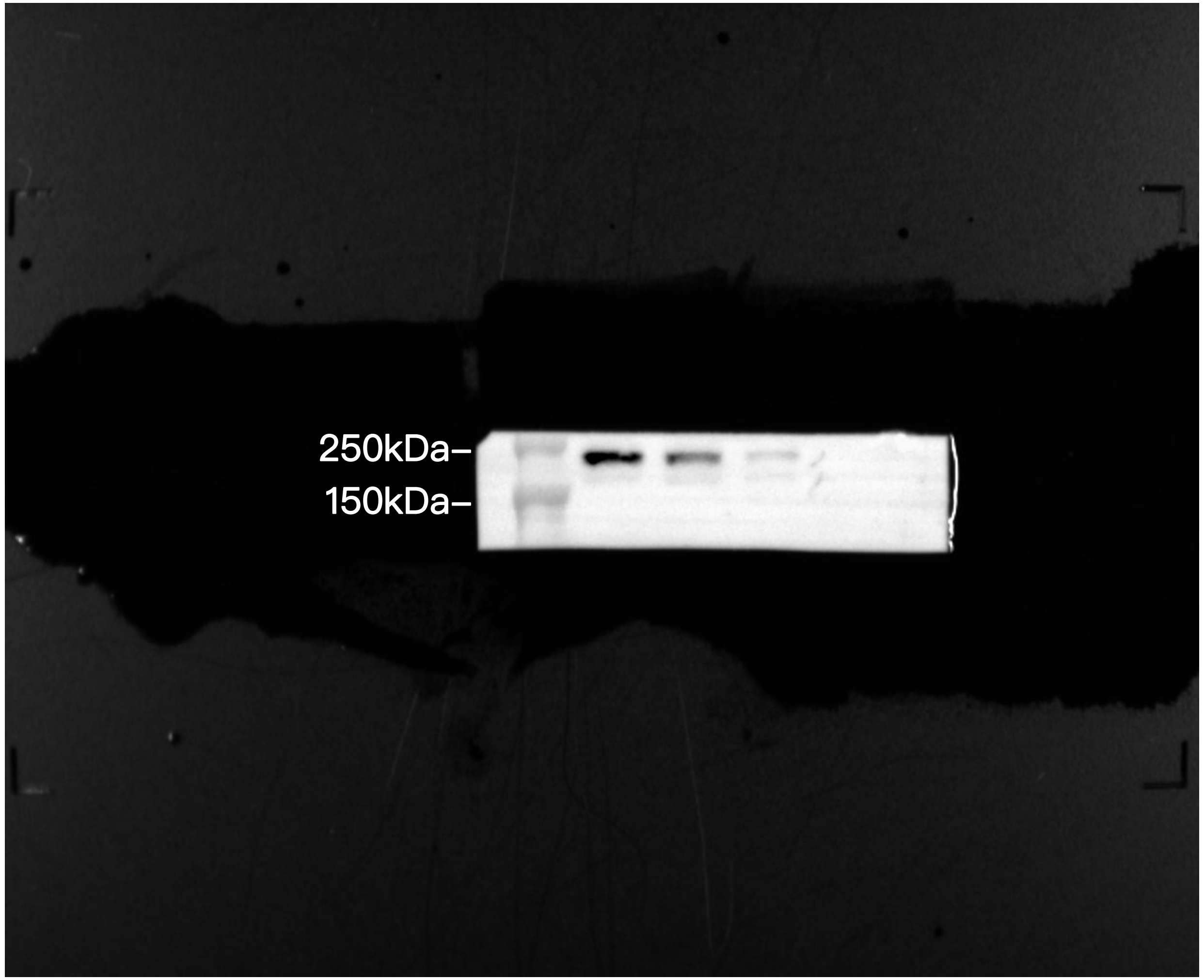

250kDa–
150kDa–

Figure 5H

Figure 5H

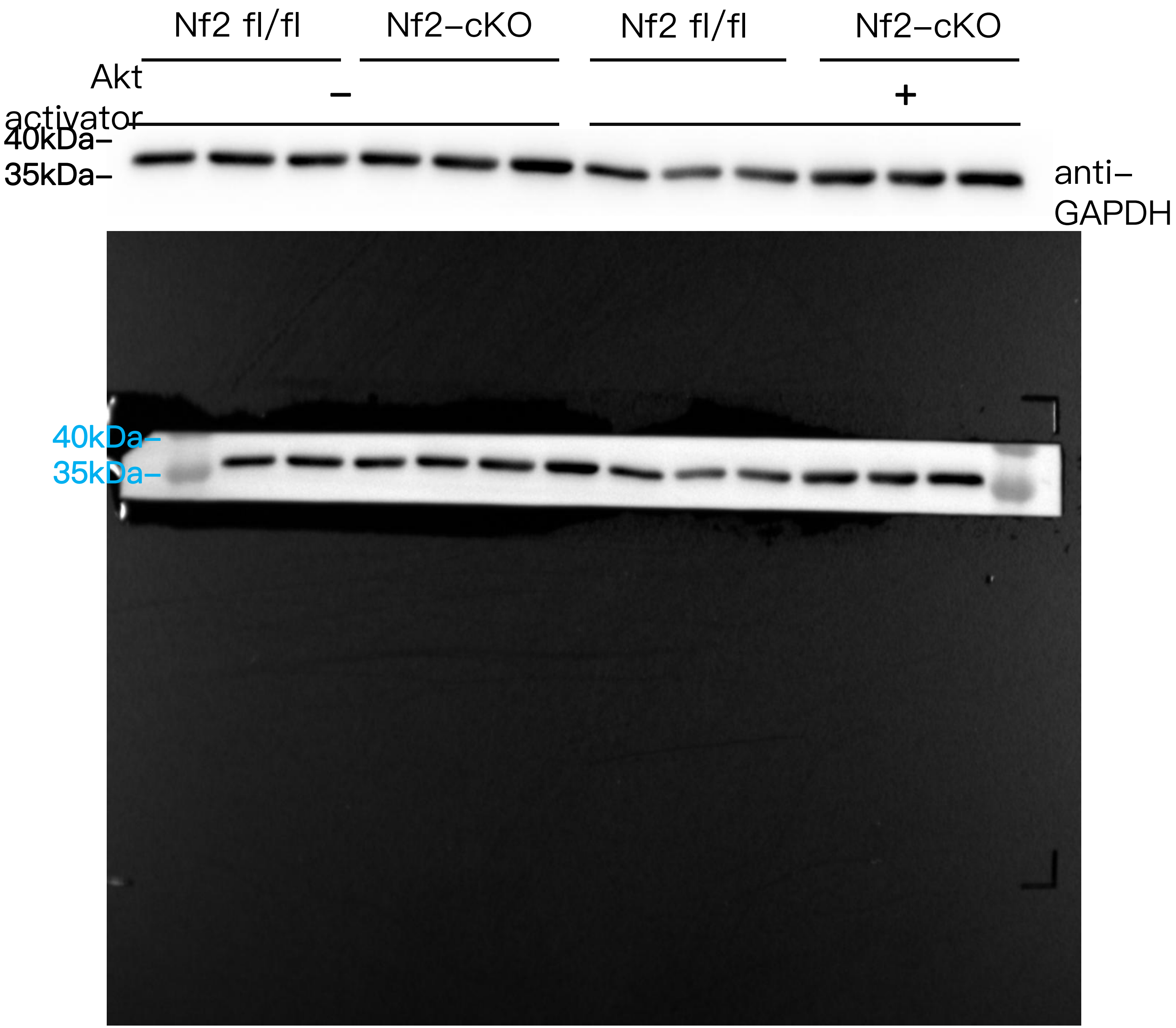

Nf2 fl/fl
Nf2−cKO
Nf2 fl/fl
Nf2−cKO
Akt activator
−
+
40kDa−
35kDa−
anti−GAPDH
40kDa−
35kDa−

Figure 5H

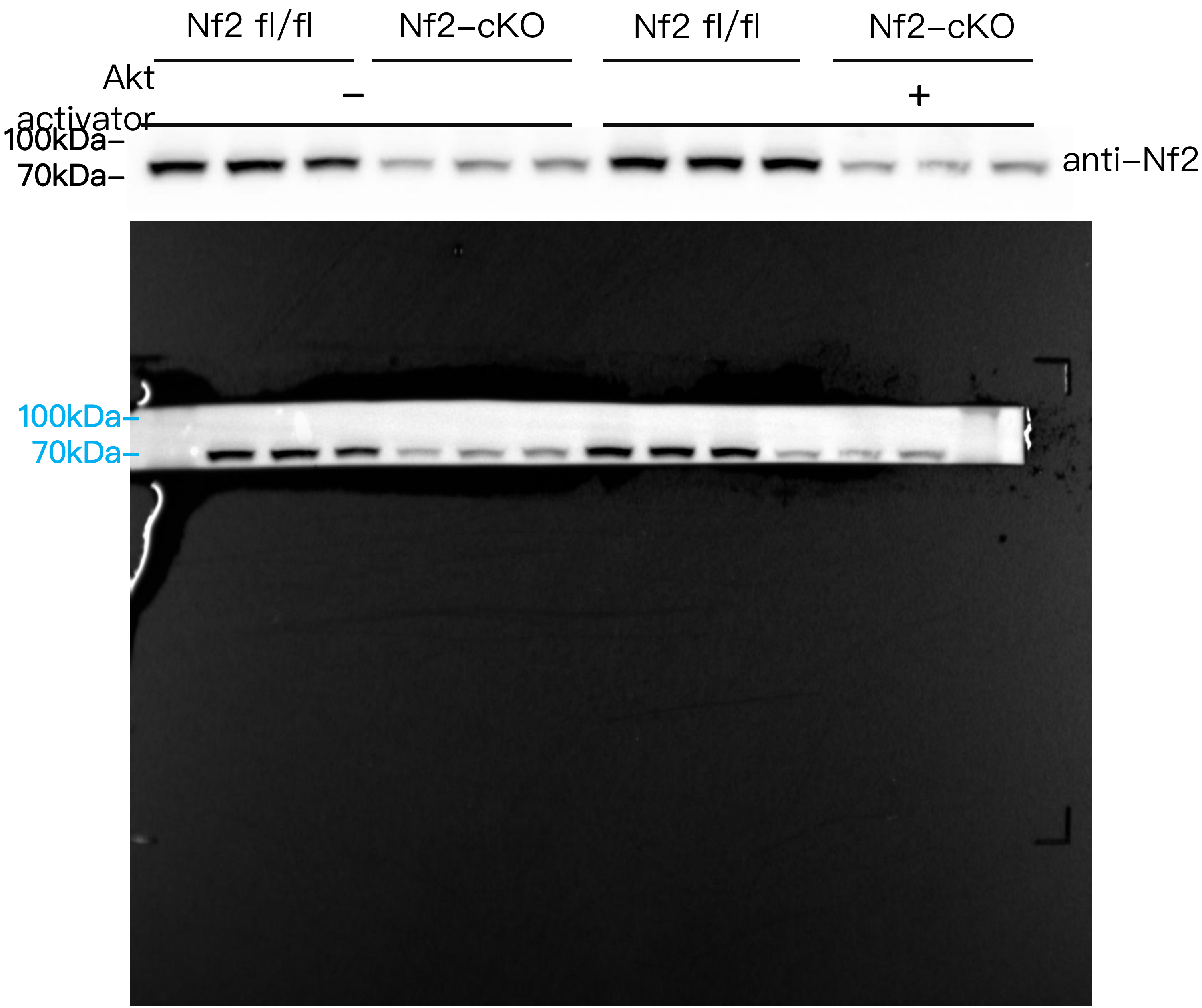

Nf2 fl/fl
Nf2−cKO
Nf2 fl/fl
Nf2−cKO
Akt activator
−
+
100kDa−
70kDa−
anti−Nf2
100kDa−
70kDa−

Figure 5H

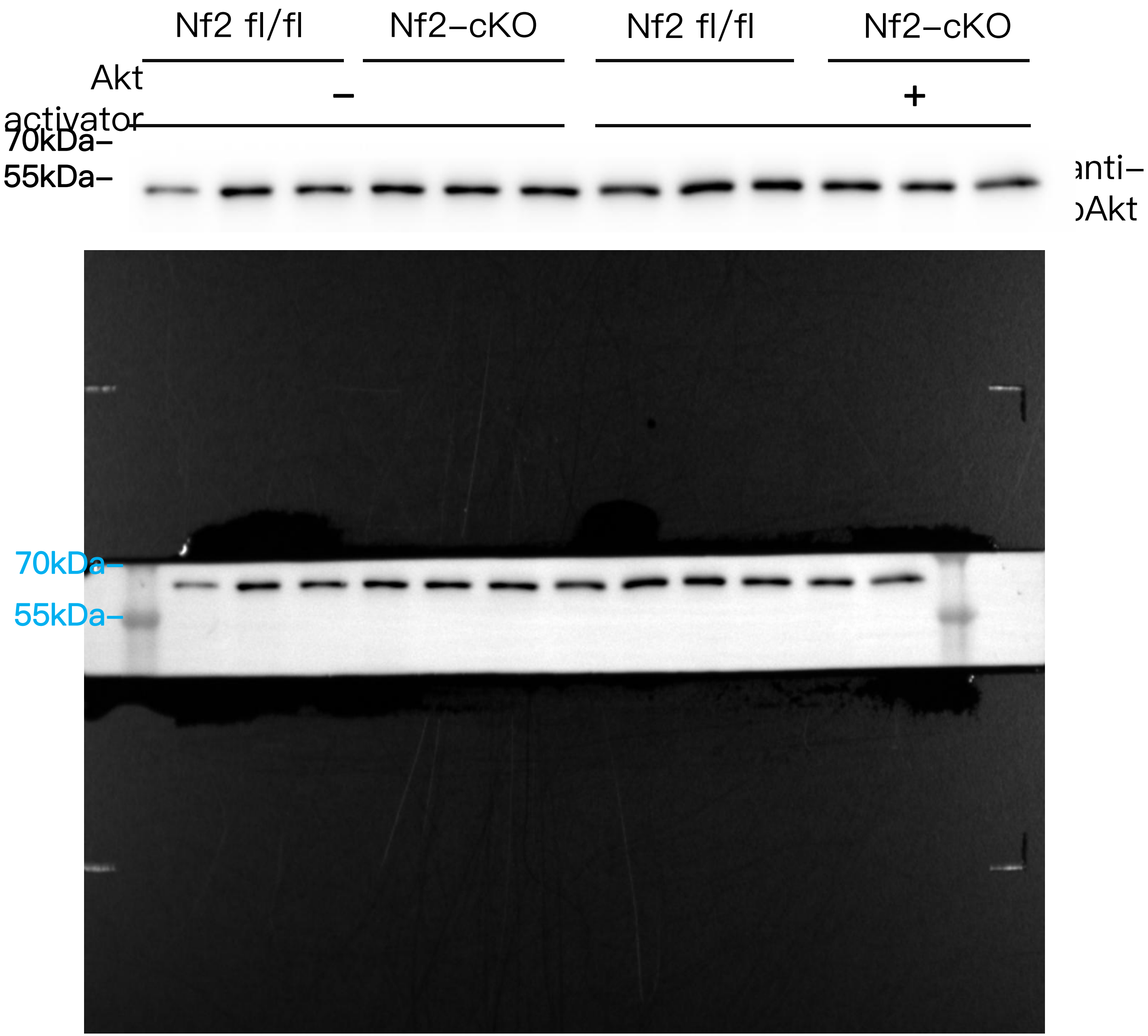
Nf2 fl/fl
Nf2−cKO
Nf2 fl/fl
Nf2−cKO
Akt activator
−
+
70kDa−
55kDa−
anti−
pAkt
70kDa−
55kDa−

Figure 5H

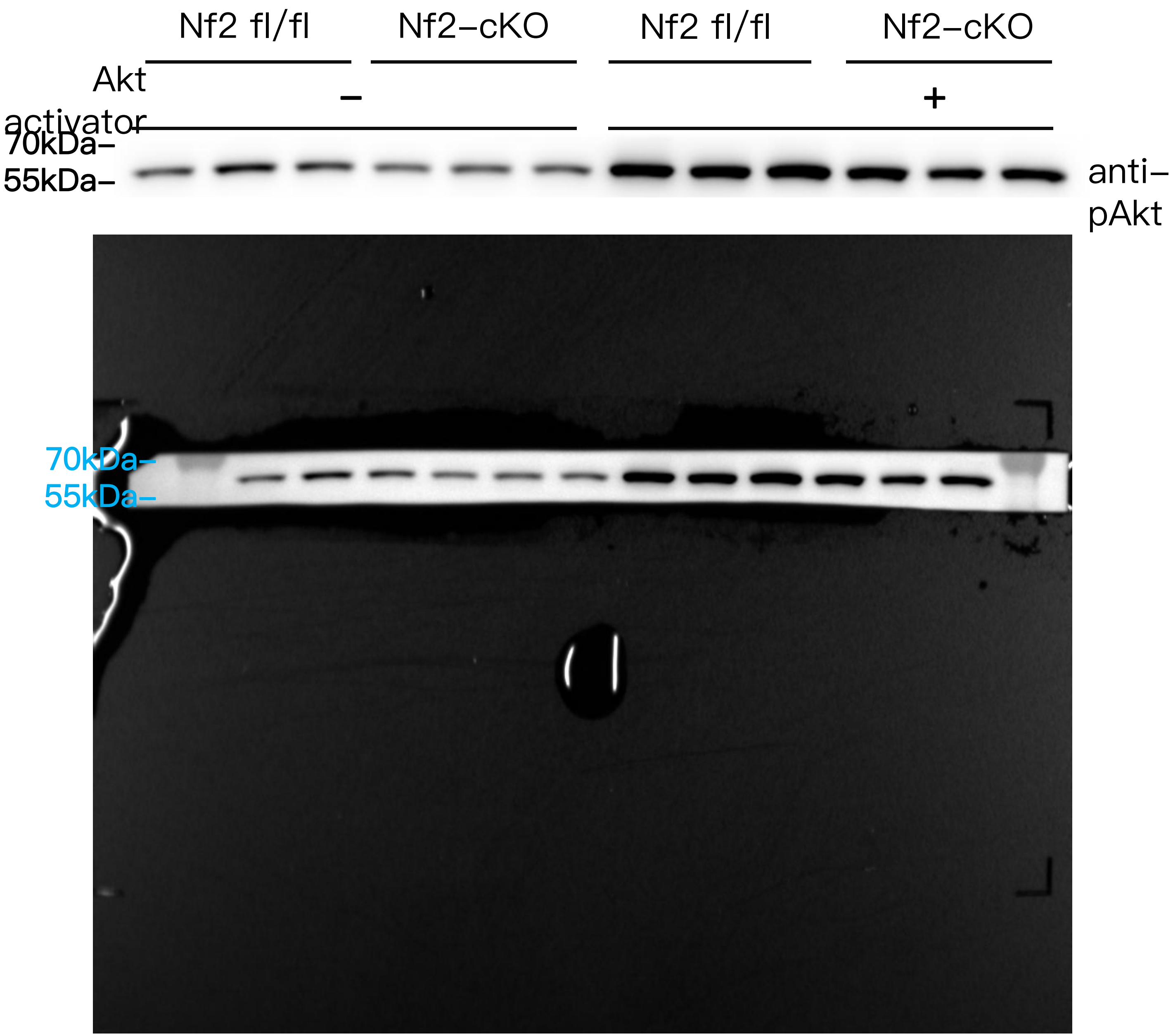
Nf2 fl/fl
Nf2–cKO
Nf2 fl/fl
Nf2–cKO
Akt activator
–
+
70kDa–
55kDa–
anti–pAkt
70kDa–
55kDa–

Figure 5H

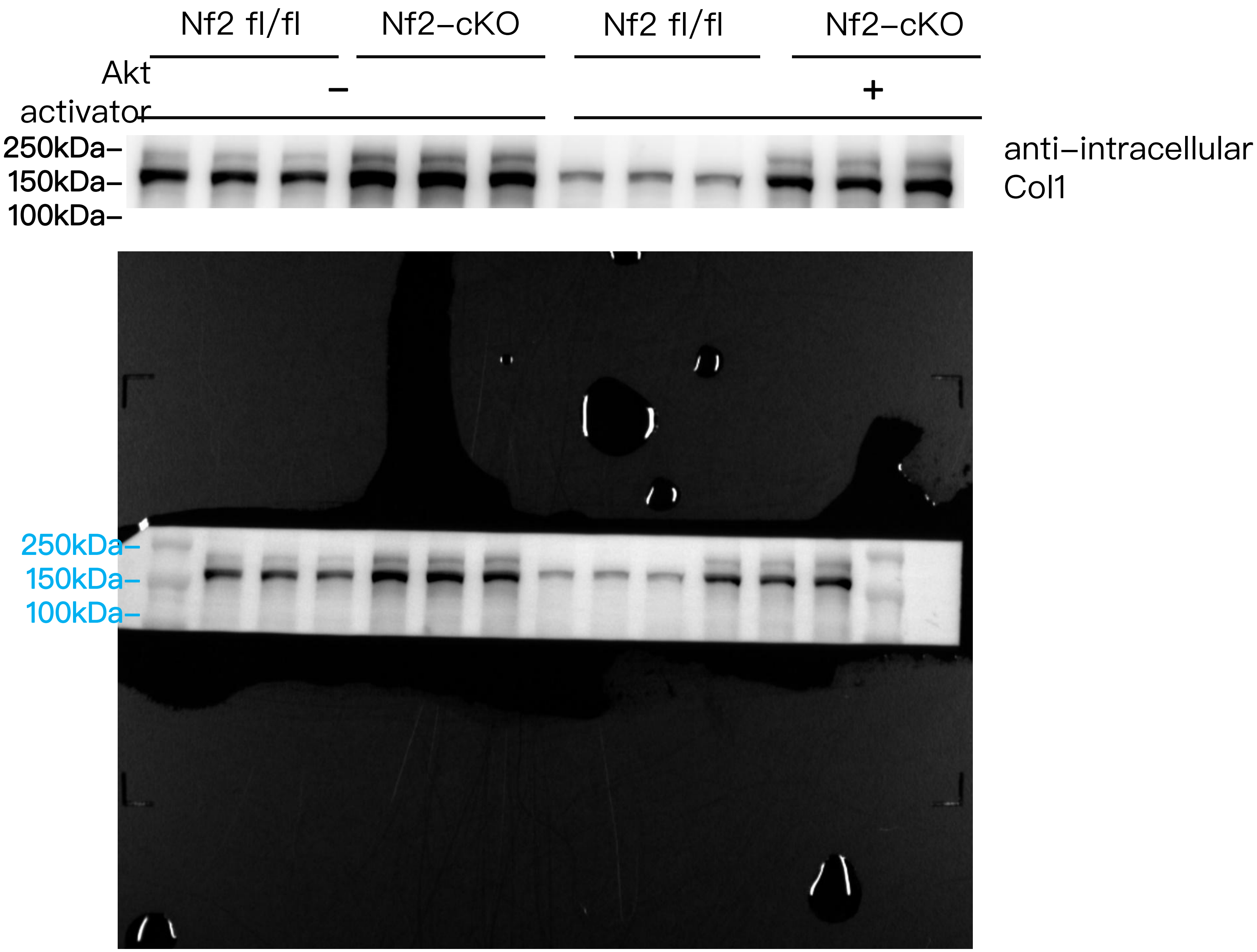
Nf2 fl/fl
Nf2−cKO
Nf2 fl/fl
Nf2−cKO
Akt activator
−
+
250kDa−
150kDa−
100kDa−
anti−intracellular Col1
250kDa−
150kDa−
100kDa−

Figure 6A

Figure 6A

2 Samples

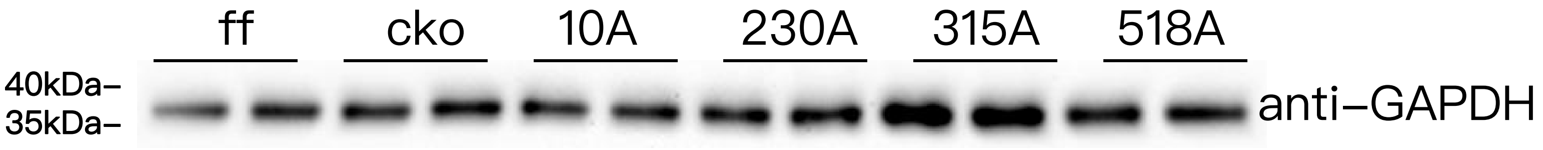

Show in Figure

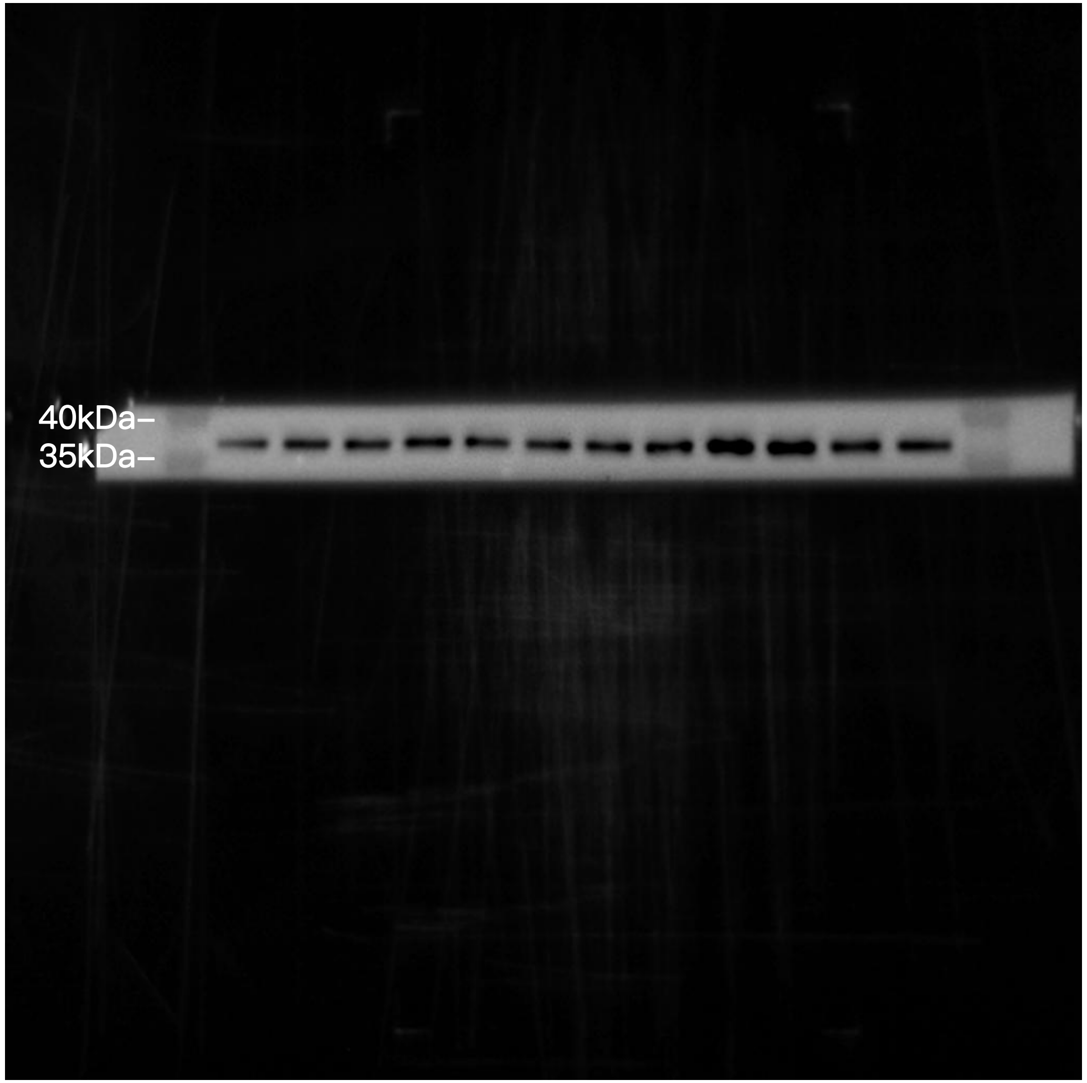

Figure 6A

2 Samples

Show in Figure

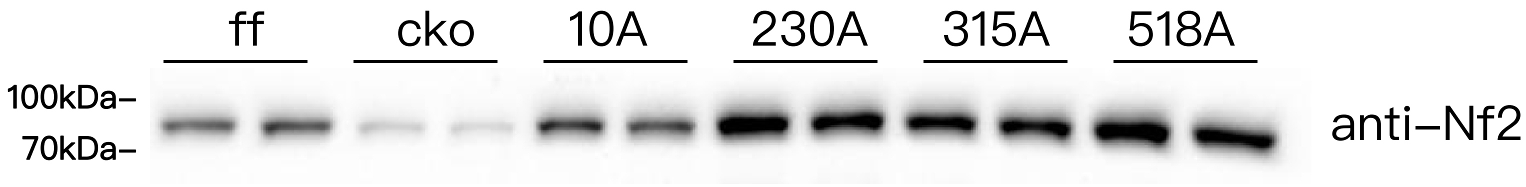

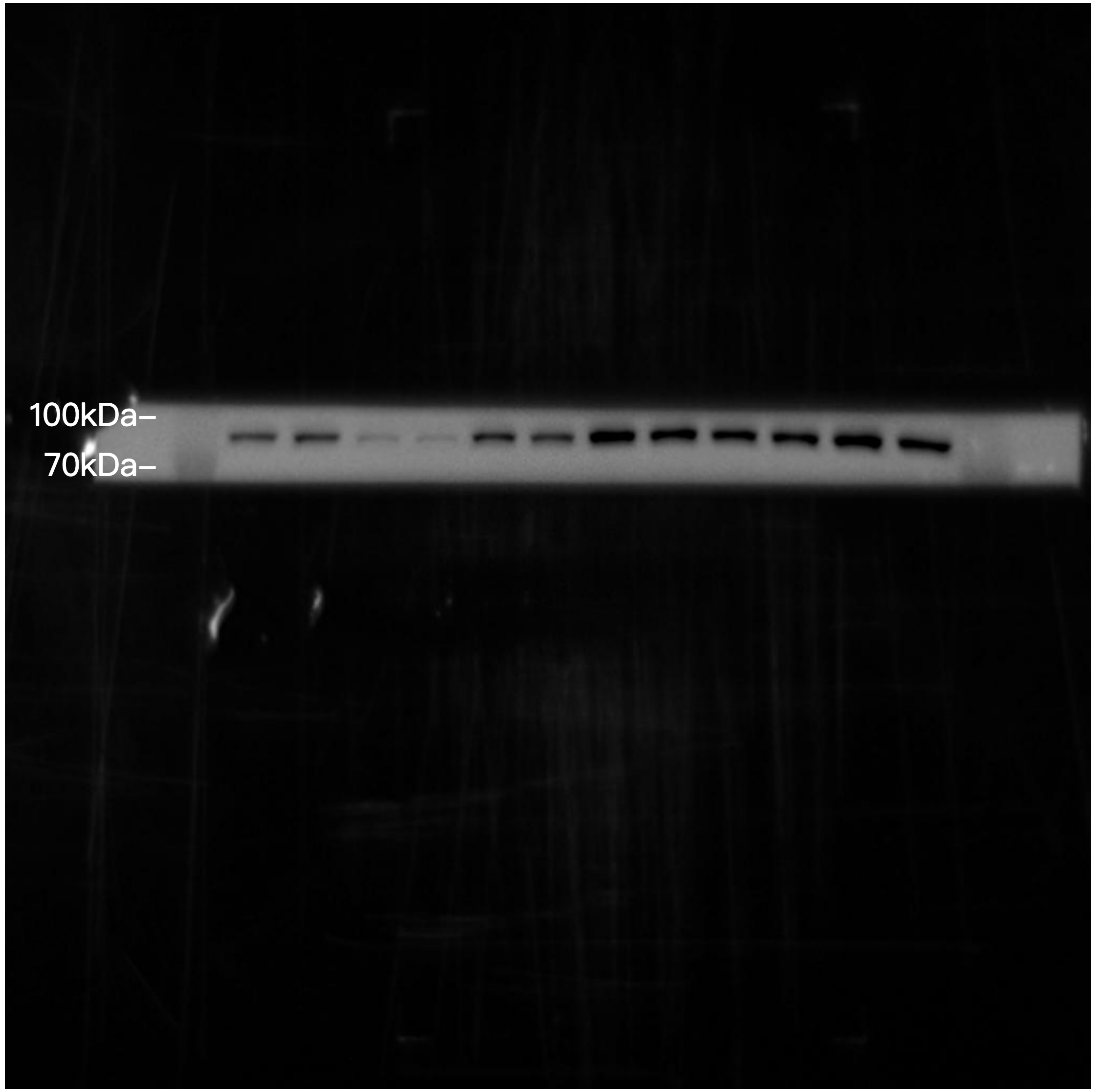

Figure 6A

2 Samples

Show in Figure

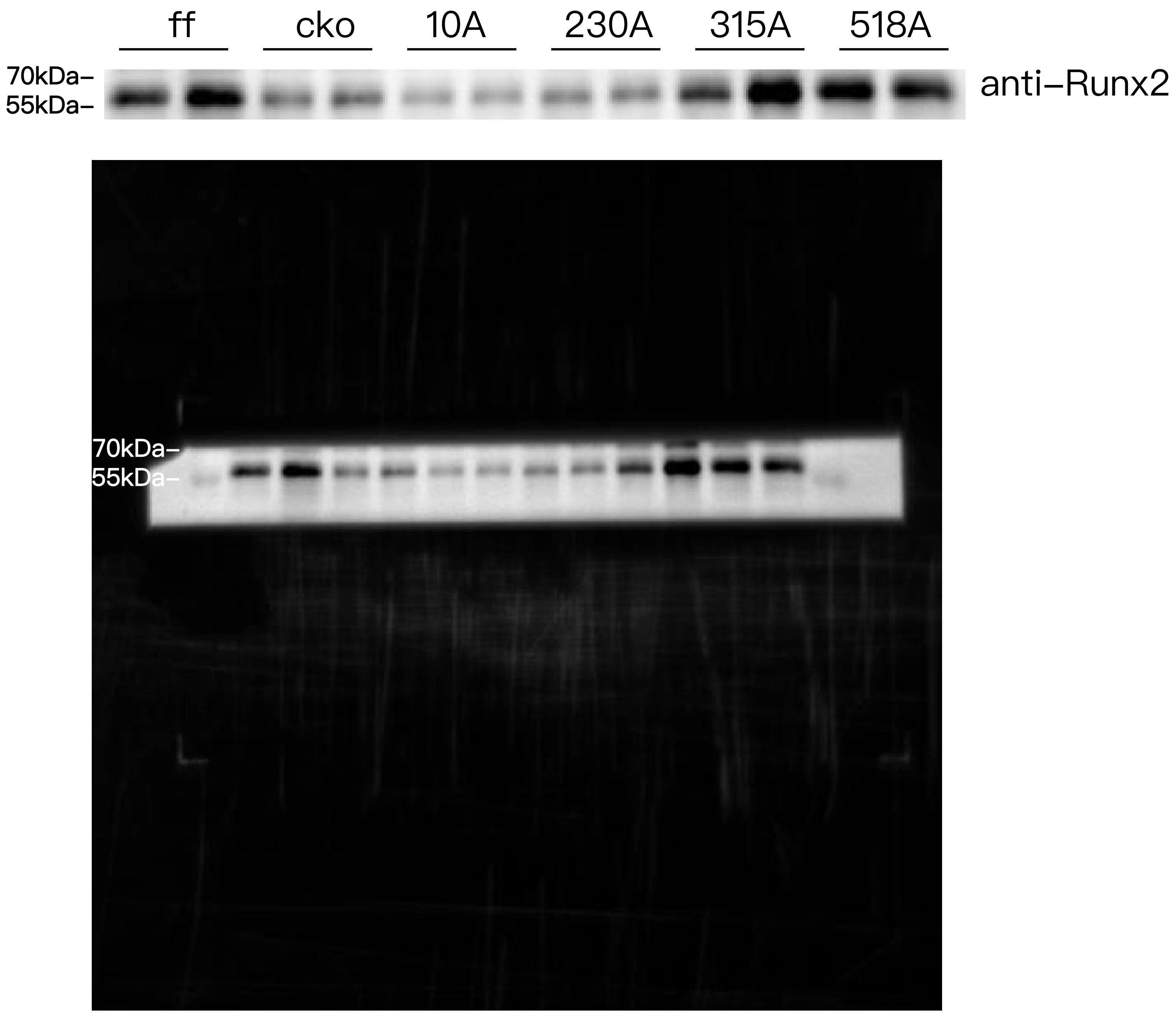

Figure 6A

other 2 Samples

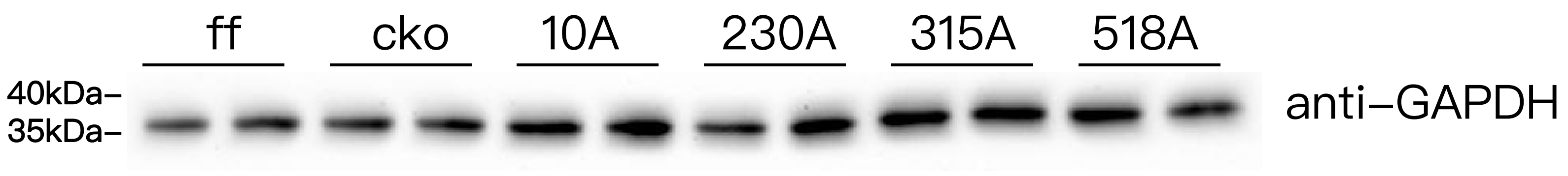

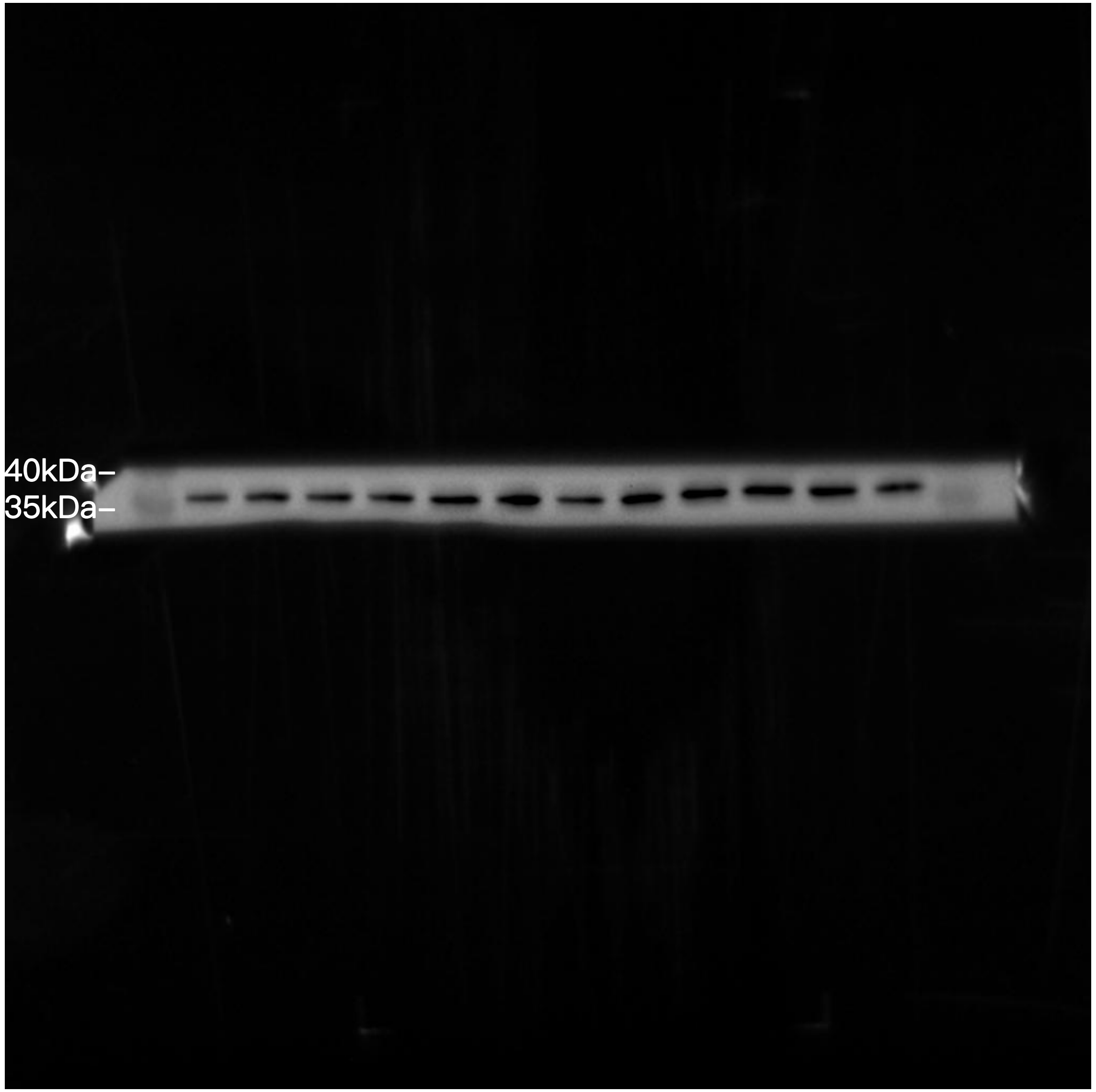

Figure 6A

other 2 Samples

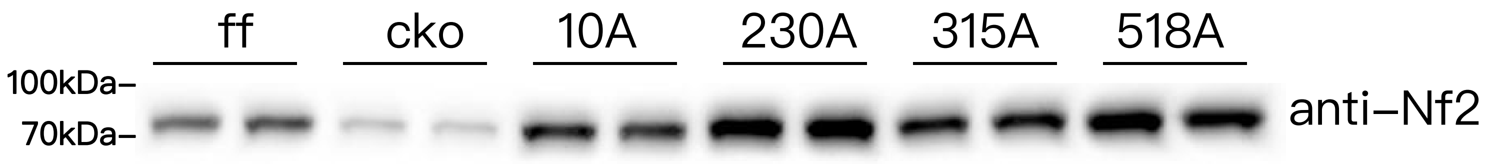

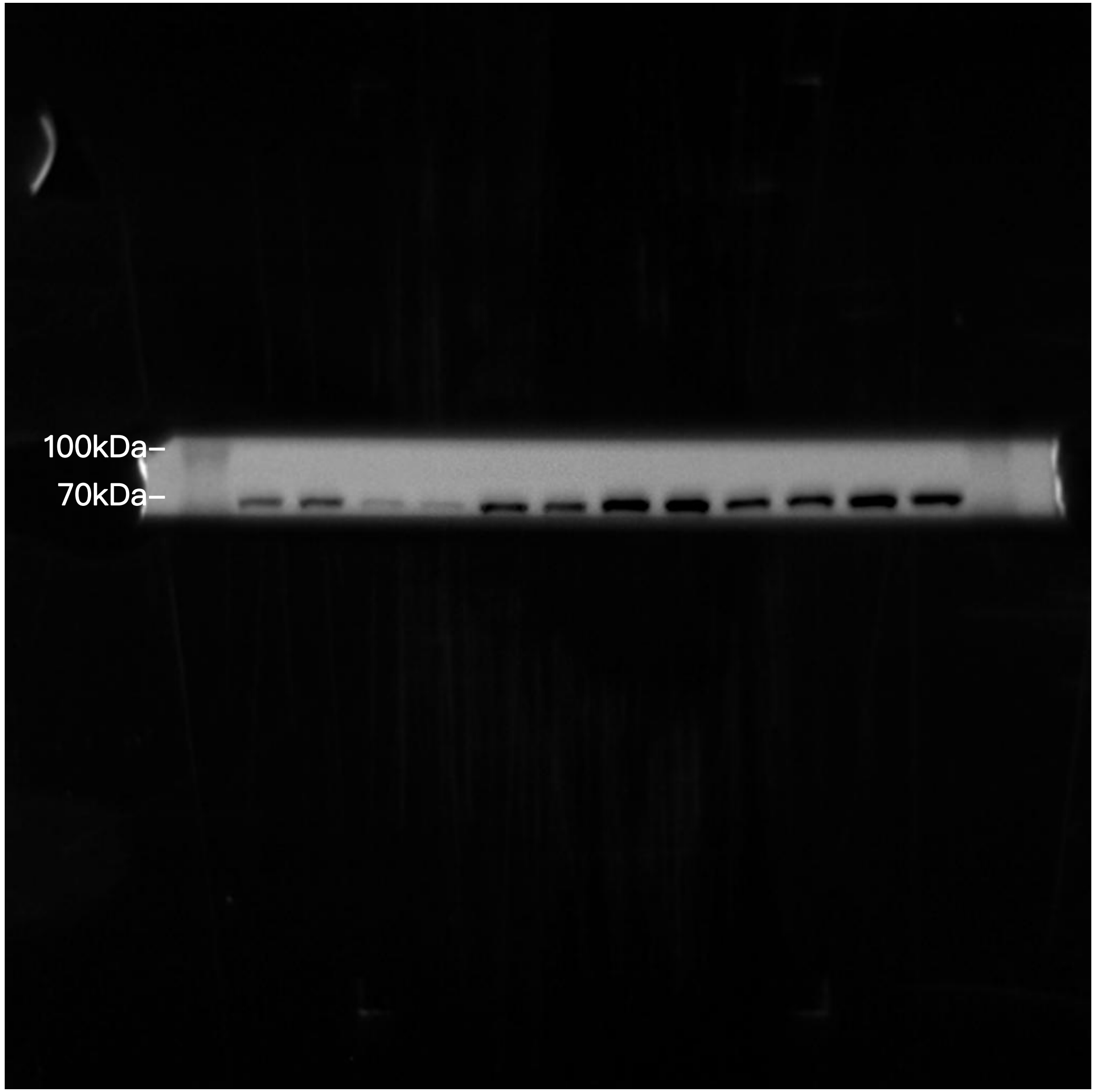

Figure 6A

other 2 Samples

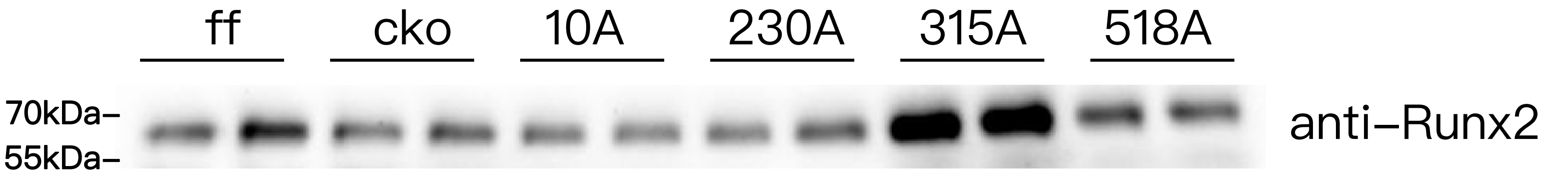

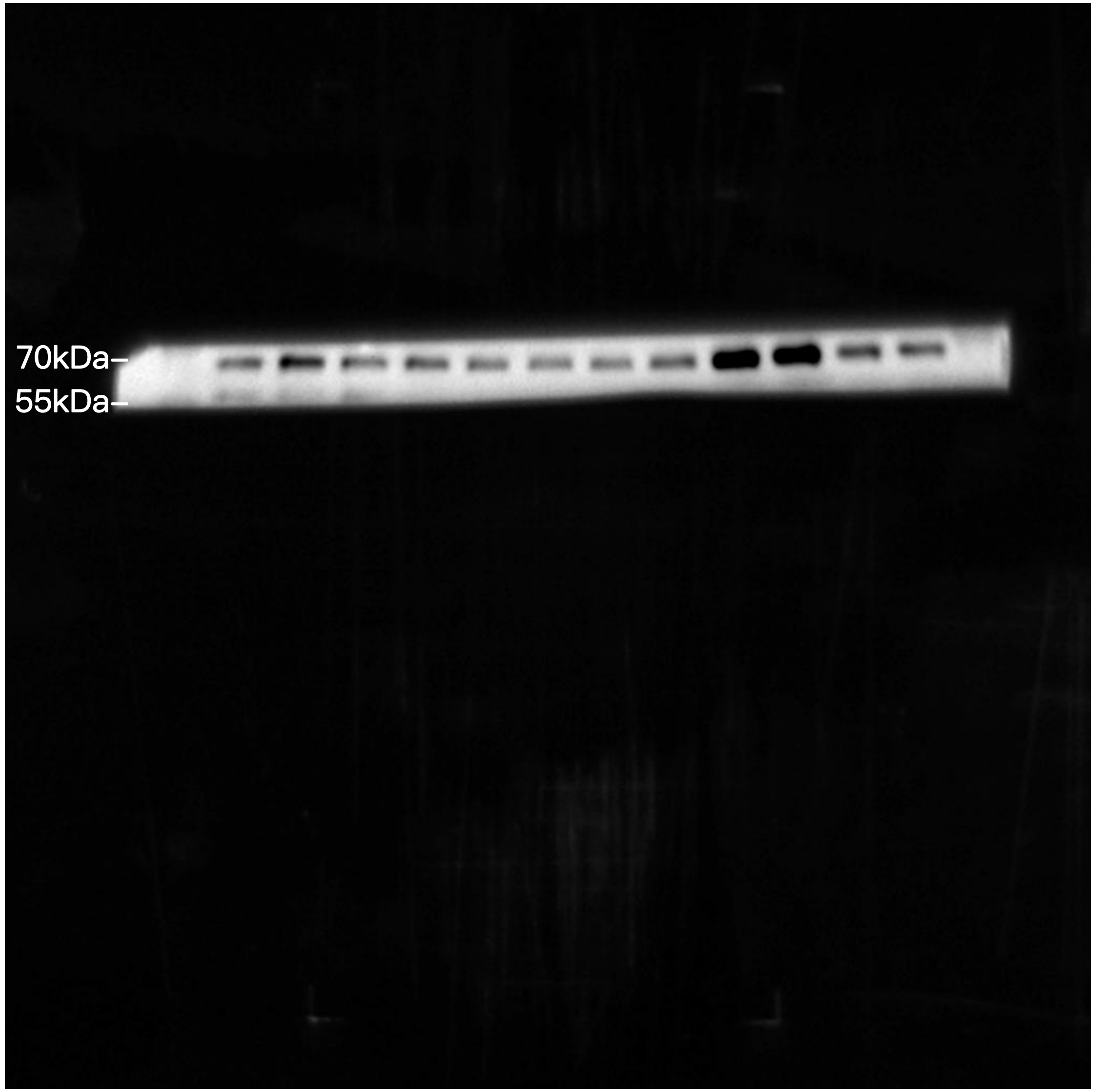

Figure 6E

Figure 6E

2 Samples

Show in Figure

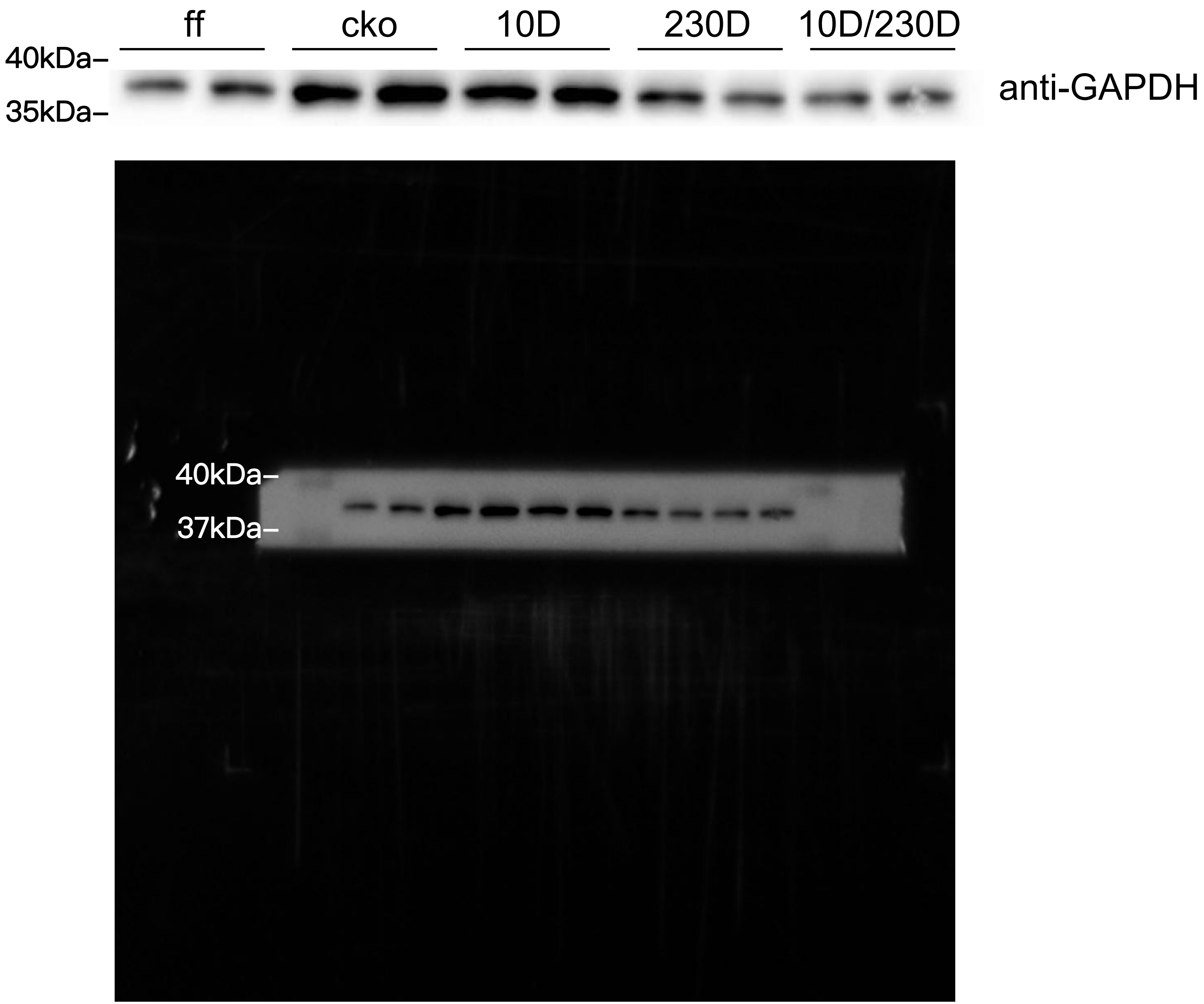

Figure 6E

2 Samples

Show in Figure

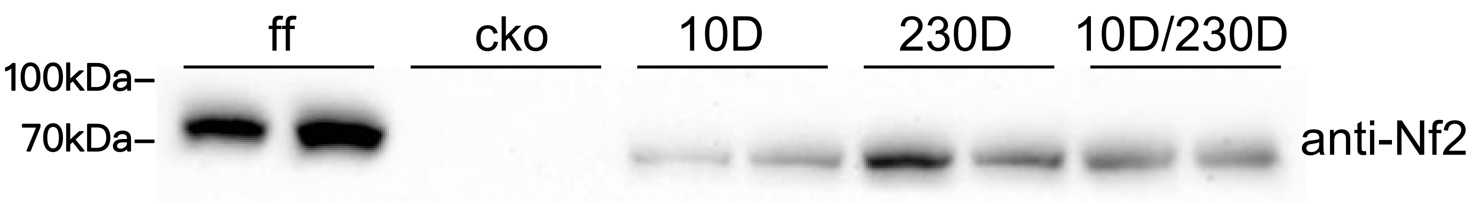

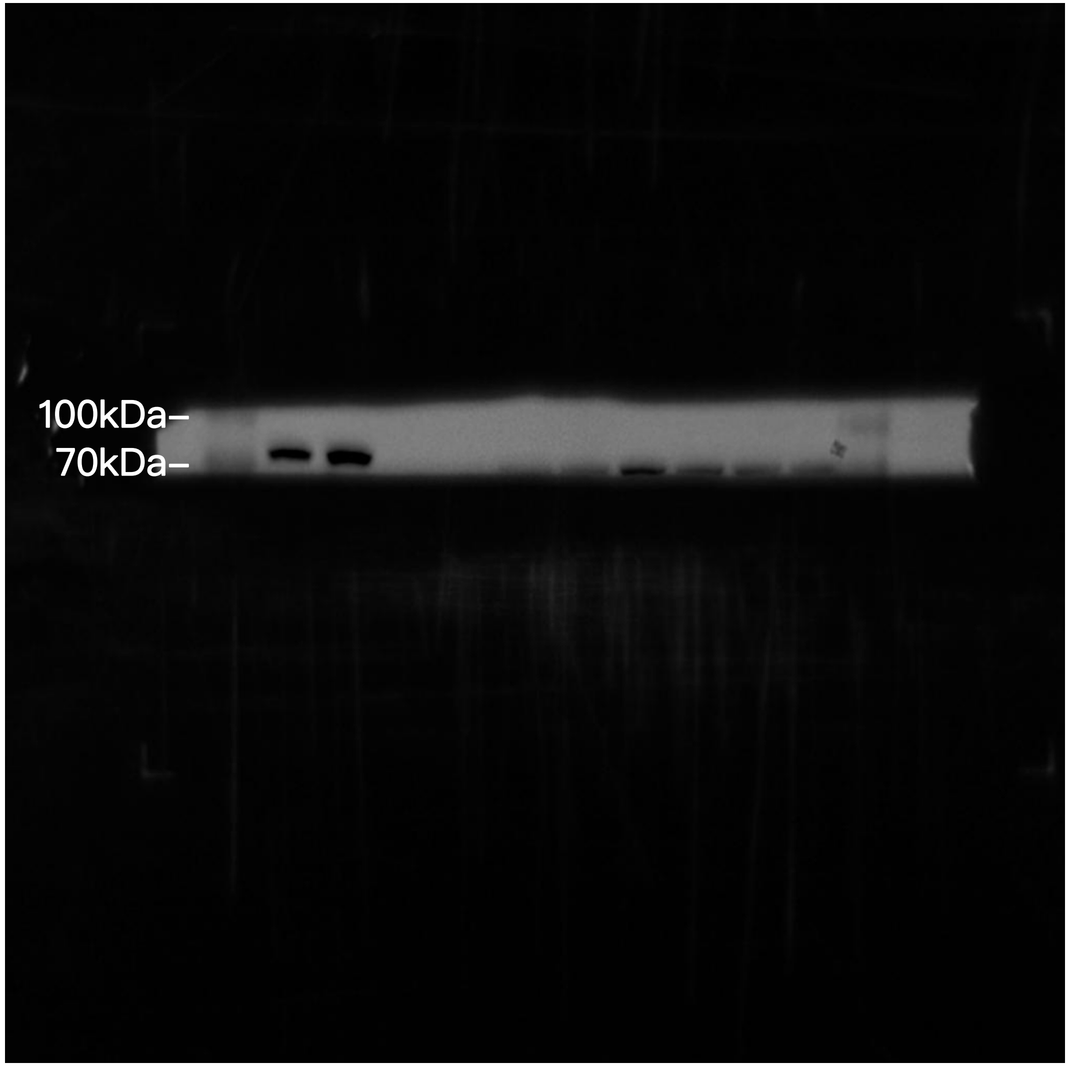

Figure 6E

2 Samples

Show in Figure

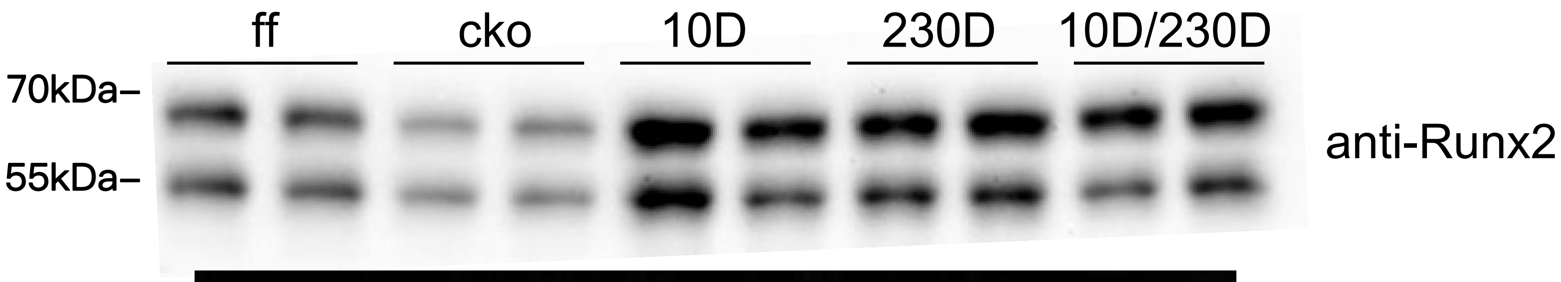

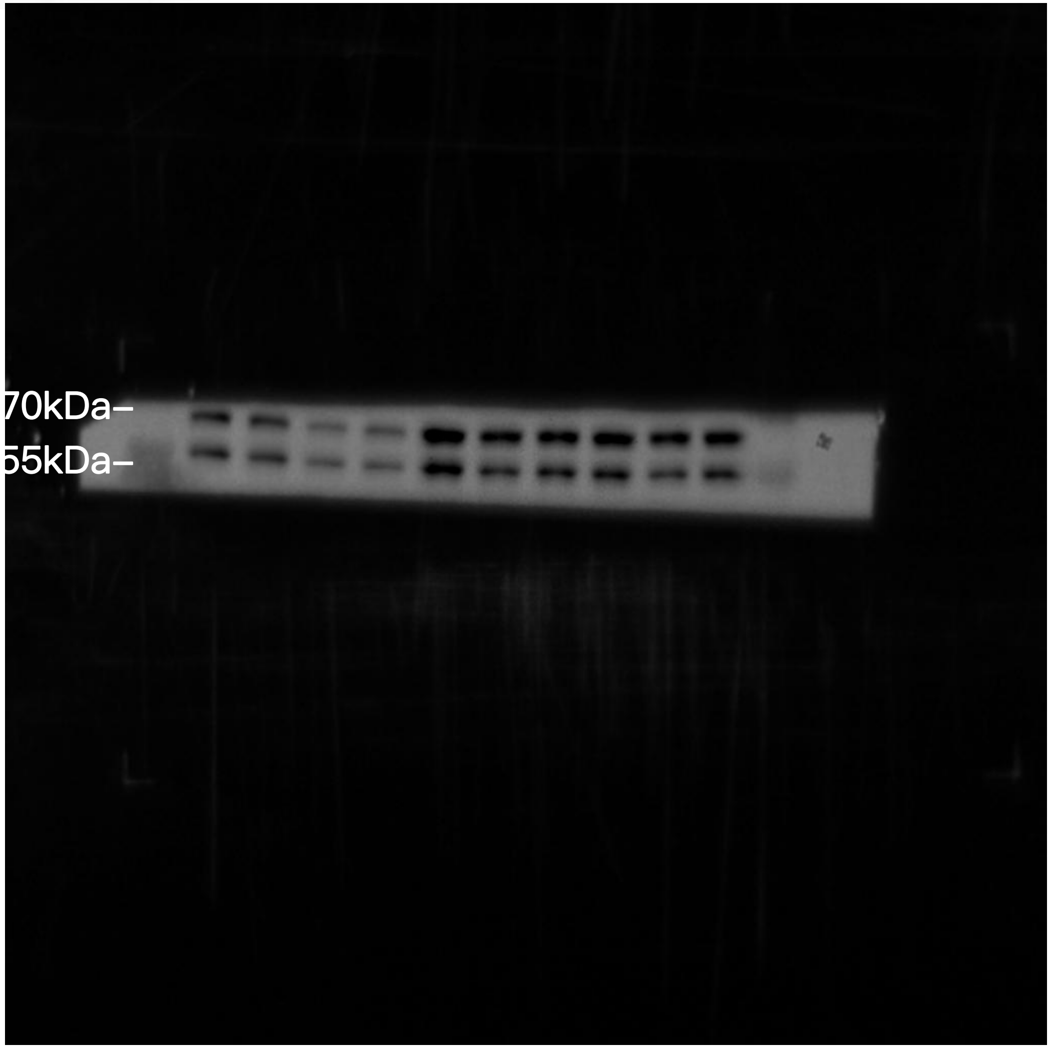

Figure 6E

other 2 Samples

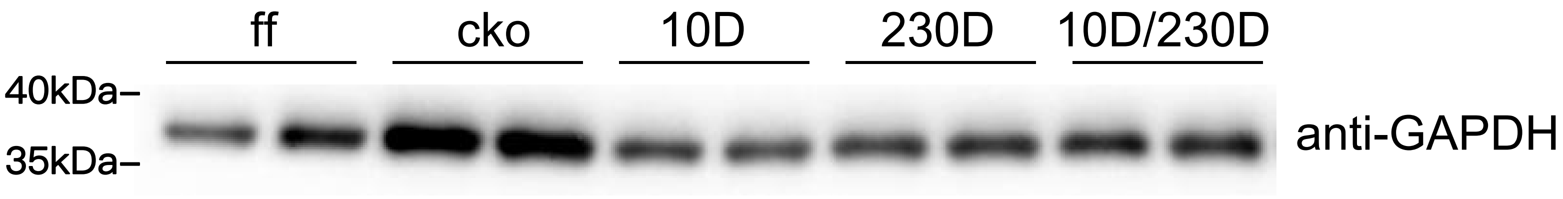

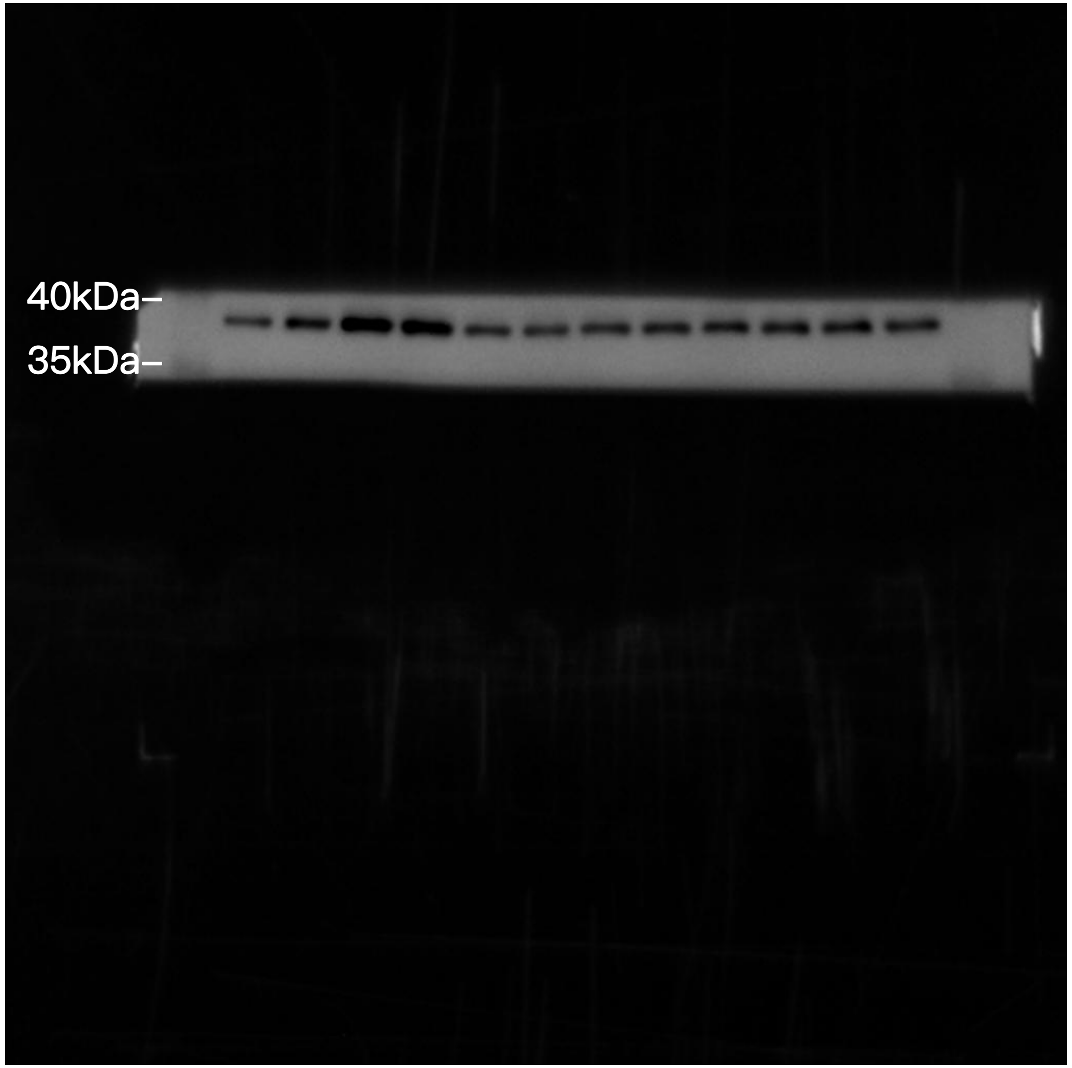

Figure 6E

other 2 Samples

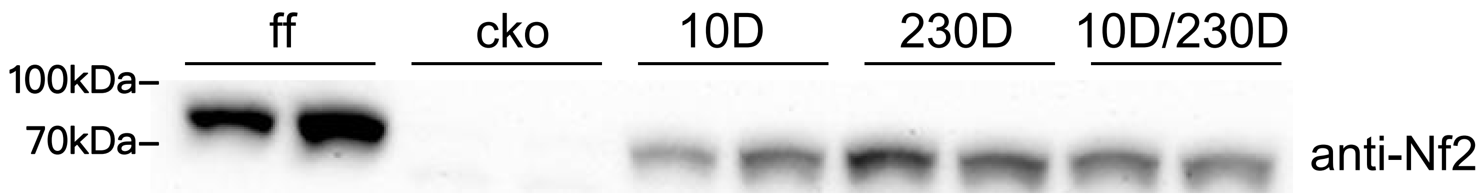

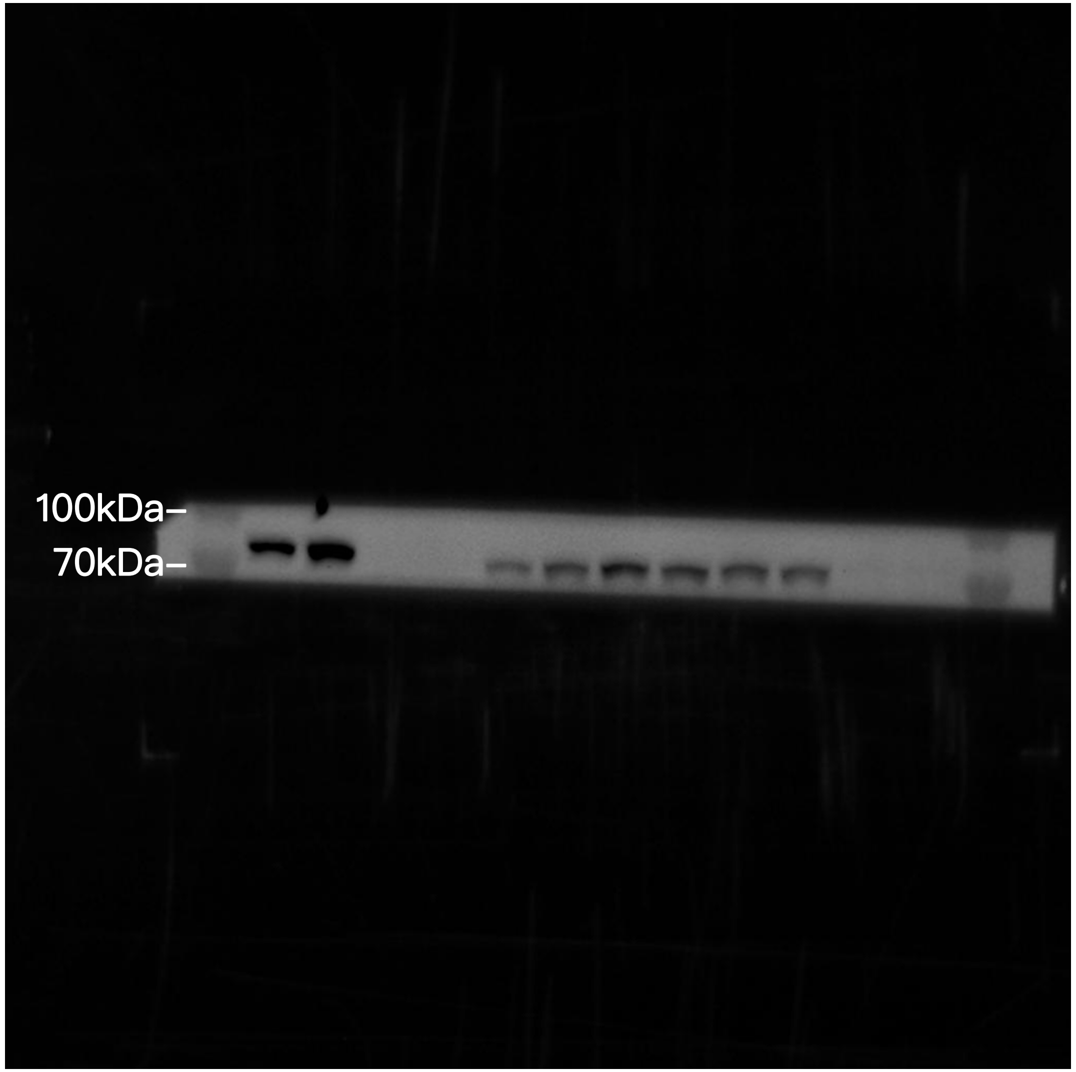

Figure 6E

other 2 Samples

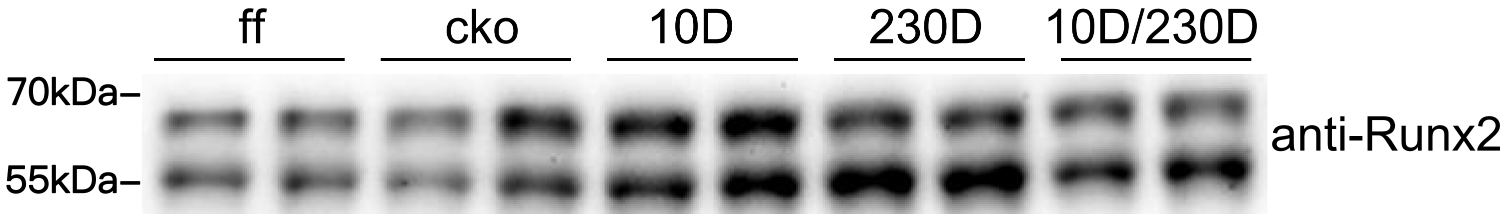

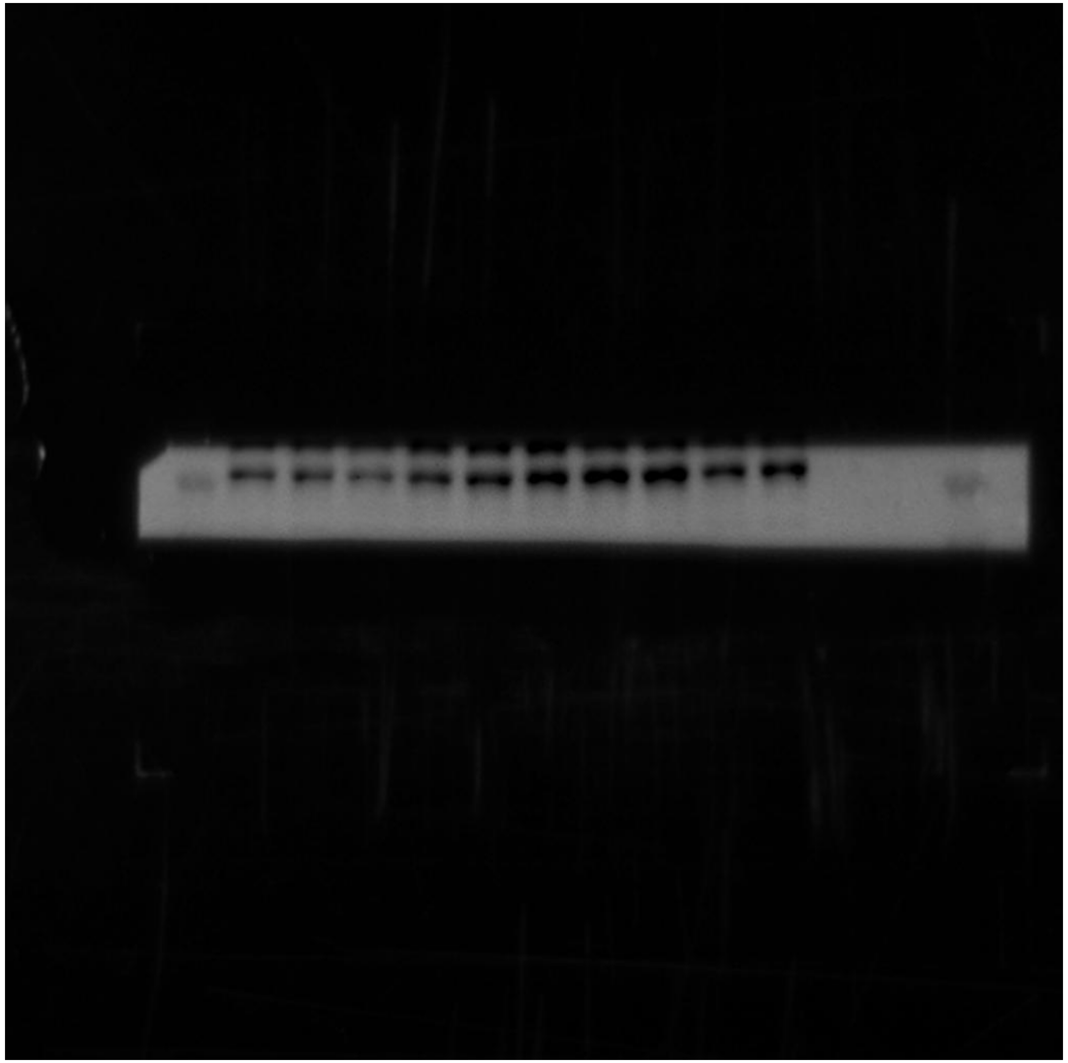

Figure 6G

Figure 6G

Show in Figure

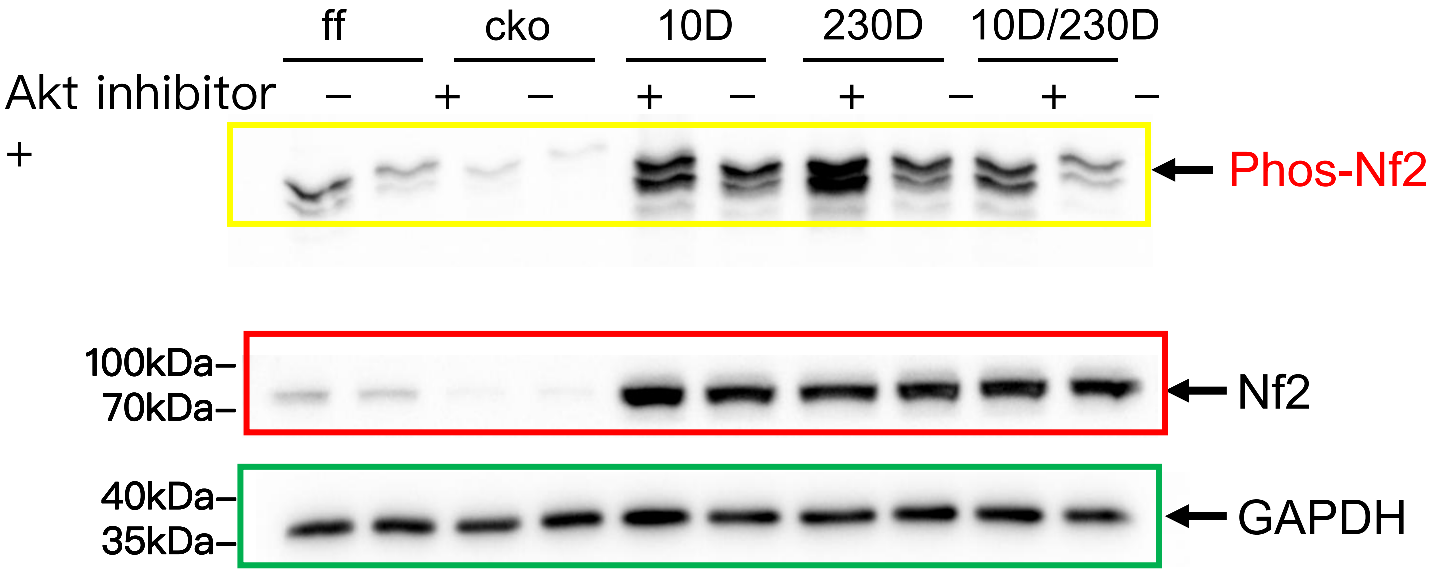

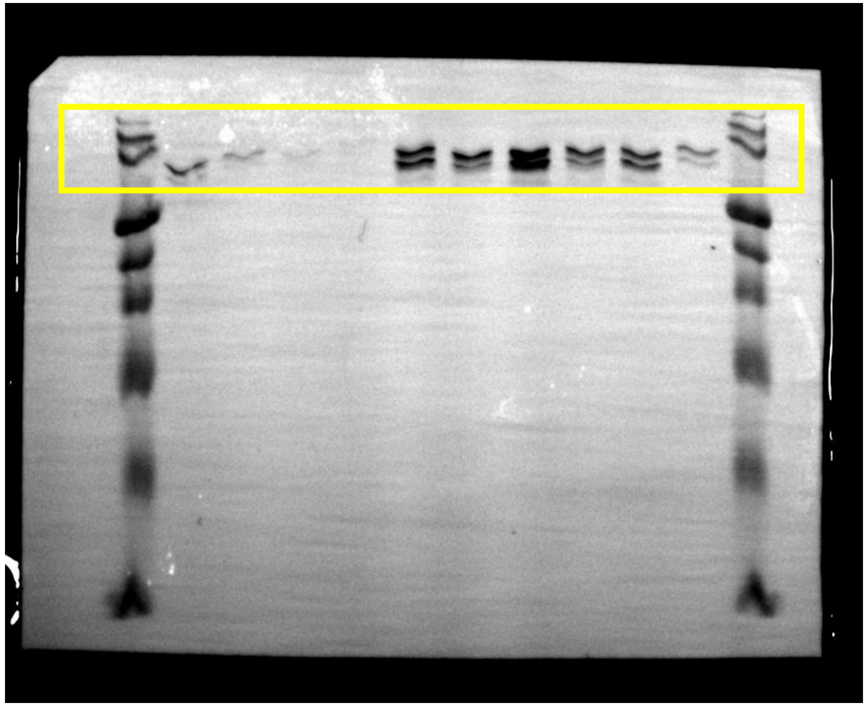

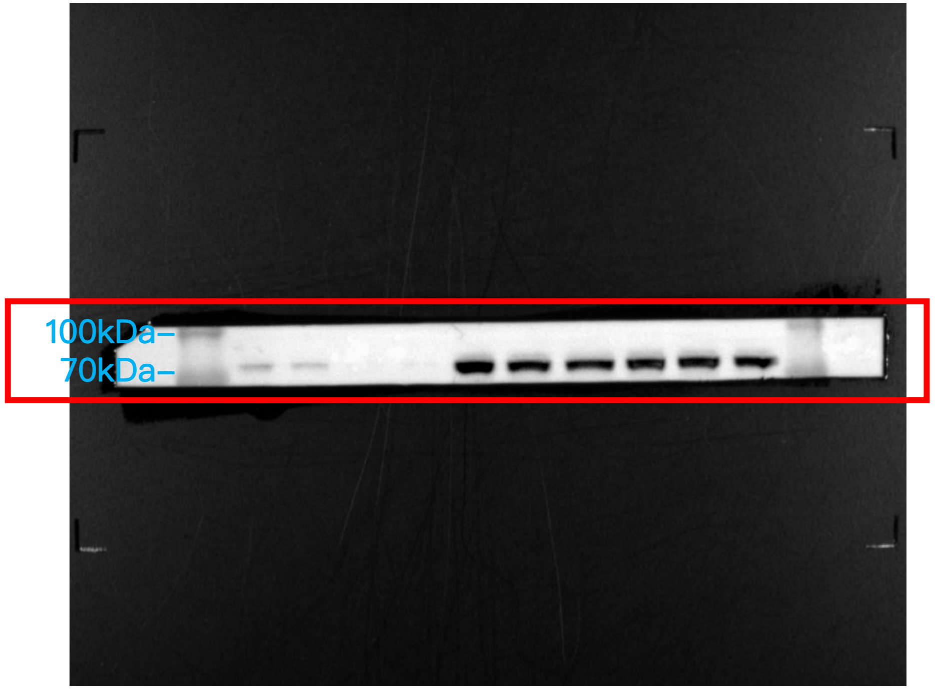

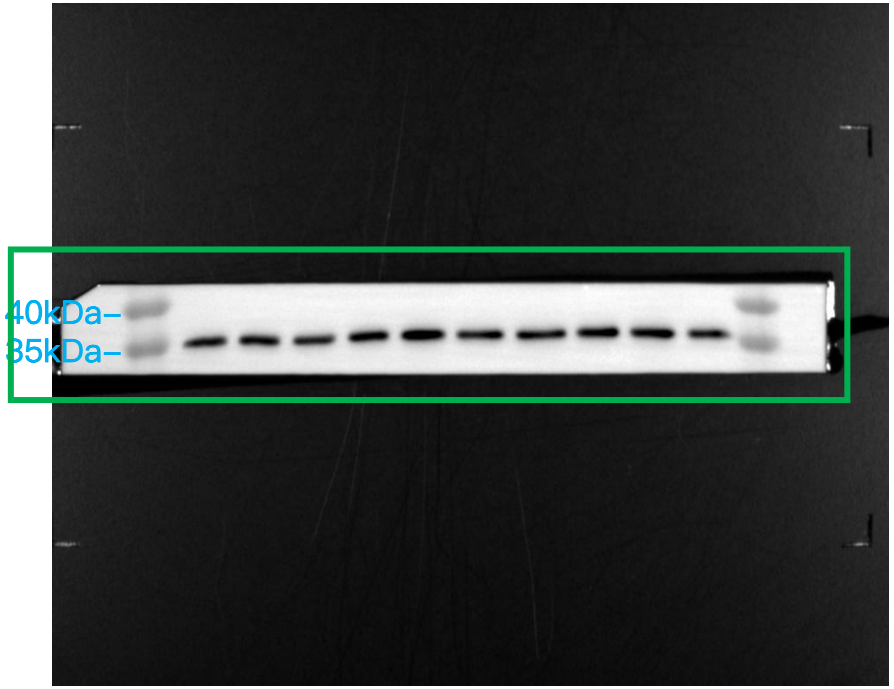

Figure 6G

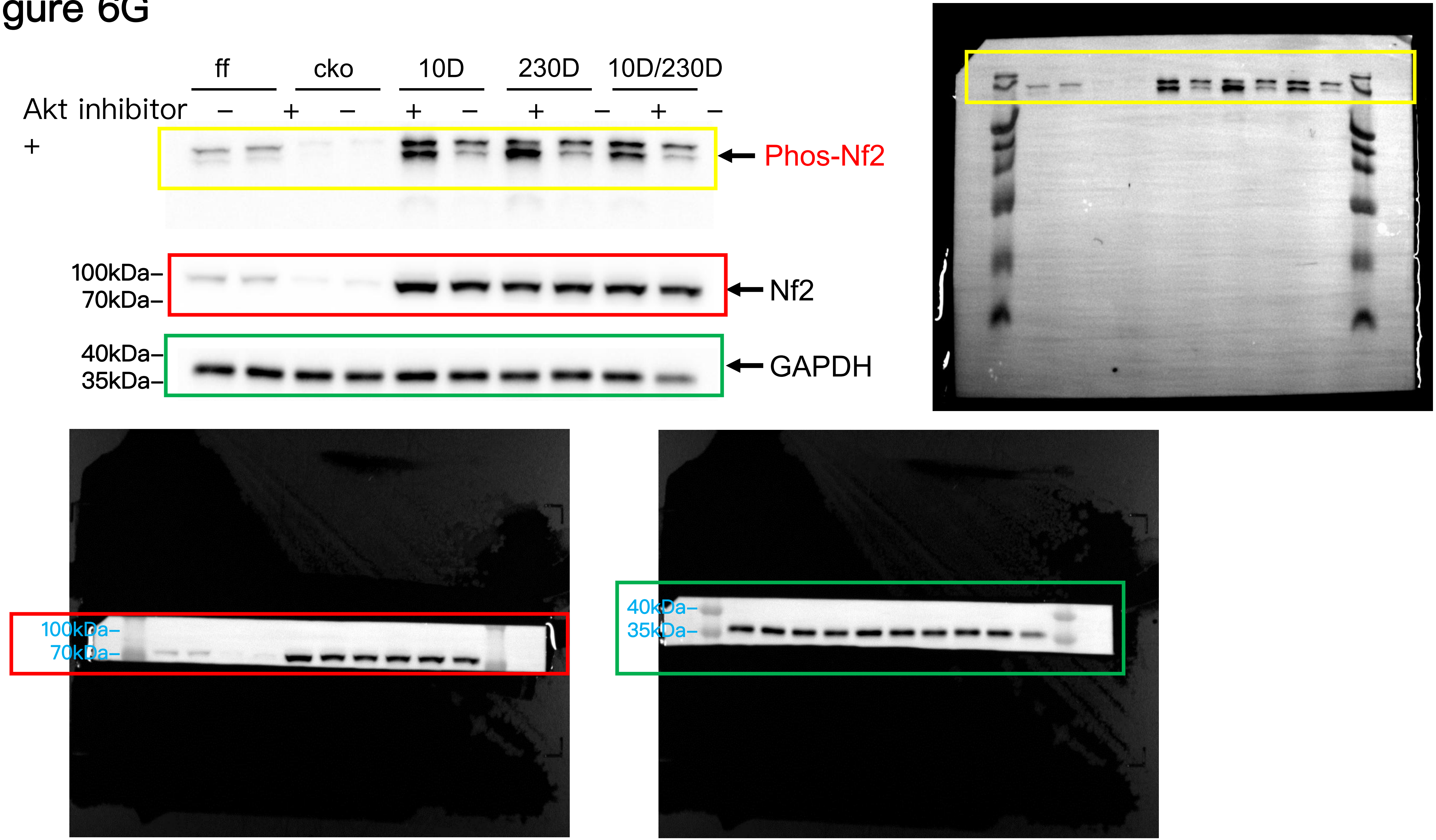

ff
cko
10D
230D
10D/230D
Akt inhibitor
− + − + − + − + − +
Phos-Nf2
100kDa–
70kDa–
Nf2
40kDa–
35kDa–
GAPDH
100kDa–
70kDa–
40kDa–
35kDa–

Figure 6G

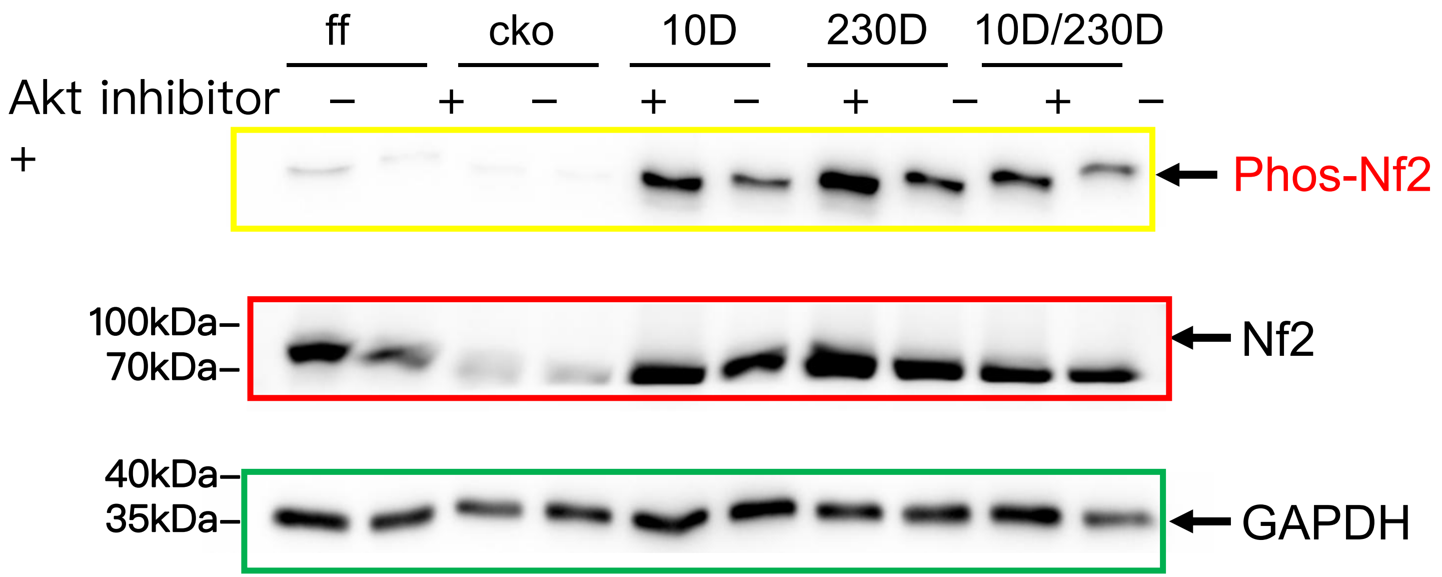
ff
cko
10D
230D
10D/230D
Akt inhibitor
+
− + − + − + − + −
Phos-Nf2
100kDa–
70kDa–
Nf2
40kDa–
35kDa–
GAPDH

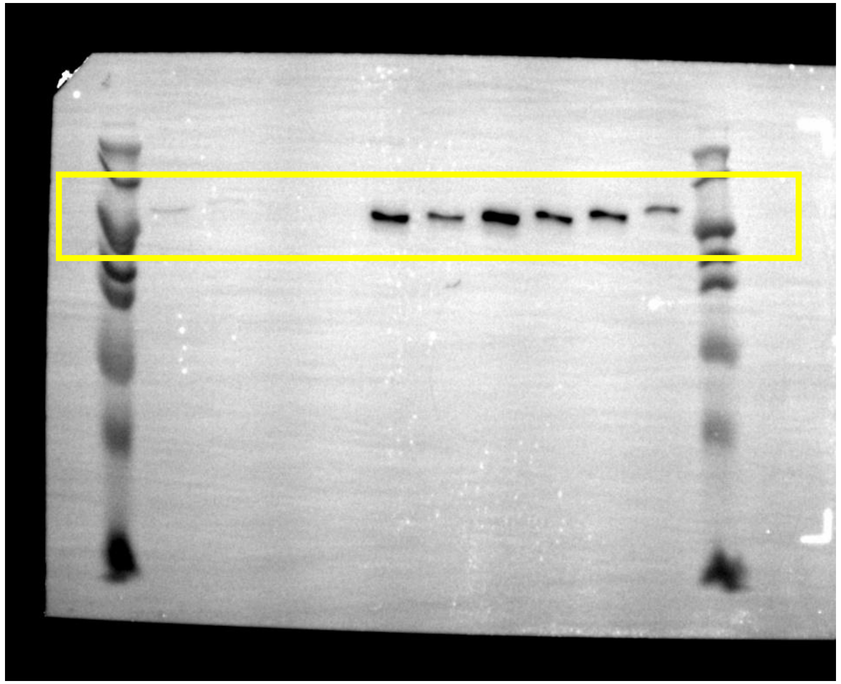

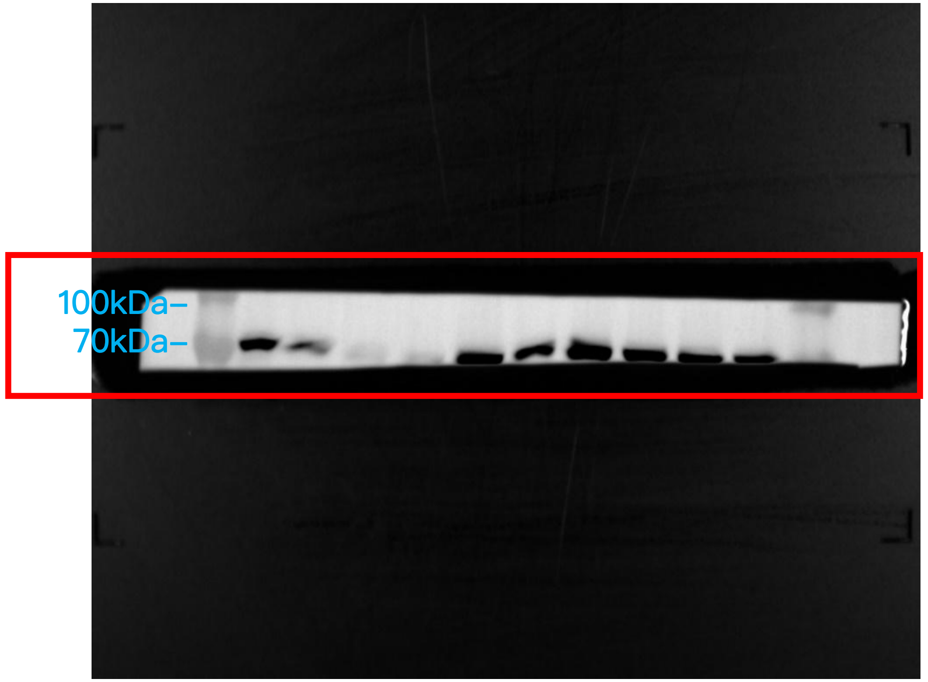
100kDa–
70kDa–

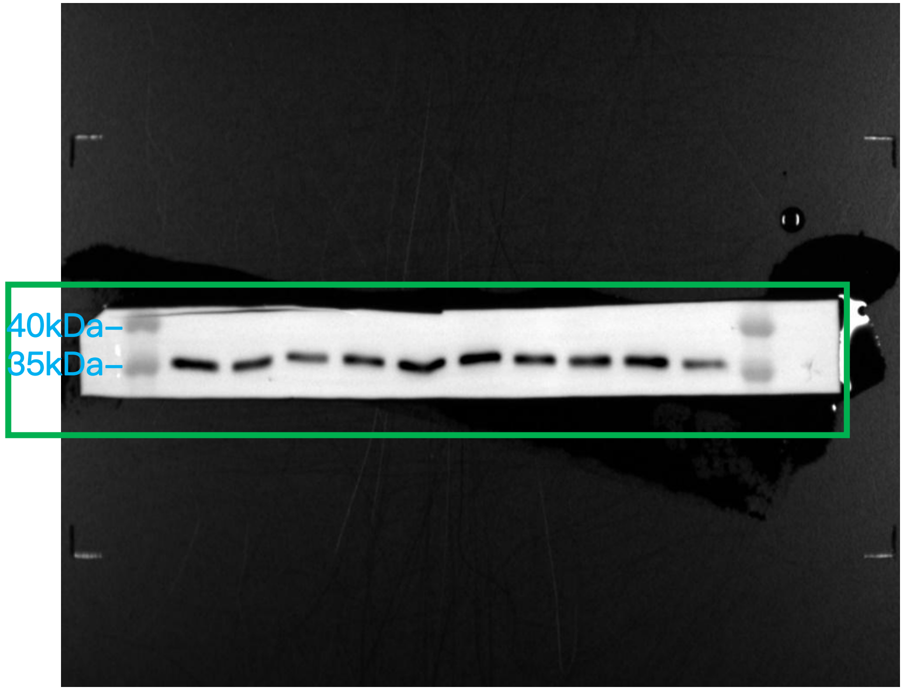
40kDa–
35kDa–

Figure 6G

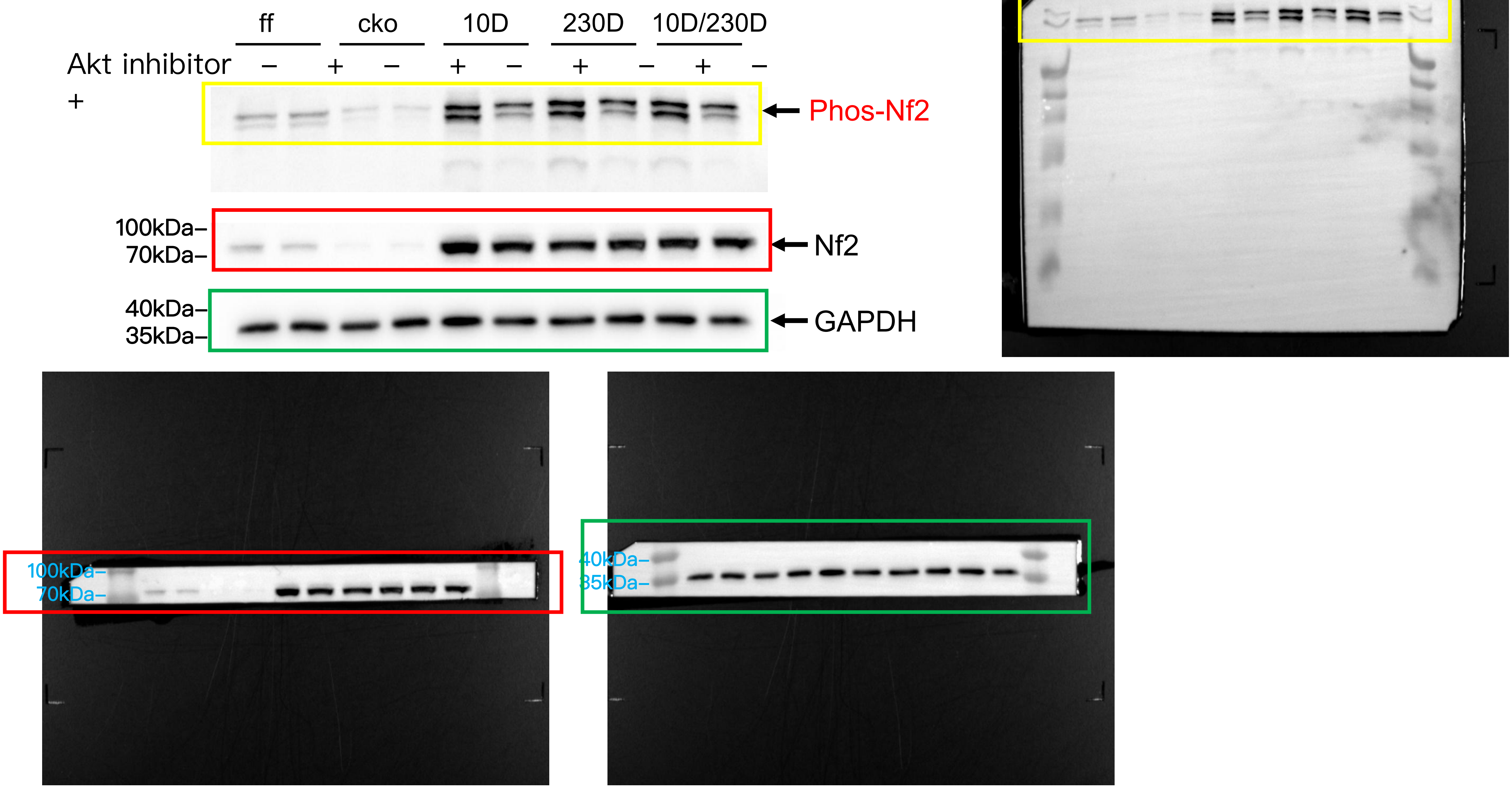

ff
cko
10D
230D
10D/230D
Akt inhibitor
–
+
–
+
–
+
–
+
–
+
Phos-Nf2
100kDa–
70kDa–
Nf2
40kDa–
35kDa–
GAPDH
100kDa–
70kDa–
40kDa–
35kDa–

Figure 6I–J

Figure 6I

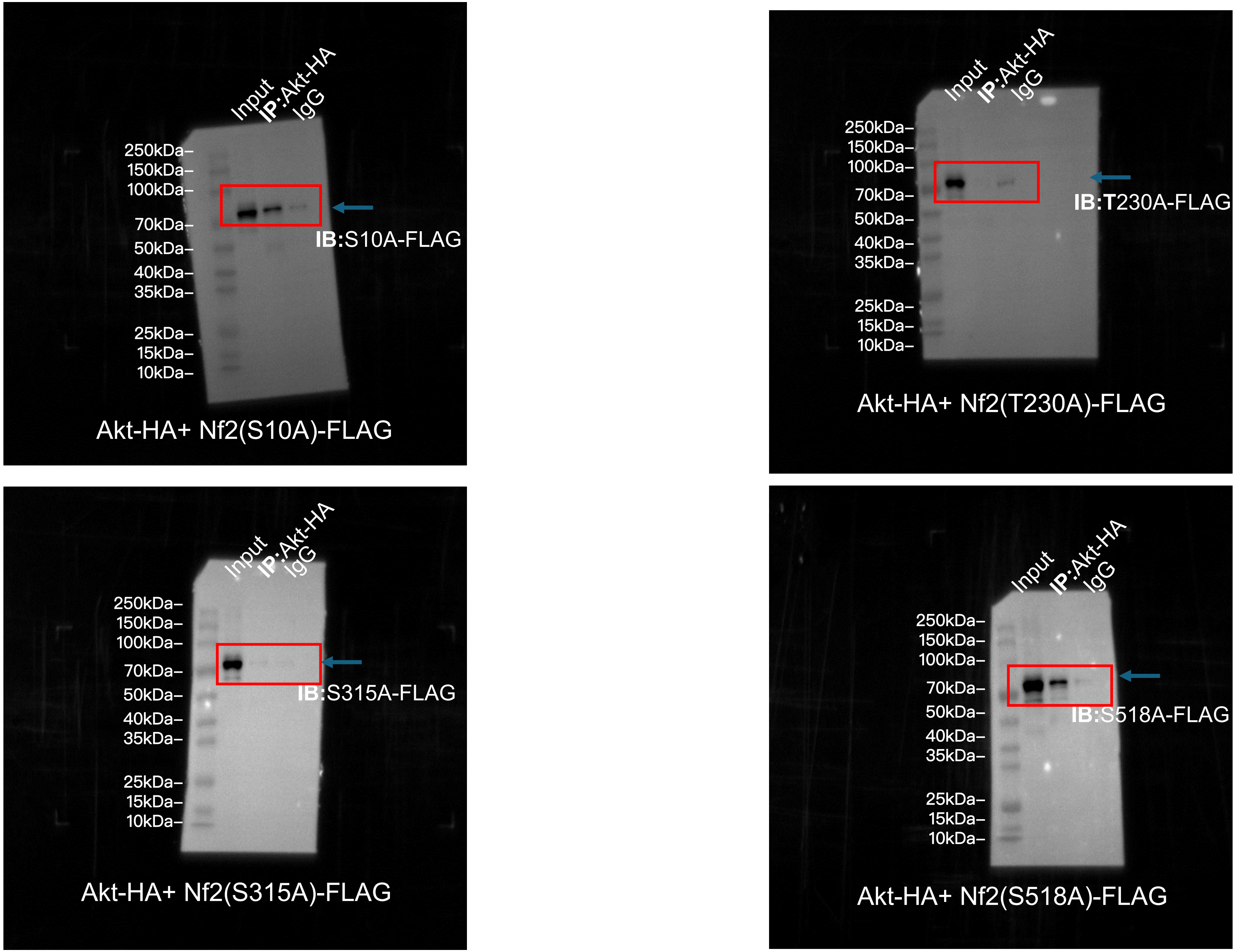
Input
IP:Akt-HA
IgG
250kDa–
150kDa–
100kDa–
70kDa–
50kDa–
40kDa–
35kDa–
25kDa–
15kDa–
10kDa–
IB:S10A-FLAG
Akt-HA+ Nf2(S10A)-FLAG
Input
IP:Akt-HA
IgG
250kDa–
150kDa–
100kDa–
70kDa–
50kDa–
40kDa–
35kDa–
25kDa–
15kDa–
10kDa–
IB:T230A-FLAG
Akt-HA+ Nf2(T230A)-FLAG
Input
IP:Akt-HA
IgG
250kDa–
150kDa–
100kDa–
70kDa–
50kDa–
40kDa–
35kDa–
25kDa–
15kDa–
10kDa–
IB:S315A-FLAG
Akt-HA+ Nf2(S315A)-FLAG
Input
IP:Akt-HA
IgG
250kDa–
150kDa–
100kDa–
70kDa–
50kDa–
40kDa–
35kDa–
25kDa–
15kDa–
10kDa–
IB:S518A-FLAG
Akt-HA+ Nf2(S518A)-FLAG

Figure 6J

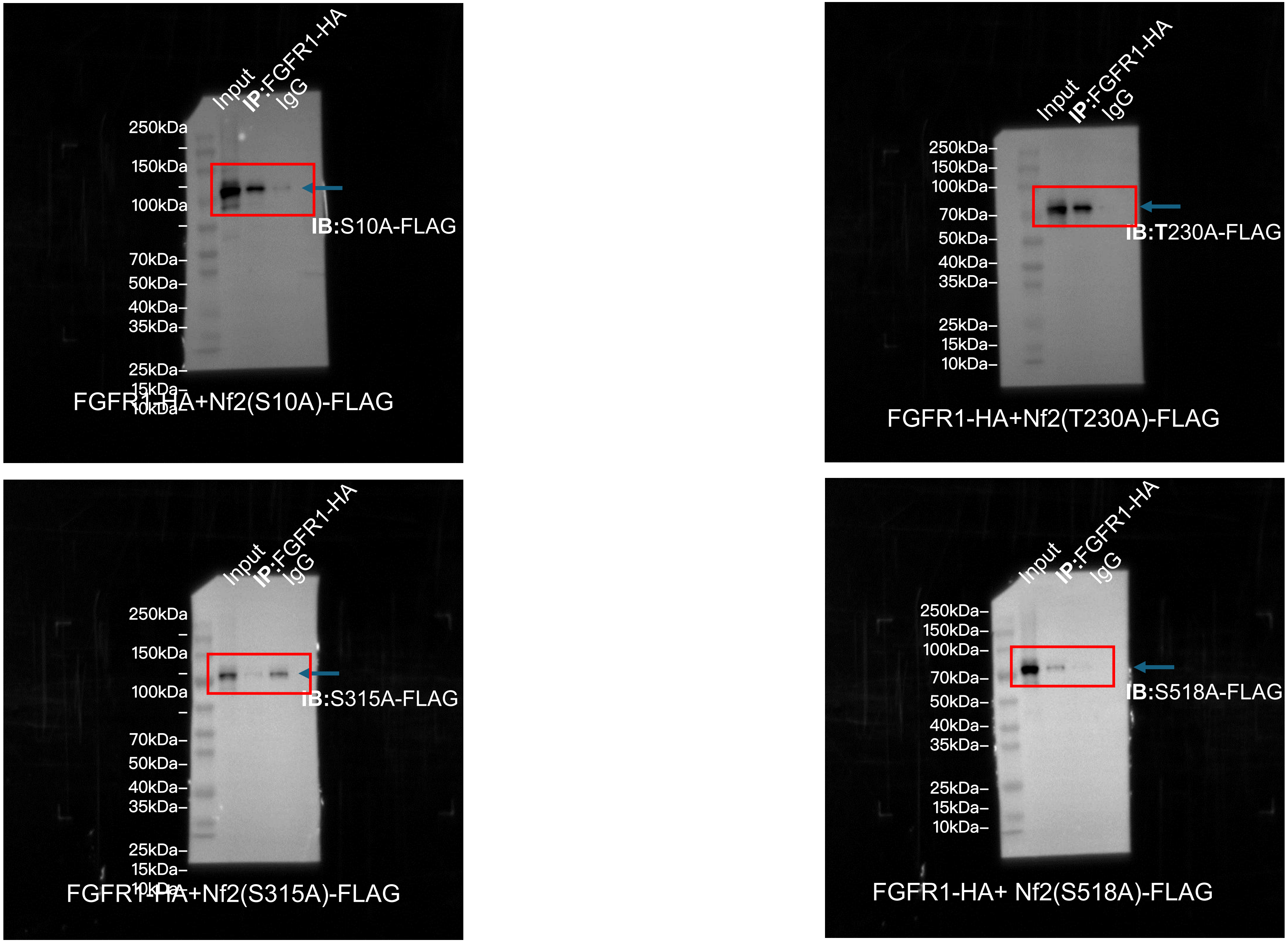
Input
IP:FGFR1-HA
IgG
250kDa
150kDa
100kDa
70kDa
50kDa
40kDa
35kDa
25kDa
15kDa
10kDa
IB:S10A-FLAG
FGFR1-HA+Nf2(S10A)-FLAG
IB:T230A-FLAG
FGFR1-HA+Nf2(T230A)-FLAG
IB:S315A-FLAG
FGFR1-HA+Nf2(S315A)-FLAG
IB:S518A-FLAG
FGFR1-HA+ Nf2(S518A)-FLAG

Supplemental data

Figure S2C

Figure S2C

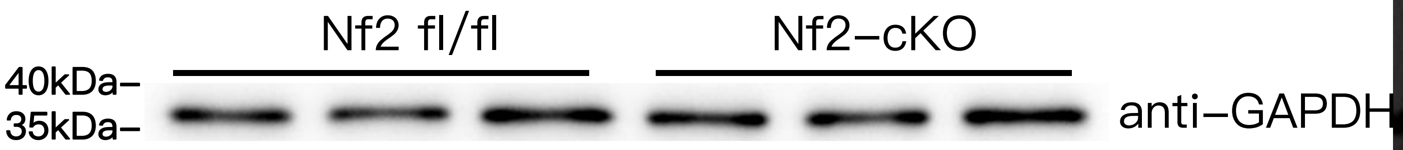

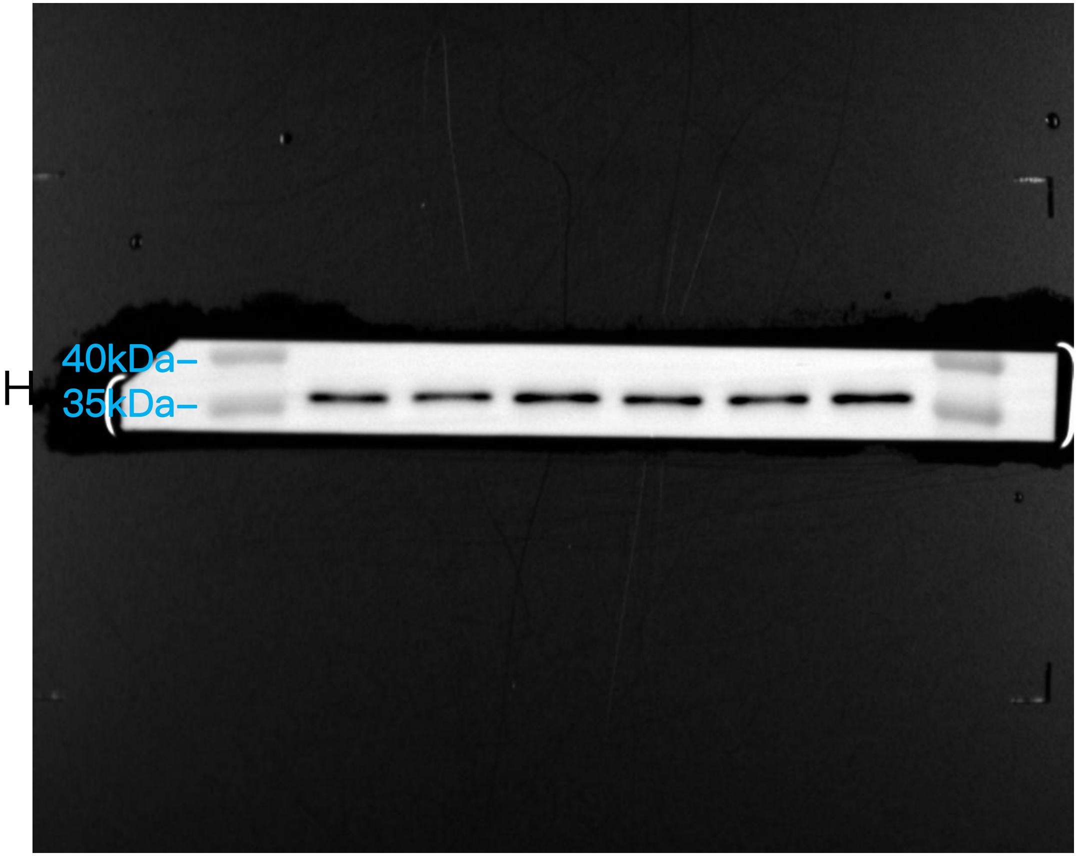

Figure S2C

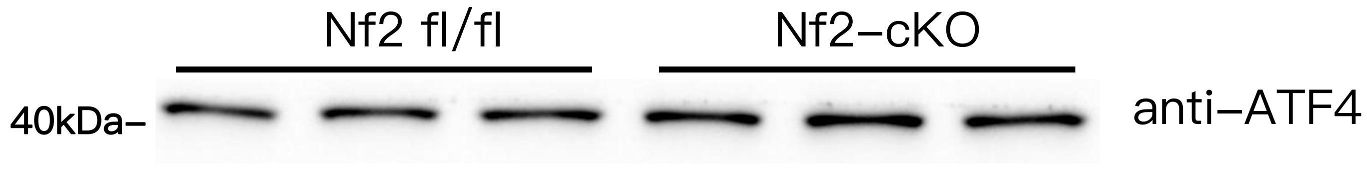

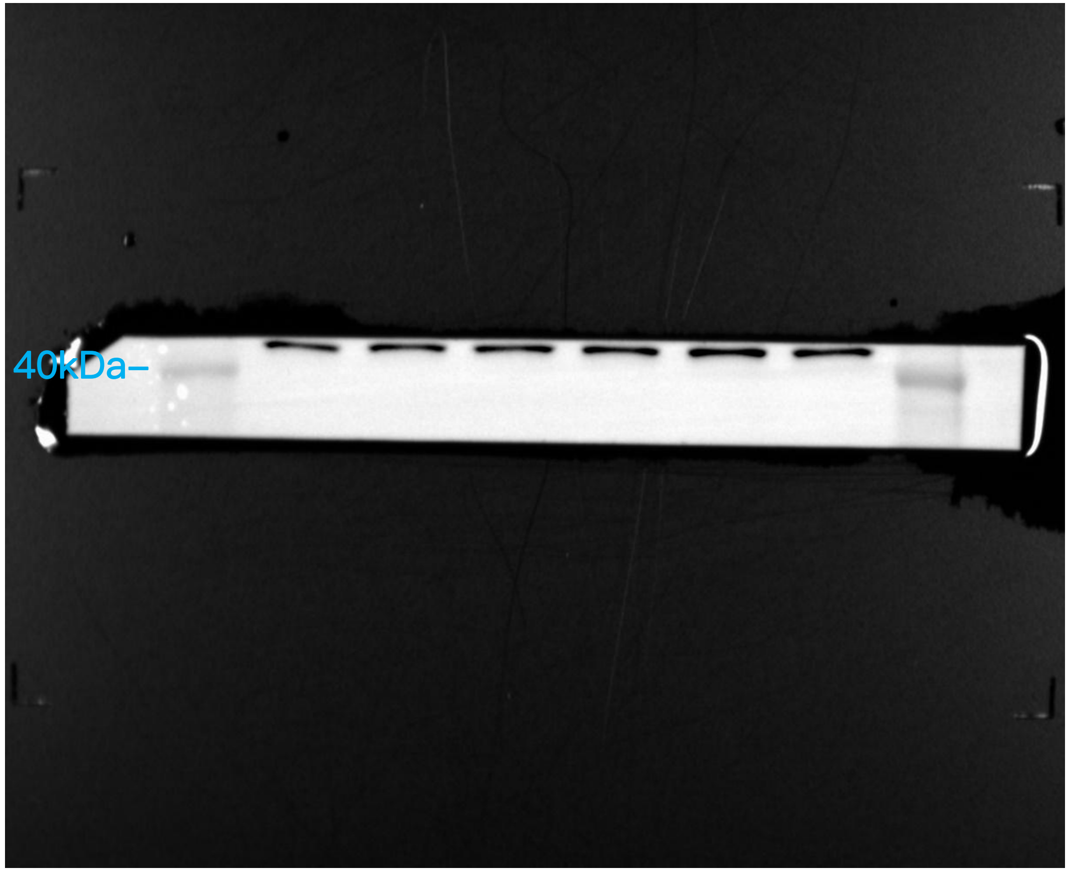

Figure S2C

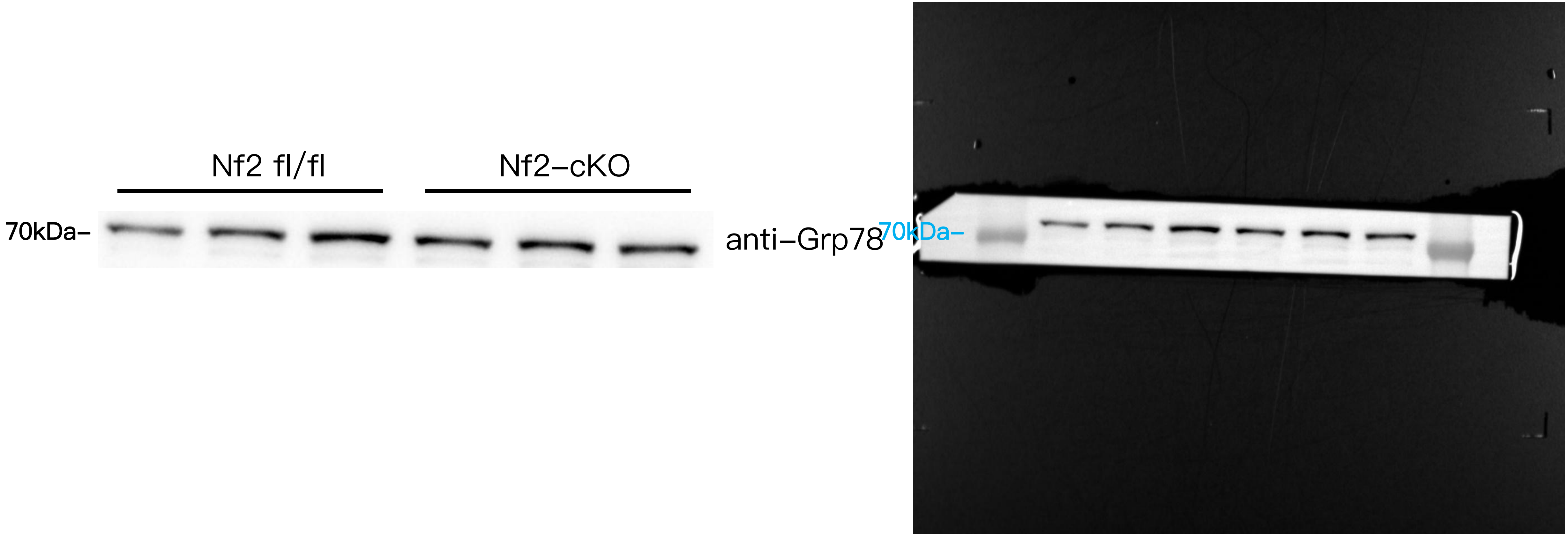

Figure S3A–B

Figure S3A–B

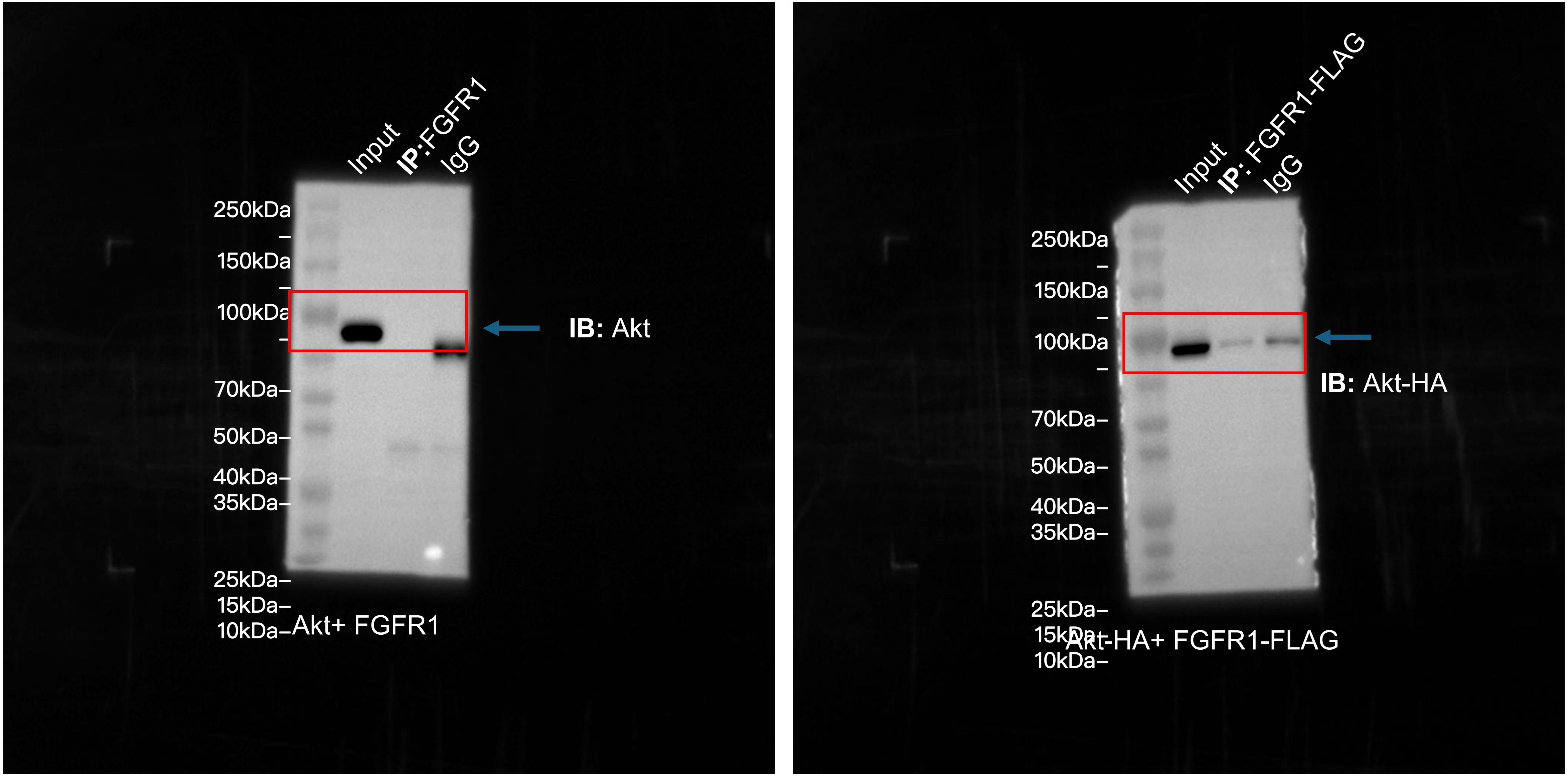
Input
IP:FGFR1
IgG
250kDa
150kDa
100kDa
70kDa
50kDa
40kDa
35kDa
25kDa
15kDa
10kDa
IB: Akt
Akt+ FGFR1
Input
IP: FGFR1-FLAG
IgG
250kDa
150kDa
100kDa
70kDa
50kDa
40kDa
35kDa
25kDa
15kDa
10kDa
IB: Akt-HA
Akt-HA+ FGFR1-FLAG

Figure
S3C

Figure S3C

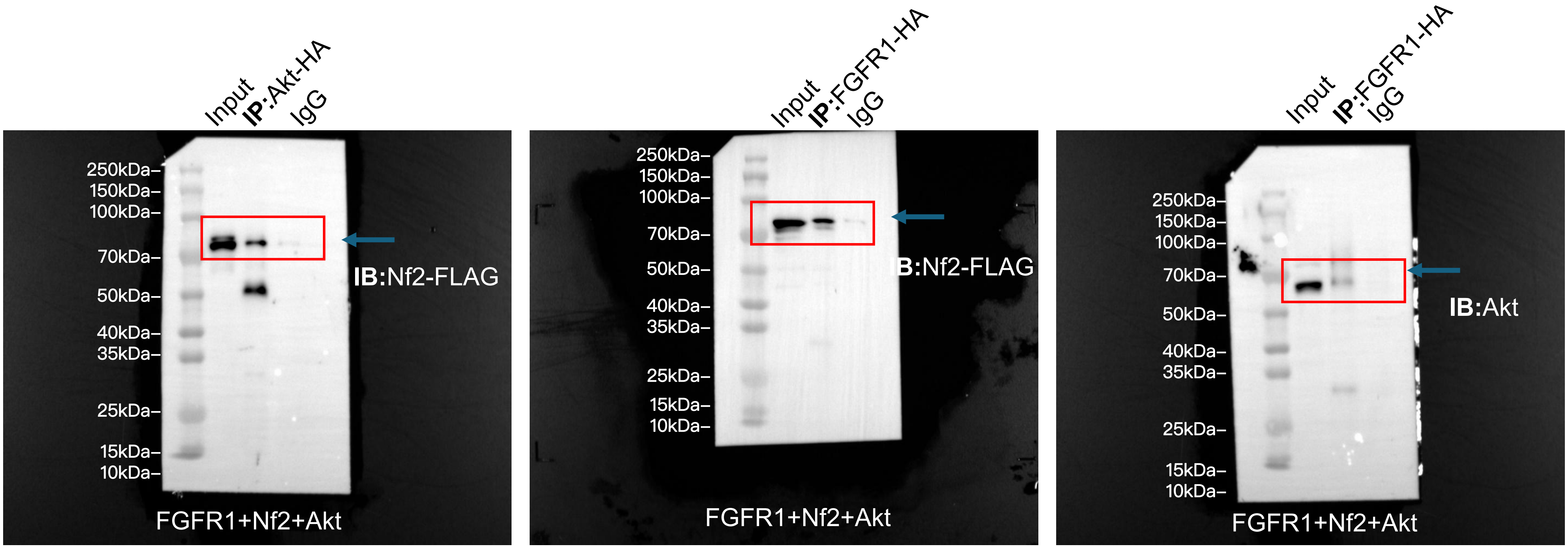
Input
IP:Akt-HA
IgG
250kDa–
150kDa–
100kDa–
70kDa–
50kDa–
40kDa–
35kDa–
25kDa–
15kDa–
10kDa–
IB:Nf2-FLAG
FGFR1+Nf2+Akt
Input
IP:FGFR1-HA
IgG
250kDa–
150kDa–
100kDa–
70kDa–
50kDa–
40kDa–
35kDa–
25kDa–
15kDa–
10kDa–
IB:Nf2-FLAG
FGFR1+Nf2+Akt
Input
IP:FGFR1-HA
IgG
250kDa–
150kDa–
100kDa–
70kDa–
50kDa–
40kDa–
35kDa–
25kDa–
15kDa–
10kDa–
IB:Akt
FGFR1+Nf2+Akt

Figure
S3D

Figure S3D

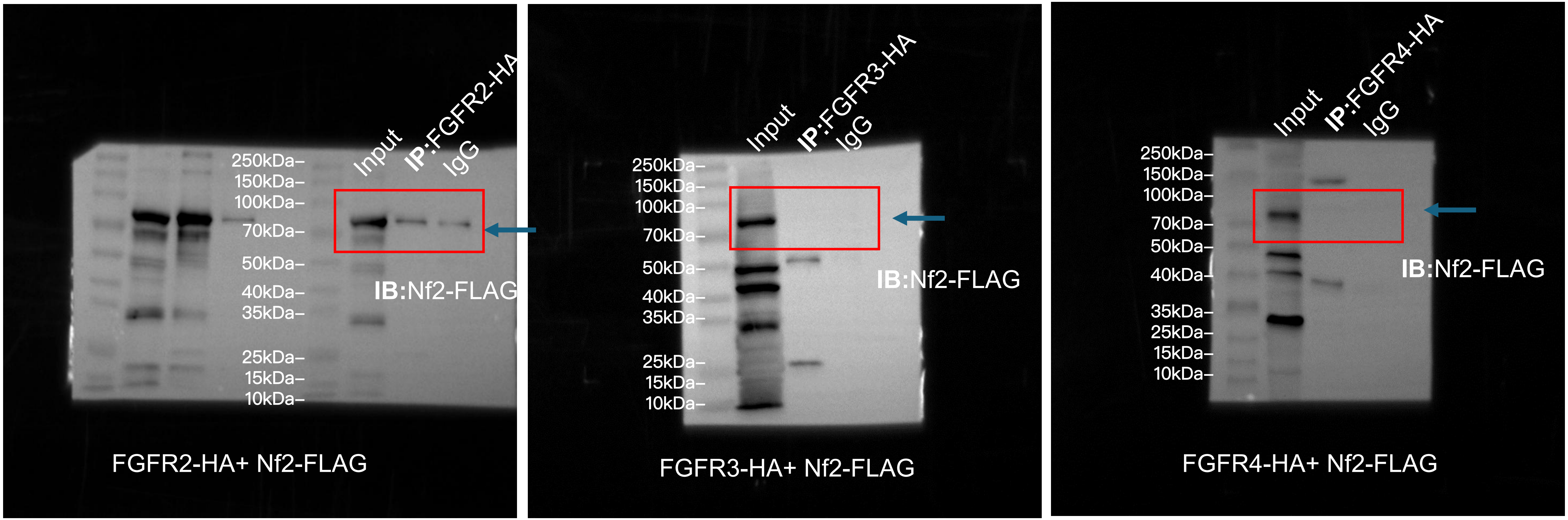
Input
IP:FGFR2-HA
IgG
250kDa–
150kDa–
100kDa–
70kDa–
50kDa–
40kDa–
35kDa–
25kDa–
15kDa–
10kDa–
IB:Nf2-FLAG
FGFR2-HA+ Nf2-FLAG
Input
IP:FGFR3-HA
IgG
250kDa–
150kDa–
100kDa–
70kDa–
50kDa–
40kDa–
35kDa–
25kDa–
15kDa–
10kDa–
IB:Nf2-FLAG
FGFR3-HA+ Nf2-FLAG
Input
IP:FGFR4-HA
IgG
250kDa–
150kDa–
100kDa–
70kDa–
50kDa–
40kDa–
35kDa–
25kDa–
15kDa–
10kDa–
IB:Nf2-FLAG
FGFR4-HA+ Nf2-FLAG

Figure S3D

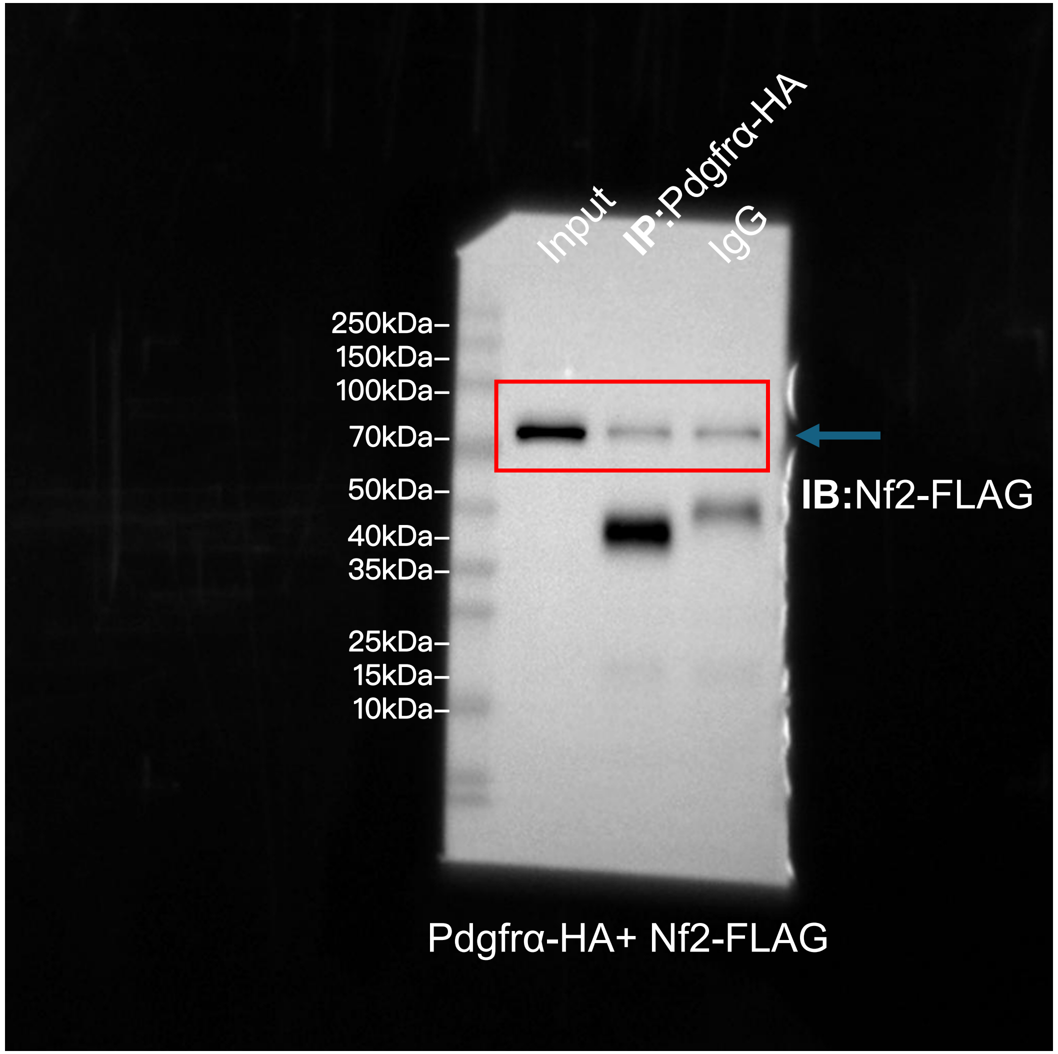

Input
IP:Pdgfrα-HA
IgG
250kDa
150kDa
100kDa
70kDa
50kDa
40kDa
35kDa
25kDa
15kDa
10kDa
IB:Nf2-FLAG
Pdgfrα-HA+ Nf2-FLAG

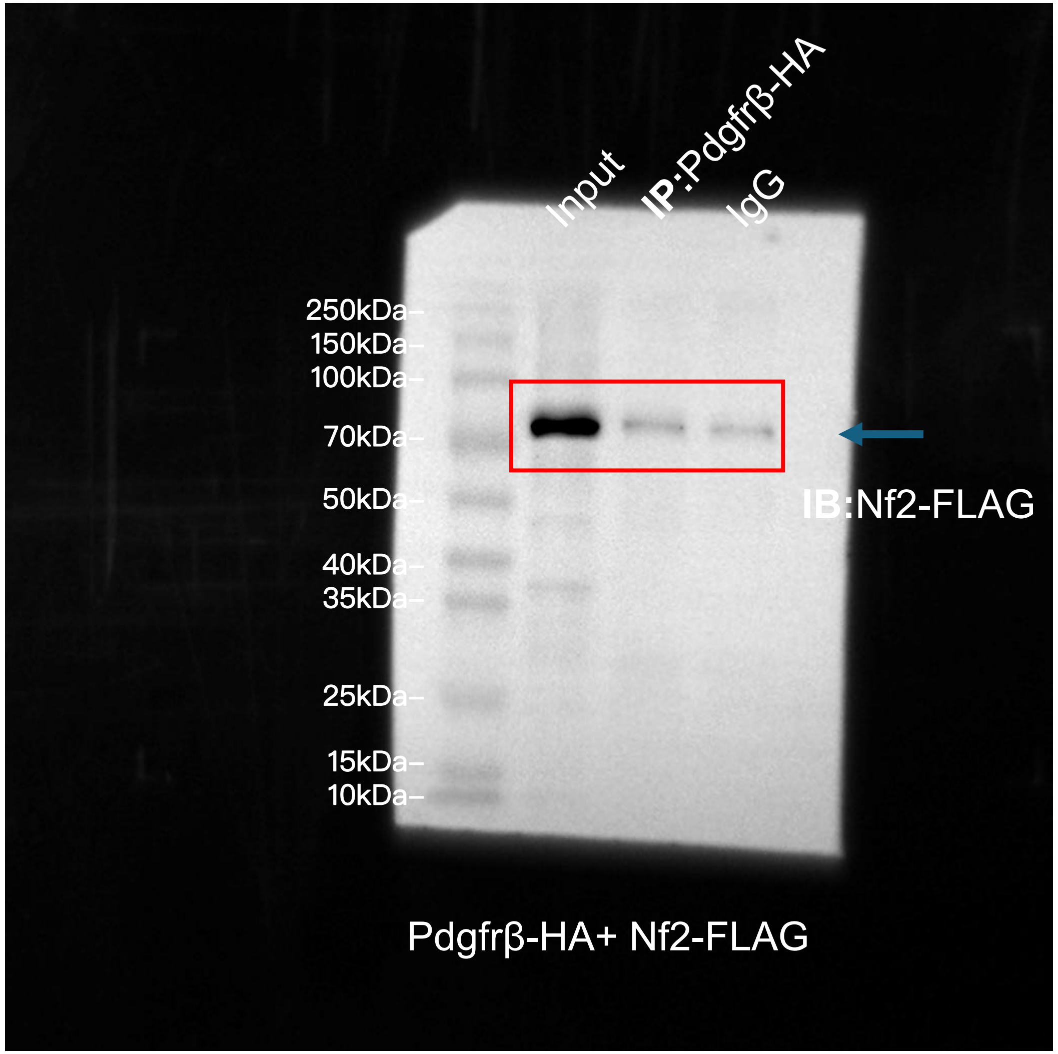

Input
IP:Pdgfrβ-HA
IgG
250kDa
150kDa
100kDa
70kDa
50kDa
40kDa
35kDa
25kDa
15kDa
10kDa
IB:Nf2-FLAG
Pdgfrβ-HA+ Nf2-FLAG

Figure
S3E

Figure S3E

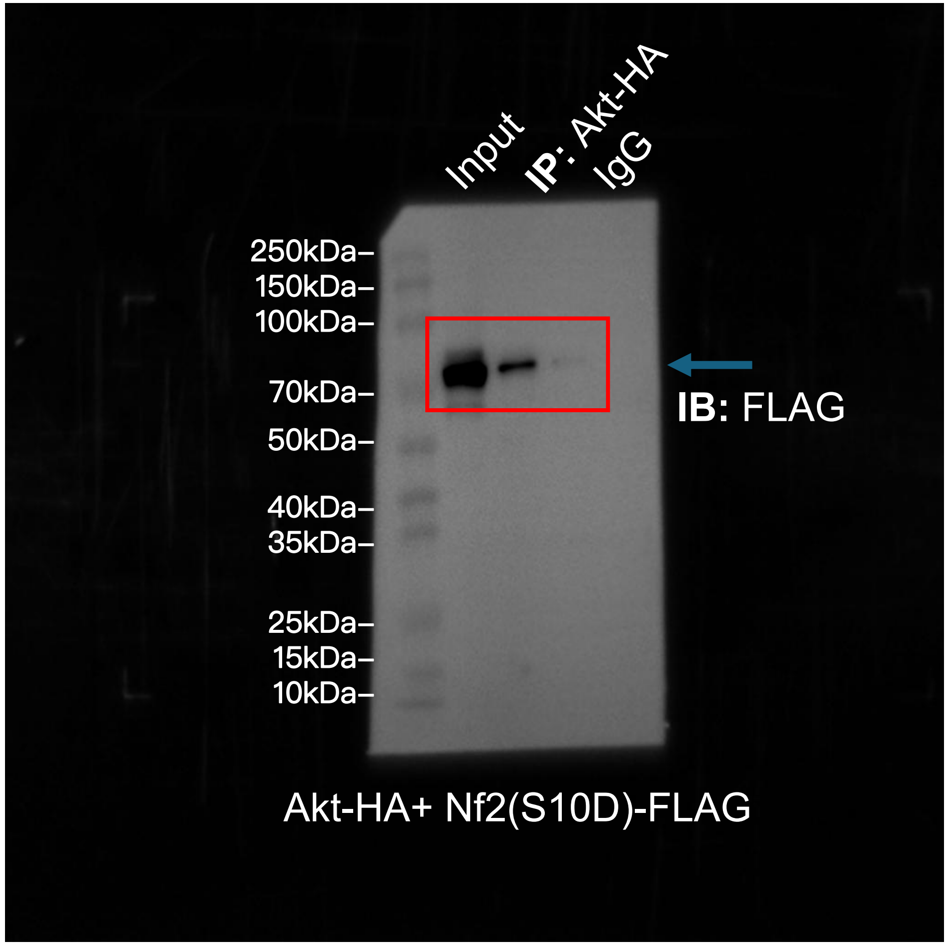

Input
IP: Akt-HA
IgG
250kDa–
150kDa–
100kDa–
70kDa–
50kDa–
40kDa–
35kDa–
25kDa–
15kDa–
10kDa–
IB: FLAG
Akt-HA+ Nf2(S10D)-FLAG

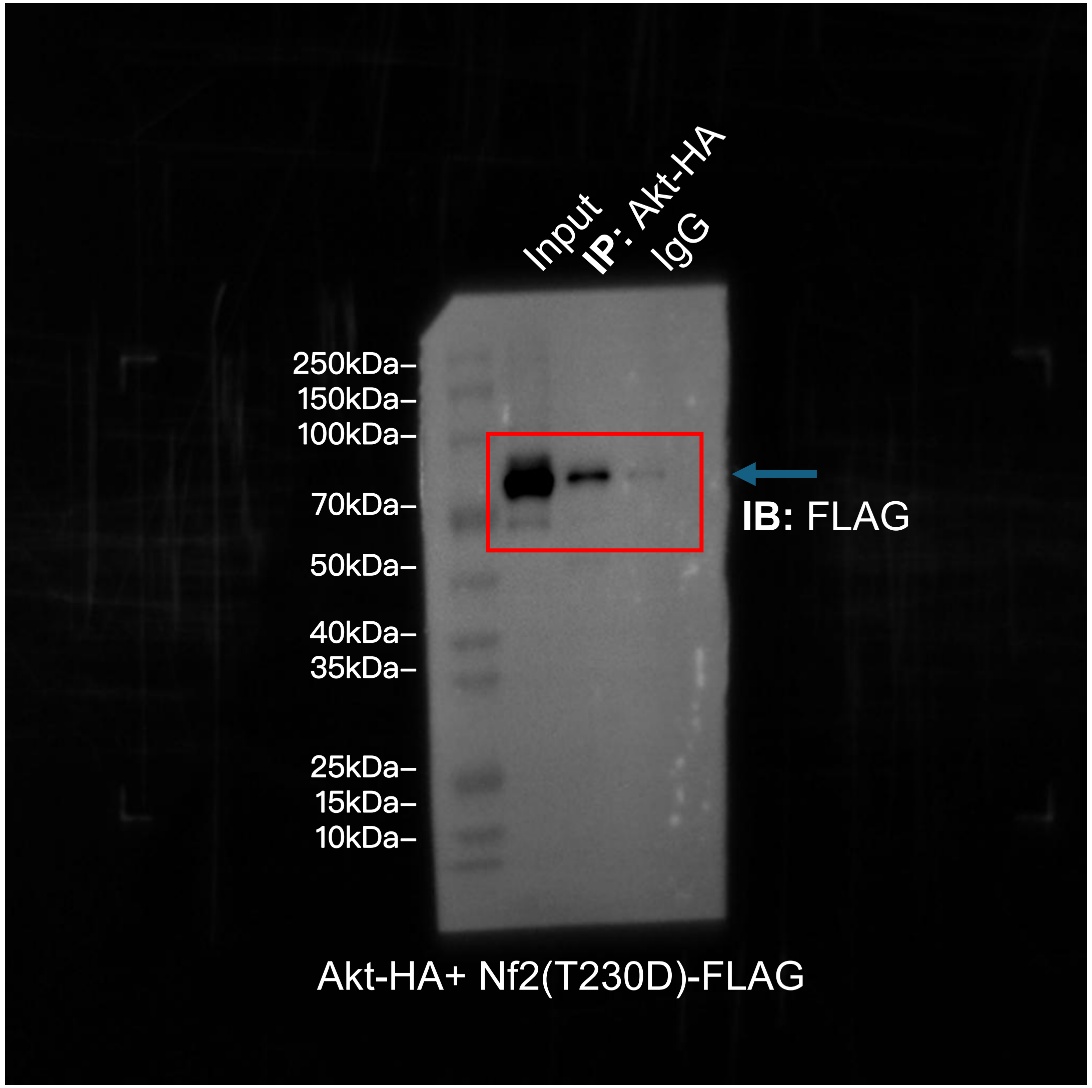

Input
IP: Akt-HA
IgG
250kDa–
150kDa–
100kDa–
70kDa–
50kDa–
40kDa–
35kDa–
25kDa–
15kDa–
10kDa–
IB: FLAG
Akt-HA+ Nf2(T230D)-FLAG

Figure S3F

Figure S3F

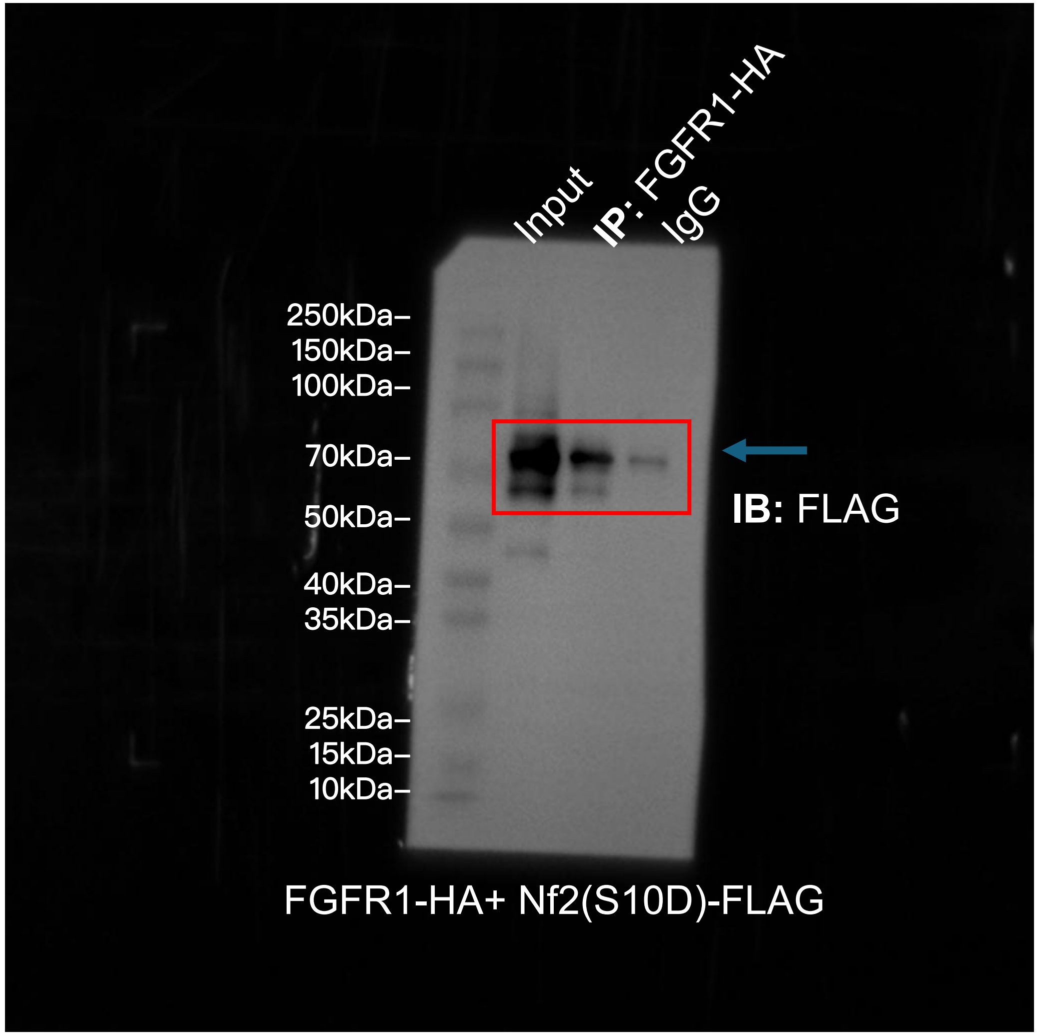

Input
IP: FGFR1-HA
IgG
250kDa–
150kDa–
100kDa–
70kDa–
50kDa–
40kDa–
35kDa–
25kDa–
15kDa–
10kDa–
IB: FLAG
FGFR1-HA+ Nf2(S10D)-FLAG

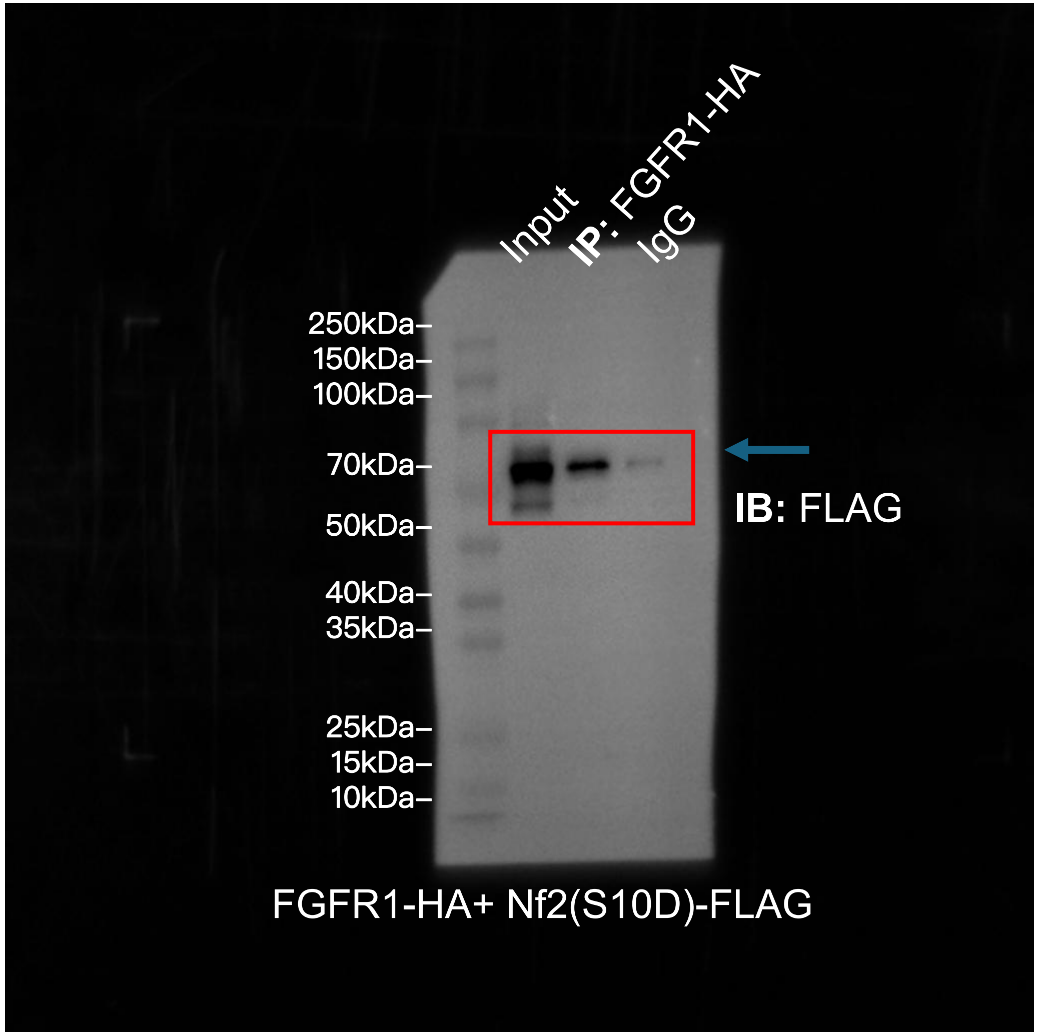

Input
IP: FGFR1-HA
IgG
250kDa–
150kDa–
100kDa–
70kDa–
50kDa–
40kDa–
35kDa–
25kDa–
15kDa–
10kDa–
IB: FLAG
FGFR1-HA+ Nf2(S10D)-FLAG
